# Inverse-Probability-Weighted Kernel Estimation of Regression Derivatives Under Missing-at-Random Responses for Stationary Ergodic Processes

**DOI:** 10.3390/e28090962

**Published:** 2026-08-27

**Authors:** Salim Bouzebda, Sultana Didi

**Affiliations:** 1 LMAC (Laboratory of Applied Mathematics of Compiègne), Université de Technologie de Compiègne, CS 60 319, 60 203 Compiègne, France; salim.bouzebda@utc.fr; 2 Department of Statistics and Operations Research, College of Sciences, Qassim University, P.O. Box 6688, Buraydah 51452, Saudi Arabia

**Keywords:** kernel derivative estimation, inverse-probability weighting, missing at random, stationary ergodic processes, nonparametric regression, density derivatives, conditional distribution estimation, uniform strong convergence, asymptotic normality, AMISE, martingale approximation, simulation, 62G07, 62G08, 62G05, 62G20, 62H05, 60G42, 60G46

## Abstract

This paper develops asymptotic theory for kernel estimation of density-weighted conditional functionals and regression derivatives when responses are missing at random (MAR) and the observations form a strictly stationary ergodic process. Sequential MAR and positivity identify the complete-data conditional target through an inverse-probability-weighted pseudo-response, while the fully observed covariate density and its derivatives are estimated without unnecessary response weighting. A martingale-predictable decomposition yields uniform almost-sure rates, pointwise Gaussian limits, variance expansions, studentization, and AMISE results under explicit projective/maximal, conditional-moment, conditional-density, and variance-stabilization conditions. These quantitative assumptions are additional to stationarity and ergodicity: the results are not asserted for arbitrary stationary ergodic sequences. Exact-quotient and multi-index identities transfer the primitive-estimator theory to regression derivatives, and feasible propensity estimation contributes an explicit additional remainder. Monte Carlo experiments show that stronger dependence, weak response probabilities, higher derivative order, propensity misspecification, and smoothing bias can materially degrade finite-sample performance; undersmoothing improves centring but need not eliminate coverage distortion at moderate sample sizes.

## 1. Introduction

Kernel smoothing is one of the principal constructive methods of nonparametric statistics. Its modern development originates in the early works of [1,2,3] and has subsequently produced a comprehensive theory for the estimation of densities, regression functions, conditional distributions, and related functionals. Classical accounts of the subject, including consistency, risk, bandwidth selection, and implementation, may be found in [4,5,6,7,8,9,10,11,12]. The appeal of kernel procedures lies not only in their analytical tractability but also in their ability to estimate local geometric features without imposing a finite-dimensional model.

The estimation of derivatives is substantially more delicate than estimation of the underlying function. Differentiation magnifies high-frequency fluctuations, changes the effective stochastic normalization, and increases the order of smoothness required for bias control. Density derivatives encode monotonicity, curvature, critical points, ridges, modal structure, and local concentration. They enter, for example, derivative-based mode testing [13], the leading bias of ordinary density estimators and the associated bandwidth problem [10,14], and refined nearest-neighbour information calculations [15]. First-order density derivatives are also central to gradient-based modal analysis and mean-shift clustering; see [16,17,18].

The mathematical literature on kernel density-derivative estimation is extensive and includes foundational and subsequent contributions by [19,20,21,22,23,24,25,26,27,28,29,30,31,32]. These works make clear that derivative estimation is not a routine corollary of function estimation: kernel order, derivative order, boundary behaviour, and bandwidth scaling must be handled explicitly.

Derivative estimation is equally important in regression and conditional analysis. It appears in Fisher-information calculations, semiparametric parameter estimation, and hypothesis testing; see [33,34,35]. In local polynomial regression, the conditional bias, variance, and optimal bandwidth depend on derivatives of the regression function [36]. In local linear quantile regression, the relevant bias and bandwidth calculations involve derivatives of the conditional distribution function [37]. Regression derivatives also describe rates of change, acceleration, turning points, and local sensitivity, and they are used in growth-curve analysis [38], kidney function modelling [39], and spectroscopic analysis [40]. They further arise in confidence-band construction [41], plug-in bandwidth selection [42], and comparisons of regression curves [43].

Kernel *M*-estimation of regression derivatives was considered in [44], while derivative-based ideas also appear in modal regression and related conditional-mode methods; see [45,46,47,48,49]. Classical aspects of nonparametric regression estimation were developed in [50,51,52,53,54]. Nevertheless, compared with the large literature on estimating a regression function itself, the systematic theory of its derivatives remains less complete, particularly when the data are dependent or incompletely observed; recent related contributions include [55,56,57,58,59].

The references above were selected to represent the four strands needed for the present problem—classical kernel-derivative theory, nonparametric regression derivatives, missing-data identification/IPW, and dependent/ergodic asymptotics—rather than as an exhaustive bibliography. Their relevance is best understood through three methodological transitions: First, classical i.i.d. kernel-derivative arguments and standard mixing-based covariance bounds cannot be invoked from ergodicity alone; quantitative rates require separate control of the martingale fluctuation, the past-predictable component, and the conditional quadratic variation. Second, moving from complete responses to MAR changes identification and the second-moment structure: response-dependent numerators require inverse-probability correction, whereas the marginal covariate density remains fully observed. Third, moving from order-zero smoothing to derivative estimation changes the stochastic normalization, smoothness requirements, kernel moment conditions, and bias–variance balance. These three failures of direct transfer define the specific research gap addressed below. Under independence and π≡1, the leading rates and bandwidth orders reduce to their familiar complete-data kernel-derivative counterparts; the contribution here is the identification and probability-theoretic justification needed when missingness and dependent sampling are present.

A second and independent difficulty is created by missing responses. Missing outcomes occur in longitudinal studies through dropout and intermittent non-response, in biomedical monitoring through device or transmission failure, and in administrative data through incomplete linkage, delayed reporting, or confidentiality restrictions. A complete-case analysis may then estimate the conditional law of a selected subpopulation rather than the full-data target. The classical missing-data taxonomy distinguishes missing completely at random, missing at random, and missing not at random according to the conditional relation between the observation indicator and the unobserved response [60].

The present paper is concerned with missing at random. In its dependent-data form, the assumption is stated sequentially: conditional on the current covariate and the complete past, the current response-observation indicator is conditionally independent of the current unobserved response, and its conditional mean is a time-homogeneous propensity score. This formulation is stronger than a purely contemporaneous MAR statement, but it is the form needed to make the past-conditioned residuals in the martingale decomposition have a conditional mean of zero.

MCAR is contained as the special case in which the propensity is constant. By contrast, under MNAR, the observation probability may still depend on the current unobserved response after conditioning on the covariate and the past; the basic IPW identification identity used here then fails without an additional selection, pattern-mixture, instrumental-variable, or sensitivity model. MAR is therefore chosen because it permits covariate-dependent non-response while preserving nonparametric identification by inverse probability weighting.

The interpretation and consequences of MAR, together with several methodological developments, are discussed in [61,62,63,64,65,66,67]. The practical advantages and robustness considerations associated with MAR-based corrections are also emphasized in [68].

The principal identification device is inverse-probability weighting, whose classical foundations include [69]. Let ξ denote the response indicator, and let

π(x)=P(ξ=1∣X=x)

be the propensity score. Under MAR and positivity, the pseudo-response

$$W\left(\psi \right)=\frac{\xi \psi (Y)}{\pi (X)}$$

satisfies

E{W(ψ)∣X=x}=E{ψ(Y)∣X=x}.

Thus, the observed-data law identifies the complete-data conditional functional through a weighted response. Related developments in inverse probability weighting, robustness, efficiency, and augmented procedures include [60,70,71].

The identification argument also determines the correct architecture of the kernel estimator. If

$$r\left(\psi ;x\right)=E\left\{\psi \left(Y\right)|X=x\right\}f_{X}\left(x\right),$$

then its derivative is estimated from the weighted pseudo-response, whereas the marginal density $f_{X}$ and its derivatives are estimated from all covariates. The latter are fully observed and, therefore, require no missingness weight. Here, $X_{i}\in R^{p}$, and *p* denotes the covariate dimension. We use a volume–bandwidth convention: $h_{n}$ is the local volume scale and $h_{n}^{1/p}$ is the corresponding isotropic coordinate bandwidth. For derivative order zero, the corresponding oracle ratio estimator is

(1) $${\hat{m}}_{n}^{\;\mathrm{IPW}}\left(\psi ;x,h_{n}\right)=\frac{\sum_{i=1}^{n}\frac{\xi_{i}\psi \left(Y_{i}\right)}{\pi (X_{i})}K\;\left(\frac{x-X_{i}}{h_{n}^{1/p}}\right)}{\sum_{i=1}^{n}K\;\left(\frac{x-X_{i}}{h_{n}^{1/p}}\right)},\;x\in I.$$

The denominator in (1) uses the full covariate sample. Weighting it by the observation indicator would be unnecessary for identification and would generally introduce avoidable Bernoulli variability.

The third difficulty addressed in this paper is temporal dependence. Much of nonparametric asymptotic theory is developed under independence or a mixing condition. Mixing assumptions provide useful quantitative approximations to independence, but they may be difficult to verify or may fail for processes with persistent dependence, discrete innovations, nonlinear recursions, or certain dynamical structures. Work on nonparametric estimation under ergodic sampling includes [72,73,74,75,76]. As noted in [77], ergodicity can be substantially easier to establish than mixing for important classes of stochastic systems.

The use of an ergodic framework requires a precise qualification. Ergodicity alone gives a qualitative law of large numbers for each fixed integrable observable; it does not provide the maximal inequalities, rates, or central limit theorems required for kernel derivative estimation. Stationarity and ergodicity are structural assumptions, whereas the projective/maximal, conditional-moment, conditional-density, and variance-stabilization conditions used below are separate theorem-specific quantitative hypotheses, not consequences or refinements of ergodicity. Accordingly, the results apply to stationary ergodic processes satisfying the explicit quantitative conditions of the corresponding theorem and are not asserted for arbitrary stationary ergodic sequences. If these additional assumptions fail, the displayed uniform rates, Gaussian limits, or variance/AMISE expansions need not remain valid even though a qualitative ergodic law of large numbers may still hold. The need for such quantitative conditions is not created by the missingness weight: comparable kernel-derivative rate and CLT statements would require quantitative dependence control even with complete responses. IPW changes the identification and conditional-moment structure; it does not turn ergodicity itself into a rate or central-limit condition.

This distinction is important when interpreting non-mixing examples. Certain observation-driven count processes need not be mixing without additional contraction assumptions; see, for example, the discussion surrounding the Poisson-type recursion in [78]. More generally, measurable functions of an ergodic shift remain ergodic under the standard factor construction; see [79]. Such examples demonstrate the breadth of the qualitative framework, but their inclusion in a particular quantitative theorem still requires verification of that theorem’s projective, conditional-density, moment, and variance-stabilization assumptions.

The objective of this paper is to develop a probability-theoretic route from sequential-MAR identification to asymptotic theory for kernel estimators of nonlinear regression derivatives under dependent sampling. The contributions are deliberately prioritized as follows:

1. **Identification and Estimator Architecture under Dependent MAR:** We formulate the observed-data experiment on a two-sided stationary probability space, impose sequential MAR and positivity, and identify the complete-data conditional functional through an IPW pseudo-response. The response-dependent numerator is weighted, whereas the fully observed marginal covariate density and its derivatives are estimated from the complete covariate sample, avoiding unnecessary Bernoulli variability.
2. **Quantitative Asymptotic Theory under Stationary Ergodic Dependence with Explicit Additional Conditions:** An exact martingale-predictable decomposition separates local stochastic fluctuation from dependence on the past. Under theorem-specific projective or maximal conditions, conditional moment/entropy requirements, local conditional density assumptions, and predictable-variance stabilization, we establish uniform almost-sure rates, pointwise Gaussian limits, direct variance expansions, studentization, and AMISE results. These conditions are stated as additional quantitative hypotheses and not as consequences of ergodicity.
3. **Propagation to Regression Derivatives and Feasible Procedures:** Exact quotient, reciprocal, and multi-index Leibniz identities transfer the joint primitive-estimator theory to first-, second-, higher-order, and multivariate regression derivatives while retaining numerator–denominator covariance. Propensity-score estimation is then treated as a rate-dependent extension through an explicit oracle–feasible remainder.

Higher derivative orders, bandwidth/AMISE calculations, feasible propensity replacement, variance estimation, and the Monte Carlo study are presented as extensions, consequences, or finite-sample diagnostics of this three-part core architecture rather than as co-equal claims of novelty.

The class of targets covered by the theory includes marginal density derivatives, derivatives of conditional means, derivatives of conditional moments, and covariate derivatives of conditional distribution functions. Complete-response estimators are recovered when the propensity score is identically one. The results are local to compact regions on which the covariate density and the response propensity are bounded away from zero.

The simulation study in Section 5 is deliberately aligned with this theoretical architecture. It investigates how derivative order, serial dependence, response probability, propensity-score specification, and bandwidth choice affect finite-sample bias, dispersion, integrated error, and coverage. In particular, it includes an irrational-rotation design to examine an ergodic non-mixing setting, while treating numerical agreement with the theory as supporting evidence rather than as verification of the projective, maximal, and conditional-variance assumptions.

To the best of our knowledge, the combination of kernel derivative estimation, sequential MAR correction, and stationary ergodic dependence subject to explicit non-mixing quantitative conditions has not previously been developed in this unified form. This novelty statement should be understood in the specific sense of the theorem architecture above: the contribution is not a claim that ergodicity alone suffices but, rather, a framework in which identification, smoothing regularity, martingale fluctuation, predictable dependence, and conditional variance stabilization are stated and analysed separately.

The remainder of this paper is organized as follows: Section 2 introduces the probability space, the observed-data sigma-fields, the sequential MAR mechanism, the target functionals, and the oracle and feasible kernel estimators. Section 3 states the modular assumptions and develops the bias expansions, uniform almost-sure rates, pointwise Gaussian limits, variance estimation, and AMISE theory. Section 4 applies the primitive results to first-, second-, higher-order, and multivariate regression derivatives. Section 5 reports the Monte Carlo experiments, including the dependence, missingness, propensity-estimation, bandwidth, and pointwise inference comparisons. Section 6 summarizes the conclusions and principal limitations. The complete proofs are given in Section 7.

## 2. Problem Formulation and Estimation

This section introduces the stochastic basis, the observed-data structure, the missingness mechanism, the target functionals, and the kernel derivative estimators studied throughout this paper. Particular care is taken to distinguish statistical identification from smoothness, stationarity from quantitative dependence control, and oracle inverse-probability weighting from feasible procedures in which the propensity score must itself be estimated.

The formulation, within a common measure-theoretic framework, covers marginal density derivatives, derivatives of density-weighted conditional moments, derivatives of regression functions, derivatives of conditional moments, and derivatives of conditional distribution functions. The covariate is assumed to be fully observed, whereas the response may be missing according to a missing-at-random mechanism.

### 2.1. Stochastic Basis and Observed-Data Experiment

Let

(Ω,A,P)

be a complete probability space. On this space, let

$$Z_{i}=\left(X_{i},Y_{i},\xi_{i}\right),\;i\in Z,$$

be a two-sided strictly stationary process taking values in

$$Z=R^{p}\times R^{q}\times \left\{0,1\right\},$$

where

$$X_{i}={\left(X_{i1},\ldots ,X_{ip}\right)}^{⊤}\in R^{p}$$

is a fully observed covariate vector,

$$Y_{i}={\left(Y_{i1},\ldots ,Y_{iq}\right)}^{⊤}\in R^{q}$$

is a response vector, and

$$\xi_{i}\in \left\{0,1\right\}$$

is the response-observation indicator. The event $\left\{\xi_{i}=1\right\}$ means that $Y_{i}$ is observed, whereas $\left\{\xi_{i}=0\right\}$ means that the response is missing.

The complete-data filtration is

$$G_{i}=\sigma \left(Z_{j}:j\leq i\right),\;i\in Z.$$

The statistical experiment generated by the first *n* observations is based on the observed-data sigma-field

$$O_{n}=\sigma \;\left(X_{i},\xi_{i},\xi_{i}Y_{i}:1\leq i\leq n\right).$$

Thus, every estimator used below must be $O_{n}$-measurable. The notation $\xi_{i}Y_{i}$ is interpreted component-wise; its value on $\left\{\xi_{i}=0\right\}$ is immaterial because $\xi_{i}$ is observed simultaneously.

We assume throughout that the state spaces are equipped with their Borel sigma-fields. Since Euclidean spaces are standard Borel spaces, regular conditional distributions such as

$$L\left(Y_{i}|X_{i}\right),\;L\left(Y_{i}|X_{i},G_{i-1}\right)$$

exist. Any conditional expectation indexed by a point $x\in R^{p}$ is understood as a selected Borel version of the corresponding regular conditional expectation.

Let

$$I=\prod_{j=1}^{p}\left[a_{j},b_{j}\right]\subset \mathrm{int}\left(J\right),\;J=\prod_{j=1}^{p}\left[c_{j},d_{j}\right]\subset R^{p},$$

where

$$-\infty <c_{j}<a_{j}<b_{j}<d_{j}<\infty ,\;j=1,\ldots ,p.$$

The compact set I is the evaluation region for uniform statements. The larger compact neighbourhood J is used to formulate local smoothness and positivity assumptions and to avoid evaluating Taylor expansions outside the region on which the relevant derivatives are controlled. We write

$$d_{0}=\mathrm{dist}\left(I,\partial J\right)>0.$$

### 2.2. Complete-Data Law and Target Functionals

Let $P_{X,Y}$ denote the common marginal law of $\left(X_{i},Y_{i}\right)$. We assume that, on $J\times R^{q}$, this law is absolutely continuous with respect to Lebesgue measure and has density

$$f_{X,Y}\left(x,y\right).$$

The marginal density of $X_{i}$ is therefore

$$f_{X}\left(x\right)=\int_{R^{q}}f_{X,Y}\left(x,y\right)\;dy,\;x\in J.$$

Let

$$s=\left(s_{1},\ldots ,s_{p}\right)\in N_{0}^{p},\;\left|s\right|=\sum_{j=1}^{p}s_{j},$$

and use the multi-index notation

$$D^{s}=\frac{\partial^{\left|s\right|}}{\partial x_{1}^{s_{1}}\cdots \partial x_{p}^{s_{p}}}.$$

For $\alpha \in N_{0}^{p}$, we also write

$$\alpha !=\prod_{j=1}^{p}\alpha_{j}!,\;u^{\alpha }=\prod_{j=1}^{p}u_{j}^{\alpha_{j}}.$$

Let $\psi :R^{q}\to R$ be Borel-measurable, and suppose that

$$E|\psi (Y_{0})|<\infty .$$

Define the density-weighted conditional functional

(2) $$r\left(\psi ;x\right)=E\;\left\{\psi \left(Y_{0}\right)|X_{0}=x\right\}f_{X}\left(x\right).$$

Whenever the conditional expectation in (2) is represented through the joint density, one has

(3) $$r\left(\psi ;x\right)=\int_{R^{q}}\psi \left(y\right)f_{X,Y}\left(x,y\right)\;dy.$$

The primitive object estimated in this paper is

(4) $$D^{s}r\left(\psi ;x\right),\;x\in I.$$

The representation

$$D^{s}r\left(\psi ;x\right)=\int_{R^{q}}\psi \left(y\right)D_{x}^{s}f_{X,Y}\left(x,y\right)\;dy$$

is not regarded as automatic. It is valid only under conditions permitting differentiation under the integral sign—for example, the existence of an integrable envelope for the relevant derivatives of $f_{X,Y}$. Such regularity conditions are imposed explicitly in Section 3.

Several important targets are embedded in (4).

1. For ψ≡1,
  $$r\left(1;x\right)=f_{X}\left(x\right),\;D^{s}r\left(1;x\right)=D^{s}f_{X}\left(x\right).$$
2. For q=1 and ψ(y)=y,
  $$m\left(x\right)=E\left(Y_{0}|X_{0}=x\right)=\frac{r(\psi ;x)}{f_{X}\left(x\right)}$$
  whenever $f_{X}\left(x\right)>0$.
3. For q=1 and $\psi \left(y\right)=y^{k}$,
  $$m_{k}\left(x\right)=E\left(Y_{0}^{k}|X_{0}=x\right)=\frac{r(\psi ;x)}{f_{X}\left(x\right)}.$$
4. For
  $$\psi_{t}\left(y\right)=1\left\{y\leq t\right\},\;t\in R^{q},$$
  where the inequality is component-wise,
  $$F_{Y|X}\left(t|x\right)=P\left(Y_{0}\leq t|X_{0}=x\right)=\frac{r(\psi_{t};x)}{f_{X}\left(x\right)}.$$

For Case 4, the identity follows directly from the indicator transformation:

$$E\left\{\psi_{t}\left(Y_{0}\right)|X_{0}=x\right\}=P\left(Y_{0}\leq t|X_{0}=x\right)=F_{Y|X}\left(t|x\right).$$

Combining this with $r\left(\psi ;x\right)=E\left\{\psi \left(Y_{0}\right)|X_{0}=x\right\}f_{X}\left(x\right)$ gives the displayed ratio whenever $f_{X}\left(x\right)>0$. All derivatives considered later are with respect to the covariate x, not the threshold t.

Thus, derivatives of regression-type functionals are obtained from derivatives of r(ψ;·) and $f_{X}$ through the multivariate quotient rule. The primitive numerator and denominator estimators are developed first; their nonlinear combinations are treated later.

### 2.3. Sequential Missing at Random and Positivity

For dependent observations, a purely contemporaneous condition of the form

$$P\left(\xi_{i}=1|X_{i},Y_{i}\right)=P\left(\xi_{i}=1|X_{i}\right)$$

is insufficient for the martingale decompositions used in the asymptotic analysis. Indeed, it does not determine $E(\xi_{i}|X_{i},Y_{i},G_{i-1})$; hence, it cannot guarantee that the localized past-conditioned residual has conditional mean zero. We therefore formulate missingness at random conditionally on the past. This choice includes MCAR as a constant-propensity special case and excludes MNAR mechanisms for which identification would require additional structure.

**Definition 1** 
(Sequential missing at random). *The response-observation mechanism satisfies sequential missing at random if there exists a Borel function*

π:J→(0,1]

*such that, for every i∈Z,*

(5) $$P\left(\xi_{i}=1|X_{i},Y_{i},G_{i-1}\right)=P\left(\xi_{i}=1|X_{i},G_{i-1}\right)=\pi \left(X_{i}\right)\;a.s.$$

The first equality in (5) expresses conditional ignorability of the missingness indicator with respect to the current response, given the current covariate and the complete past. The second equality excludes additional dependence of the observation probability on the past after conditioning on $X_{i}$. This second restriction is stronger than general sequential MAR, but it is precisely what identifies the target using a time-homogeneous propensity score π(x).

We also impose local positivity:

(6) $$\pi_{★}:=\underset{x\in J}{\inf}\pi \left(x\right)>0.$$

Positivity is an identification condition, not merely a technical boundedness assumption. If π(x)=0 on a set of positive $P_{X}$-measures, responses are never observed there, and the inverse weight 1/π(x) is undefined; consequently, the conditional law of the response on that set is not identified from the observed-data distribution without additional structure. Zero-propensity points outside the target neighbourhood need not invalidate a local result, but a target point with zero propensity cannot be covered by the present identification argument.

For a given transformation ψ, define the oracle inverse-probability-weighted pseudo-response

(7) $$W_{i}\left(\psi \right)=\frac{\xi_{i}\psi \left(Y_{i}\right)}{\pi (X_{i})}.$$

Although $Y_{i}$ is unobserved when $\xi_{i}=0$, the observed product is understood operationally as

$$\xi_{i}\psi \left(Y_{i}\right)=\left\{\begin{array}{cc} \psi (Y_{i}), & \xi_{i}=1,\\ 0, & \xi_{i}=0. \end{array}\right.$$

Thus, no evaluation or imputation of $\psi (Y_{i})$ is required when the response is missing; the product—and hence, $W_{i}\left(\psi \right)$—is $O_{n}$-measurable for 1≤i≤n.

**Proposition 1** 
(Conditional IPW identification). *Assume* (5), (6), *and $E|\psi (Y_{0})|<\infty$. Then,*

(8) $$E\;\left\{W_{i}\left(\psi \right)|X_{i},G_{i-1}\right\}=E\;\left\{\psi \left(Y_{i}\right)|X_{i},G_{i-1}\right\}\;a.s.$$

*Consequently,*

(9) $$E\;\left\{W_{i}\left(\psi \right)|X_{i}\right\}=E\;\left\{\psi \left(Y_{i}\right)|X_{i}\right\}\;a.s.,$$

*and*

(10) $$r\left(\psi ;x\right)=E\;\left\{W_{i}\left(\psi \right)|X_{i}=x\right\}f_{X}\left(x\right)$$

*for $P_{X}$-almost every x.*

**Proof.**  Since $\pi \left(X_{i}\right)\geq \pi_{★}>0$, $W_{i}\left(\psi \right)$ is integrable. By iterated conditional expectation,

$$\begin{array}{cc} E\;\left\{W_{i}\left(\psi \right)|X_{i},Y_{i},G_{i-1}\right\} & =\frac{\psi (Y_{i})}{\pi (X_{i})}E\;\left\{\xi_{i}|X_{i},Y_{i},G_{i-1}\right\}\\ & =\psi (Y_{i}) \end{array}$$

almost surely. Conditioning the last identity on $\sigma \left(X_{i}\right)\vee G_{i-1}$ gives (8). A further application of the tower property yields (9), and multiplication by $f_{X}\left(x\right)$ gives (10). □

The preceding proposition gives an exact identification identity under the maintained sequential-MAR restriction (5); the restriction is not an approximation used in the proof. It does not require independence between $\xi_{i}$ and $Y_{i}$ unconditionally. In an application, MAR may of course be only approximately plausible; in that case, the IPW estimator can be biased for the complete-data target. This is a model-robustness issue, not an approximation entering the displayed identification equality.

The IPW transformation changes higher conditional moments. In particular, when $E\{\psi^{2}\left(Y_{i}\right)|X_{i}=x\}<\infty$,

(11) $$E\;\left\{W_{i}^{2}\left(\psi \right)|X_{i}=x\right\}=\frac{1}{\pi (x)}E\;\left\{\psi^{2}\left(Y_{i}\right)|X_{i}=x\right\}.$$

Hence, missingness inflates the local second moment by the factor 1/π(x). This is the source of the propensity-dependent inflation in the asymptotic variance.

More generally, for every real a>0 for which the conditional absolute moment exists, the following identity is valid for non-integer *a* as well, because $\xi_{i}^{a}=\xi_{i}$ for every a>0 when $\xi_{i}\in \left\{0,1\right\}$:

(12) $$E\;\left\{|W_{i}{\left(\psi \right)|}^{a}|X_{i}=x\right\}=\pi {\left(x\right)}^{1-a}E\;\left\{|\psi \left(Y_{i}\right){|}^{a}|X_{i}=x\right\}.$$

In particular, the equality for the first absolute moment holds for arbitrary real-valued ψ:

$$E\;\left\{|W_{i}\left(\psi \right)||X_{i}=x\right\}=E\;\left\{|\psi \left(Y_{i}\right)||X_{i}=x\right\}.$$

### 2.4. Kernel, Bandwidth, and Derivative Scaling

Let

$$K:R^{p}\to R$$

be a measurable kernel satisfying

(13) $$\int_{R^{p}}K\left(u\right)\;du=1.$$

The complete assumptions on support, differentiability, moment cancellation, boundary behaviour, and integrability are stated in Section 3. At this stage, assume only that $D^{s}K$ exists in the weak or classical sense required by the subsequent theory.

Let

$$h_{n}>0,\;h_{n}⟶0.$$

We adopt the volume-bandwidth parametrization

$$b_{n}=h_{n}^{1/p},\;h_{n}=b_{n}^{p}.$$

Thus, $b_{n}$ is the usual coordinate bandwidth, and $h_{n}$ is the volume of the isotropic smoothing window up to a kernel-dependent constant. Define

(14) $$K_{h_{n}}\left(u\right)=\frac{1}{h_{n}}K\;\left(\frac{u}{h_{n}^{1/p}}\right).$$

Its derivative with respect to u is

(15) $$D^{s}K_{h_{n}}\left(u\right)=\frac{1}{h_{n}^{1+|s|/p}}D^{s}K\;\left(\frac{u}{h_{n}^{1/p}}\right).$$

The exponent 1+|s|/p is therefore dictated by the chain rule and the volume–bandwidth convention; it is not an additional modelling choice.

For later use, write

$$K_{n,x}\left(u\right)=K\;\left(\frac{x-u}{h_{n}^{1/p}}\right),\;K_{n,x}^{\left(s\right)}\left(u\right)=D^{s}K\;\left(\frac{x-u}{h_{n}^{1/p}}\right).$$

### 2.5. Primitive Oracle Estimators

Because the covariates are always observed, the marginal density and its derivatives should be estimated from the complete covariate sample, without inverse probability weighting.

**Definition 2** 
(Marginal density-derivative estimator). *For x∈I, define*

(16) $$D^{s}f_{X;n}\left(x,h_{n}\right)=\frac{1}{nh_{n}^{1+|s|/p}}\sum_{i=1}^{n}D^{s}K\;\left(\frac{x-X_{i}}{h_{n}^{1/p}}\right).$$

No missingness indicator appears in (16). Introducing a factor $\xi_{i}/\pi \left(X_{i}\right)$ would preserve the expectation under MAR, but it would replace an observed quantity with a noisier unbiased surrogate and would therefore generally increase variance without improving identification.

The density-weighted response functional does involve the possibly missing response and, consequently, requires IPW correction.

**Definition 3** 
(Oracle IPW derivative estimator). *Assume that the propensity score π is known. For x∈I, define*

(17) $$D^{s}r_{n}^{\;\mathrm{IPW}}\left(\psi ;x,h_{n}\right)=\frac{1}{nh_{n}^{1+|s|/p}}\sum_{i=1}^{n}W_{i}\left(\psi \right)D^{s}K\;\left(\frac{x-X_{i}}{h_{n}^{1/p}}\right),$$

*where $W_{i}\left(\psi \right)$ is given by (7).*

The term “oracle” means only that π is treated as known. It does not mean that the estimator has access to unobserved responses: because $\xi_{i}\psi \left(Y_{i}\right)=0$ when $\xi_{i}=0$, every summand in (17) is observed.

For s=0, the primitive estimators become

(18) $$r_{n}^{\;\mathrm{IPW}}\left(\psi ;x,h_{n}\right)=\frac{1}{nh_{n}}\sum_{i=1}^{n}W_{i}\left(\psi \right)K\;\left(\frac{x-X_{i}}{h_{n}^{1/p}}\right)$$

and

(19) $$f_{X;n}\left(x,h_{n}\right)=\frac{1}{nh_{n}}\sum_{i=1}^{n}K\;\left(\frac{x-X_{i}}{h_{n}^{1/p}}\right).$$

**Remark 1** 
(Sign convention). *The derivative in (16) is taken with respect to the evaluation point x. Because the kernel argument is $\left(x-X_{i}\right)/h_{n}^{1/p}$, no factor ${\left(-1\right)}^{\left|s\right|}$ appears in the estimator. Such a factor would arise if one differentiated a kernel written as $K(\left(X_{i}-x\right)/h_{n}^{1/p})$ instead.*

**Remark 2** 
(Expectation as convolution derivative). *Under Fubini’s theorem and the regularity required to differentiate under the integral sign,*

$$E\;\left[D^{s}f_{X;n}\left(x,h_{n}\right)\right]=D^{s}\left\{K_{h_{n}}*f_{X}\right\}\left(x\right),$$

*and Proposition 1 gives*

$$E\;\left[D^{s}r_{n}^{\;\mathrm{IPW}}\left(\psi ;x,h_{n}\right)\right]=D^{s}\left\{K_{h_{n}}*r\left(\psi ;\cdot \right)\right\}\left(x\right).$$

*These identities explain why the deterministic biases of the density and IPW numerator estimators have the same kernel-convolution form. Missingness affects the stochastic variance but not the oracle smoothing bias.*

### 2.6. Regression-Type Ratio Estimator

Whenever $f_{X}\left(x\right)>0$, define

$$m\left(\psi ;x\right)=E\left\{\psi \left(Y_{0}\right)|X_{0}=x\right\}=\frac{r(\psi ;x)}{f_{X}\left(x\right)}.$$

The natural oracle IPW Nadaraya–Watson estimator is

(20) $${\hat{m}}_{n}^{\;\mathrm{IPW}}\left(\psi ;x,h_{n}\right)=\frac{r_{n}^{\;\mathrm{IPW}}\left(\psi ;x,h_{n}\right)}{f_{X;n}\left(x,h_{n}\right)}=\frac{\sum_{i=1}^{n}\frac{\xi_{i}\psi \left(Y_{i}\right)}{\pi (X_{i})}K\;\left(\frac{x-X_{i}}{h_{n}^{1/p}}\right)}{\sum_{i=1}^{n}K\;\left(\frac{x-X_{i}}{h_{n}^{1/p}}\right)}$$

whenever the denominator is nonzero.

The denominator in (20) uses all observed covariates. This is not merely a computational preference. The denominator estimates $f_{X}\left(x\right)$, which is a functional of a fully observed variable. Replacing it with

$$\sum_{i=1}^{n}\frac{\xi_{i}}{\pi (X_{i})}K\;\left(\frac{x-X_{i}}{h_{n}^{1/p}}\right)$$

would produce another unbiased estimating device for the same local mass but would add Bernoulli noise and would change the joint covariance structure of the ratio estimator.

To avoid undefined ratios, one may use the trimmed version

(21) $${\hat{m}}_{n,\tau_{n}}^{\;\mathrm{IPW}}\left(\psi ;x,h_{n}\right)=\frac{r_{n}^{\;\mathrm{IPW}}\left(\psi ;x,h_{n}\right)}{f_{X;n}\left(x,h_{n}\right)\vee \tau_{n}},$$

where $\tau_{n}↓0$. If $f_{X}$ is bounded away from zero on I and the density estimator converges uniformly, trimming is asymptotically inactive.

For derivatives of m(ψ;·), directly differentiating the random ratio (20) is algebraically possible but obscures the primitive stochastic structure. We instead estimate the required derivatives of r(ψ;·) and $f_{X}$ separately and substitute them into the multivariate quotient identities. This construction is developed in Section 4.

### 2.7. Feasible Inverse Probability Weighting

The oracle estimator is the primary theoretical benchmark: it isolates the kernel-derivative and dependence mechanisms without nuisance-estimation error. The feasible construction below is a rate-dependent extension, rather than a co-equal baseline, because replacing π with ${\hat{\pi }}_{n}$ generates an additional remainder that must be negligible at the scale of the theorem being used.

In applications, π is usually unknown. Let

$${\hat{\pi }}_{n}:J\to \left[0,1\right]$$

be an $O_{n}$-measurable estimator. To prevent unstable inverse weights, define a deterministic truncation level

$${\underline{\pi }}_{n}↓0$$

and the stabilized propensity estimator

(22) $${\hat{\pi }}_{n}^{†}\left(x\right)={\hat{\pi }}_{n}\left(x\right)\vee {\underline{\pi }}_{n}.$$

The feasible pseudo-response is

$${\hat{W}}_{i,n}\left(\psi \right)=\frac{\xi_{i}\psi \left(Y_{i}\right)}{{\hat{\pi }}_{n}^{†}\left(X_{i}\right)}.$$

Here and throughout, the adjective *feasible* is used in its standard statistical sense: the estimator can be implemented from the observed sample after replacing the unknown propensity score π with an estimator ${\hat{\pi }}_{n}$. It is therefore contrasted with the *oracle* estimator, which treats the true propensity score as known. Feasibility refers to implementability and does not, by itself, assert oracle-equivalent asymptotic behaviour.

**Definition 4** 
(Feasible IPW derivative estimator). *For x∈I, define*

(23) $$D^{s}{\hat{r}}_{n}^{\;\mathrm{IPW}}\left(\psi ;x,h_{n}\right)=\frac{1}{nh_{n}^{1+|s|/p}}\sum_{i=1}^{n}{\hat{W}}_{i,n}\left(\psi \right)D^{s}K\;\left(\frac{x-X_{i}}{h_{n}^{1/p}}\right).$$

The difference between the feasible and oracle estimators is exactly

(24) $$\begin{array}{cc} \; & D^{s}{\hat{r}}_{n}^{\;\mathrm{IPW}}\left(\psi ;x,h_{n}\right)-D^{s}r_{n}^{\;\mathrm{IPW}}\left(\psi ;x,h_{n}\right)\\ \; & \;=\frac{1}{nh_{n}^{1+|s|/p}}\sum_{i=1}^{n}\xi_{i}\psi \left(Y_{i}\right)\left\{\frac{1}{{\hat{\pi }}_{n}^{†}\left(X_{i}\right)}-\frac{1}{\pi (X_{i})}\right\}D^{s}K\;\left(\frac{x-X_{i}}{h_{n}^{1/p}}\right). \end{array}$$

Equation (24) shows why mere uniform consistency

$$∥{\hat{\pi }}_{n}{-\pi ∥}_{\infty ,J}=o_{P}\left(1\right)$$

does not automatically imply oracle-equivalent central limit theory. The required rate depends on the stochastic normalization, the smoothness of the weight map u↦1/u, the empirical-process behaviour of the weighted kernel class, and whether orthogonalization or sample splitting is used.

The word *feasible* explains only that the procedure is computable with an estimated propensity; it does not make oracle equivalence automatic. Writing $\delta_{n}={∥{\hat{\pi }}_{n}-\pi ∥}_{\infty ,J}$, the direct plug-in comparison below shows that a sufficient condition for the propensity-estimation remainder to vanish under the pointwise CLT normalization is

$$\sqrt{nh_{n}}\;\delta_{n}⟶0\;\mathrm{in}\;\mathrm{probability};$$

see (78). This condition is used together with positivity/stabilization and the moment and stochastic-process assumptions required by the oracle CLT. If one has only $\delta_{n}=o_{P}\left(1\right)$, the additional remainder can still be of first order at the Gaussian scale. Orthogonal or cross-fitted constructions can potentially relax this rate requirement, but they require a separate estimating equation and corresponding theory.

For a direct plug-in estimator without orthogonalization, a simple sufficient bound derived from (24) is generally too strong to be attractive. A sharp feasible theory should therefore be stated as a separate result and should specify at least one of the following:

1. A parametric or sufficiently fast semiparametric propensity estimator;
2. Cross-fitting together with a Neyman-orthogonal estimating equation;
3. An explicit stochastic expansion for ${\hat{\pi }}_{n}-\pi$ under dependence;
4. Entropy and moment conditions controlling the random function class induced by ${\hat{\pi }}_{n}$.

Accordingly, the principal asymptotic results of this paper are first proven for the oracle estimator (17). Any feasible extension must verify that the remainder in (24) is negligible at the scale of the particular theorem under consideration.

The corresponding feasible Nadaraya–Watson estimator is

(25) $${\hat{m}}_{n}^{\;\mathrm{FIPW}}\left(\psi ;x,h_{n}\right)=\frac{\sum_{i=1}^{n}\frac{\xi_{i}\psi \left(Y_{i}\right)}{{\hat{\pi }}_{n}^{†}\left(X_{i}\right)}K\;\left(\frac{x-X_{i}}{h_{n}^{1/p}}\right)}{\sum_{i=1}^{n}K\;\left(\frac{x-X_{i}}{h_{n}^{1/p}}\right)}.$$

As in the oracle estimator, the denominator remains unweighted.

### 2.8. Important Special Cases

**Remark 3** 
(Complete responses). *If $\xi_{i}=1$ almost surely for all i, then π≡1 and*

$$W_{i}\left(\psi \right)=\psi \left(Y_{i}\right).$$

*The oracle IPW estimator reduces to the ordinary complete-data kernel derivative estimator. Thus, the missing-response theory contains the complete-response theory as a special case.*

**Remark 4** 
(Marginal density derivatives). *Although the population identity*

$$r\left(1;x\right)=f_{X}\left(x\right)$$

*holds, the statistically preferred estimator of $D^{s}f_{X}$ under response missingness is* (16), *not* (17) *with ψ≡1. Indeed, the latter would equal*

$$\frac{1}{nh_{n}^{1+|s|/p}}\sum_{i=1}^{n}\frac{\xi_{i}}{\pi (X_{i})}D^{s}K\;\left(\frac{x-X_{i}}{h_{n}^{1/p}}\right),$$

*which discards the variance advantage of observing every $X_{i}$.*

**Remark 5** 
(Conditional moments). *For a scalar response and*

$$\psi_{k}\left(y\right)=y^{k},$$

*the ratio estimator estimates*

$$m_{k}\left(x\right)=E\left(Y_{0}^{k}|X_{0}=x\right).$$

*The moment assumptions required later must be adjusted to k. For example, a second-moment calculation for the IPW estimator of $m_{k}$ requires a conditional moment of order 2k for $Y_{0}$, together with positivity.*

**Remark 6** 
(Conditional distribution functions). *For*

$$\psi_{t}\left(y\right)=1\left\{y\leq t\right\},$$

*the pseudo-response is bounded by $1/\pi_{★}$, which simplifies the conditional Bernstein and Lindeberg conditions. Uniformity with respect to t, however, introduces an additional empirical-process index and requires entropy control for the class of lower orthants:*

$$\left\{\left(-\infty ,t\right]:t\in R^{q}\right\}.$$

*Pointwise results in t do not require this additional uniformity.*

**Remark 7** 
(Signed and higher-order kernels). *If the kernel order exceeds two, the moment conditions*

$$\int u^{\alpha }K\left(u\right)\;du=0,\;1\leq \left|\alpha \right|\leq \ell -1,$$

*generally force K to take both positive and negative values. Such a kernel should not be described as a probability density. Non-negativity is compatible with the standard second-order case but not with arbitrary higher-order cancellation.*

**Remark 8** 
(Anisotropic bandwidths). *The isotropic volume bandwidth is used to keep the notation transparent. For coordinate bandwidths*

$$b_{n}=\left(b_{n,1},\ldots ,b_{n,p}\right),\;b_{n,j}>0,$$

*one replaces*

$$h_{n}\;\mathrm{by}\;\left|b_{n}\right|=\prod_{j=1}^{p}b_{n,j}$$

*and uses*

$$K_{b_{n}}\left(x-X_{i}\right)=\frac{1}{|b_{n}|}K\;\left(\frac{x_{1}-X_{i1}}{b_{n,1}},\ldots ,\frac{x_{p}-X_{ip}}{b_{n,p}}\right).$$

*The derivative normalization becomes*

$$\frac{1}{|b_{n}|\prod_{j=1}^{p}b_{n,j}^{s_{j}}}.$$

*The resulting theory requires coordinate-wise bias expansions and balance conditions preventing one bandwidth component from vanishing too rapidly relative to the others.*

**Remark 9** 
(Identification versus asymptotics). *Sequential MAR and positivity identify the target. They do not, by themselves, imply consistency rates, maximal inequalities, or central limit theorems. Strict stationarity and ergodicity yield qualitative long-run averaging for integrable fixed functions, but quantitative uniform rates and asymptotic normality require additional assumptions. The projective, conditional-moment, smoothness, stabilization, and bandwidth conditions used for those conclusions are stated separately in Section 3.*

*Guide to the Main Notation*

SymbolMeaning$X_{i}\in R^{p}$, $Y_{i}\in R^{q}$Fully observed covariate and possibly missing response.

$\xi_{i}$

Response-observation indicator; $\xi_{i}=1$ when $Y_{i}$ is observed.$G_{i}$, $O_{n}$Complete-past filtration and observed-data sigma-field.

π(x)

Sequential-MAR response propensity.

$W_{i}\left(\psi \right)$

Oracle IPW pseudo-response $\xi_{i}\psi \left(Y_{i}\right)/\pi \left(X_{i}\right)$.$f_{X}$, r(ψ;x)Covariate density and density-weighted conditional functional.s, |s|, $D^{s}$Derivative multi-index, total derivative order, and corresponding partial derivative.$h_{n}$, $b_{n}=h_{n}^{1/p}$Volume bandwidth and isotropic coordinate bandwidth.*K*, $D^{s}K$Kernel and kernel derivative.

$D^{s}r_{n}^{\mathrm{IPW}}$

Oracle primitive IPW kernel derivative estimator.

$D^{s}{\hat{r}}_{n}^{\mathrm{IPW}}$

Feasible primitive estimator using a stabilized ${\hat{\pi }}_{n}$.$M_{n,s}$, $P_{n,s}$Martingale and predictable components of the centred kernel process.

### 2.9. Summary of the Estimation Architecture

The subsequent theory is organized around the following hierarchy:

1. The observed-data law identifies r(ψ;x) through the sequential-MAR identity (10).
2. The derivative $D^{s}r\left(\psi ;x\right)$ is estimated by the oracle primitive estimator (17).
3. The marginal density derivatives are estimated from all covariates by (16).
4. Regression functions, conditional moments, and conditional distribution functions are formed as ratios of the primitive numerator and density estimators.
5. Derivatives of these ratios are obtained from the collection of primitive derivative estimators through exact multivariate quotient identities.
6. A feasible propensity estimator introduces the explicit additional remainder (24); oracle-equivalent inference is valid only after that remainder has been controlled at the relevant stochastic scale.

This separation between identification, primitive estimation, nonlinear functional construction, and propensity estimation is essential. It prevents the missingness correction from being introduced where it is unnecessary, makes the source of variance inflation transparent, and allows the dependence conditions required for each asymptotic result to be stated and verified without ambiguity.

## 3. Assumptions and Asymptotic Properties

This section gives the precise analytical and probabilistic conditions under which the estimators introduced in Section 2 possess uniform strong-consistency rates, pointwise Gaussian limits, consistent studentization, and asymptotic mean integrated squared error expansions.

A fundamental distinction must be maintained throughout. Strict stationarity and ergodicity are qualitative structural properties. They provide an appropriate invariant probabilistic framework and, for each fixed integrable observable, a pointwise ergodic theorem. They do not by themselves imply a convergence rate, an exponential inequality, a functional maximal inequality, or a central limit theorem. The latter conclusions require additional quantitative hypotheses. We therefore organize the assumptions into separate modules and invoke only the modules required by each theorem.

The assumptions should also be distinguished from empirical diagnostics. Positivity can be investigated through the distribution and low quantiles of an estimated propensity, effective-weight diagnostics, and sensitivity to trimming or overlap restrictions. Moment assumptions can be probed through tail plots and sensitivity to truncation or robustification. Serial dependence can be explored through autocorrelation, block-variance comparisons, and stability across subsamples or block lengths. Such diagnostics can reveal practical instability or clear violations but cannot certify theorem-level projective or conditional-variance assumptions from a single finite series. Likewise, there is no universal sample size at which the asymptotic approximation becomes accurate: the relevant scale depends jointly on dimension, derivative order, bandwidth, local design density, propensity, tail behaviour, and dependence. Section 5 therefore examines finite-sample behaviour over several sample sizes and dependence strengths as a diagnostic complement to—not a substitute for—the asymptotic theory.

For practical use, several concrete warning signs should trigger caution even though no universal numerical cutoff can certify failure. These include estimated propensities concentrated close to zero or repeatedly hitting the stabilization threshold, a few inverse-probability weights dominating the total weight, a large coefficient of variation of the weights, or a sharply reduced weight-based effective sample size:

$$N_{\mathrm{eff}}=\frac{{\left(\sum_{i=1}^{n}w_{i}\right)}^{2}}{\sum_{i=1}^{n}w_{i}^{2}},\;w_{i}=\frac{\xi_{i}}{{\hat{\pi }}_{n}^{†}\left(X_{i}\right)}.$$

Further warning signs include substantial changes in the estimate under modest trimming or bandwidth perturbations, very low estimated design density near the evaluation point, slowly decaying sample autocorrelations, pronounced block-variance inflation, or instability across block lengths and subsamples. When several of these features occur together, the effective local information can be much smaller than the nominal sample size, and the first-order approximation should be regarded as potentially unreliable.

Throughout, let $Z_{i}=\left(X_{i},Y_{i},\xi_{i}\right),\;i\in Z,$ be the two-sided process of Section 2, and let $G_{i}=\sigma \left(Z_{j}:j\leq i\right).$ For a Borel transformation $\psi :R^{q}\to R$, define

$$W_{i}\left(\psi \right)=\frac{\xi_{i}\psi \left(Y_{i}\right)}{\pi (X_{i})}.$$

For a fixed multi-index $s\in N_{0}^{p}$, write

$$\kappa_{s}=D^{s}K,\;b_{n}=h_{n}^{1/p},$$

so that

$$D^{s}r_{n}^{\;\mathrm{IPW}}\left(\psi ;x,h_{n}\right)=\frac{1}{nh_{n}^{1+|s|/p}}\sum_{i=1}^{n}W_{i}\left(\psi \right)\kappa_{s}\left(\frac{x-X_{i}}{b_{n}}\right).$$

For a>0, define the conditional IPW absolute-moment function,

$$\Psi_{a}^{\mathrm{IPW}}\left(x,\psi \right)=E\;\left[|W_{0}{\left(\psi \right)|}^{a}|X_{0}=x\right],$$

whenever finite. Under sequential MAR and positivity,

(26) $$\Psi_{a}^{\mathrm{IPW}}\left(x,\psi \right)=\pi {\left(x\right)}^{1-a}E\;\left[|\psi \left(Y_{0}\right){|}^{a}|X_{0}=x\right].$$

In particular,

(27) $$\Psi_{2}^{\mathrm{IPW}}\left(x,\psi \right)=\frac{E\{\psi^{2}\left(Y_{0}\right)|X_{0}=x\}}{\pi (x)}.$$

The local second-moment density is

(28) $$q_{\psi }\left(x\right)=f_{X}\left(x\right)\Psi_{2}^{\mathrm{IPW}}\left(x,\psi \right).$$

### 3.1. The Canonical Martingale-Predictable Decomposition

The asymptotic theory is most transparent when the estimator is decomposed relative to the past filtration. Define

$$\Theta_{i,n}\left(x\right)=W_{i}\left(\psi \right)\kappa_{s}\left(\frac{x-X_{i}}{b_{n}}\right)$$

and

$$A_{i,n}\left(x\right)=E\left\{\Theta_{i,n}\left(x\right)|G_{i-1}\right\}.$$

Then,

$$\Delta_{i,n}\left(x\right)=\Theta_{i,n}\left(x\right)-A_{i,n}\left(x\right)$$

is a martingale difference:

$$E\{\Delta_{i,n}\left(x\right)|G_{i-1}\}=0.$$

Consequently,

(29) $$\begin{array}{cc} \; & D^{s}r_{n}^{\;\mathrm{IPW}}\left(\psi ;x,h_{n}\right)-ED^{s}r_{n}^{\;\mathrm{IPW}}\left(\psi ;x,h_{n}\right)\\ \; & \;=M_{n,s}\left(x\right)+P_{n,s}\left(x\right), \end{array}$$

where

(30) $$M_{n,s}\left(x\right)=\frac{1}{nh_{n}^{1+|s|/p}}\sum_{i=1}^{n}\Delta_{i,n}\left(x\right)$$

and

(31) $$P_{n,s}\left(x\right)=\frac{1}{nh_{n}^{1+|s|/p}}\sum_{i=1}^{n}\left[A_{i,n}\left(x\right)-EA_{i,n}\left(x\right)\right].$$

The first term in (29) is controlled by martingale exponential inequalities or a martingale triangular-array central limit theorem. The second term carries the residual temporal dependence of the conditional law given the past. It cannot be discarded merely because the process is ergodic.

To express the predictable term more explicitly, let

$$f_{i}\left(u\right)=f_{X_{i}|G_{i-1}}\left(u\right)$$

be a selected version of the conditional density of $X_{i}$ given $G_{i-1}$, and define

(32) $$g_{i,\psi }\left(u\right)=E\;\left[W_{i}\left(\psi \right)|X_{i}=u,G_{i-1}\right]f_{i}\left(u\right).$$

Under sequential MAR,

$$g_{i,\psi }\left(u\right)=E\;\left[\psi \left(Y_{i}\right)|X_{i}=u,G_{i-1}\right]f_{i}\left(u\right).$$

Whenever the required weak derivatives and integration-by-parts identities hold,

(33) $$\frac{A_{i,n}\left(x\right)}{h_{n}^{1+|s|/p}}=\int_{R^{p}}K\left(v\right)D^{s}g_{i,\psi }\left(x-b_{n}v\right)\;dv.$$

Thus, the predictable part is a smoothed average of the centred random functions

(34) $$G_{i,\psi }^{\left(s\right)}\left(u\right)=D^{s}g_{i,\psi }\left(u\right)-D^{s}r\left(\psi ;u\right).$$

Identity (33) is central: it separates the singular derivative-kernel normalization from the temporal dependence and shows that the predictable contribution is governed by partial sums of ordinary smooth random functions.

### 3.2. Assumption Modules

We now state the assumptions in modules. Some are standing assumptions, while others are invoked only for particular conclusions.

Structural and identification assumptions

(S.1) *Two-Sided Stationarity and Ergodicity:* The process
  $$\left\{Z_{i}:i\in Z\right\}$$
  is strictly stationary and ergodic on (Ω,A,P), and $Z_{i}$ is $G_{i}$-measurable.
(S.2) *Sequential MAR and Positivity:* For every i∈Z,
  $$P\left(\xi_{i}=1|X_{i},Y_{i},G_{i-1}\right)=P\left(\xi_{i}=1|X_{i},G_{i-1}\right)=\pi \left(X_{i}\right)\;a.s.,$$
  where π is continuous on J and
  $$0<\pi_{★}\leq \underset{x\in J}{\inf}\pi \left(x\right)\leq 1.$$
(S.3) *Interior Evaluation Region:* The compact sets satisfy
  $$I\subset \mathrm{int}\left(J\right),\;d_{0}=\mathrm{dist}\left(I,\partial J\right)>0.$$
  All uniform conclusions are stated on I; the smoothness and conditional-density assumptions are imposed on an open neighbourhood of J.

Kernel and deterministic smoothness assumptions

(K.1) *Kernel Regularity:* The kernel $K:R^{p}\to R$ is bounded, integrable, compactly supported, and satisfies
  $$\int_{R^{p}}K\left(u\right)\;du=1.$$
  For every derivative multi-index used in the paper, $D^{s}K$ exists as a weak derivative and belongs to
  $$L^{1}\left(R^{p}\right)\cap L^{2}\left(R^{p}\right)\cap L^{\infty }\left(R^{p}\right).$$
  For the uniform theorem, $D^{s}K$ is Lipschitz:
  $$|D^{s}K\left(u\right)-D^{s}K\left(v\right)|\leq L_{s}∥u-v∥.$$
  Compact support and weak differentiability are assumed to be sufficiently strong to justify
  $$\int a\left(x-bu\right)D^{s}K\left(u\right)\;du=b^{\left|s\right|}\int D^{s}a\left(x-bu\right)K\left(u\right)\;du$$
  for the functions *a* appearing below.
(K.2) *Kernel Order:* For an integer ℓ≥1,
  $$\int_{R^{p}}u^{\alpha }K\left(u\right)\;du=0,\;1\leq \left|\alpha \right|\leq \ell -1,$$
  and
  $$\int_{R^{p}}{∥u∥}^{\ell }\left|K\left(u\right)\right|\;du<\infty .$$
  We write
  $$\mu_{\alpha }\left(K\right)=\int_{R^{p}}u^{\alpha }K\left(u\right)\;du$$
  and
  (35) $$R_{s}\left(K\right)=\int_{R^{p}}{\left\{D^{s}K\left(u\right)\right\}}^{2}\;du.$$
  When ℓ>2, *K* is allowed to be signed.
(R.1) *Smoothness of the Target:* The function r(ψ;·) extends to an open neighbourhood of J, belongs to $C^{|s|+\ell }$ there, and has bounded derivatives through order |s|+ℓ. Its derivatives of total order |s|+ℓ are uniformly continuous on J.
  The same assumption, with r(ψ;·) replaced by $f_{X}$, is imposed when a marginal density derivative is studied. Whenever a regression quotient is considered,
  $$\underset{x\in J}{\inf}f_{X}\left(x\right)>0.$$
(R.2) *Differentiation under the Response Integral:* For every multi-index β with |β|≤|s|+ℓ, the derivative
  $$D_{x}^{\beta }f_{X,Y}\left(x,y\right)$$
  exists for almost every y, and there is an integrable envelope $H_{\beta ,\psi }$ such that
  $$\underset{x\in J}{\sup}\left|\psi \left(y\right)\right|\left|D_{x}^{\beta }f_{X,Y}\left(x,y\right)\right|\leq H_{\beta ,\psi }\left(y\right),$$
  with
  $$\int_{R^{q}}H_{\beta ,\psi }\left(y\right)\;dy<\infty .$$
  This assumption guarantees
  $$D^{\beta }r\left(\psi ;x\right)=\int_{R^{q}}\psi \left(y\right)D_{x}^{\beta }f_{X,Y}\left(x,y\right)\;dy.$$

Predictable-component assumptions

Let $G_{i,\psi }^{\left(s\right)}$ be defined by (34). We distinguish an $L^{2}$ condition used for pointwise Gaussian limits from a stronger maximal condition used for almost-sure uniform rates.

(P.1) *Conditional First-Moment Density Regularity:* For every *i*, the conditional density $f_{i}$ and the function $g_{i,\psi }$ in (32) possess versions whose derivatives through order |s| are continuous on J. Moreover,
  $$\underset{i\in Z}{\sup}{∥\underset{u\in J}{\sup}\left|D^{s}g_{i,\psi }\left(u\right)\right|∥}_{2}<\infty ,$$
  and
  $$ED^{s}g_{i,\psi }\left(u\right)=D^{s}r\left(\psi ;u\right)$$
  for every u∈J.
(P.2) *Quadratic Predictable Stabilization:* There exists a finite constant $C_{P}$ such that
  (36) $$\underset{n\geq 1}{\sup}\frac{1}{n}E\left[\underset{u\in J}{\sup}{\left|\sum_{i=1}^{n}G_{i,\psi }^{\left(s\right)}\left(u\right)\right|}^{2}\right]\leq C_{P}.$$
  For a merely pointwise theorem at x, it is sufficient to impose (36) on a fixed neighbourhood of x, or after integration against the translates of *K*.
(P.3) *Almost-Sure Predictable Maximal Bound:* For the strong uniform theorem,
  (37) $$\underset{u\in J}{\sup}\left|\sum_{i=1}^{n}G_{i,\psi }^{\left(s\right)}\left(u\right)\right|=O_{a.s.}\left\{{\left(n\log n\right)}^{1/2}\right\}.$$
  A sharper iterated-logarithm bound may replace (37). The displayed assumption is stated explicitly because neither ergodicity nor an $L^{2}$ partial-sum estimate alone implies an almost-sure maximal rate.

A useful sufficient route to (P.2) is available through Hilbert-valued projective criteria. Let m>p/2, and suppose that $G_{i,\psi }^{\left(s\right)}$ is a stationary random element of the Sobolev space $H^{m}\left(J\right)$. By the Sobolev embedding theorem,

$$H^{m}\left(J\right)↪C\left(J\right).$$

If

(38) $$\sum_{k\in Z}{∥P_{0}G_{k,\psi }^{\left(s\right)}∥}_{L^{2}\left(\Omega ;H^{m}\left(J\right)\right)}<\infty ,$$

then the associated Hannan-type martingale approximation yields a linear $L^{2}$ partial-sum bound in $H^{m}\left(J\right)$, and hence, (P.2) by continuous embedding. Condition (38) is absolute projective summability; square summability of projection norms is not an interchangeable substitute.

Condition (P.3) requires a corresponding almost-sure maximal theorem. It may be verified, for example, through a strong invariance principle, a martingale–coboundary decomposition with suitable $L^{2+\delta }$ control, or a directly established functional maximal inequality. We do not infer it automatically from (38).

Martingale moment and entropy assumptions for uniform rates

(U.1) *Uniform Conditional-Density Bound:* There exists $C_{f}<\infty$ such that
  $$\underset{i\in Z}{\sup}\underset{u\in J}{\sup}f_{i}\left(u\right)\leq C_{f}\;a.s.$$
(U.2) *Conditional Bernstein Condition:* There exist constants $v_{\psi }>0$ and $c_{\psi }>0$ such that, for every integer ν≥2,
  (39) $$\underset{i\in Z}{\sup}\underset{u\in J}{\sup}E\;\left[|W_{i}{\left(\psi \right)|}^{\nu }|X_{i}=u,G_{i-1}\right]\leq \frac{\nu !}{2}v_{\psi }^{2}c_{\psi }^{\nu -2}\;a.s.$$
  A bounded pseudo-response is a sufficient special case. Assumption (39) may be replaced by an explicit truncation scheme, but a finite (2+η)-moment by itself does not justify a Bernstein exponential inequality for the untruncated process.
(U.3) *Translation-Class Entropy:* For
  $$K_{n,s}=\left\{u\mapsto \kappa_{s}\left(\frac{x-u}{b_{n}}\right):x\in I\right\},$$
  the covering numbers satisfy, for constants A,V<∞,
  $$N\;\left(\varepsilon ∥\kappa_{s}{∥}_{\infty },K_{n,s},{∥\cdot ∥}_{\infty }\right)\leq A{\left(\frac{A}{\varepsilon b_{n}}\right)}^{V},\;0<\varepsilon \leq 1.$$
  For a compactly supported Lipschitz kernel indexed by translations in $R^{p}$, this polynomial entropy bound holds with a finite exponent depending only on *p*.

Conditional variance and Lindeberg assumptions

Define the predictable local second-moment density

(40) $$q_{i,\psi }\left(u\right)=E\;\left[W_{i}^{2}\left(\psi \right)|X_{i}=u,G_{i-1}\right]f_{i}\left(u\right).$$

(C.1) *Local Conditional Variance Stabilization:* For each fixed x∈I, there exists a neighbourhood $N_{x}$ whose closure is contained in J such that
  (41) $$\underset{u\in N_{x}}{\sup}\left|\frac{1}{n}\sum_{i=1}^{n}q_{i,\psi }\left(u\right)-q_{\psi }\left(u\right)\right|\overset{P}{\to }0.$$
  The functions $q_{i,\psi }$ are locally equicontinuous in probability on $N_{x}$, and $q_{\psi }$ is continuous at x.
(C.2) *Conditional Lindeberg Moment:* There exists η>0 and a finite random variable $C_{2+\eta }$, bounded in probability, such that
  (42) $$\underset{i}{\sup}\underset{u\in N_{x}}{\sup}E\;\left[|W_{i}{\left(\psi \right)|}^{2+\eta }|X_{i}=u,G_{i-1}\right]\leq C_{2+\eta }\;a.s.$$
  Together with $nh_{n}\to \infty$, this implies the conditional Lindeberg condition for the normalized martingale array.
(C.3) *Nondegeneracy:* At the evaluation point,
  $$0<q_{\psi }\left(x\right)<\infty$$
  and
  $$0<R_{s}\left(K\right)<\infty .$$

Continuity of the unconditional function $\Psi_{2}^{\mathrm{IPW}}$ is not a replacement for (41). The martingale central limit theorem requires convergence of the sum of conditional variances, which is a past-conditioned object.

Bandwidth modules

(H.1) *Basic Localization:*
$$h_{n}↓0,\;nh_{n}^{1+2|s|/p}⟶\infty .$$
(H.2) *Uniform-Rate Bandwidth:*
(43) $$\frac{nh_{n}^{1+2|s|/p}}{\log n}⟶\infty .$$
  This condition implies that $nh_{n}\to \infty$ and $\log\left(1/h_{n}\right)=O\left(\log n\right)$.
(H.3) *Undersmoothing for Target-Centred Inference:*
(44) $$\sqrt{nh_{n}^{1+2|s|/p}}\;h_{n}^{\ell /p}⟶0.$$
(H.4) *Finite Nonzero Asymptotic Bias:* For a biased Gaussian limit, one may replace (44) with
  (45) $$\sqrt{nh_{n}^{1+2|s|/p}}\;h_{n}^{\ell /p}⟶\lambda \in \left[0,\infty \right).$$

Integrated-risk assumptions

(I.1) *Uniform Variance Stabilization on I:* Condition (41) holds uniformly on I, $q_{\psi }$ is bounded and continuous on I, and the variance remainders are uniformly integrable after multiplication by $nh_{n}^{1+2|s|/p}$.
(I.2) *Negligible Integrated Predictable Variance:*
  $$nh_{n}^{1+2|s|/p}\int_{I}E\left[P_{n,s}^{2}\left(x\right)\right]\;dx⟶0,$$
  and the integrated covariance between $M_{n,s}$ and $P_{n,s}$ is
  $$o\;\left(\frac{1}{nh_{n}^{1+2|s|/p}}\right).$$
  Since the martingale and predictable terms are orthogonal only under special additional structure, this covariance condition is stated rather than silently assumed.
(I.3) *Uniform $L^{2}$ Bias Remainder:* The remainder in the bias expansion below is $o(h_{n}^{\ell /p})$ in $L^{2}\left(I\right)$.

### 3.3. Deterministic Bias Expansion

Define

(46) $$B_{s,\ell }\left(x\right)={\left(-1\right)}^{\ell }\sum_{|\alpha |=\ell }\frac{\mu_{\alpha }\left(K\right)}{\alpha !}D^{s+\alpha }r\left(\psi ;x\right).$$

**Proposition 2** 
(Uniform bias expansion). *Assume* (S.3), (K.1)–(K.2)*, and* (R.1)–(R.2)*. Then,*

(47) $$\underset{x\in I}{\sup}\left|ED^{s}r_{n}^{\;\mathrm{IPW}}\left(\psi ;x,h_{n}\right)-D^{s}r\left(\psi ;x\right)-h_{n}^{\ell /p}B_{s,\ell }\left(x\right)\right|=o\left(h_{n}^{\ell /p}\right).$$

*In particular,*

$$\underset{x\in I}{\sup}\left|ED^{s}r_{n}^{\;\mathrm{IPW}}\left(\psi ;x,h_{n}\right)-D^{s}r\left(\psi ;x\right)\right|=O\left(h_{n}^{\ell /p}\right).$$

**Proof.**  The IPW identification identity gives

$$ED^{s}r_{n}^{\;\mathrm{IPW}}\left(\psi ;x,h_{n}\right)=\frac{1}{h_{n}^{1+|s|/p}}E\;\left[W_{0}\left(\psi \right)\kappa_{s}\left(\frac{x-X_{0}}{b_{n}}\right)\right].$$

After conditioning on $X_{0}$, changing variables, and integrating by parts,

$$ED^{s}r_{n}^{\;\mathrm{IPW}}\left(\psi ;x,h_{n}\right)=\int_{R^{p}}K\left(u\right)D^{s}r\left(\psi ;x-b_{n}u\right)\;du.$$

A multivariate Taylor expansion of $D^{s}r\left(\psi ;x-b_{n}u\right)$ through order *ℓ*, combined with the kernel moment cancellations, yields (47). Uniformity follows from the compact support of *K*, the positive distance between I and ∂J, and the uniform continuity of the derivatives of total order |s|+ℓ. □

**Remark 10.** 
*Missingness does not alter the oracle smoothing bias. Under correct propensity weighting, the expectation of the pseudo-response reproduces the complete-data conditional mean. Missingness enters the leading stochastic term through the second moment* (27).

### 3.4. Uniform Almost-Sure Stochastic Bounds

The next theorem separates the martingale fluctuation from the predictable dependence remainder.

**Theorem 1** 
(Uniform centred rate). *Assume* (S.1)–(S.3), (K.1), (P.1), (P.3), (U.1)–(U.3), *and* (H.2)*. Then,*

(48) $$\underset{x\in I}{\sup}\left|D^{s}r_{n}^{\;\mathrm{IPW}}\left(\psi ;x,h_{n}\right)-ED^{s}r_{n}^{\;\mathrm{IPW}}\left(\psi ;x,h_{n}\right)\right|=O_{a.s.}\left[{\left\{\frac{\log n}{nh_{n}^{1+2|s|/p}}\right\}}^{1/2}\right].$$

*More precisely,*

$$\underset{x\in I}{\sup}\left|M_{n,s}\left(x\right)\right|=O_{a.s.}\left[{\left\{\frac{\log n}{nh_{n}^{1+2|s|/p}}\right\}}^{1/2}\right]$$

*and*

$$\underset{x\in I}{\sup}\left|P_{n,s}\left(x\right)\right|=O_{a.s.}{\left\{\frac{\log n}{n}\right\}}^{1/2}.$$

*Because $h_{n}\to 0$, the predictable term is asymptotically no larger than the displayed martingale rate.*

The first bound is obtained from a conditional Bernstein inequality for the martingale differences, a polynomial covering of the translation class, and a Borel–Cantelli argument. The second follows directly from (33) and (P.3):

$$\underset{x\in I}{\sup}|P_{n,s}{\left(x\right)|\leq ∥K∥}_{1}\frac{1}{n}\underset{u\in J}{\sup}\left|\sum_{i=1}^{n}G_{i,\psi }^{\left(s\right)}\left(u\right)\right|.$$

Combining Theorem 1 with Proposition 2 yields the full uniform rate.

**Theorem 2** 
(Uniform strong consistency). *Assume the conditions of Theorem 1 and Proposition 2. Then,*

(49) $$\begin{array}{cc} \; & \underset{x\in I}{\sup}\left|D^{s}r_{n}^{\;\mathrm{IPW}}\left(\psi ;x,h_{n}\right)-D^{s}r\left(\psi ;x\right)\right|\\ \; & \;=O_{a.s.}\left[{\left\{\frac{\log n}{nh_{n}^{1+2|s|/p}}\right\}}^{1/2}\right]+O\left(h_{n}^{\ell /p}\right). \end{array}$$

*In particular,*

$$\underset{x\in I}{\sup}\left|D^{s}r_{n}^{\;\mathrm{IPW}}\left(\psi ;x,h_{n}\right)-D^{s}r\left(\psi ;x\right)\right|⟶0\;a.s.$$

**Corollary 1** 
(Marginal density derivatives). *Suppose the corresponding assumptions hold with the pseudo-response replaced by 1, with*

$$g_{i,1}\left(u\right)=f_{i}\left(u\right),$$

*and with $r\left(1;u\right)=f_{X}\left(u\right)$. Then,*

$$\underset{x\in I}{\sup}\left|D^{s}f_{X;n}\left(x,h_{n}\right)-D^{s}f_{X}\left(x\right)\right|=O_{a.s.}\left[{\left\{\frac{\log n}{nh_{n}^{1+2|s|/p}}\right\}}^{1/2}\right]+O\left(h_{n}^{\ell /p}\right).$$

**Remark 11** 
(Finite moments instead of Bernstein moments). *A finite conditional (2+η)-moment can be used for uniform consistency, but not by simply applying the Bernstein proof above. One must truncate $W_{i}\left(\psi \right)$ at a level $\tau_{n}$, establish the exponential bound for the truncated martingale array, and control the tail array separately. The choices of $\tau_{n}$, $h_{n}$, and the covering mesh must be balanced so that both tail probabilities are summable. Without such a calculation, the rate (48) is not justified by a (2+η)-moment alone.*

### 3.5. Pointwise Martingale Central Limit Theorem

Fix x∈I. Define

(50) $$\sigma_{\psi ,s}^{2}\left(x\right)=q_{\psi }\left(x\right)R_{s}\left(K\right)=f_{X}\left(x\right)\frac{E\{\psi^{2}\left(Y_{0}\right)|X_{0}=x\}}{\pi (x)}R_{s}\left(K\right).$$

The normalized martingale increments are

(51) $$d_{i,n}\left(x\right)=\frac{1}{\sqrt{nh_{n}}}\left[\Theta_{i,n}\left(x\right)-A_{i,n}\left(x\right)\right].$$

Indeed,

$$\sqrt{nh_{n}^{1+2|s|/p}}M_{n,s}\left(x\right)=\sum_{i=1}^{n}d_{i,n}\left(x\right).$$

The cancellation of the derivative order from the external normalization in (51) is a consequence of the volume–bandwidth parametrization.

**Theorem 3** 
(Centred pointwise CLT). *Assume* (S.1)–(S.3), (K.1), (P.1)–(P.2), (C.1)–(C.3)*, and* (H.1)*. Then,*

(52) $$\sqrt{nh_{n}^{1+2|s|/p}}\left[D^{s}r_{n}^{\;\mathrm{IPW}}\left(\psi ;x,h_{n}\right)-ED^{s}r_{n}^{\;\mathrm{IPW}}\left(\psi ;x,h_{n}\right)\right]\overset{D}{\to }N\left(0,\sigma_{\psi ,s}^{2}\left(x\right)\right).$$

The conditional-variance calculation underlying Theorem 3 is

(53) $$\sum_{i=1}^{n}E\left\{d_{i,n}^{2}\left(x\right)|G_{i-1}\right\}\overset{P}{\to }q_{\psi }\left(x\right)R_{s}\left(K\right).$$

The contribution of the squared conditional means is negligible because

$$A_{i,n}\left(x\right)=O_{P}\left(h_{n}^{1+|s|/p}\right),$$

uniformly locally, and therefore

$$\frac{1}{nh_{n}}\sum_{i=1}^{n}A_{i,n}^{2}\left(x\right)=O_{P}\left(h_{n}^{1+2|s|/p}\right)=o_{P}\left(1\right).$$

Assumption (C.2) implies the conditional Lindeberg condition because

$$\sum_{i=1}^{n}E\left[d_{i,n}^{2}\left(x\right)1\{|d_{i,n}\left(x\right)\left|>\varepsilon \right\}|G_{i-1}\right]=O_{P}\left\{{\left(nh_{n}\right)}^{-\eta /2}\right\}.$$

Finally, (P.2) gives

$$\sqrt{nh_{n}^{1+2|s|/p}}P_{n,s}\left(x\right)=O_{L^{2}}\left(h_{n}^{1/2+|s|/p}\right)=o_{L^{2}}\left(1\right).$$

**Theorem 4** 
(Target-centred pointwise CLT). *Assume the conditions of Theorem 3 and Proposition 2.*

1. *Under the undersmoothing condition* (H.3)*,*
  (54) $$\sqrt{nh_{n}^{1+2|s|/p}}\left[D^{s}r_{n}^{\;\mathrm{IPW}}\left(\psi ;x,h_{n}\right)-D^{s}r\left(\psi ;x\right)\right]\overset{D}{\to }N\left(0,\sigma_{\psi ,s}^{2}\left(x\right)\right).$$
2. *Under the finite-bias regime* (H.4)*,*
  (55) $$\sqrt{nh_{n}^{1+2|s|/p}}\left[D^{s}r_{n}^{\;\mathrm{IPW}}\left(\psi ;x,h_{n}\right)-D^{s}r\left(\psi ;x\right)\right]\overset{D}{\to }N\left(\lambda B_{s,\ell }\left(x\right),\sigma_{\psi ,s}^{2}\left(x\right)\right).$$

**Corollary 2** 
(Density-derivative CLT). *For the unweighted marginal-density estimator,*

$$q_{1}\left(x\right)=f_{X}\left(x\right),$$

*and therefore,*

$$\sigma_{f,s}^{2}\left(x\right)=f_{X}\left(x\right)R_{s}\left(K\right).$$

*Under the corresponding assumptions,*

$$\sqrt{nh_{n}^{1+2|s|/p}}\left[D^{s}f_{X;n}\left(x,h_{n}\right)-ED^{s}f_{X;n}\left(x,h_{n}\right)\right]\overset{D}{\to }N\left(0,\sigma_{f,s}^{2}\left(x\right)\right).$$

*The target-centred form follows under the corresponding bias condition.*

### 3.6. A Joint Primitive-Estimator Central Limit Theorem

Regression derivatives are nonlinear functions of several numerator and density derivatives. Their limit theory requires a joint central limit theorem, not a collection of unrelated marginal limits.

Let d≥1. For a=1,…,d, let $V_{i}^{\left(a\right)}$ be either an IPW pseudo-response

$$V_{i}^{\left(a\right)}=W_{i}\left(\psi_{a}\right)$$

or the constant 1, and let $s_{a}\in N_{0}^{p}.$ Define

$$T_{n,a}\left(x\right)=\frac{1}{nh_{n}^{1+|s_{a}|/p}}\sum_{i=1}^{n}V_{i}^{\left(a\right)}D^{s_{a}}K\left(\frac{x-X_{i}}{b_{n}}\right).$$

Let $\theta_{a}\left(x\right)$ denote the corresponding population derivative:

$$\theta_{a}\left(x\right)=\left\{\begin{array}{cc} D^{s_{a}}r\left(\psi_{a};x\right), & V_{i}^{\left(a\right)}=W_{i}\left(\psi_{a}\right),\\ D^{s_{a}}f_{X}\left(x\right), & V_{i}^{\left(a\right)}=1. \end{array}\right.$$

For a,b∈{1,…,d}, define

(56) $$q_{ab}\left(x\right)=f_{X}\left(x\right)E\;\left[V_{0}^{\left(a\right)}V_{0}^{\left(b\right)}|X_{0}=x\right].$$

Important special cases are

$$q_{ab}\left(x\right)=\frac{f_{X}\left(x\right)E\left\{\psi_{a}\left(Y_{0}\right)\psi_{b}\left(Y_{0}\right)|X_{0}=x\right\}}{\pi (x)}$$

when both entries are IPW pseudo-responses,

$$q_{ab}\left(x\right)=r\left(\psi_{a};x\right)$$

when $V^{\left(a\right)}=W\left(\psi_{a}\right)$ and $V^{\left(b\right)}=1$, and

$$q_{ab}\left(x\right)=f_{X}\left(x\right)$$

when both entries equal 1.

Set

(57) $$\Sigma_{ab}\left(x\right)=q_{ab}\left(x\right)\int_{R^{p}}D^{s_{a}}K\left(u\right)D^{s_{b}}K\left(u\right)\;du.$$

**Theorem 5** 
(Joint primitive CLT). *Suppose that the conditional first- and second-moment stabilization assumptions, the predictable $L^{2}$ conditions, and the conditional Lindeberg conditions hold jointly for $\left\{V_{i}^{\left(a\right)}:1\leq a\leq d\right\}$, including stabilization of every conditional cross-moment. Then,*

(58) $${\left(\sqrt{nh_{n}^{1+2|s_{a}|/p}}\left[T_{n,a}\left(x\right)-ET_{n,a}\left(x\right)\right]\right)}_{a=1}^{d}\overset{D}{\to }N_{d}\left(0,\Sigma (x)\right),$$

*where*

$$\Sigma \left(x\right)={\left\{\Sigma_{ab}\left(x\right)\right\}}_{a,b=1}^{d}.$$

*If the corresponding bias terms are negligible at their respective normalizations, $ET_{n,a}\left(x\right)$ may be replaced by $\theta_{a}\left(x\right)$.*

The theorem follows by the Cramér–Wold device applied to the vector martingale array. It remains valid when the covariance matrix is singular. This may occur, for example, when two components are algebraically redundant. The mixed normalizations in (58) are essential when the derivative orders $|s_{a}|$ differ.

### 3.7. Direct Variance Expansion

Convergence in distribution does not imply convergence of variances. The next result therefore derives the variance expansion under explicit second-moment and uniform-integrability assumptions.

**Proposition 3** 
(Pointwise variance expansion). *Assume the conditions of Theorem 3, and in addition, assume uniform integrability of the squared normalized estimator and*

$$nh_{n}^{1+2|s|/p}\mathrm{Var}\left\{P_{n,s}\left(x\right)\right\}⟶0.$$

*Then,*

(59) $$\mathrm{Var}\;\left\{D^{s}r_{n}^{\;\mathrm{IPW}}\left(\psi ;x,h_{n}\right)\right\}=\frac{\sigma_{\psi ,s}^{2}\left(x\right)}{nh_{n}^{1+2|s|/p}}+o\;\left(\frac{1}{nh_{n}^{1+2|s|/p}}\right).$$

The proof is based on direct conditional second-moment calculations for the martingale component and a separate control of the predictable component. It does not infer (59) from the CLT.

### 3.8. Asymptotic Mean Integrated Squared Error

Define

(60) $${\mathrm{MISE}}_{n}\left(h_{n}\right)=E\int_{I}{\left[D^{s}r_{n}^{\;\mathrm{IPW}}\left(\psi ;x,h_{n}\right)-D^{s}r\left(\psi ;x\right)\right]}^{2}\;dx.$$

Let

(61) $$B_{s,\ell }=\int_{I}B_{s,\ell }^{2}\left(x\right)\;dx$$

and

(62) $$V_{s,\psi }=R_{s}\left(K\right)\int_{I}q_{\psi }\left(x\right)\;dx.$$

Under MAR,

(63) $$V_{s,\psi }=R_{s}\left(K\right)\int_{I}f_{X}\left(x\right)\frac{E\{\psi^{2}\left(Y_{0}\right)|X_{0}=x\}}{\pi (x)}\;dx.$$

**Theorem 6** 
(AMISE expansion). *Assume* (S.1)–(S.3)*,* (K.1)–(K.2)*,* (R.1)–(R.2)*,* (P.1)–(P.2)*,* (C.1)–(C.3)*,* (H.1)*, and* (I.1)–(I.3)*. Then,*

(64) $${\mathrm{MISE}}_{n}\left(h_{n}\right)=h_{n}^{2\ell /p}B_{s,\ell }+\frac{V_{s,\psi }}{nh_{n}^{1+2|s|/p}}+o\;\left[h_{n}^{2\ell /p}+\frac{1}{nh_{n}^{1+2|s|/p}}\right].$$

*The first two terms on the right-hand side constitute the asymptotic mean integrated squared error:*

$${\mathrm{AMISE}}_{n}\left(h\right)=h^{2\ell /p}B_{s,\ell }+\frac{V_{s,\psi }}{nh^{1+2|s|/p}}.$$

Assume

$$0<B_{s,\ell }<\infty ,\;0<V_{s,\psi }<\infty .$$

Differentiating the AMISE with respect to *h* gives

$$\frac{2\ell }{p}B_{s,\ell }h^{2\ell /p-1}-\frac{p+2|s|}{p}\frac{V_{s,\psi }}{n}h^{-(p+2|s|)/p-1}=0.$$

Therefore,

(65) $$h_{n,\mathrm{AMISE}}={\left\{\frac{\left(p+2|s|\right)V_{s,\psi }}{2\ell B_{s,\ell }\;n}\right\}}^{\;p/(p+2|s|+2\ell )}.$$

Equivalently,

$$h_{n,\mathrm{AMISE}}≍n^{-p/(p+2|s|+2\ell )}.$$

At this bandwidth,

(66) $$\underset{h>0}{\inf}{\mathrm{AMISE}}_{n}\left(h\right)≍n^{-2\ell /(p+2|s|+2\ell )}.$$

An explicit expression for the minimized AMISE is obtained from the identity

$$\frac{2\ell }{p}B_{s,\ell }h_{n,\mathrm{AMISE}}^{2\ell /p}=\frac{p+2|s|}{p}\frac{V_{s,\psi }}{nh_{n,\mathrm{AMISE}}^{1+2|s|/p}}.$$

Hence,

(67) $$\begin{array}{cc} {\mathrm{AMISE}}_{n}\left(h_{n,\mathrm{AMISE}}\right)\; & =\frac{p+2|s|+2\ell }{p+2|s|}B_{s,\ell }h_{n,\mathrm{AMISE}}^{2\ell /p}\\ \; & =\frac{p+2|s|+2\ell }{2\ell }\frac{V_{s,\psi }}{nh_{n,\mathrm{AMISE}}^{1+2|s|/p}}. \end{array}$$

**Remark 12** 
(AMISE bandwidth and inference bandwidth). *The AMISE-optimal bandwidth generally produces a non-negligible leading bias under the pointwise Gaussian normalization. Consequently, it is not normally appropriate for an uncorrected target-centred confidence interval. Pointwise inference requires undersmoothing, explicit bias correction, or a robust bias-corrected procedure. Estimation-optimal and inference-valid bandwidths solve different problems.*

Dimension and derivative order.

The theory is formulated for a fixed covariate dimension *p*, but the AMISE rate $n^{-2\ell /(p+2|s|+2\ell )}$ makes the curse of dimensionality and the statistical cost of differentiation explicit. Increasing either *p* or |s| slows the attainable rate, raises the effective sample-size requirement, and makes bandwidth choice and residual bias more consequential. Direct multivariate smoothing is therefore most practically credible in low- to moderate-dimensional problems. In higher dimensions, one would normally require additional structure such as dimension reduction, additive or sparse modelling, coordinate-specific smoothing, or anisotropic bandwidths. Likewise, the general higher-order theory should not be read as implying equal finite-sample reliability across orders: first derivatives are typically more stable, whereas second and higher derivatives can remain bias- and variance-dominated unless the sample is substantially larger and smoothness is strong.

### 3.9. Estimation of the Asymptotic Variance

Because

$$\sigma_{\psi ,s}^{2}\left(x\right)=R_{s}\left(K\right)q_{\psi }\left(x\right),$$

it is unnecessary to estimate $f_{X}\left(x\right)$ and $\Psi_{2}^{\mathrm{IPW}}\left(x,\psi \right)$ separately. Their product can be estimated directly.

Let $L:R^{p}\to \left[0,\infty \right)$ be a bounded, compactly supported pilot kernel satisfying

$$\int_{R^{p}}L\left(u\right)\;du=1.$$

Let $c_{n}↓0$ be a pilot volume bandwidth. Define

(68) $${\hat{q}}_{\psi ,n}\left(x,c_{n}\right)=\frac{1}{nc_{n}}\sum_{i=1}^{n}W_{i}^{2}\left(\psi \right)L\left(\frac{x-X_{i}}{c_{n}^{1/p}}\right)$$

and

(69) $${\hat{\sigma }}_{\psi ,s}^{2}\left(x\right)=R_{s}\left(K\right){\hat{q}}_{\psi ,n}\left(x,c_{n}\right).$$

**Proposition 4** 
(Consistency of the variance estimator). *Suppose that the order-zero pointwise consistency assumptions apply to the transformed response $W_{i}^{2}\left(\psi \right)$, with target $q_{\psi }$. In particular, assume that $q_{\psi }$ is continuous at x,*

$$c_{n}\to 0,\;nc_{n}\to \infty ,$$

*the corresponding predictable remainder is negligible, and a conditional moment of order strictly greater than two is available for $W_{i}^{2}\left(\psi \right)$. Then,*

$${\hat{q}}_{\psi ,n}\left(x,c_{n}\right)\overset{P}{\to }q_{\psi }\left(x\right)$$

*and*

(70) $${\hat{\sigma }}_{\psi ,s}^{2}\left(x\right)\overset{P}{\to }\sigma_{\psi ,s}^{2}\left(x\right).$$

A sufficient moment condition for a direct application of a (2+δ)-moment argument to $W_{i}^{2}\left(\psi \right)$ is a conditional moment of order 4+2δ for $W_{i}\left(\psi \right)$. Under positivity, this translates into a corresponding conditional moment assumption on $\psi (Y_{i})$.

Under the target-centred CLT and (70),

(71) $$\frac{\sqrt{nh_{n}^{1+2|s|/p}}\left[D^{s}r_{n}^{\;\mathrm{IPW}}\left(\psi ;x,h_{n}\right)-D^{s}r\left(\psi ;x\right)\right]}{{\hat{\sigma }}_{\psi ,s}\left(x\right)}\overset{D}{\to }N\left(0,1\right).$$

Accordingly, an asymptotic pointwise 100(1−α)% confidence interval is

(72) $$D^{s}r_{n}^{\;\mathrm{IPW}}\left(\psi ;x,h_{n}\right)\pm z_{1-\alpha /2}\frac{{\hat{\sigma }}_{\psi ,s}\left(x\right)}{\sqrt{nh_{n}^{1+2|s|/p}}}.$$

Interval (72) is target-valid under undersmoothing. At an AMISE-optimal bandwidth, a bias estimate must be subtracted or a robust bias-corrected construction must be used.

### 3.10. Transfer from Oracle to Feasible Propensity Weighting

Let

$${\hat{\pi }}_{n}^{†}\left(x\right)={\hat{\pi }}_{n}\left(x\right)\vee {\underline{\pi }}_{n}$$

be the stabilized estimator introduced in Section 2, and define

$$\delta_{n}={∥{\hat{\pi }}_{n}^{†}-\pi ∥}_{\infty ,J}.$$

On the event

$$\delta_{n}\leq \pi_{★}/2,$$

the inverse map is Lipschitz:

$$\underset{u\in J}{\sup}\left|\frac{1}{{\hat{\pi }}_{n}^{†}\left(u\right)}-\frac{1}{\pi (u)}\right|\leq \frac{2}{\pi_{★}^{2}}\delta_{n}.$$

Define the local empirical envelope

(73) $$A_{n,\psi ,s}=\underset{x\in I}{\sup}\frac{1}{nh_{n}}\sum_{i=1}^{n}\xi_{i}\left|\psi \left(Y_{i}\right)\right|\left|D^{s}K\left(\frac{x-X_{i}}{b_{n}}\right)\right|.$$

**Proposition 5** 
(Oracle–feasible deterministic comparison). *On the event $\left\{\delta_{n}\leq \pi_{★}/2\right\}$,*

(74) $$\begin{array}{cc} \; & \underset{x\in I}{\sup}\left|D^{s}{\hat{r}}_{n}^{\;\mathrm{IPW}}\left(\psi ;x,h_{n}\right)-D^{s}r_{n}^{\;\mathrm{IPW}}\left(\psi ;x,h_{n}\right)\right|\\ \; & \;\leq \frac{2}{\pi_{★}^{2}}\delta_{n}h_{n}^{-|s|/p}A_{n,\psi ,s}. \end{array}$$

*If*

$$A_{n,\psi ,s}=O_{P}\left(1\right),$$

*then*

(75) $$\underset{x\in I}{\sup}\left|D^{s}{\hat{r}}_{n}^{\;\mathrm{IPW}}-D^{s}r_{n}^{\;\mathrm{IPW}}\right|=O_{P}\left(\delta_{n}h_{n}^{-|s|/p}\right).$$

The comparison gives transparent, albeit conservative, sufficient conditions.

1. For oracle-equivalent uniform consistency, it is sufficient that
  (76) $$\delta_{n}h_{n}^{-|s|/p}⟶0\;\mathrm{in}\;\mathrm{probability}.$$
2. To preserve the oracle *overall uniform estimation rate* (stochastic fluctuation plus deterministic smoothing bias), it is sufficient that
  (77) $$\delta_{n}h_{n}^{-|s|/p}=o_{P}\left[{\left\{\frac{\log n}{nh_{n}^{1+2|s|/p}}\right\}}^{1/2}+h_{n}^{\ell /p}\right].$$
  If the objective is specifically to preserve only the centred oracle stochastic rate, the corresponding sufficient requirement is the sharper comparison
  $$\delta_{n}h_{n}^{-|s|/p}=o_{P}\;\left[{\left\{\frac{\log n}{nh_{n}^{1+2|s|/p}}\right\}}^{1/2}\right].$$
  The right-hand side contains only the centred stochastic term and no smoothing-bias term.
3. For oracle-equivalent pointwise Gaussian inference, it is sufficient that
  (78) $$\sqrt{nh_{n}}\;\delta_{n}⟶0\;\mathrm{in}\;\mathrm{probability}.$$
  Indeed, multiplying (75) by the CLT normalization cancels the derivative factor:
  $$\sqrt{nh_{n}^{1+2|s|/p}}\delta_{n}h_{n}^{-|s|/p}=\sqrt{nh_{n}}\;\delta_{n}.$$

Condition (78) is often demanding for a nonparametric propensity estimator. Slower nuisance rates require a sharper stochastic expansion, sample splitting, or an orthogonal or doubly robust estimating equation. Mere uniform consistency of ${\hat{\pi }}_{n}$ is not sufficient for oracle-equivalent inference.

### 3.11. Logical Scope of the Results

The assumptions above have deliberately been separated according to their logical roles:

1. (S.1)–(S.3) define the stationary observed-data experiment and identify the full-data target under sequential MAR and positivity.
2. (K.1)–(K.2) and (R.1)–(R.2) determine the deterministic bias and its leading constant.
3. (P.1)–(P.3) control the predictable component generated by the past-dependent conditional law. These conditions are not consequences of ergodicity alone.
4. (U.1)–(U.3) are used for the exponential maximal inequality and the almost-sure uniform rate. They are stronger than the moment assumptions needed for a pointwise CLT.
5. (C.1)–(C.3) control the conditional quadratic variation and Lindeberg remainder of the martingale array.
6. (I.1)–(I.3) are required for an actual AMISE expansion with explicit constants. A pointwise CLT alone cannot establish such an expansion.
7. The feasible propensity result requires its own rate conditions. It is not a formal consequence of the oracle theory.

The phrase “under stationary ergodic dependence” must therefore be read together with the quantitative modules invoked by each theorem. The theory does not assume a mixing coefficient, but neither does it claim that every stationary ergodic process satisfies the required maximal, projective, or conditional-variance stabilization properties. For any proposed non-mixing example, those properties must be verified directly or through an appropriate martingale approximation, physical-dependence estimate, coupling argument, or functional projective criterion. The modules above are transparent *sufficient* conditions and are not claimed to be minimal. Kernel/smoothness conditions control deterministic bias; positivity controls identification and IPW moments; projective/maximal conditions control the predictable dependence term; Bernstein or (2+η)-moment assumptions provide uniform exponential control or pointwise Lindeberg control, respectively; and local variance stabilization identifies the Gaussian variance. Alternative dependence hypotheses may replace the chosen modules whenever they supply these same probabilistic inputs. Failure of a module has a correspondingly specific consequence: for example, loss of positivity destroys local identification, failure of the maximal condition can invalidate the uniform rate, and failure of conditional variance stabilization can invalidate the stated Gaussian variance even if ergodicity itself remains true.

## 4. Application to the Regression Derivatives

This section transfers the primitive kernel-derivative theory of Section 3 to derivatives of conditional regression functionals. The transfer is nonlinear: a regression derivative is a finite-dimensional rational functional of the complete collection of numerator and density derivatives of lower or equal order. Consequently, marginal consistency or marginal central limit theorems for the primitive estimators are not sufficient. The analysis requires denominator separation, joint primitive-estimator limits, explicit control of the nonlinear Taylor remainder, and propagation of every primitive smoothing-bias component through the quotient map.

For a Borel function $\psi :R^{q}\to R$, define

(79) $$m\left(\psi ;x\right)=E\left\{\psi \left(Y_{0}\right)|X_{0}=x\right\}=\frac{r(\psi ;x)}{f_{X}\left(x\right)},\;x\in J.$$

Throughout this section, the regression functional is studied only on a region where the design density is separated from zero:

(80) $$f_{★}:=\underset{x\in J}{\inf}f_{X}\left(x\right)>0.$$

The numerator is estimated by inverse probability weighting because it depends on the incompletely observed response. The denominator is estimated directly from the complete covariate sample because $X_{i}$ is always observed. This construction estimates the two population objects appearing in (79) without introducing a response indicator into the marginal-density estimator. No universal finite-sample variance ordering between this ratio and every alternative weighted-denominator ratio is asserted: such an ordering depends on the full numerator–denominator covariance structure.

All assertions below are conditional on the corresponding primitive assumptions of Section 3. In particular, stationarity and ergodicity alone are not used to infer a rate, a maximal inequality, or a Gaussian limit.

### 4.1. Exact Differential Calculus for a Regression Quotient

The algebraic identities in this subsection are deterministic. They define the population derivatives and the corresponding plug-in estimators; no asymptotic approximation is used.

#### 4.1.1. Univariate Covariate

Suppose first that p=1, and write

$$r_{\psi }\left(x\right)=r\left(\psi ;x\right),\;f\left(x\right)=f_{X}\left(x\right),\;m_{\psi }\left(x\right)=m\left(\psi ;x\right).$$

Then, $m_{\psi }=r_{\psi }f^{-1}$. Direct differentiation gives

(81) $$m_{\psi }^{\prime }\left(x\right)=\frac{r_{\psi }^{\prime }\left(x\right)}{f(x)}-\frac{r_{\psi }\left(x\right)f^{\prime }\left(x\right)}{f^{2}\left(x\right)}=\frac{r_{\psi }^{\prime }\left(x\right)f\left(x\right)-r_{\psi }\left(x\right)f^{\prime }\left(x\right)}{f^{2}\left(x\right)}.$$

A second differentiation gives

(82) $$\begin{array}{cc} m_{\psi }^{\prime \prime }\left(x\right)\; & =\frac{r_{\psi }^{\prime \prime }\left(x\right)}{f(x)}-\frac{2r_{\psi }^{\prime }\left(x\right)f^{\prime }\left(x\right)}{f^{2}\left(x\right)}\\ \; & \;+\frac{r_{\psi }\left(x\right)\left[2{\left\{f^{\prime }\left(x\right)\right\}}^{2}-f\left(x\right)f^{\prime \prime }\left(x\right)\right]}{f^{3}\left(x\right)}. \end{array}$$

For an integer k≥0, Leibniz’s formula yields

(83) mψ(k)(x)=∑j=0kkjrψ(j)(x){f−1}(k−j)(x).

The reciprocal derivatives can be generated without an ambiguous expansion. Set

(84) $$q_{0}\left(x\right)=f^{-1}\left(x\right),$$

and, for a≥1, define

(85) qa(x)=−1f(x)∑j=1aajf(j)(x)qa−j(x).

Indeed, differentiating $fq_{0}=1$ exactly *a* times gives

0=∑j=0aajf(j)qa−j,

which isolates the term $fq_{a}$ and proves that $q_{a}={\left(f^{-1}\right)}^{\left(a\right)}$.

For completeness, the same derivative admits the Bell-polynomial expression

(86) $${\left\{f^{-1}\right\}}^{\left(a\right)}\left(x\right)=\sum_{\nu =1}^{a}{\left(-1\right)}^{\nu }\nu !f{\left(x\right)}^{-\nu -1}B_{a,\nu }\left(f^{\prime }\left(x\right),\ldots ,f^{\left(a-\nu +1\right)}\left(x\right)\right),\;a\geq 1,$$

where $B_{a,\nu }$ is the partial exponential Bell polynomial. Formula (86) follows from the one-dimensional Faà di Bruno formula applied to the map $u\mapsto u^{-1}$; the recursion (85) is preferable for numerical implementation.

#### 4.1.2. Multivariate Covariate

For multi-indices $\alpha ,\beta \in N_{0}^{p}$, write

$$\beta \leq \alpha \;⟺\;0\leq \beta_{j}\leq \alpha_{j}\;\left(j=1,\ldots ,p\right),$$

and

αβ=∏j=1pαjβj.

The multi-index Leibniz rule gives

(87) Dsm(ψ;x)=∑j≤ssjDjr(ψ;x)Ds−j{fX−1}(x).

To compute the reciprocal derivatives, set

(88) $$Q_{0}\left(x\right)=f_{X}^{-1}\left(x\right),$$

and, for every nonzero multi-index α, define

(89) Qα(x)=−1fX(x)∑0<β≤ααβDβfX(x)Qα−β(x).

Applying $D^{\alpha }$ to $f_{X}f_{X}^{-1}=1$ proves recursively that

$$Q_{\alpha }=D^{\alpha }\left(f_{X}^{-1}\right).$$

For a fixed s, let

$$I_{s}=\left\{\alpha \in N_{0}^{p}:\alpha \leq s\right\},$$

and define the finite jets

$$J_{s}r\left(x\right)={\left(D^{\alpha }r\left(\psi ;x\right)\right)}_{\alpha \in I_{s}},\;J_{s}f\left(x\right)={\left(D^{\alpha }f_{X}\left(x\right)\right)}_{\alpha \in I_{s}}.$$

For arrays $a=(a_{\alpha })$ and $b=(b_{\alpha })$, with $b_{0}>0$, define

(90) $$T_{s}\left(a,b\right)$$

to be the rational differential polynomial obtained from (87) and (89) after replacing $D^{\alpha }r$ with $a_{\alpha }$ and $D^{\alpha }f_{X}$ with $b_{\alpha }$. Thus,

$$D^{s}m\left(\psi ;x\right)=T_{s}\left\{J_{s}r\left(x\right),J_{s}f\left(x\right)\right\}.$$

**Proposition 6** 
(Smooth rational structure of the quotient jet). *For every fixed s, the map $T_{s}$ is a rational function of finitely many real variables whose only possible singularity is at $b_{0}=0$. It is therefore $C^{\infty }$ on*

$$D_{s}=\left\{\left(a,b\right):b_{0}>0\right\}.$$

*On every compact subset of $D_{s}$, all derivatives of $T_{s}$ are bounded.*

*If a,b,c,d are sufficiently differentiable scalar functions with f>0, the first and second Fréchet variations of the quotient satisfy*

(91) $$DQ_{\left(r,f\right)}\left[a,b\right]=\frac{a}{f}-\frac{rb}{f^{2}},\;Q\left(r,f\right)=\frac{r}{f},$$

*and*

(92) $$D^{2}Q_{\left(r,f\right)}\left[\left(a,b\right),\left(c,d\right)\right]=-\frac{ad+bc}{f^{2}}+\frac{2rbd}{f^{3}}.$$

*Consequently,*

(93) $$DT_{s}\left\{J_{s}r,J_{s}f\right\}\left[J_{s}a,J_{s}b\right]=D^{s}\left(\frac{a}{f}-\frac{rb}{f^{2}}\right).$$

**Proof.**  The reciprocal recursion shows inductively that every $Q_{\alpha }$ is a polynomial in the derivatives $D^{\beta }f$, 0<β≤α, divided by a positive integer power of *f*. Substitution into (87) proves the rational structure and, therefore, smoothness on $b_{0}>0$. Boundedness of all derivatives on compact subsets follows from continuity.

For |t| sufficiently small,

$$\frac{r+ta}{f+tb}=\left(r+ta\right){\left(f+tb\right)}^{-1}.$$

Differentiation at t=0 proves (91). Applying a second directional derivative, first in direction (a,b) and then in direction (c,d), gives (92). Finally, differentiation with respect to x is a continuous linear operation on the finite jet, so it commutes with the directional derivative and yields (93). □

### 4.2. Primitive Estimators and Stabilized Plug-In Derivatives

For every α≤s, define

(94) $${\hat{r}}_{\psi ,n}^{(\alpha ),\mathrm{IPW}}\left(x\right)=D^{\alpha }r_{n}^{\;\mathrm{IPW}}\left(\psi ;x,h_{n}\right)$$

and

(95) $${\hat{f}}_{n}^{\left(\alpha \right)}\left(x\right)=D^{\alpha }f_{X;n}\left(x,h_{n}\right).$$

Explicitly,

$${\hat{r}}_{\psi ,n}^{(\alpha ),\mathrm{IPW}}\left(x\right)=\frac{1}{nh_{n}^{1+|\alpha |/p}}\sum_{i=1}^{n}W_{i}\left(\psi \right)D^{\alpha }K\left(\frac{x-X_{i}}{h_{n}^{1/p}}\right),$$

and

$${\hat{f}}_{n}^{\left(\alpha \right)}\left(x\right)=\frac{1}{nh_{n}^{1+|\alpha |/p}}\sum_{i=1}^{n}D^{\alpha }K\left(\frac{x-X_{i}}{h_{n}^{1/p}}\right).$$

Because kernels of order greater than two may be signed, the order-zero density estimator need not be positive in a finite sample. Let $\tau_{n}↓0$, and define the stabilized base density

(96) $${\bar{f}}_{n}\left(x\right)={\hat{f}}_{n}^{\left(0\right)}\left(x\right)\vee \tau_{n}.$$

This operation stabilizes only the base density appearing in the denominators; it is not interpreted as differentiation of the non-smooth map $u\mapsto u\vee \tau_{n}$. The derivative coordinates ${\hat{f}}_{n}^{\left(\alpha \right)}$, |α|≥1, remain the ordinary kernel derivative estimators. On the event

$$E_{n}=\left\{\underset{x\in I}{\inf}{\hat{f}}_{n}^{\left(0\right)}\left(x\right)\geq f_{★}/2\right\},$$

trimming is inactive whenever $\tau_{n}<f_{★}/2$.

Define

(97) $${\hat{Q}}_{0,n}\left(x\right)={\bar{f}}_{n}^{-1}\left(x\right),$$

and, for α≠0, recursively set

(98) Q^α,n(x)=−1f¯n(x)∑0<β≤ααβf^n(β)(x)Q^α−β,n(x).

**Definition 5** 
(Multivariate regression-derivative estimator). *For $s\in N_{0}^{p}$, define*

(99) Dsm^ψ,nIPW(x)=∑j≤ssjr^ψ,n(j),IPW(x)Q^s−j,n(x).

For s=0, this becomes

(100) $${\hat{m}}_{\psi ,n}^{\mathrm{IPW}}\left(x\right)=\frac{{\hat{r}}_{\psi ,n}^{(0),\mathrm{IPW}}\left(x\right)}{{\bar{f}}_{n}\left(x\right)}.$$

In the univariate case, p=1, the primitive estimators are

(101) $${\hat{r}}_{\psi ,n}^{(j),\mathrm{IPW}}\left(x\right)=\frac{1}{nh_{n}^{1+j}}\sum_{i=1}^{n}\frac{\xi_{i}\psi \left(Y_{i}\right)}{\pi (X_{i})}K^{\left(j\right)}\left(\frac{x-X_{i}}{h_{n}}\right),\;j\geq 0,$$

and

(102) $${\hat{f}}_{X,n}^{\left(j\right)}\left(x\right)=\frac{1}{nh_{n}^{1+j}}\sum_{i=1}^{n}K^{\left(j\right)}\left(\frac{x-X_{i}}{h_{n}}\right),\;j\geq 0.$$

Writing ${\bar{f}}_{n}\left(x\right)={\hat{f}}_{X,n}^{\left(0\right)}\left(x\right)\vee \tau_{n}$, the stabilized first derivative estimator is

(103) $${\hat{m}}_{\psi ,n}^{\prime ,\mathrm{IPW}}\left(x\right)=\frac{{\hat{r}}_{\psi ,n}^{(1),\mathrm{IPW}}\left(x\right)}{{\bar{f}}_{n}\left(x\right)}-\frac{{\hat{r}}_{\psi ,n}^{(0),\mathrm{IPW}}\left(x\right){\hat{f}}_{X,n}^{\left(1\right)}\left(x\right)}{{\bar{f}}_{n}^{2}\left(x\right)}.$$

The stabilized second derivative estimator is

(104) $$\begin{array}{cc} {\hat{m}}_{\psi ,n}^{\prime \prime ,\mathrm{IPW}}\left(x\right)\; & =\frac{{\hat{r}}_{\psi ,n}^{(2),\mathrm{IPW}}\left(x\right)}{{\bar{f}}_{n}\left(x\right)}-\frac{2{\hat{r}}_{\psi ,n}^{(1),\mathrm{IPW}}\left(x\right){\hat{f}}_{X,n}^{\left(1\right)}\left(x\right)}{{\bar{f}}_{n}^{2}\left(x\right)}\\ \; & \;+\frac{{\hat{r}}_{\psi ,n}^{(0),\mathrm{IPW}}\left(x\right)\left[2{\left\{{\hat{f}}_{X,n}^{\left(1\right)}\left(x\right)\right\}}^{2}-{\bar{f}}_{n}\left(x\right){\hat{f}}_{X,n}^{\left(2\right)}\left(x\right)\right]}{{\bar{f}}_{n}^{3}\left(x\right)}. \end{array}$$

On $E_{n}$, these formulae coincide exactly with the first and second derivatives of the untrimmed kernel ratio.

For a fixed integer k≥0, define

(105) m^ψ,n(k),IPW(x)=∑j=0kkjr^ψ,n(j),IPW(x)q^k−j,n(x),

where ${\hat{q}}_{0,n}={\bar{f}}_{n}^{-1}$ and

(106) q^a,n(x)=−1f¯n(x)∑j=1aajf^X,n(j)(x)q^a−j,n(x),a≥1.

**Remark 13** 
(Algebraic interpretation). *When the same kernel and bandwidth are used for every primitive derivative and trimming is inactive, (99) is algebraically identical to*

$$D^{s}\left\{\frac{r_{n}^{\;\mathrm{IPW}}\left(\psi ;x,h_{n}\right)}{f_{X;n}\left(x,h_{n}\right)}\right\}.$$

*The recursive representation is nevertheless essential for the asymptotic analysis because it displays the primitive derivative orders, their mixed normalizations, and their covariance structure.*

### 4.3. Uniform Well-Posedness and Consistency

For α≤s, define

(107) $$\Delta_{n,\alpha }^{r}=\underset{x\in I}{\sup}\left|{\hat{r}}_{\psi ,n}^{(\alpha ),\mathrm{IPW}}\left(x\right)-D^{\alpha }r\left(\psi ;x\right)\right|$$

and

(108) $$\Delta_{n,\alpha }^{f}=\underset{x\in I}{\sup}\left|{\hat{f}}_{n}^{\left(\alpha \right)}\left(x\right)-D^{\alpha }f_{X}\left(x\right)\right|.$$

The order-zero density consistency and (80) imply that

(109) $$P(E_{n})⟶1.$$

If the primitive convergence is almost sure, then $E_{n}$ occurs for all sufficiently large *n*, almost surely.

**Lemma 1** 
(Local Lipschitz stability of the quotient derivative). *Fix $s\in N_{0}^{p}$. Assume* (80) *and boundedness on I of all derivatives*

$$D^{\alpha }r\left(\psi ;\cdot \right),\;D^{\alpha }f_{X},\;\alpha \leq s.$$

*There exist finite constants $C_{s}$ and $\varepsilon_{s}>0$, depending only on the population jets, the index s, and $f_{★}$, such that, whenever*

$$\max_{\alpha \leq s}\left(\Delta_{n,\alpha }^{r}+\Delta_{n,\alpha }^{f}\right)\leq \varepsilon_{s}$$

*and $\tau_{n}<f_{★}/2$,*

(110) $$\begin{array}{cc} \; & \underset{x\in I}{\sup}\left|D^{s}{\hat{m}}_{\psi ,n}^{\;\mathrm{IPW}}\left(x\right)-D^{s}m\left(\psi ;x\right)\right|\\ \; & \;\leq C_{s}\sum_{\alpha \leq s}\left(\Delta_{n,\alpha }^{r}+\Delta_{n,\alpha }^{f}\right). \end{array}$$

**Proof.**  Let

$$z\left(x\right)=\left\{J_{s}r\left(x\right),J_{s}f\left(x\right)\right\},\;{\hat{z}}_{n}\left(x\right)=\left\{J_{s}{\hat{r}}_{n}\left(x\right),J_{s}{\hat{f}}_{n}\left(x\right)\right\}.$$

Choose $\varepsilon_{s}\leq f_{★}/2$. Then, every convex combination of z(x) and ${\hat{z}}_{n}\left(x\right)$ has a base-density coordinate of at least $f_{★}/2$. The population jets are bounded on the compact set I, and the estimated jets lie in a fixed bounded enlargement. The line segments therefore lie in a compact subset $K_{s}\subset D_{s}$. By Proposition 6,

$$C_{s}^{\prime }:=\underset{z\in K_{s}}{\sup}{∥DT_{s}\left(z\right)∥}_{\mathrm{op}}<\infty .$$

The integral mean-value formula gives

$$\begin{array}{cc} \; & T_{s}\left({\hat{z}}_{n}\left(x\right)\right)-T_{s}\left(z\left(x\right)\right)\\ \; & \;=\int_{0}^{1}DT_{s}\left(z\left(x\right)+t\{{\hat{z}}_{n}\left(x\right)-z\left(x\right)\}\right)\left[{\hat{z}}_{n}\left(x\right)-z\left(x\right)\right]\;dt. \end{array}$$

The Euclidean norm of the finite jet difference is bounded by a constant times the sum of the coordinate errors. Taking suprema proves (110). □

**Corollary 3** 
(Uniform consistency of first and second derivatives). *Assume that Theorem 2 applies to the numerator and density derivatives of orders zero, one, and two, and assume (80). Then,*

$$\underset{x\in I}{\sup}\left|{\hat{m}}_{\psi ,n}^{\prime ,\mathrm{IPW}}\left(x\right)-m_{\psi }^{\prime }\left(x\right)\right|⟶0$$

*and*

$$\underset{x\in I}{\sup}\left|{\hat{m}}_{\psi ,n}^{\prime \prime ,\mathrm{IPW}}\left(x\right)-m_{\psi }^{\prime \prime }\left(x\right)\right|⟶0$$

*in the same mode as the primitive convergence. More precisely,*

(111) $$\underset{x\in I}{\sup}\left|{\hat{m}}_{\psi ,n}^{\prime ,\mathrm{IPW}}\left(x\right)-m_{\psi }^{\prime }\left(x\right)\right|=O\left(\sum_{j=0}^{1}\Delta_{n,j}^{r}+\sum_{j=0}^{1}\Delta_{n,j}^{f}\right),$$

*and*

(112) $$\underset{x\in I}{\sup}\left|{\hat{m}}_{\psi ,n}^{\prime \prime ,\mathrm{IPW}}\left(x\right)-m_{\psi }^{\prime \prime }\left(x\right)\right|=O\left(\sum_{j=0}^{2}\Delta_{n,j}^{r}+\sum_{j=0}^{2}\Delta_{n,j}^{f}\right),$$

*where the O-symbol has the same deterministic, probabilistic, or almost-sure interpretation as the primitive bounds.*

**Corollary 4** 
(Uniform consistency of a general derivative). *Let $s\in N_{0}^{p}$ be fixed. Assume the hypotheses of Theorem 2 for every numerator and density derivative indexed by α≤s, together with (80). Then,*

(113) $$\underset{x\in I}{\sup}\left|D^{s}{\hat{m}}_{\psi ,n}^{\;\mathrm{IPW}}\left(x\right)-D^{s}m\left(\psi ;x\right)\right|⟶0$$

*in the same mode as the primitive estimators.*

When a common volume bandwidth and a common order-*ℓ* kernel are used for all primitive derivatives, the largest derivative order determines the slowest stochastic rate. Thus, under the assumptions of Theorem 2,

(114) $$\begin{array}{cc} \; & \underset{x\in I}{\sup}\left|D^{s}{\hat{m}}_{\psi ,n}^{\;\mathrm{IPW}}\left(x\right)-D^{s}m\left(\psi ;x\right)\right|\\ \; & \;=O_{a.s.}\left[{\left\{\frac{\log n}{nh_{n}^{1+2|s|/p}}\right\}}^{1/2}\right]+O\left(h_{n}^{\ell /p}\right). \end{array}$$

This conclusion uses the primitive rate at every α≤s; it is not obtained by applying a one-dimensional delta method to the ratio alone.

### 4.4. Exact Linearization and Quantitative Nonlinear Remainders

For the univariate first derivative, define

$$\Phi_{1}\left(a_{0},a_{1},b_{0},b_{1}\right)=\frac{a_{1}}{b_{0}}-\frac{a_{0}b_{1}}{b_{0}^{2}}.$$

Let

$$e_{r,j}\left(x\right)={\hat{r}}_{\psi ,n}^{(j),\mathrm{IPW}}\left(x\right)-r_{\psi }^{\left(j\right)}\left(x\right),\;e_{f,j}\left(x\right)={\hat{f}}_{X,n}^{\left(j\right)}\left(x\right)-f^{\left(j\right)}\left(x\right).$$

On $E_{n}$, Taylor’s formula yields

(115) $$\begin{array}{cc} {\hat{m}}_{\psi ,n}^{\prime ,\mathrm{IPW}}\left(x\right)-m_{\psi }^{\prime }\left(x\right)\; & =\frac{e_{r,1}\left(x\right)}{f(x)}-\frac{f^{\prime }\left(x\right)}{f^{2}\left(x\right)}e_{r,0}\left(x\right)-\frac{r_{\psi }\left(x\right)}{f^{2}\left(x\right)}e_{f,1}\left(x\right)\\ \; & \;+\left\{\frac{2r_{\psi }\left(x\right)f^{\prime }\left(x\right)}{f^{3}\left(x\right)}-\frac{r_{\psi }^{\prime }\left(x\right)}{f^{2}\left(x\right)}\right\}e_{f,0}\left(x\right)+R_{1,n}\left(x\right). \end{array}$$

There is a deterministic constant $C_{1}<\infty$ such that

(116) $$\underset{x\in I}{\sup}\left|R_{1,n}\left(x\right)\right|\leq C_{1}{\left[\max_{j=0,1}\left\{\Delta_{n,j}^{r}+\Delta_{n,j}^{f}\right\}\right]}^{2}$$

on every sufficiently small denominator-stable neighbourhood.

For the second derivative, define

$$\Phi_{2}\left(a_{0},a_{1},a_{2},b_{0},b_{1},b_{2}\right)=\frac{a_{2}}{b_{0}}-\frac{2a_{1}b_{1}}{b_{0}^{2}}+\frac{a_{0}\left(2b_{1}^{2}-b_{0}b_{2}\right)}{b_{0}^{3}}.$$

Its gradient at $\left(r_{\psi },r_{\psi }^{\prime },r_{\psi }^{\prime \prime },f,f^{\prime },f^{\prime \prime }\right)$ is

(117) $$\begin{array}{cccccc} \frac{\partial \Phi_{2}}{\partial a_{0}}\; & =\frac{2{\left(f^{\prime }\right)}^{2}-ff^{\prime \prime }}{f^{3}},\; & \frac{\partial \Phi_{2}}{\partial a_{1}}\; & =-\frac{2f^{\prime }}{f^{2}},\; & \frac{\partial \Phi_{2}}{\partial a_{2}}\; & =\frac{1}{f},\\ \frac{\partial \Phi_{2}}{\partial b_{0}}\; & =-\frac{r_{\psi }^{\prime \prime }}{f^{2}}+\frac{4r_{\psi }^{\prime }f^{\prime }}{f^{3}}-\frac{6r_{\psi }{\left(f^{\prime }\right)}^{2}}{f^{4}}+\frac{2r_{\psi }f^{\prime \prime }}{f^{3}}, & & & & \\ \frac{\partial \Phi_{2}}{\partial b_{1}}\; & =-\frac{2r_{\psi }^{\prime }}{f^{2}}+\frac{4r_{\psi }f^{\prime }}{f^{3}},\; & \frac{\partial \Phi_{2}}{\partial b_{2}}\; & =-\frac{r_{\psi }}{f^{2}}. & & \end{array}$$

Consequently,

(118) $${\hat{m}}_{\psi ,n}^{\prime \prime ,\mathrm{IPW}}\left(x\right)-m_{\psi }^{\prime \prime }\left(x\right)=\nabla \Phi_{2}{\left(x\right)}^{⊤}\left(\begin{array}{c} e_{r,0}\left(x\right)\\ e_{r,1}\left(x\right)\\ e_{r,2}\left(x\right)\\ e_{f,0}\left(x\right)\\ e_{f,1}\left(x\right)\\ e_{f,2}\left(x\right) \end{array}\right)+R_{2,n}\left(x\right),$$

where

(119) $$\underset{x\in I}{\sup}\left|R_{2,n}\left(x\right)\right|\leq C_{2}{\left[\max_{j=0,1,2}\left\{\Delta_{n,j}^{r}+\Delta_{n,j}^{f}\right\}\right]}^{2}.$$

For a general multi-index, let

$$z\left(x\right)=\left\{J_{s}r\left(x\right),J_{s}f\left(x\right)\right\},\;e_{n}\left(x\right)={\hat{z}}_{n}\left(x\right)-z\left(x\right).$$

The exact second-order Taylor formula is

(120) $$\begin{array}{cc} \; & D^{s}{\hat{m}}_{\psi ,n}^{\;\mathrm{IPW}}\left(x\right)-D^{s}m\left(\psi ;x\right)\\ \; & \;=DT_{s}\left\{z\left(x\right)\right\}\left[e_{n}\left(x\right)\right]+R_{s,n}\left(x\right), \end{array}$$

where

(121) $$R_{s,n}\left(x\right)=\int_{0}^{1}\left(1-t\right)D^{2}T_{s}\left\{z\left(x\right)+te_{n}\left(x\right)\right\}\left[e_{n}\left(x\right),e_{n}\left(x\right)\right]\;dt.$$

On a compact denominator-stable set,

(122) $$|R_{s,n}\left(x\right)|\leq \frac{H_{s}}{2}{∥e_{n}\left(x\right)∥}_{2}^{2},$$

where

$$H_{s}=\underset{z\in K_{s}}{\sup}{∥D^{2}T_{s}\left(z\right)∥}_{\mathrm{op}}<\infty .$$

**Lemma 2** 
(Sufficient rate for the nonlinear remainder). *Let*

$$N_{n,s}=nh_{n}^{1+2|s|/p}.$$

*Suppose, for every α≤s, that the pointwise primitive errors satisfy*

$${\hat{r}}_{\psi ,n}^{(\alpha ),\mathrm{IPW}}\left(x\right)-D^{\alpha }r\left(\psi ;x\right)=O_{P}\left[{\left\{nh_{n}^{1+2|\alpha |/p}\right\}}^{-1/2}+h_{n}^{\ell /p}\right],$$

*and analogously for the density derivatives. Then,*

(123) $$\begin{array}{cc} \sqrt{N_{n,s}}R_{s,n}\left(x\right)=O_{P}( & N_{n,s}^{-1/2}+h_{n}^{\ell /p}\\ \; & +\sqrt{N_{n,s}}h_{n}^{2\ell /p}). \end{array}$$

*In particular, the normalized remainder is $o_{P}\left(1\right)$ under either the undersmoothing regime or the finite-bias regime of Section 3.*

**Proof.**  The finite number of jet coordinates and (122) imply that

$$R_{s,n}\left(x\right)=O_{P}\left(a_{n,s}^{2}\right),$$

where

$$a_{n,s}=N_{n,s}^{-1/2}+h_{n}^{\ell /p},$$

because the highest derivative order has the largest stochastic error under a common bandwidth. Expanding $a_{n,s}^{2}$ and multiplying by $\sqrt{N_{n,s}}$ gives (123). If $\sqrt{N_{n,s}}h_{n}^{\ell /p}\to \lambda <\infty$, then

$$\sqrt{N_{n,s}}h_{n}^{2\ell /p}=h_{n}^{\ell /p}\left\{\sqrt{N_{n,s}}h_{n}^{\ell /p}\right\}⟶0.$$

The other two terms also converge to zero. □

### 4.5. Propagation of the Smoothing Bias

Define the order-zero primitive leading-bias fields

(124) $$\beta_{r,\ell }\left(x\right)={\left(-1\right)}^{\ell }\sum_{|\alpha |=\ell }\frac{\mu_{\alpha }\left(K\right)}{\alpha !}D^{\alpha }r\left(\psi ;x\right)$$

and

(125) $$\beta_{f,\ell }\left(x\right)={\left(-1\right)}^{\ell }\sum_{|\alpha |=\ell }\frac{\mu_{\alpha }\left(K\right)}{\alpha !}D^{\alpha }f_{X}\left(x\right).$$

The primitive bias expansion gives, for each j≤s,

$$E{\hat{r}}_{\psi ,n}^{(j),\mathrm{IPW}}-D^{j}r=h_{n}^{\ell /p}D^{j}\beta_{r,\ell }+o\left(h_{n}^{\ell /p}\right),$$

and

$$E{\hat{f}}_{n}^{\left(j\right)}-D^{j}f_{X}=h_{n}^{\ell /p}D^{j}\beta_{f,\ell }+o\left(h_{n}^{\ell /p}\right),$$

uniformly on I, provided the smoothness assumptions hold through total order |s|+ℓ.

By (93), the complete leading bias propagated through the quotient is

(126) $$B_{m,s,\ell }\left(x\right)=D^{s}\left[\frac{\beta_{r,\ell }\left(x\right)}{f_{X}\left(x\right)}-\frac{r\left(\psi ;x\right)\beta_{f,\ell }\left(x\right)}{f_{X}^{2}\left(x\right)}\right].$$

This expression contains the bias contributions of every lower primitive derivative entering the quotient identity. A formula retaining only the two primitive derivatives of total order |s| is valid only when the omitted lower-order bias combination vanishes identically.

**Proposition 7** 
(Regression-derivative expectation expansion). *Assume the primitive uniform bias expansions through order s, denominator separation, and the uniform mean-square bounds*

$$\begin{array}{cc} \; & \underset{x\in I}{\sup}E{\left|{\hat{r}}_{\psi ,n}^{(\alpha ),\mathrm{IPW}}\left(x\right)-D^{\alpha }r\left(\psi ;x\right)\right|}^{2}\\ \; & \;\leq C\left\{\frac{1}{nh_{n}^{1+2|\alpha |/p}}+h_{n}^{2\ell /p}\right\}, \end{array}$$

*with the analogous bounds for the density derivatives, uniformly over α≤s. Suppose further that*

(127) $$nh_{n}^{1+2|s|/p+\ell /p}⟶\infty ,$$

*and that the contribution of the trimming event satisfies*

(128) $$\begin{array}{cc} \; & \underset{x\in I}{\sup}E[(1+\left|D^{s}{\hat{m}}_{\psi ,n}^{\;\mathrm{IPW}}\left(x\right)\right|\\ \; & \;\;+\sum_{\alpha \leq s}(\left|{\hat{r}}_{\psi ,n}^{(\alpha ),\mathrm{IPW}}\left(x\right)-D^{\alpha }r\left(\psi ;x\right)\right|\\ \; & \;\;\;+\left|{\hat{f}}_{n}^{\left(\alpha \right)}\left(x\right)-D^{\alpha }f_{X}\left(x\right)\right|))1_{E_{n}^{c}}]=o\left(h_{n}^{\ell /p}\right). \end{array}$$

*Then,*

(129) $$\begin{array}{cc} \; & \underset{x\in I}{\sup}\left|ED^{s}{\hat{m}}_{\psi ,n}^{\;\mathrm{IPW}}\left(x\right)-D^{s}m\left(\psi ;x\right)-h_{n}^{\ell /p}B_{m,s,\ell }\left(x\right)\right|\\ \; & \;=o(h_{n}^{\ell /p}). \end{array}$$

**Proof.**  On $E_{n}$, apply (120) and take expectations. The linear term is obtained by applying $DT_{s}$ to the vector of primitive biases; (93) identifies it with $h_{n}^{\ell /p}B_{m,s,\ell }+o\left(h_{n}^{\ell /p}\right)$. By (122) and the mean-square hypotheses,

$$\underset{x\in I}{\sup}E\{|R_{s,n}\left(x\right)\left|1_{E_{n}}\right\}\leq C\left\{\frac{1}{nh_{n}^{1+2|s|/p}}+h_{n}^{2\ell /p}\right\}.$$

Condition (127) makes the first term $o(h_{n}^{\ell /p})$, and $h_{n}^{2\ell /p}=o\left(h_{n}^{\ell /p}\right)$. The complementary event is controlled by (128). Combining the three terms proves (129). □

### 4.6. Highest-Order Stochastic Reduction

At the stochastic normalization associated with |s|, every centred primitive derivative of strictly smaller total order vanishes under a common bandwidth. The same normalization does not remove its deterministic smoothing bias, which has already been incorporated into $B_{m,s,\ell }$.

**Lemma 3** 
(Highest-order reduction). *Let s≠0. Assume the joint primitive CLT conditions of Theorem 5 for all numerator and density derivatives indexed by α≤s, the primitive bias expansions, denominator separation, and the finite-bias or undersmoothing bandwidth regime. Then,*

(130) $$\begin{array}{cc} \; & \sqrt{nh_{n}^{1+2|s|/p}}\left[D^{s}{\hat{m}}_{\psi ,n}^{\;\mathrm{IPW}}\left(x\right)-D^{s}m\left(\psi ;x\right)-h_{n}^{\ell /p}B_{m,s,\ell }\left(x\right)\right]\\ \; & \;=\frac{1}{f_{X}\left(x\right)}\sqrt{nh_{n}^{1+2|s|/p}}\left[{\hat{r}}_{\psi ,n}^{(s),\mathrm{IPW}}\left(x\right)-D^{s}r\left(\psi ;x\right)-h_{n}^{\ell /p}D^{s}\beta_{r,\ell }\left(x\right)\right]\\ \; & \;-\frac{r(\psi ;x)}{f_{X}^{2}\left(x\right)}\sqrt{nh_{n}^{1+2|s|/p}}\left[{\hat{f}}_{n}^{\left(s\right)}\left(x\right)-D^{s}f_{X}\left(x\right)-h_{n}^{\ell /p}D^{s}\beta_{f,\ell }\left(x\right)\right]+o_{P}\left(1\right). \end{array}$$

*For s=0, the same representation holds without a lower-order reduction argument.*

**Proof.**  Apply (120). A primitive centred derivative of order |α| has stochastic scale ${\left\{nh_{n}^{1+2|\alpha |/p}\right\}}^{-1/2}$. Multiplication by the highest-order normalization gives

$$h_{n}^{(|s|-|\alpha |)/p},$$

which tends to zero whenever |α|<|s|. If α≤s and |α|=|s|, then necessarily α=s. The coefficient of $D^{s}r$ in the quotient derivative is $f_{X}^{-1}$. The coefficient of $D^{s}f_{X}$ is $-rf_{X}^{-2}$, as follows from differentiating $f_{X}f_{X}^{-1}=1$ and isolating the highest derivative. The bias terms combine according to (126), and Lemma 2 makes the nonlinear remainder negligible. □

### 4.7. Pointwise Gaussian Limits and Covariance Calculations

Define the conditional pseudo-response residual variance

(131) $$v_{\psi }\left(x\right)=\mathrm{Var}\left\{W_{0}\left(\psi \right)|X_{0}=x\right\}.$$

Sequential MAR gives

(132) $$v_{\psi }\left(x\right)=\frac{E\{\psi^{2}\left(Y_{0}\right)|X_{0}=x\}}{\pi (x)}-m^{2}\left(\psi ;x\right).$$

This quantity is non-negative because it is a conditional variance of the observed-data pseudo-response. Define

(133) $$\tau_{\psi ,s}^{2}\left(x\right)=\frac{R_{s}\left(K\right)}{f_{X}\left(x\right)}v_{\psi }\left(x\right),$$

or, equivalently,

(134) $$\tau_{\psi ,s}^{2}\left(x\right)=\frac{R_{s}\left(K\right)}{f_{X}\left(x\right)}\left[\frac{E\{\psi^{2}\left(Y_{0}\right)|X_{0}=x\}}{\pi (x)}-m^{2}\left(\psi ;x\right)\right].$$

The limiting covariance matrix of the top-order primitive pair is determined by the joint second-moment densities. If, in addition to the joint CLT, second moments of the normalized pair are uniformly integrable and the predictable covariance terms are negligible at the CLT scale, then

(135) $$\begin{array}{cc} \; & nh_{n}^{1+2|s|/p}\mathrm{Var}\left\{{\hat{r}}_{\psi ,n}^{(s),\mathrm{IPW}}\left(x\right)\right\}⟶f_{X}\left(x\right)\frac{E\{\psi^{2}\left(Y_{0}\right)|X_{0}=x\}}{\pi (x)}R_{s}\left(K\right),\\ \; & nh_{n}^{1+2|s|/p}\mathrm{Var}\left\{{\hat{f}}_{n}^{\left(s\right)}\left(x\right)\right\}⟶f_{X}\left(x\right)R_{s}\left(K\right),\\ \; & nh_{n}^{1+2|s|/p}\mathrm{Cov}\left\{{\hat{r}}_{\psi ,n}^{(s),\mathrm{IPW}}\left(x\right),{\hat{f}}_{n}^{\left(s\right)}\left(x\right)\right\}⟶r\left(\psi ;x\right)R_{s}\left(K\right). \end{array}$$

The last limit uses $f_{X}\left(x\right)E\left\{W_{0}\left(\psi \right)|X_{0}=x\right\}=r\left(\psi ;x\right)$.

**Theorem 7** 
(Regression-derivative central limit theorem). *Let $s\in N_{0}^{p}$ be fixed. Assume the following:*

1. *The joint primitive CLT of Theorem 5 for the order-s IPW numerator derivative and the order-s density derivative;*
2. *Pointwise primitive consistency for every lower derivative entering (99);*
3. *The primitive uniform or pointwise bias expansions needed to define $B_{m,s,\ell }$;*
4. *Denominator separation and $\tau_{n}<f_{★}/2$ for all sufficiently large n;*
5. *The primitive pointwise rates in Lemma 2.*

*If*

(136) $$\sqrt{nh_{n}^{1+2|s|/p}}h_{n}^{\ell /p}⟶\lambda \in \left[0,\infty \right),$$

*then*

(137) $$\begin{array}{cc} \; & \sqrt{nh_{n}^{1+2|s|/p}}\left[D^{s}{\hat{m}}_{\psi ,n}^{\;\mathrm{IPW}}\left(x\right)-D^{s}m\left(\psi ;x\right)\right]\\ \; & \;\overset{D}{\to }N\left(\lambda B_{m,s,\ell }\left(x\right),\tau_{\psi ,s}^{2}\left(x\right)\right). \end{array}$$

*Under the undersmoothing condition*

(138) $$\sqrt{nh_{n}^{1+2|s|/p}}h_{n}^{\ell /p}⟶0,$$

*the limit is centred:*

(139) $$\sqrt{nh_{n}^{1+2|s|/p}}\left[D^{s}{\hat{m}}_{\psi ,n}^{\;\mathrm{IPW}}\left(x\right)-D^{s}m\left(\psi ;x\right)\right]\overset{D}{\to }N\left\{0,\tau_{\psi ,s}^{2}\left(x\right)\right\}.$$

The variance calculation is exact. The limiting top-order primitive covariance matrix is

$$R_{s}\left(K\right)\left(\begin{array}{cc} f_{X}E\left(W_{0}^{2}|X_{0}=x\right) & r\\ r & f_{X} \end{array}\right),$$

and the top-order gradient is

$$\left(\begin{array}{c} f_{X}^{-1}\\ -rf_{X}^{-2} \end{array}\right).$$

The corresponding quadratic form equals

$$\frac{R_{s}\left(K\right)}{f_{X}}\left\{E\left(W_{0}^{2}|X_{0}=x\right)-m^{2}\right\},$$

which is (133). This calculation shows why dividing the primitive numerator variance by $f_{X}^{2}$ would be incorrect: the numerator–density covariance is a leading term.

For p=1 and s=k, define

$$R_{k}\left(K\right)=\int_{R}{\left\{K^{\left(k\right)}\left(u\right)\right\}}^{2}\;du.$$

Then,

(140) $$\tau_{\psi ,k}^{2}\left(x\right)=\frac{R_{k}\left(K\right)}{f_{X}\left(x\right)}\left[\frac{E\{\psi^{2}\left(Y_{0}\right)|X_{0}=x\}}{\pi (x)}-m_{\psi }^{2}\left(x\right)\right].$$

The theorem applies to (103) and (104) with k=1 and k=2.

**Remark 14** 
(Direct local martingale representation). *Let*

$$U_{i,n}\left(x\right)=\frac{W_{i}\left(\psi \right)-m\left(\psi ;x\right)}{f_{X}\left(x\right)}D^{s}K\left(\frac{x-X_{i}}{h_{n}^{1/p}}\right).$$

*Under the assumptions of Theorem 7, the leading bias-corrected stochastic term satisfies*

(141) $$\begin{array}{cc} \; & \sqrt{nh_{n}^{1+2|s|/p}}\left[D^{s}{\hat{m}}_{\psi ,n}^{\;\mathrm{IPW}}\left(x\right)-D^{s}m\left(\psi ;x\right)-h_{n}^{\ell /p}B_{m,s,\ell }\left(x\right)\right]\\ \; & \;=\frac{1}{\sqrt{nh_{n}}}\sum_{i=1}^{n}\left[U_{i,n}\left(x\right)-E\left\{U_{i,n}\left(x\right)|G_{i-1}\right\}\right]+o_{P}\left(1\right). \end{array}$$

*The predictable linear combination of the two primitive predictable components is included in the $o_{P}\left(1\right)$ term under the joint primitive CLT assumptions. The limiting conditional quadratic variation of the martingale array in* (141) *is $\tau_{\psi ,s}^{2}\left(x\right)$.*

### 4.8. Consistent Variance Estimation, Studentization, and Bias Correction

Let *L* be a bounded, compactly supported, non-negative pilot kernel of unit mass, and let $c_{n}↓0$ be a pilot volume bandwidth. Define

(142) $${\hat{f}}_{n,L}\left(x;c_{n}\right)=\frac{1}{nc_{n}}\sum_{i=1}^{n}L\left(\frac{x-X_{i}}{c_{n}^{1/p}}\right),$$

(143) $${\hat{m}}_{\psi ,n,L}^{\;\mathrm{IPW}}\left(x;c_{n}\right)=\frac{{\left(nc_{n}\right)}^{-1}\sum_{i=1}^{n}W_{i}\left(\psi \right)L\left\{\left(x-X_{i}\right)/c_{n}^{1/p}\right\}}{{\hat{f}}_{n,L}\left(x;c_{n}\right)\vee \tau_{n}},$$

and

(144) $$\begin{array}{cc} {\hat{v}}_{\psi ,n}\left(x;c_{n}\right)\; & =\frac{{\left(nc_{n}\right)}^{-1}\sum_{i=1}^{n}{\left[W_{i}\left(\psi \right)-{\hat{m}}_{\psi ,n,L}^{\;\mathrm{IPW}}\left(x;c_{n}\right)\right]}^{2}L\left\{\left(x-X_{i}\right)/c_{n}^{1/p}\right\}}{{\hat{f}}_{n,L}\left(x;c_{n}\right)\vee \tau_{n}}. \end{array}$$

Set

(145) $${\hat{\tau }}_{\psi ,s}^{2}\left(x\right)=\frac{R_{s}\left(K\right)}{{\hat{f}}_{n,L}\left(x;c_{n}\right)\vee \tau_{n}}\left\{{\hat{v}}_{\psi ,n}\left(x;c_{n}\right)\vee 0\right\}.$$

Because ${\hat{v}}_{\psi ,n}$ is a weighted average of squared residuals, it is non-negative in exact arithmetic. The positive-part operation is therefore redundant for (144); it is retained only to cover an algebraically equivalent difference-of-moments implementation subject to finite-precision error.

**Proposition 8** 
(Consistency of the regression variance estimator). *Suppose that the order-zero primitive consistency theory applies, with pilot bandwidth $c_{n}$, to the three pseudo-responses*

$$1,\;W_{i}\left(\psi \right),\;W_{i}^{2}\left(\psi \right).$$

*Assume $c_{n}\to 0$, $nc_{n}\to \infty$, continuity at x of the three corresponding local moment densities, and the conditional moment and predictable-remainder hypotheses required for those three consistency results. If $f_{X}\left(x\right)>0$ and $v_{\psi }\left(x\right)>0$, then*

(146) $${\hat{\tau }}_{\psi ,s}^{2}\left(x\right)\overset{P}{\to }\tau_{\psi ,s}^{2}\left(x\right).$$

Under (139) and (146), Slutsky’s theorem gives

(147) $$\frac{\sqrt{nh_{n}^{1+2|s|/p}}\left[D^{s}{\hat{m}}_{\psi ,n}^{\;\mathrm{IPW}}\left(x\right)-D^{s}m\left(\psi ;x\right)\right]}{{\hat{\tau }}_{\psi ,s}\left(x\right)}\overset{D}{\to }N\left(0,1\right).$$

An undersmoothed pointwise confidence interval with asymptotic coverage 1−α is

(148) $$\left[D^{s}{\hat{m}}_{\psi ,n}^{\;\mathrm{IPW}}\left(x\right)-z_{1-\alpha /2}\frac{{\hat{\tau }}_{\psi ,s}\left(x\right)}{\sqrt{nh_{n}^{1+2|s|/p}}},\;D^{s}{\hat{m}}_{\psi ,n}^{\;\mathrm{IPW}}\left(x\right)+z_{1-\alpha /2}\frac{{\hat{\tau }}_{\psi ,s}\left(x\right)}{\sqrt{nh_{n}^{1+2|s|/p}}}\right].$$

At a bandwidth for which the leading bias is not negligible, let ${\hat{B}}_{m,s,\ell }$ estimate (126). If

(149) $$\sqrt{nh_{n}^{1+2|s|/p}}h_{n}^{\ell /p}\left[{\hat{B}}_{m,s,\ell }\left(x\right)-B_{m,s,\ell }\left(x\right)\right]\overset{P}{\to }0,$$

then

(150) $$D^{s}{\hat{m}}_{\psi ,n}^{\;\mathrm{BC}}\left(x\right)=D^{s}{\hat{m}}_{\psi ,n}^{\;\mathrm{IPW}}\left(x\right)-h_{n}^{\ell /p}{\hat{B}}_{m,s,\ell }\left(x\right)$$

has the centred Gaussian limit with variance $\tau_{\psi ,s}^{2}\left(x\right)$. Condition (149) is essential: if the bias estimator has a non-negligible stochastic component, its variance and covariance with the primary estimator must be included in studentization.

**Remark 15** 
(Pointwise inference). *The interval* (148) *is pointwise in x. A simultaneous band would require a functional Gaussian approximation in a uniform norm, anti-concentration for the supremum, and a valid dependent-data critical-value procedure. None of these follows from the pointwise theorem.*

### 4.9. Asymptotic Mean Integrated Squared Error for Regression

Define

(151) $${\mathrm{MISE}}_{m,s}\left(h_{n}\right)=E\int_{I}{\left[D^{s}{\hat{m}}_{\psi ,n}^{\;\mathrm{IPW}}\left(x\right)-D^{s}m\left(\psi ;x\right)\right]}^{2}\;dx.$$

Let

(152) $$B_{m,s,\ell }=\int_{I}B_{m,s,\ell }^{2}\left(x\right)\;dx$$

and

(153) $$V_{m,\psi ,s}=R_{s}\left(K\right)\int_{I}\frac{v_{\psi }\left(x\right)}{f_{X}\left(x\right)}\;dx.$$

Under sequential MAR,

$$V_{m,\psi ,s}=R_{s}\left(K\right)\int_{I}\frac{1}{f_{X}\left(x\right)}\left[\frac{E\{\psi^{2}\left(Y_{0}\right)|X_{0}=x\}}{\pi (x)}-m^{2}\left(\psi ;x\right)\right]\;dx.$$

**Theorem 8** 
(Regression-derivative AMISE). *Assume the integrated primitive bias and joint variance expansions, the regression expectation expansion of Proposition 7, uniform integrability of the normalized squared top-order primitive pair, integrated negligibility of the predictable covariance terms, and*

$$\int_{I}E{\left|R_{s,n}\left(x\right)\right|}^{2}\;dx=o\left[h_{n}^{2\ell /p}+{\left\{nh_{n}^{1+2|s|/p}\right\}}^{-1}\right].$$

*Then,*

(154) $$\begin{array}{cc} {\mathrm{MISE}}_{m,s}\left(h_{n}\right)\; & =h_{n}^{2\ell /p}B_{m,s,\ell }+\frac{V_{m,\psi ,s}}{nh_{n}^{1+2|s|/p}}\\ \; & \;+o\left[h_{n}^{2\ell /p}+\frac{1}{nh_{n}^{1+2|s|/p}}\right]. \end{array}$$

If $0<B_{m,s,\ell }<\infty$ and $0<V_{m,\psi ,s}<\infty$, differentiation of the leading risk gives the unique minimizer

(155) $$h_{n,\mathrm{AMISE}}^{m,s}={\left\{\frac{\left(p+2|s|\right)V_{m,\psi ,s}}{2\ell B_{m,s,\ell }n}\right\}}^{p/(p+2|s|+2\ell )}.$$

Consequently,

$$h_{n,\mathrm{AMISE}}^{m,s}≍n^{-p/(p+2|s|+2\ell )},$$

and

$$\underset{h>0}{\inf}{\mathrm{AMISE}}_{m,s}\left(h\right)≍n^{-2\ell /(p+2|s|+2\ell )}.$$

At the minimizer, the exact balance identity is

$$2\ell B_{m,s,\ell }h^{2\ell /p}=\left(p+2|s|\right)\frac{V_{m,\psi ,s}}{nh^{1+2|s|/p}}.$$

At the AMISE bandwidth, the product of the Gaussian normalization and $h_{n}^{\ell /p}$ is of constant order. Consequently, when the leading bias coefficient is nonzero, an uncorrected target-centred confidence interval is not asymptotically centred.

### 4.10. Feasible Propensity-Score Replacement

Let

$${\hat{W}}_{i,n}\left(\psi \right)=\frac{\xi_{i}\psi \left(Y_{i}\right)}{{\hat{\pi }}_{n}^{†}\left(X_{i}\right)},\;\delta_{n}={∥{\hat{\pi }}_{n}^{†}-\pi ∥}_{\infty ,J}.$$

Construct the feasible numerator derivatives and regression derivative by replacing $W_{i}\left(\psi \right)$ with ${\hat{W}}_{i,n}\left(\psi \right)$; the density estimators are unchanged.

For α≤s, let $A_{n,\psi ,\alpha }$ be the local envelope in (73), with s replaced by α. On $\left\{\delta_{n}\leq \pi_{★}/2\right\}$, Proposition 5 and the local Lipschitz bound imply

(156) $$\begin{array}{cc} \; & \underset{x\in I}{\sup}\left|D^{s}{\hat{m}}_{\psi ,n}^{\;\mathrm{FIPW}}\left(x\right)-D^{s}{\hat{m}}_{\psi ,n}^{\;\mathrm{IPW}}\left(x\right)\right|\\ \; & \;\leq C_{s}\delta_{n}\sum_{\alpha \leq s}h_{n}^{-|\alpha |/p}A_{n,\psi ,\alpha }. \end{array}$$

If the finitely many envelopes are $O_{P}\left(1\right)$ and $h_{n}<1$, then

$$\underset{x\in I}{\sup}\left|D^{s}{\hat{m}}_{\psi ,n}^{\;\mathrm{FIPW}}-D^{s}{\hat{m}}_{\psi ,n}^{\;\mathrm{IPW}}\right|=O_{P}\left\{\delta_{n}h_{n}^{-|s|/p}\right\}.$$

A conservative sufficient condition for oracle-equivalent pointwise inference is therefore

(157) $$\sqrt{nh_{n}}\;\delta_{n}⟶0\;\mathrm{in}\;\mathrm{probability}.$$

Indeed, multiplication by the top-order normalization gives

$$\sqrt{nh_{n}^{1+2|s|/p}}\delta_{n}h_{n}^{-|s|/p}=\sqrt{nh_{n}}\;\delta_{n}.$$

For oracle-equivalent uniform consistency, it is sufficient that $\delta_{n}h_{n}^{-|s|/p}\to 0$ in probability; preservation of the oracle uniform rate requires the stronger comparison with the right-hand side of (114). These are sufficient, not necessary, conditions. Cross-fitting does not by itself remove the first-order propensity error; slower nuisance rates require an explicitly orthogonal or augmented estimating equation and a separate dependent-data analysis.

### 4.11. Important Special Cases

#### 4.11.1. Conditional Mean Regression

For a scalar response and ψ(y)=y, $m\left(\psi ;x\right)=E\left(Y_{0}|X_{0}=x\right)$. The asymptotic variance is

(158) $$\tau_{Y,s}^{2}\left(x\right)=\frac{R_{s}\left(K\right)}{f_{X}\left(x\right)}\left[\frac{E(Y_{0}^{2}|X_{0}=x)}{\pi (x)}-m^{2}\left(x\right)\right].$$

If $\sigma_{Y}^{2}\left(x\right)=\mathrm{Var}\left(Y_{0}|X_{0}=x\right)$, then

(159) $$\frac{E(Y_{0}^{2}|X_{0}=x)}{\pi (x)}-m^{2}\left(x\right)=\frac{\sigma_{Y}^{2}\left(x\right)}{\pi (x)}+\left\{\frac{1}{\pi (x)}-1\right\}m^{2}\left(x\right).$$

The second term is the additional Bernoulli-observation component induced by inverse probability weighting.

#### 4.11.2. Conditional Moments

For $\psi \left(y\right)=y^{a}$, a≥1, the target is $m_{a}\left(x\right)=E\left(Y_{0}^{a}|X_{0}=x\right)$. Its local pseudo-response variance contains

(160) $$\frac{E(Y_{0}^{2a}|X_{0}=x)}{\pi (x)}-m_{a}^{2}\left(x\right).$$

A (2+η)-moment Lindeberg argument for the pseudo-response $W_{i}\left(y^{a}\right)$ requires a conditional moment of order a(2+η) for $|Y_{i}|$, together with positivity and the other conditional assumptions of Section 3.

#### 4.11.3. Conditional Distribution Functions

For $\psi_{t}\left(y\right)=1\left\{y\leq t\right\}$, the target is $F_{Y|X}\left(t|x\right)$. For fixed t,

(161) $$v_{t}\left(x\right)=\frac{F_{Y|X}\left(t|x\right)}{\pi (x)}-F_{Y|X}^{2}\left(t|x\right).$$

In the univariate case, the order-zero estimator is

(162) $${\hat{F}}_{n}^{\;\mathrm{IPW}}\left(t|x\right)=\frac{\sum_{i=1}^{n}\frac{\xi_{i}1\left\{Y_{i}\leq t\right\}}{\pi (X_{i})}K\left\{\left(x-X_{i}\right)/h_{n}\right\}}{\sum_{i=1}^{n}K\left\{\left(x-X_{i}\right)/h_{n}\right\}}$$

whenever the denominator is positive, or with the stabilized denominator used above. Covariate derivatives are obtained from the same reciprocal recursion. Uniformity simultaneously over x and t is not claimed: it requires entropy and stochastic equicontinuity for the product of the kernel-translation class and the lower-orthant indicator class.

#### 4.11.4. Complete Responses

If π≡1, then $W_{i}\left(\psi \right)=\psi \left(Y_{i}\right)$ and

$$v_{\psi }\left(x\right)=\mathrm{Var}\left\{\psi \left(Y_{0}\right)|X_{0}=x\right\}.$$

Thus, (133) reduces to the usual complete-response kernel regression-derivative variance.

### 4.12. Alternative Denominators and Complete-Case Ratios

The unweighted complete-case ratio

(163) $$\frac{\sum_{i=1}^{n}\xi_{i}\psi \left(Y_{i}\right)K\left\{\left(x-X_{i}\right)/h_{n}^{1/p}\right\}}{\sum_{i=1}^{n}\xi_{i}K\left\{\left(x-X_{i}\right)/h_{n}^{1/p}\right\}}$$

identifies m(ψ;x) at order zero under MAR because its population numerator and denominator are proportional to π(x)r(ψ;x) and $\pi \left(x\right)f_{X}\left(x\right)$, respectively. Hence, it would be incorrect to claim that every complete-case ratio is inconsistent for the conditional mean under MAR.

The derivative analysis is nevertheless different. The primitive functions for the complete-case ratio are πr and $\pi f_{X}$, so their kernel biases involve derivatives of π, and their joint variance differs from the IPW/full-covariate construction. Although the population quotient simplifies to *m*, the cancellation of propensity derivatives occurs only after the complete quotient differentiation and does not make the primitive bias or covariance calculations identical.

An IPW-weighted denominator,

$$\sum_{i=1}^{n}\frac{\xi_{i}}{\pi (X_{i})}K\left\{\left(x-X_{i}\right)/h_{n}^{1/p}\right\},$$

also targets the marginal design density. It replaces a fully observed local covariate mass with an unbiased but noisier per-observation surrogate. However, for the final ratio, the weighted numerator and weighted denominator share the same random weight, so their covariance can offset part of the marginal variance increase. Without additional assumptions, there is no universal finite-sample variance ordering between the two ratio constructions.

The estimator studied in this paper was selected because its primitive objects match the identification decomposition

$$r\left(\psi ;x\right)=E\left\{W_{0}\left(\psi \right)|X_{0}=x\right\}f_{X}\left(x\right),$$

and because it estimates the fully observed design density directly. All bias, covariance, and inferential statements in this paper refer specifically to this construction.

## 5. Monte Carlo Experiments

### 5.1. Objectives and Scope

The purpose of this section is to examine, under controlled finite-sample experiments, the numerical implications of the asymptotic theory developed in Section 2, Section 3 and Section 4. The analysis is organized around three distinct levels of the estimation problem: The first concerns the primitive inverse-probability-weighted kernel derivative estimator $D^{s}r_{n}^{\mathrm{IPW}}\left(\psi ;x,h_{n}\right)$ together with the unweighted marginal-density derivative estimator $D^{s}f_{X;n}\left(x,h_{n}\right)$. The second concerns the nonlinear propagation of these primitive errors through the quotient map leading to ${\hat{m}}_{\psi ,n}^{\mathrm{IPW}}$ and its first- and second-order derivatives. The third concerns studentization, bias, bandwidth choice, propensity-score replacement, and the extent to which the asymptotic linear representations are informative at the sample sizes considered.

The numerical design is deliberately aligned with the decomposition underlying the theory. In particular, the Fréchet linearization of ${\hat{m}}_{\psi ,n}^{\prime ,\mathrm{IPW}}$ is evaluated through its exact remainder, the variance calculation retains the full covariance matrix of the primitive numerator and density terms, and the comparison between weighted and unweighted denominator constructions is interpreted through their joint covariance structure rather than through marginal variances alone. Thus, the Monte Carlo analysis targets the mechanisms that enter the proofs, rather than only reporting aggregate MISE values.

The numerical scope is intentionally finite: n∈{250,500,1000,2000}, studentized inference for regression derivatives is carried out for the first derivative, and no pilot-based leading-bias correction is implemented. These restrictions delimit the numerical conclusions and are not used to extrapolate beyond the experiments actually performed.

A strict distinction is maintained throughout between theorem-level statements and simulation-level evidence. A Monte Carlo experiment can reveal pre-asymptotic behaviour, diagnose a proposed studentization, or exhibit a finite-sample mechanism compatible with an asymptotic expansion; it cannot, by itself, establish projective conditions, conditional-density regularity, conditional quadratic-variation stabilization, or any other probabilistic hypothesis appearing in the limit theory. Accordingly, numerical agreement with a theorem is described only as compatibility with its predicted mechanism, whereas designs falling outside a theorem’s hypotheses are treated explicitly as robustness or stress-test experiments.

### 5.2. Data-Generating Mechanisms

#### 5.2.1. Covariate Processes and Dependence

**Figure 1:**
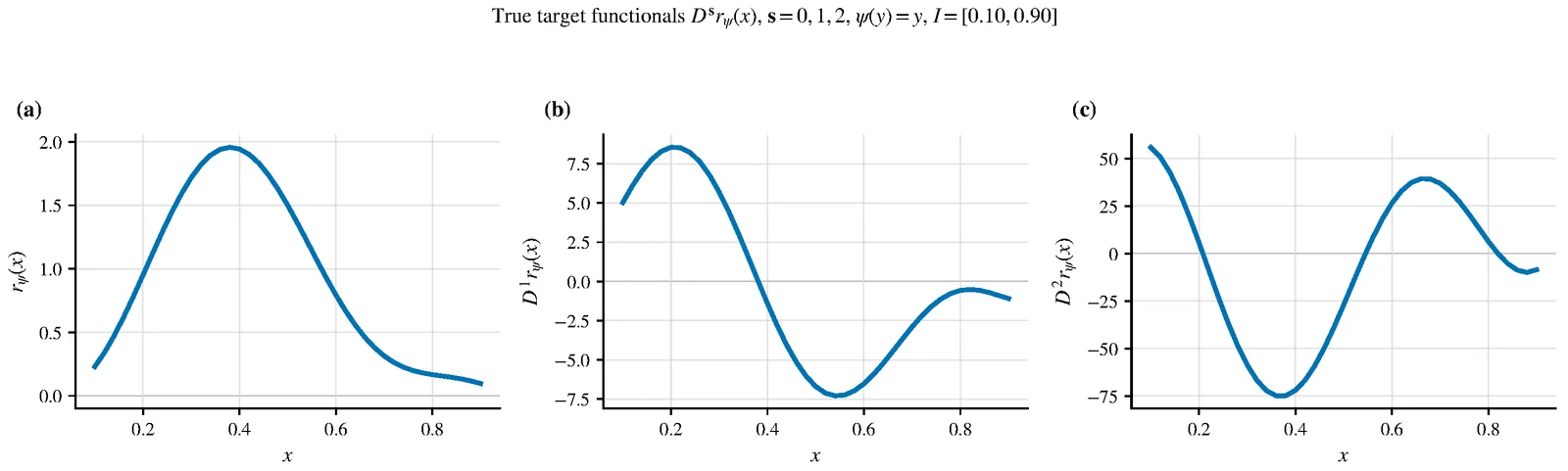
True target functionals $D^{s}r_{\psi }\left(x\right)$, |s|=0,1,2, ψ(y)=y, evaluation domain I=[0.10,0.90].

**Figure 2:**
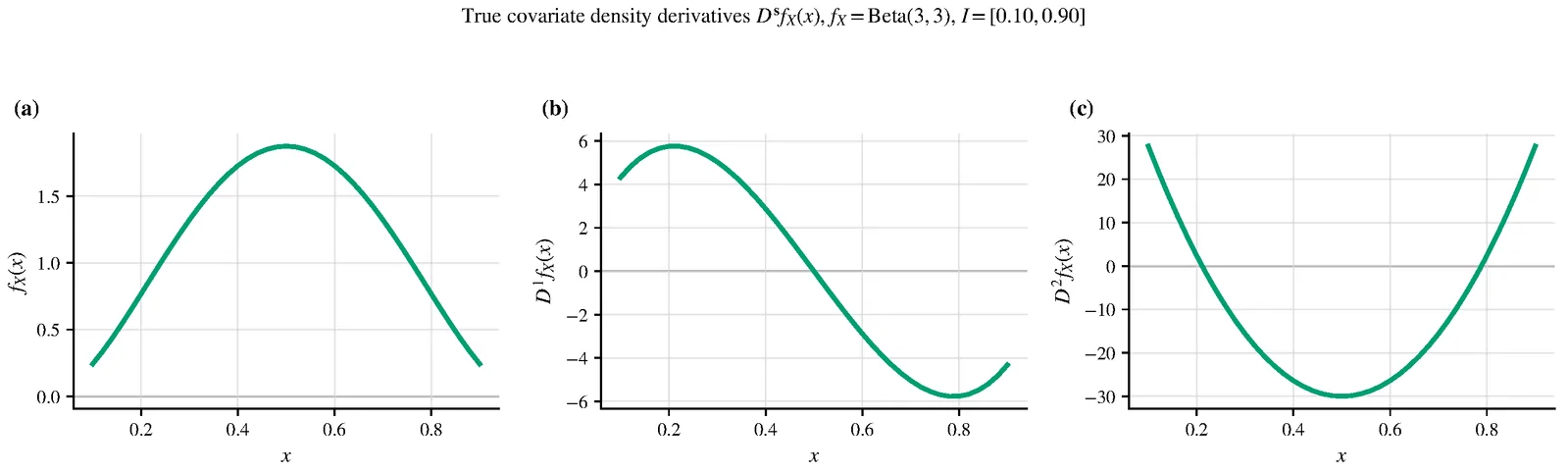
True covariate density derivatives $D^{s}f_{X}\left(x\right)$, $f_{X}=\mathrm{Beta}\left(3,3\right)$, evaluation domain I=[0.10,0.90].

The marginal covariate distribution is fixed at $f_{X}=\mathrm{Beta}\left(3,3\right)$ on [0,1], and all reported integrated criteria are computed on the interior set I=[0.10,0.90]⊂J=[0,1]; see Figure 1 and Figure 2. Dependence is introduced through the probability integral transform $X_{i}=F_{X}^{-1}\left(U_{i}\right)$. The configurations are as follows: (D1) i.i.d. $U_{i}∼\mathrm{Unif}\left(0,1\right)$; (D2) a stationary Gaussian AR(1) latent process

$$Z_{i}=\rho Z_{i-1}+\sqrt{1-\rho^{2}}\eta_{i},$$

initialized from its invariant N(0,1) law, with ρ∈{0.3,0.7,0.9} and $U_{i}=\Phi \left(Z_{i}\right)$; and (D4) the irrational rotation $U_{i}=\left(U_{0}+i\alpha \right)\;\mathrm{mod}\;1$, where $\alpha =(\sqrt{5}-1)/2$ and $U_{0}∼\mathrm{Unif}\left(0,1\right)$.

The rotation is measure-preserving and ergodic with respect to Lebesgue measure and is not mixing; the measurable transformation $F_{X}^{-1}$ preserves stationarity and ergodicity. Its role in the numerical study must, however, be stated precisely. Relative to a filtration containing the previous covariate, the next rotation state is determined by the past, so the one-step conditional law is degenerate. Consequently, whenever the main theorems require a Lebesgue conditional density of $X_{i}$ given $G_{i-1}$, D4 lies outside that hypothesis. It is therefore used here as an ergodic non-mixing *stress test*, not as a theorem-verifying example. For D2, the additional predictable-component and conditional-variance assumptions required by the main results are likewise not inferred from the Monte Carlo output; their verification is an analytic question distinct from finite-sample performance.

#### 5.2.2. Regression and Response Models

$$m_{\psi }\left(x\right)=0.5+x-0.8x^{2}+0.6\sin\left(2\pi x\right),$$

and

$$r_{\psi }\left(x\right)=m_{\psi }\left(x\right)f_{X}\left(x\right).$$

Figure 1 displays $r_{\psi }$ and its first two derivatives, which are the primitive targets for |s|=0,1,2. Three conditional response laws are considered: The first is homoscedastic Gaussian, $Y_{i}=m_{\psi }\left(X_{i}\right)+0.5\varepsilon_{i}$ with $\varepsilon_{i}∼N\left(0,1\right)$ and $\mathrm{Var}(Y_{i}|X_{i})=0.25$. The second is heteroscedastic Gaussian with conditional scale σ(x)=0.3+0.4x and, hence, conditional variance ranging from 0.09 to 0.49 on [0,1]. The third uses a standardized Student $t_{6}$ innovation rescaled to variance 0.25. The latter possesses moments of order strictly smaller than 6 and, therefore, satisfies the conditional (2+η) moment requirement used for pointwise Lindeberg arguments for every η<4 under the present bounded-overlap design. It should not be confused with the stronger conditional Bernstein condition used for exponential uniform bounds, which a polynomial-tailed $t_{6}$ law does not satisfy without an additional truncation argument.

For all three response specifications, $\Psi_{2}^{\mathrm{IPW}}\left(x,\psi \right)=E\left\{Y^{2}|X=x\right\}/\pi \left(x\right)$ is available analytically from the data-generating mechanism and is used whenever a population variance benchmark is required.

#### 5.2.3. Missing-at-Random Mechanisms

Six observation mechanisms are considered: M0 (π≡1), M1a and M1b (MCAR with $\pi_{0}\in \left\{0.8,0.6\right\}$), M2 (moderate smooth logistic MAR), M3 (severe heterogeneous MAR), and M4 (smooth non-monotone MAR with $\pi \left(x\right)=0.30+0.55\sin^{2}\left(\pi x\right)$). The observation indicator is generated from the stated propensity mechanism, so the missingness structure is imposed by construction; positivity over the evaluation region is checked directly from the implemented propensity functions. Table 1 reports the resulting local propensity ranges, the mean response probability under $f_{X}$, and the realized response rate for the n=1000, N=500 experiment used in Section 5.12.

These mechanisms are intentionally MAR. The severe heterogeneous and non-monotone designs serve as weak-overlap stress tests while preserving identification. We do not label any design as MNAR: under genuine MNAR, the identity defining the proposed IPW target generally fails, and a principled analysis would require an additional selection or sensitivity model. Likewise, a design with π(x)=0 on the evaluation region would illustrate non-identification rather than robustness of the present estimator. The simulation evidence should therefore be read as robustness within identified MAR regimes, not as evidence of validity under MNAR.

The leading pointwise variance is intrinsically local through

$$\sigma_{\psi ,\mathrm{IPW}}^{2}\left(x\right)=f_{X}\left(x\right)\Psi_{2}^{\mathrm{IPW}}\left(x,\psi \right)R_{s}\left(K\right).$$

**Figure 3:**
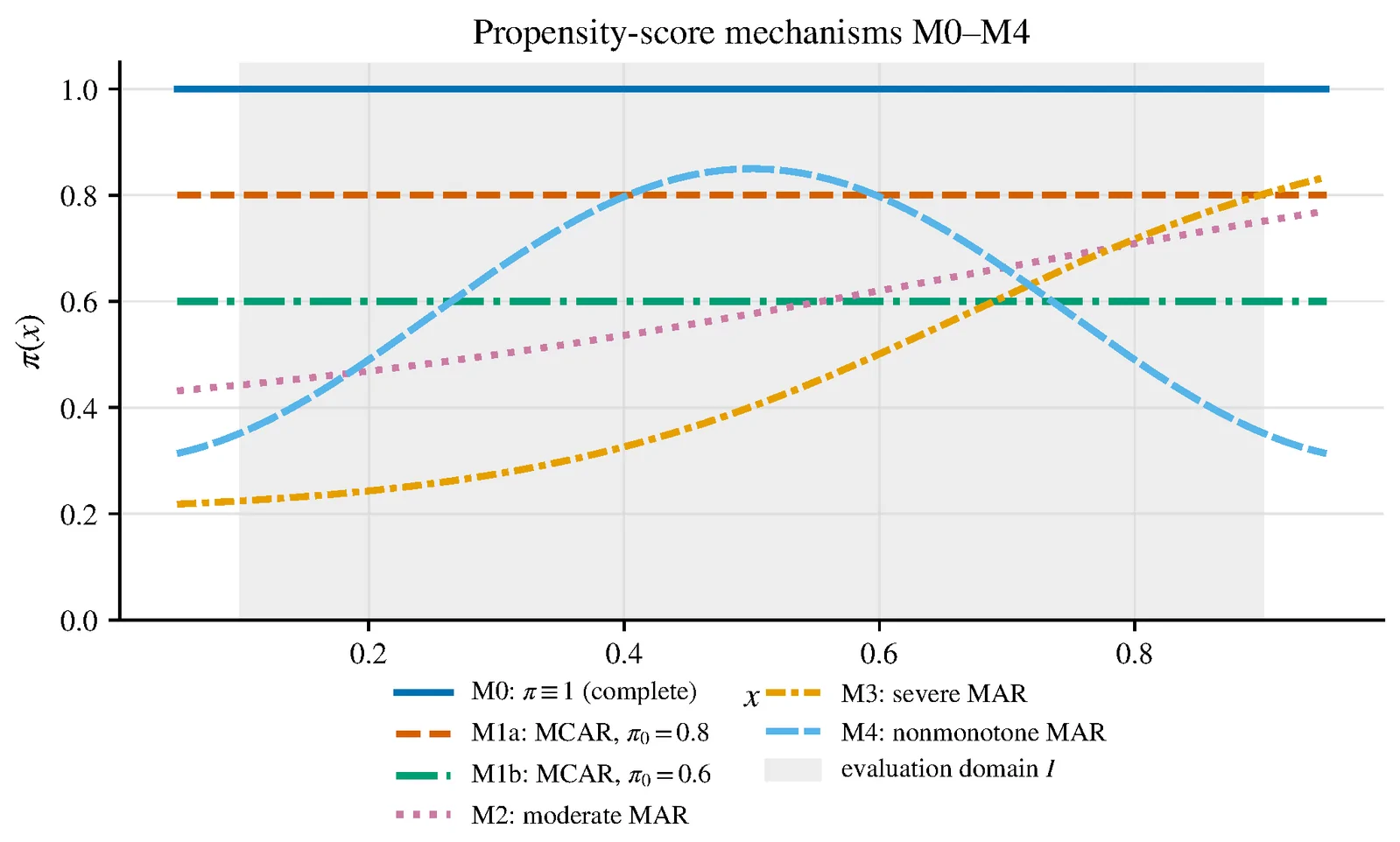
Propensity-score mechanisms M0–M4 over J=[0,1]; shaded band marks I=[0.10,0.90].

We therefore retain three fixed interior locations under M2: x=0.15, 0.50, and 0.85, with propensities 0.455, 0.585, and 0.751, respectively, see Figure 3. These locations do not isolate propensity variation alone: because the Beta(3,3) density is symmetric, $f_{X}\left(0.15\right)=f_{X}\left(0.85\right)=0.488$, whereas $f_{X}\left(0.50\right)=1.875$. Hence, the flanking points simultaneously represent lower design density. This confounding is material for nonlinear regression-derivative inference because the Fréchet coefficients contain inverse powers of $f_{X}$; all pointwise comparisons below are interpreted with this local conditioning in view.

### 5.3. Estimators and Competing Procedures

The primitive and ratio estimators are compared under six constructions: E1 is the oracle IPW estimator using the true propensity π. E2 replaces π with the fitted propensity ${\hat{\pi }}_{n}$ from the correctly specified logistic model $\mathrm{logit}\pi \left(x\right)=\beta_{0}+\beta_{1}x$, estimated by Newton–Raphson IRLS. E3 deliberately misspecifies the nuisance model by using an intercept-only logistic fit. E4 omits the inverse-probability correction in the response-dependent numerator while retaining the fully observed covariate denominator; at the primitive level, this changes the target from $r_{\psi }\left(x\right)$ to $\pi \left(x\right)r_{\psi }\left(x\right)$ whenever π is nonconstant. E5 is the infeasible full-response benchmark corresponding to M0.

For ratio estimators, we additionally compare the construction analysed in the paper, denoted ${\text{E1}}_{\mathrm{NW}}$, whose marginal-density denominator uses all observed covariates without response weighting, with E6, which replaces that denominator with an IPW version. Under correct weighting, both denominator constructions can target $f_{X}$; their stochastic structures are nevertheless different because E6 introduces the same Bernoulli/IPW factor into the numerator and denominator. Consequently, no marginal-variance argument alone determines the variance ordering of the resulting ratio estimators; this issue is examined through their empirical covariance structure in Section 5.14.

Thus, the numerical comparison explicitly includes the full-response benchmark (E5), the uncorrected/naive complete-case construction (E4), correctly specified and misspecified feasible IPW estimators (E2–E3), and an alternative IPW denominator (E6). A genuinely doubly robust estimator is not added here, because it would require specifying and estimating an additional outcome model and developing the corresponding orthogonal derivative-functional theory; this is a distinct extension rather than a drop-in comparator for the present estimator class.

**Figure 4:**
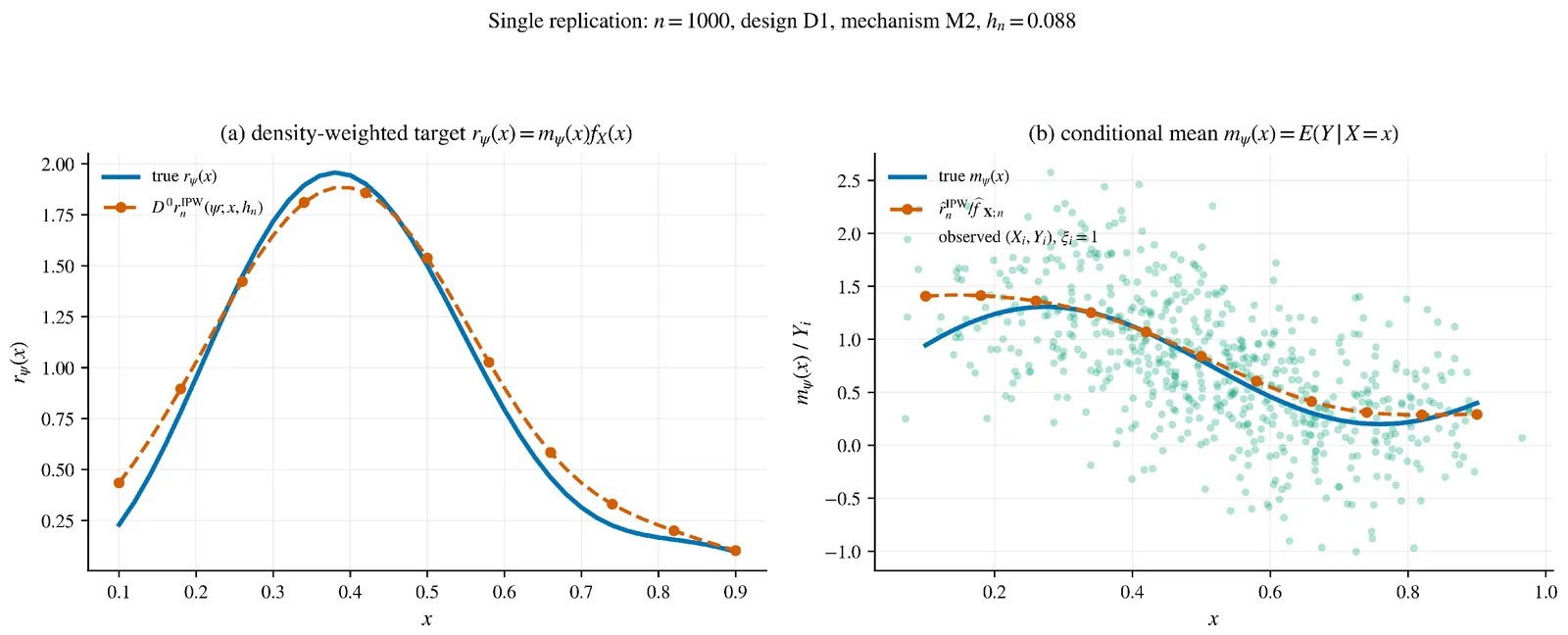
Single Monte Carlo replication, n=1000, design D1, mechanism M2, $h_{n}=0.088$. Panel (**a**): $r_{\psi }\left(x\right)$, the density-weighted estimand of $D^{0}r_{n}^{\mathrm{IPW}}$. Panel (**b**): the derived ratio estimator of $m_{\psi }\left(x\right)=E\left(Y|X=x\right)$, shown with observed pairs $\left(X_{i},Y_{i}\right)$, $\xi_{i}=1$.

The regression derivatives themselves are never obtained by numerical differentiation. The primitive estimators ${\hat{r}}_{\psi ,n}^{(j),\mathrm{IPW}}$ and ${\hat{f}}_{X,n}^{\left(j\right)}$, j=0,1,2, are computed separately and substituted into the exact quotient identities (103) and (104), using the bandwidth convention of Equation (101) and plotted in Figure 4.

### 5.4. Bandwidth Rules

Four bandwidth prescriptions are used. Since the simulations are one-dimensional (p=1) with kernel order ℓ=2, B1 adopts the AMISE-rate form $h_{n}=c\;n^{-1/(1+2|s|+2\ell )}$ with c=0.35. B2 is an infeasible oracle benchmark: for each replication, the bandwidth is chosen from a 25-point geometric grid by minimizing the exact integrated squared error with respect to the known target. B3 is a covariate-ordered leave-one-block-out criterion based on contiguous blocks of length 20 after sorting by *X*, and it minimizes $\sum_{i}{\left\{W_{i}\left(\psi \right)-{\hat{r}}_{-\mathrm{block}(i)}\left(X_{i}\right)\right\}}^{2}$. Thus, B3 is a prediction-error rule for the raw IPW pseudo-response; it is neither an ISE-targeted selector nor, after sorting by the covariate, a time-series block cross-validation scheme.

**Figure 5:**
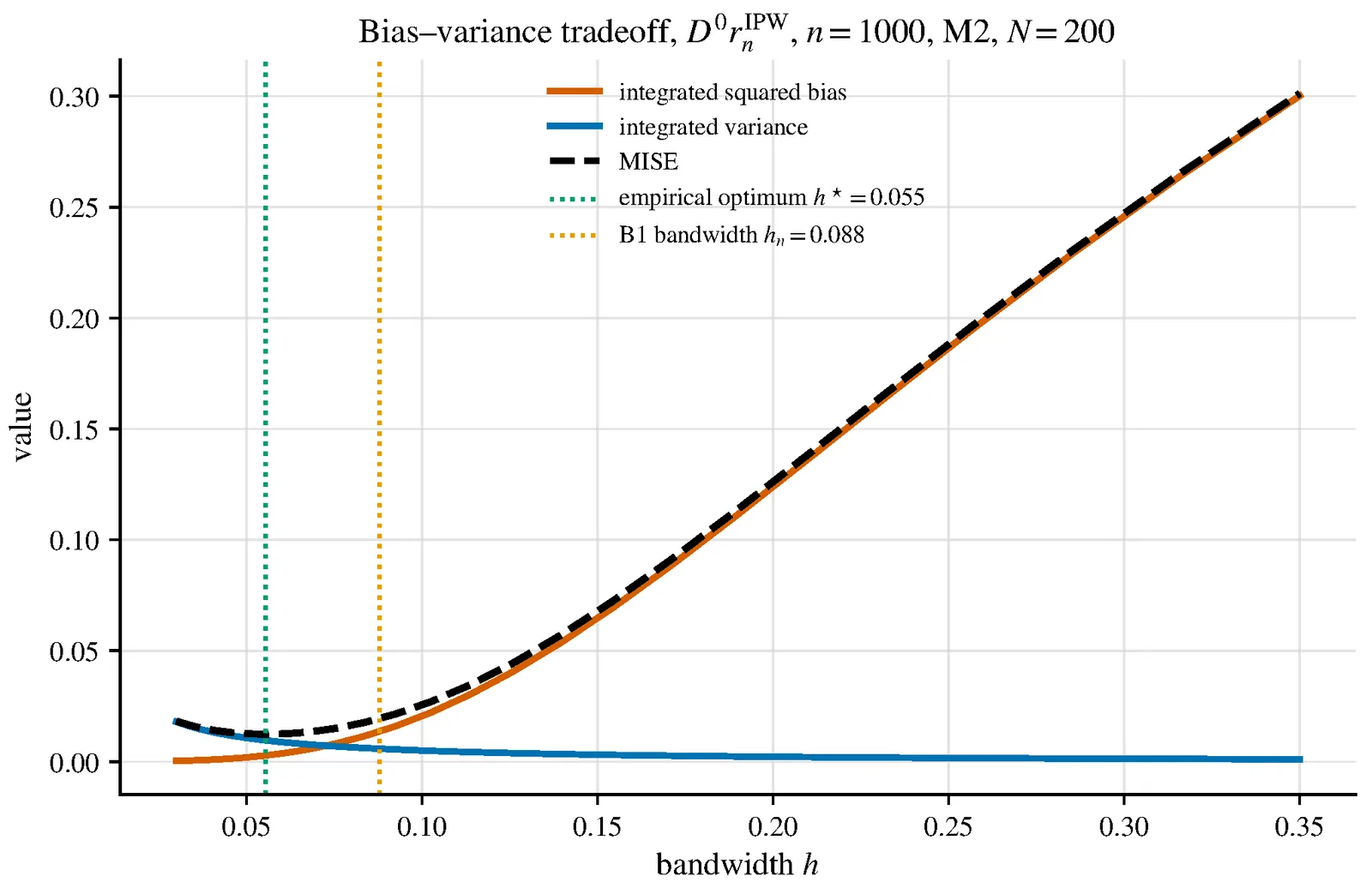
Empirical bias–variance decomposition of $D^{0}r_{n}^{\mathrm{IPW}}\left(\psi ;\cdot ,h\right)$ vs. bandwidth *h*, design D1, mechanism M2, n=1000, N=200, integrated over *I*.

B4 imposes additional undersmoothing, $h_{n}=c\;n^{-[1/(1+2|s|+2\ell )+\delta ]}$ with δ=0.05. At the level of exponents, this construction is designed to satisfy the asymptotic bias-negligibility requirement more strongly than B1. This exponent comparison is only an asymptotic statement: it does not imply that the normalized bias is numerically negligible for the available sample sizes. Section 5.17 and Section 5.18 therefore evaluate B4 empirically rather than assigning it, a priori, the status of a finite-sample inference bandwidth, see Figure 5.

### 5.5. Performance Criteria and Monte Carlo Uncertainty

For each configuration, based on the replication count *N* reported in the corresponding table, we record pointwise bias and standard deviation, RMSE, integrated squared bias, integrated variance, MISE, and the sup-norm error on a fixed 41-point grid over *I*. Monte Carlo uncertainty is quantified rather than suppressed: for MISE, we use $\hat{\mathrm{se}}\left(\hat{\mathrm{MISE}}\right)=\mathrm{sd}\left({\mathrm{ISE}}_{1},\ldots ,{\mathrm{ISE}}_{N}\right)/\sqrt{N}$, and for a coverage estimate $\hat{p}$ we report the binomial Monte Carlo standard error ${\left\{\hat{p}\left(1-\hat{p}\right)/N\right\}}^{1/2}$. Numerical differences smaller than approximately two relevant Monte Carlo standard errors are not interpreted as systematic design effects.

To make the finite-sample efficiency comparison explicit, whenever two procedures *A* and *B* target the same functional, we use the relative MISE efficiency

$$\mathrm{RE}\left(A:B\right)=\frac{\hat{\mathrm{MISE}}\left(B\right)}{\hat{\mathrm{MISE}}\left(A\right)}.$$

Thus, RE(A:B)>1 favours procedure *A*, whereas a value below one favours *B*. MISE is the primary quantitative efficacy criterion because it combines integrated squared bias and integrated variance; pointwise RMSE, sup-norm error, and coverage are reported separately because a method can have favourable MISE yet still be unsuitable for target-centred inference if its smoothing bias is large on the studentized scale.

The replication counts are chosen so that Monte Carlo noise is small relative to the large design effects emphasized below. For a coverage proportion, the worst-case binomial Monte Carlo standard error is bounded by $1/(2\sqrt{N})$, which equals approximately 0.0158, 0.0224, and 0.0354 for N=1000, 500, and 200, respectively. For MISE, the replication-specific ISE standard deviation is used in the formula above (and, where displayed graphically, the resulting Monte Carlo uncertainty is shown by error bars). We therefore avoid interpreting differences that are of the same order as these simulation errors.

### 5.6. Primitive Density-Weighted Derivative Estimation

For each derivative order, both bias and MISE decrease over the simulated sample-size sequence. Under D1, this behaviour is compatible with the consistency theory, but the table is not used as an empirical proof of an asymptotic rate. The finite-sample difficulty increases sharply with |s|: at |s|=2, the estimator remains strongly bias-dominated even at n=2000, so the experiment is plainly pre-asymptotic at that derivative order for the constant c=0.35. This feature is central when interpreting the empirical slopes in Section 5.20 and the studentized regression-derivative results below in Table 2.

The restriction is therefore a finite-sample one for the present bandwidth and sample range, not a new restriction on the consistency theorem. In particular, the order-two estimates in this experiment should not be used for precise target-centred inference at n≤2000 with c=0.35. The bias can be reduced by using a smaller derivative-specific bandwidth (or stronger undersmoothing when inference is the goal), by explicit analytical or robust bias correction, by higher-order/local-polynomial derivative smoothing when the required smoothness is credible, or simply by increasing the sample size. These remedies are not cost-free: more aggressive undersmoothing increases variance, while higher-order bias correction requires additional smoothness and estimation of further derivatives. The practical objective is therefore to verify that the estimated bias is small relative to the stochastic standard error, rather than to assume that a single bandwidth constant is adequate for every derivative order.

The marginal-density derivative estimator $D^{s}f_{X;n}$ exhibits errors of comparable order. Because its construction uses the complete covariate sample and contains neither $\xi_{i}$ nor $\pi (X_{i})$, its numerical output is invariant, up to Monte Carlo noise, to the response-missingness mechanism when the covariate path and bandwidth are held fixed. This is an implementation consequence of the estimator definition rather than an empirical identification argument.

### 5.7. Regression-Derivative Estimation

The plug-in regression derivatives exhibit the same pronounced bias domination as their primitive ingredients, with a further amplification induced by the nonlinear quotient map. In the first derivative, the linearized error depends on the order-zero and order-one numerator and density errors; in the second derivative, it additionally involves the order-two primitive terms. Because the associated coefficients contain inverse powers of $f_{X}$, the local design density also controls the conditioning of this transformation. The next subsection evaluates this linearization directly rather than inferring its quality from the aggregate MISE values, see Table 3.

### 5.8. Accuracy of the Fréchet Linearization

**Figure 6:**
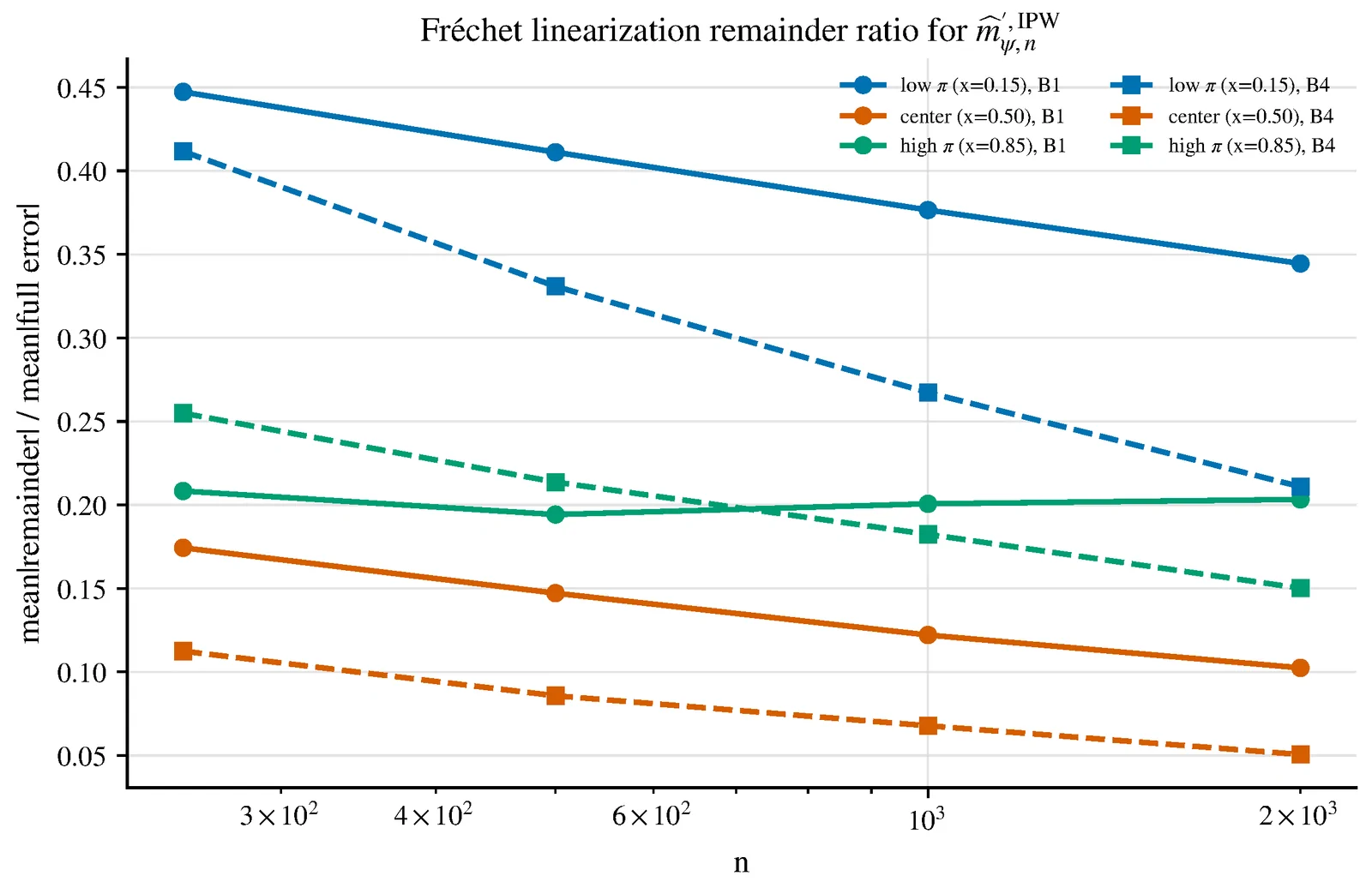
Ratio of mean absolute linearization remainder to mean absolute full estimation error for ${\hat{m}}_{\psi ,n}^{\prime ,\mathrm{IPW}}$, design D1, mechanism M2, N=1000, bandwidth rules B1 and B4. The plotted symbols are the four Monte Carlo summaries reported in Table 4; adjacent symbols are joined only by straight line segments as visual guides, with no fitted or smoothed curve.

For ${\hat{m}}_{\psi ,n}^{\prime ,\mathrm{IPW}}$, consider the first-order Fréchet expansion of the quotient functional at the population vector $\left(r_{\psi },r_{\psi }^{\prime },f_{X},f_{X}^{\prime }\right)$:

$$\begin{array}{c} {\hat{m}}_{\psi ,n}^{\prime ,\mathrm{IPW}}\left(x\right)-m_{\psi }^{\prime }\left(x\right)\\ \;=a\left(x\right)\left\{{\hat{r}}^{\prime }\left(x\right)-r_{\psi }^{\prime }\left(x\right)\right\}+b\left(x\right)\left\{\hat{r}\left(x\right)-r_{\psi }\left(x\right)\right\}\\ \;\;+c\left(x\right)\left\{{\hat{f}}^{\prime }\left(x\right)-f_{X}^{\prime }\left(x\right)\right\}+d\left(x\right)\left\{\hat{f}\left(x\right)-f_{X}\left(x\right)\right\}+R_{1,n}\left(x\right), \end{array}$$

where $a=1/f_{X}$, $b=-f_{X}^{\prime }/f_{X}^{2}$, $c=-r_{\psi }/f_{X}^{2}$, and $d=2r_{\psi }f_{X}^{\prime }/f_{X}^{3}-r_{\psi }^{\prime }/f_{X}^{2}$. These coefficients are precisely the differential of the quotient map underlying (81) and were verified symbolically before numerical use. For each D1/M2 replication and for both B1 and B4 at derivative order one, we compute the exact estimation error, the corresponding four-term linear contribution, and the residual $R_{1,n}\left(x\right)$ at the three locations in Table 1. The reported ratio is therefore an exact finite-sample diagnostic of the nonlinear remainder for the implemented estimator, see Figure 6 and Table 4.

At the central design point, the relative remainder decreases monotonically with *n* under both bandwidth rules, from 0.174 to 0.102 under B1 and from 0.112 to 0.051 under B4. This is compatible with increasing accuracy of the first-order linearization, although four sample sizes do not identify a reliable asymptotic exponent. The behaviour is materially less favourable at x=0.15. There, the relevant Fréchet map is substantially less well conditioned: $f_{X}\left(0.15\right)/f_{X}\left(0.50\right)≃0.260$, so coefficients containing $f_{X}^{-2}$ and $f_{X}^{-3}$ are amplified by factors of approximately 15 and 57, respectively, relative to the centre. This deterministic amplification provides a natural mechanism for the larger linear and nonlinear errors at the flanking point, but it does not by itself identify the complete source of the finite-sample remainder. We therefore interpret the low-density discrepancy as a local pre-asymptotic conditioning phenomenon rather than as evidence against the differentiability argument.

The slight non-monotonicity of the B1 curve at the point labelled “high π” (x=0.85: 0.208,0.194,0.201,0.203) should not be read as a separate asymptotic phenomenon. The label refers to a high *response propensity*, not to a high covariate design density. Because the simulation uses the symmetric Beta(3,3) design, $f_{X}\left(0.85\right)/f_{X}\left(0.50\right)≃0.260$, the same low-density conditioning that affects x=0.15 also amplifies the quotient-map coefficients at x=0.85. Moreover, the plotted ordinate is a ratio of two finite-sample Monte Carlo averages—mean absolute remainder divided by mean absolute full error—and neither component is required to decrease at the same rate with *n*. A small reversal can therefore arise from the changing bias–variance balance and Monte Carlo variation. No monotonicity claim is attached to this curve.

### 5.9. Highest-Order Stochastic Reduction for Regression

The asymptotic theory for regression derivatives ultimately singles out the highest derivative-order primitive components after normalization. The present Monte Carlo diagnostics do not attempt to estimate a separate normalized contribution vector for $\left\{{\hat{r}}^{\prime }-r_{\psi }^{\prime },\hat{r}-r_{\psi },{\hat{f}}^{\prime }-f_{X}^{\prime },\hat{f}-f_{X}\right\}$ while simultaneously removing their deterministic smoothing biases. Instead, Table 4 assesses the aggregate first-order representation, and Table 5 resolves the repeated-sampling covariance structure of the primitive terms. These calculations are sufficient to study linearization and variance propagation, but they should not be interpreted as a numerical verification of the separate highest-order stochastic reduction. That stronger diagnostic would require bias-separated, normalization-specific component calculations.

### 5.10. Denominator Stability and Trimming

To prevent numerical instability of the ratio map when the estimated design density is very small, the implementation follows the convention stated after Equation (104): the plug-in ratio is suppressed when ${\hat{f}}_{X,n}^{\left(0\right)}\left(x\right)$ falls below $10^{-6}$. For each sample size and 500 replications, we record the minimum estimated density over the evaluation grid and the frequency with which the threshold is activated.

No trimming event occurs in the 2000 replications summarized in Table 6; the smallest realized value of ${\hat{f}}_{X,n}^{\left(0\right)}$ over *I* is 0.244, far above the numerical floor. This conclusion is strictly design-specific. The evaluation set is interior, $f_{X}$ is bounded away from zero on *I*, and n≥250; the experiment therefore provides no evidence concerning boundary evaluation, near-vanishing design densities, or substantially smaller effective local sample sizes.

### 5.11. Effect of Serial Dependence

**Figure 7:**
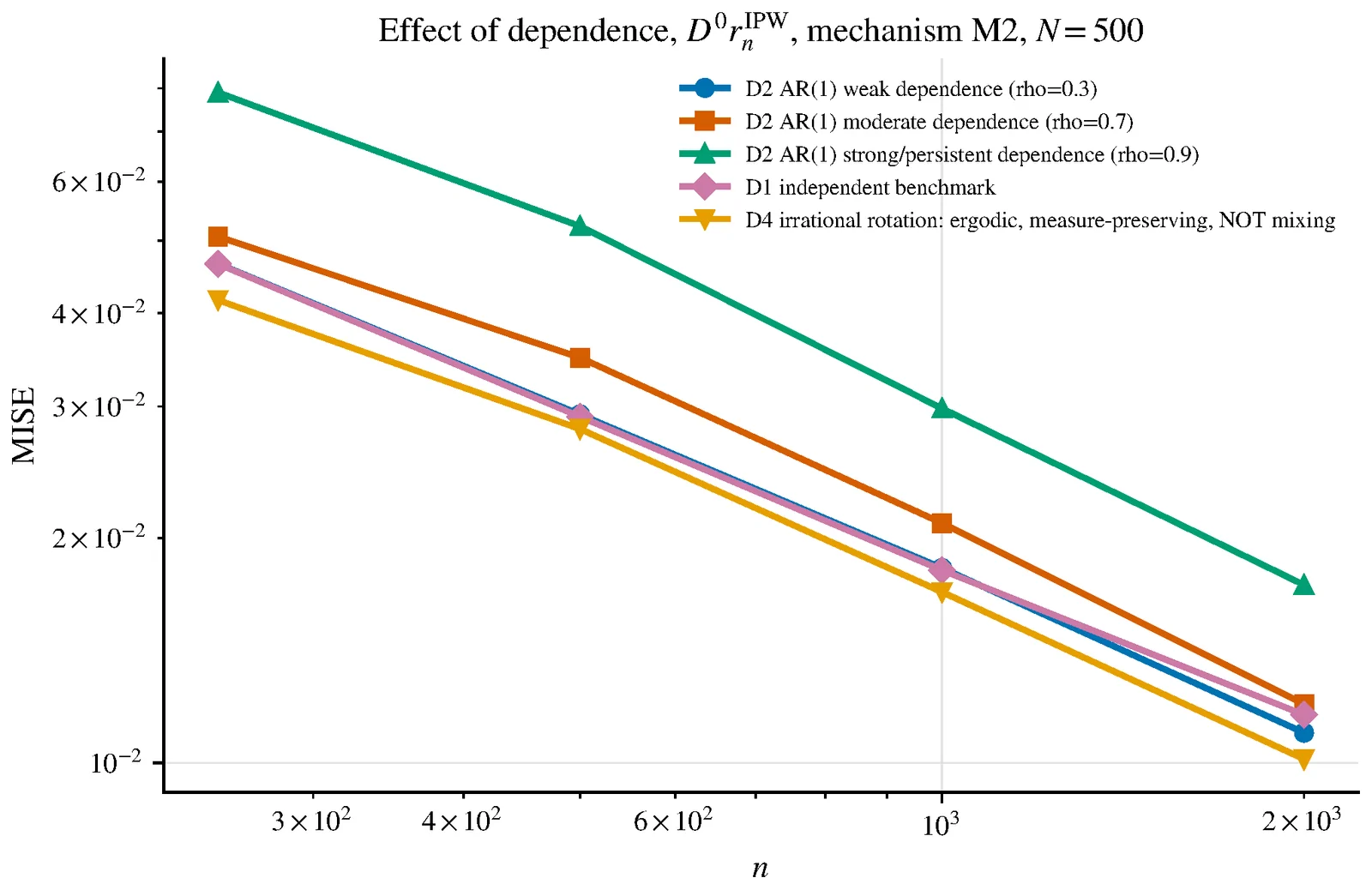
MISE of $D^{0}r_{n}^{\mathrm{IPW}}$ vs. *n* (log–log axes) across five dependence designs, mechanism M2, N=500.

The dependence experiment varies persistence systematically within the same stationary Gaussian-copula AR(1) family, using the independent benchmark and ρ∈{0.3,0.7,0.9}. This isolates the effect of increasing temporal persistence while keeping the marginal covariate law fixed, see the Table 7 and Figure 7.

Across the Gaussian AR(1) configurations, increasing persistence is accompanied by increasing pointwise dispersion at matched *n*: the mean standard deviation rises from approximately 0.072 under independence to 0.127 at ρ=0.9. This is a finite-sample association, not an estimate of any projective norm or long-run variance component appearing in the theoretical assumptions. In particular, no quantitative dependence condition is inferred from these MISE values.

The irrational-rotation configuration behaves differently: at n=1000, its mean standard deviation is 0.063, below the i.i.d. benchmark of 0.072. Since D4 is non-mixing and, more importantly for the present theorem architecture, falls outside the stated conditional-density hypothesis when conditioning on the past, this comparison is deliberately theorem-external. It demonstrates that favourable finite-sample behaviour can occur in an ergodic non-mixing design; it does not verify, refute, or calibrate the projective and conditional variance conditions used in the limit theory.

### 5.12. Effect of the Missingness Mechanism

**Figure 8:**
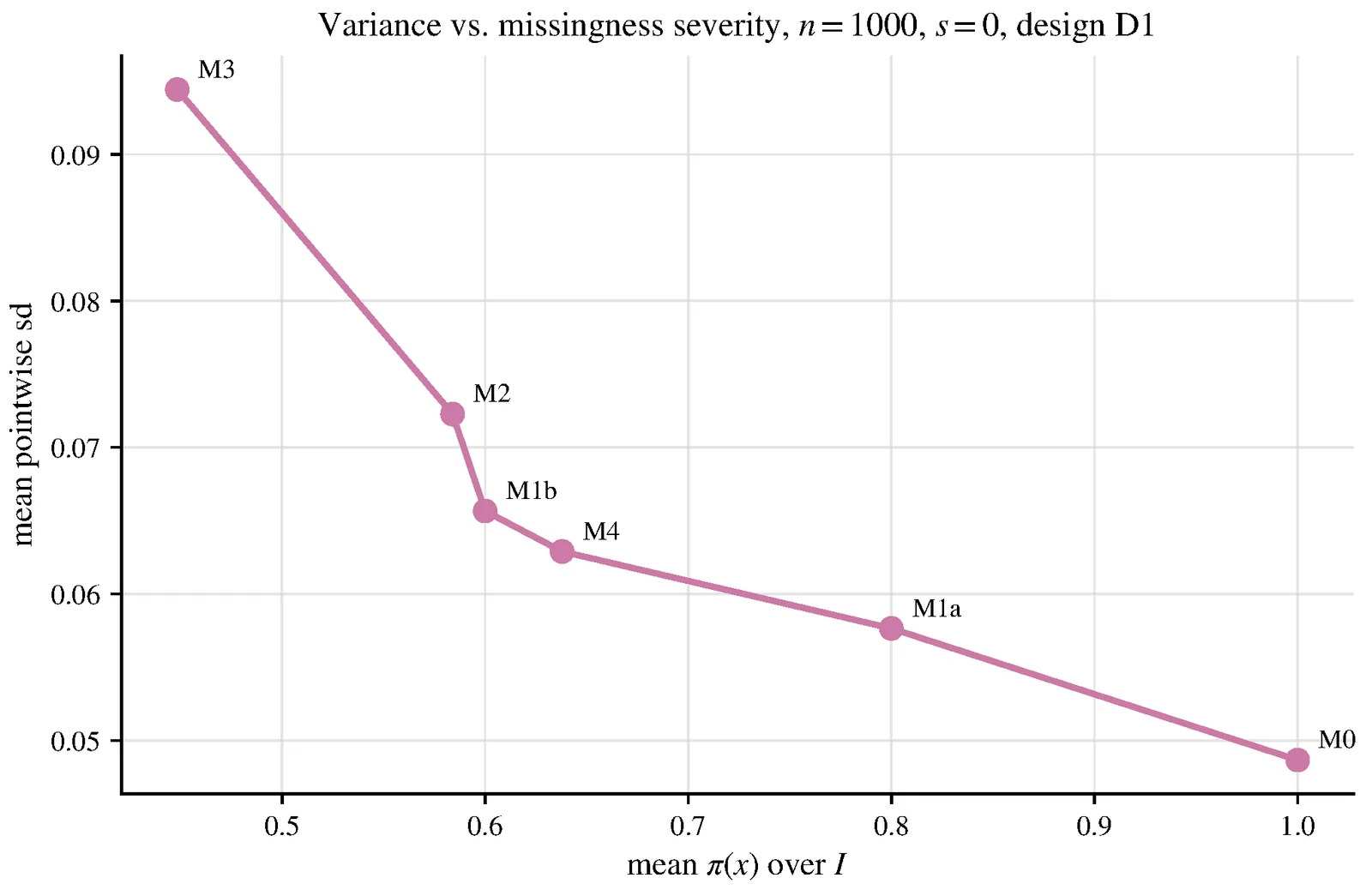
Mean pointwise sd of $D^{0}r_{n}^{\mathrm{IPW}}$ vs. domain-average $\bar{\pi }$, all six mechanisms, design D1, n=1000, N=500.

The domain-averaged pointwise standard deviation decreases across these six configurations as the domain-average observation probability increases. This ordering is descriptively compatible with the 1/π(x) inflation entering the local IPW variance. It is not a pointwise monotonicity theorem. The asymptotic variance depends on the local propensity π(x), the local design density, and the conditional second moment of the response; two propensity functions with different shapes can therefore have the same or reversed domain-average ordering while exhibiting different pointwise variances. The three-location analysis under M2 in Section 5.17 is used precisely to avoid conflating $\bar{\pi }$ with the local variance mechanism, see Table 8 and Figure 8.

### 5.13. Oracle and Feasible Propensity-Score Estimation

**Figure 9:**
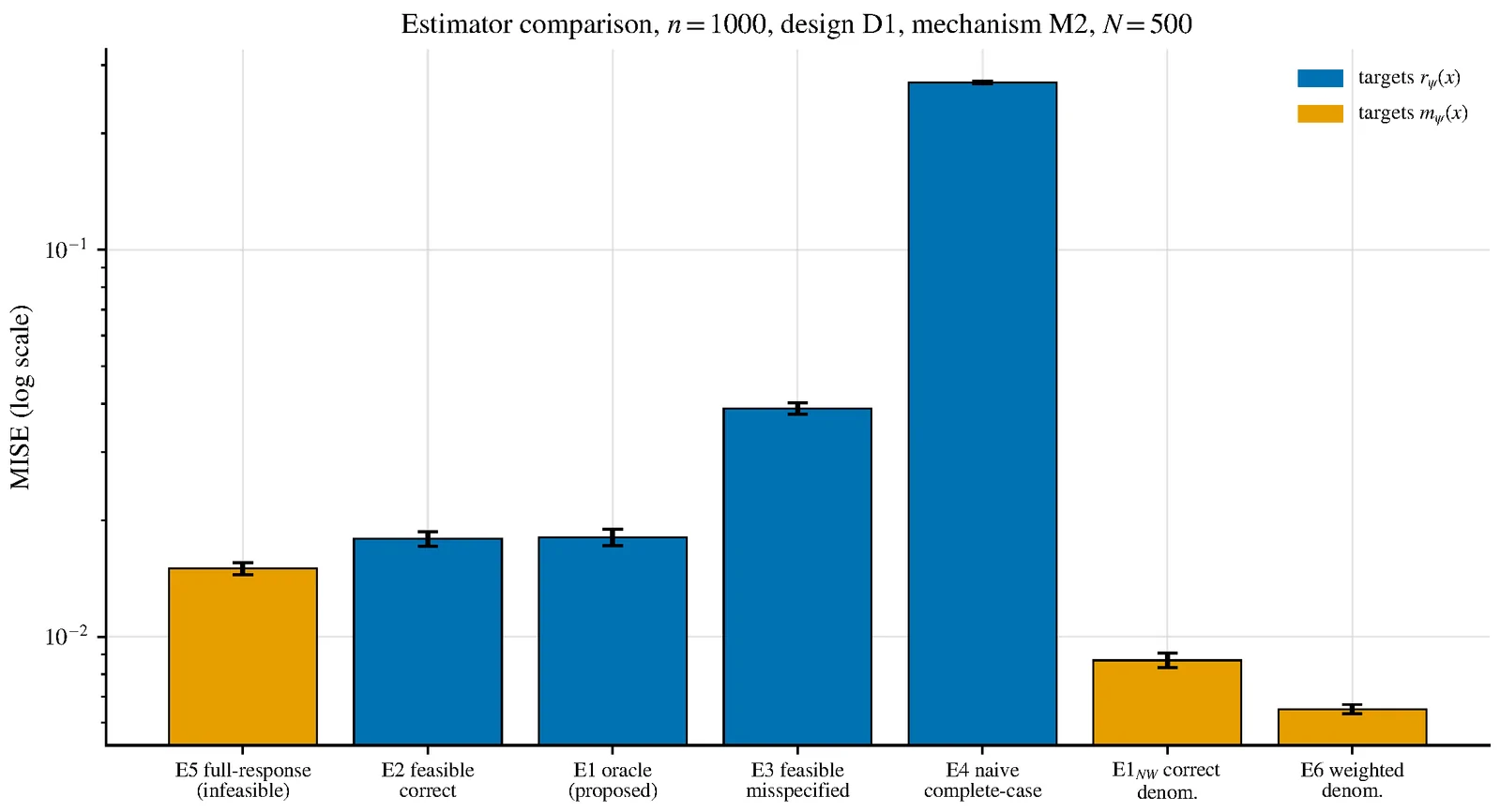
MISE (log scale), n=1000, design D1, mechanism M2, N=500. Error bars are ±1.96 Monte Carlo standard errors.

For the primitive target $r_{\psi }$, the oracle IPW estimator is dramatically more accurate than the uncorrected complete-case construction in this MAR design: at n=1000, the MISE ratio is approximately 15. This difference is consistent with the target discrepancy at the primitive level, since omitting $1/\pi (X_{i})$ changes the numerator expectation from $r_{\psi }\left(x\right)$ to $\pi \left(x\right)r_{\psi }\left(x\right)$ when π is nonconstant. The correctly specified feasible estimator E2 is numerically very close to the oracle E1 in MISE (0.0179 versus 0.0181), whereas the intercept-only misspecified estimator E3 more than doubles the oracle MISE, see Table 9 and Figure 9.

**Figure 10:**
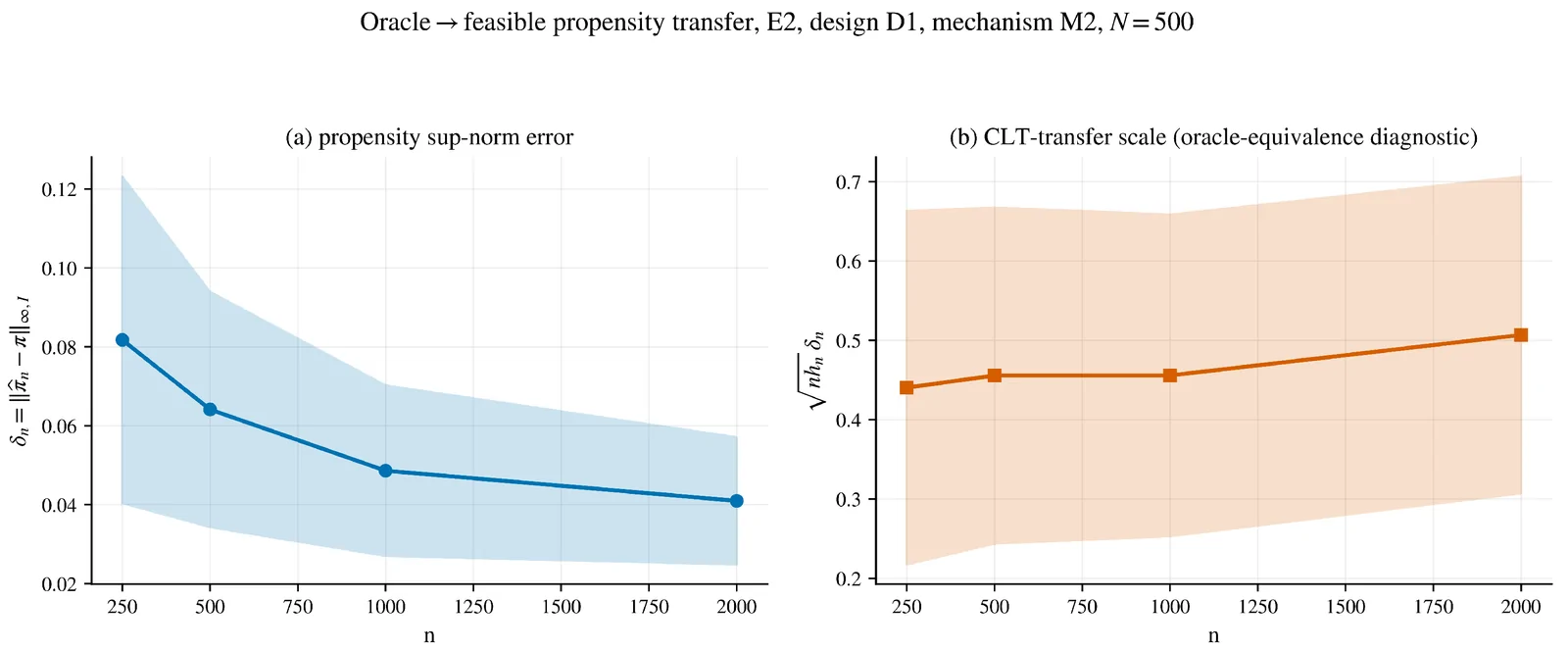
(**a**) $\delta_{n}={∥{\hat{\pi }}_{n}-\pi ∥}_{\infty ,I}$ vs. *n*; (**b**) $\sqrt{nh_{n}}\;\delta_{n}$ vs. *n* (CLT-transfer scale), E2, design D1, mechanism M2, N=500; shaded bands are ±1 Monte Carlo sd.

Closeness of MISE is not, however, equivalent to oracle equivalence at the Gaussian scale. To examine the sufficient transfer condition from the theory, we compute in every replication the grid approximation to $\delta_{n}={∥{\hat{\pi }}_{n}-\pi ∥}_{\infty ,I}$ together with the normalized quantity $\sqrt{nh_{n}}\;\delta_{n}$ for |s|=0, see Table 10 and Figure 10.

The unnormalized discrepancy $\delta_{n}$ decreases over the simulated range, whereas $\sqrt{nh_{n}}\;\delta_{n}$ does not display a corresponding decline (0.440 at n=250 and 0.507 at n=2000). The latter variation is small relative to its Monte Carlo dispersion and should not be interpreted as an increasing asymptotic trend. What the experiment establishes is narrower: the finite-sample sequence considered here does not numerically exhibit the vanishing transfer term required by the conservative sufficient condition for oracle-equivalent pointwise inference. This observation neither contradicts nor establishes any separate parametric-rate argument for the correctly specified logistic estimator; it only shows that oracle equivalence is not visible from this diagnostic over n≤2000.

### 5.14. Numerator–Denominator Covariance and Alternative Denominator Weighting

**Figure 11:**
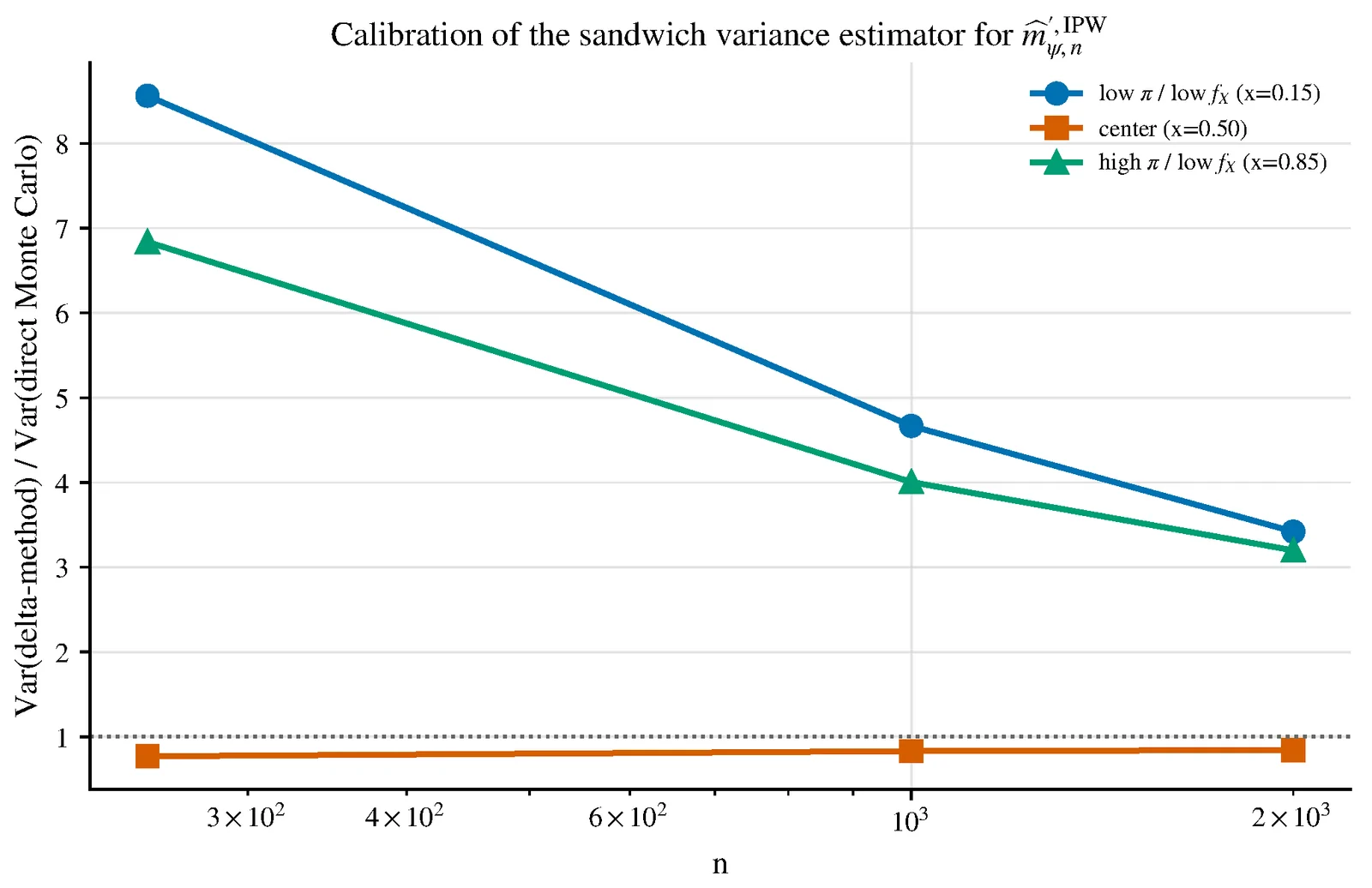
Ratio of delta-method to direct Monte Carlo variance of ${\hat{m}}_{\psi ,n}^{\prime ,\mathrm{IPW}}$, design D1, mechanism M2, N=500.

For ${\hat{m}}_{\psi ,n}^{\prime ,\mathrm{IPW}}$, variance propagation is assessed from the full primitive covariance vector rather than from marginal variances. Across N=500 replications, we compute the empirical 4×4 covariance matrix of $\left({\hat{r}}^{\left(1\right)},{\hat{r}}^{\left(0\right)},{\hat{f}}^{\left(1\right)},{\hat{f}}^{\left(0\right)}\right)$ under the order-one B1 bandwidth. The first-order delta-method variance, obtained by contracting this matrix with the exact Fréchet coefficient vector at $\left(r_{\psi },r_{\psi }^{\prime },f_{X},f_{X}^{\prime }\right)$, is then compared with the repeated-sampling variance of ${\hat{m}}_{\psi ,n}^{\prime ,\mathrm{IPW}}$ computed directly from the Monte Carlo replications. The latter contains no delta-method approximation and, therefore, provides an external calibration benchmark for the linear variance propagation, see Figure 11.

At the central design point, the first-order variance approximation is reasonably calibrated: over the sample sizes represented in the corresponding diagnostic, the delta/direct variance ratio lies between 0.77 and 0.84. At the two flanking locations, the approximation is substantially less accurate, with overestimation factors between 3.2 and 8.6. These are simultaneously low-density points, and the Fréchet coefficient multiplying the order-zero density error contains an $f_{X}^{-3}$ factor. Since $f_{X}\left(0.50\right)/f_{X}\left(0.15\right)≃3.84$, this component can be amplified by a factor of approximately $3.84^{3}≃57$ relative to the centre. Such conditioning can magnify both covariance-estimation noise and higher-order terms omitted by the linear approximation. The data support the conclusion that the delta approximation is poorly calibrated at these particular low-density points over the present sample-size range; they do not support a general failure of the asymptotic variance formula.

The comparison between ${\text{E1}}_{\mathrm{NW}}$ and the IPW-weighted denominator E6 also illustrates why marginal denominator variance does not determine ratio variance. At the reported n=1000 configuration, E6 has lower pointwise standard deviation for the final ratio (0.039 versus 0.059), even though its denominator variance is larger (0.00725 versus 0.00287). The empirical numerator–denominator correlation is correspondingly stronger under E6 (0.795 versus 0.516), because the numerator and denominator share the factor $\xi_{i}/\pi \left(X_{i}\right)$. The covariance term therefore offsets part of the marginal variance inflation.

This finding has a precise scope: It does not imply a universal efficiency ordering between weighted and unweighted denominator constructions. Under correct propensity weighting, both may target the marginal design density, but they generate different joint stochastic structures. This paper analyses the unweighted denominator because the covariates are fully observed and because that construction separates the response-missingness correction from direct estimation of $f_{X}$; the simulation shows that this structural choice should not be justified by a universal finite-sample ratio-variance claim.

### 5.15. Bias Propagation and Bias Correction

The numerical analysis reports the magnitude of the empirical regression-derivative bias and the accuracy of the first-order linearization, but it does not separately compute the propagated leading bias obtained by inserting the primitive bias expansions of Lemma 19 into the differential of the quotient map, nor is an oracle- or pilot-based bias-corrected estimator included. Consequently, Table 3 and Table 4 diagnose bias domination and nonlinear remainder size, but they are not a numerical test of the explicit leading-bias constant. A direct bias-propagation experiment would require computing that constant and comparing uncorrected, undersmoothed, and bias-corrected procedures on the same stochastic scale.

### 5.16. Bandwidth Behaviour

B1 is systematically smoother than the infeasible ISE-optimal choice B2 over the simulated range, with an MISE ratio of approximately 1.6–1.9. The covariate-ordered prediction criterion B3 selects substantially larger bandwidths and incurs MISE roughly 7–14 times that of B1 in the reported design. Because B3 optimizes squared prediction error for the raw pseudo-response $W_{i}\left(\psi \right)=\xi_{i}Y_{i}/\pi \left(X_{i}\right)$ rather than integrated error of the smoothed target, this behaviour is not unexpected: the criterion is directly exposed to the high conditional variability of the IPW pseudo-response. The result concerns this particular criterion only and provides no general negative conclusion about data-driven bandwidth selection for IPW kernel derivative estimators, see Table 11.

### 5.17. Pointwise Gaussian Approximation for Primitive Estimators

**Figure 12:**
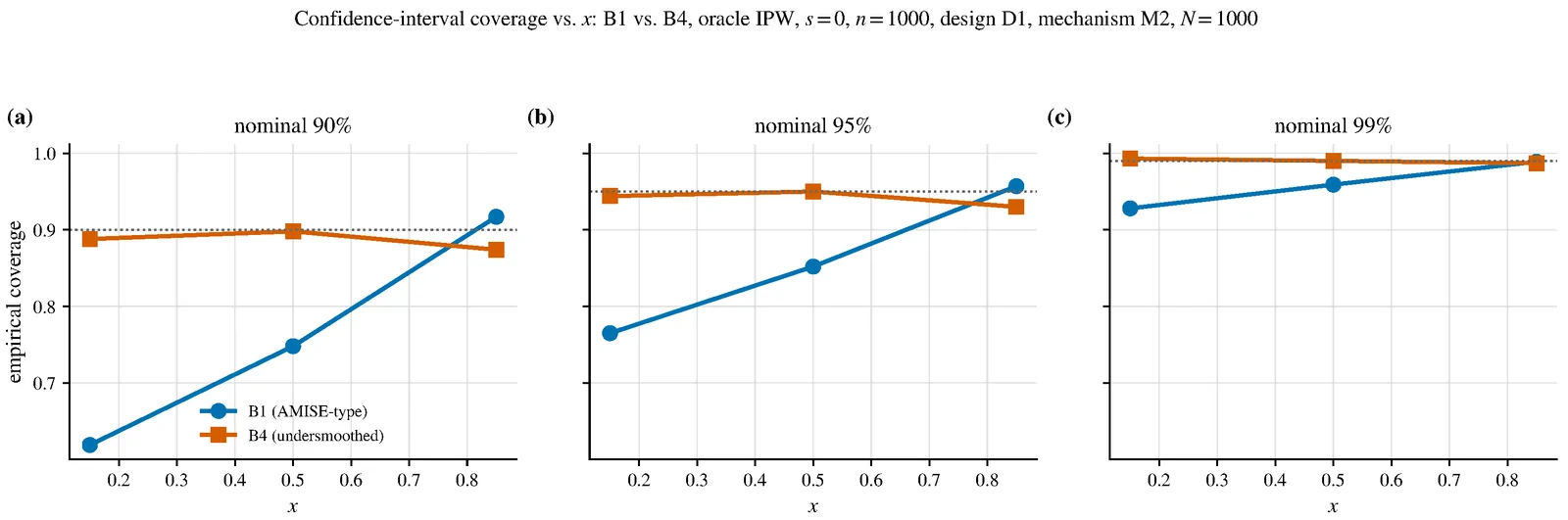
Empirical coverage of nominal 90%/95%/99% intervals for $r_{\psi }\left(x\right)$ vs. *x*, B1 vs. B4, oracle IPW, |s|=0, n=1000, design D1, mechanism M2, N=1000.

**Figure 13:**
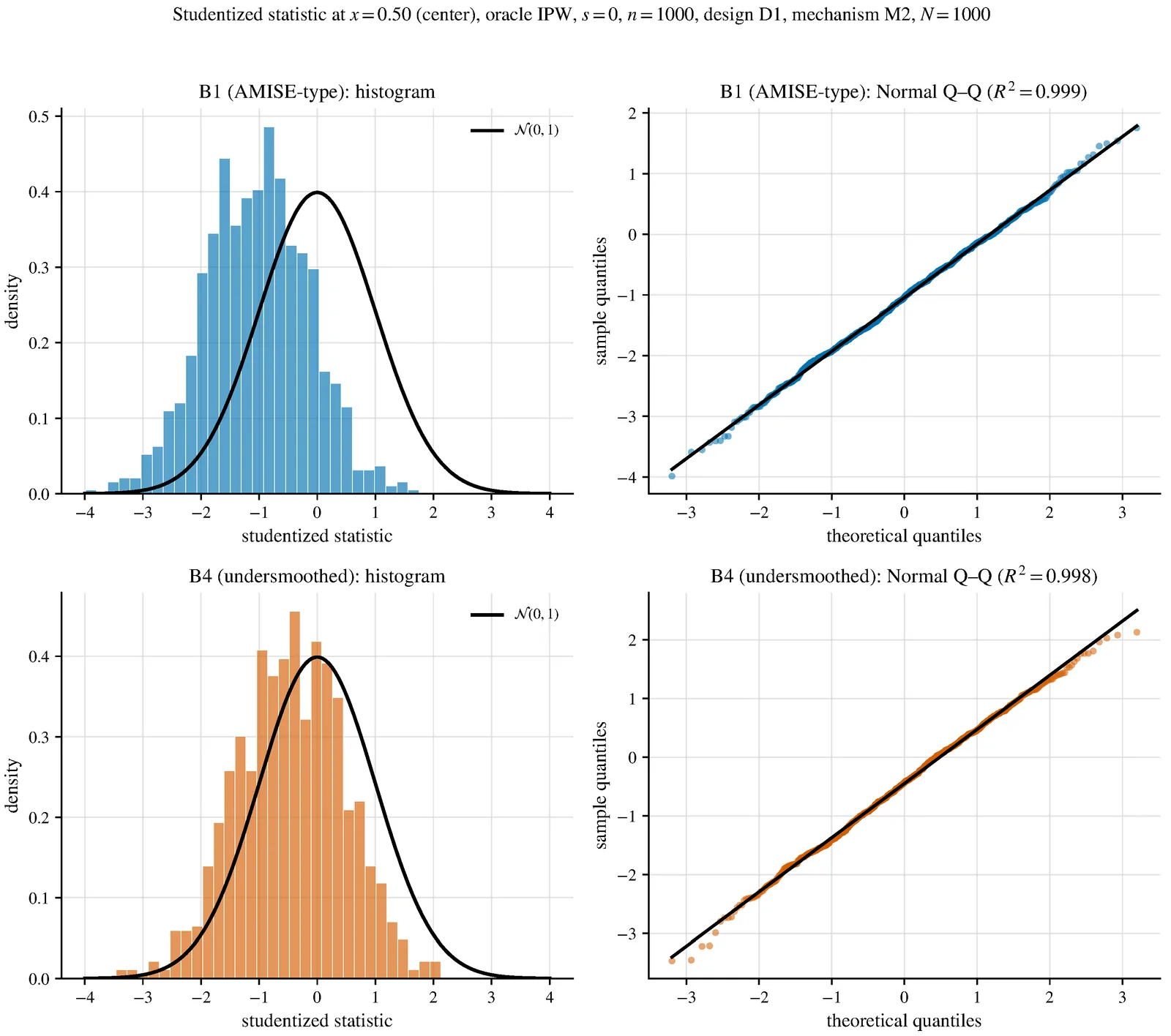
Histogram and normal Q–Q plot of the studentized statistic at x=0.50, B1 vs. B4, oracle IPW, |s|=0, n=1000, N=1000.

For the primitive order-zero estimator, B4 produces a clear reduction in studentized centring error relative to B1 at each of the three locations. Coverage correspondingly moves substantially toward nominal levels. At the 95% level, B4 attains 0.950 at the centre, while the low- and high-propensity points yield 0.944 and 0.930, respectively. Thus, undersmoothing materially improves the order-zero finite-sample approximation, but the improvement is not spatially uniform, and residual bias remains visible at the flanking points, see Table 12, Figure 12 and Figure 13.

This behaviour is compatible with the bias-negligibility mechanism in Theorem 4; it is not an empirical verification of that theorem. The experiment concerns |s|=0 only. Primitive studentization for |s|=1,2 is not reported; the substantially more difficult first regression derivative is examined separately in Section 5.18.

### 5.18. Studentized Inference for Regression Derivatives

For the first regression derivative, the studentizing variance is constructed from the covariance-inclusive sandwich of the four primitive estimators. The Fréchet coefficient vector is evaluated at $\left(\hat{r},{\hat{r}}^{\prime },\hat{f},{\hat{f}}^{\prime }\right)$, yielding a fully feasible standard-error estimate. We then study $\left\{{\hat{m}}_{\psi ,n}^{\prime ,\mathrm{IPW}}\left(x\right)-m_{\psi }^{\prime }\left(x\right)\right\}/\hat{\mathrm{se}}\left(x\right)$ at the three evaluation points under B1 and B4, for n∈{1000,2000} and N=1000 replications.

The regression-derivative experiment isolates a markedly different phenomenon from the primitive order-zero calculation. The estimated standard errors are well calibrated in scale: the ratio of the average estimated standard error to the empirical Monte Carlo standard deviation lies between 0.950 and 1.030 throughout the table. Nevertheless, the studentized statistics are severely miscentred, with absolute means between approximately 2.6 and 13.4. Consequently, B1 produces zero empirical coverage at the reported 90% and 95% levels, while B4 remains far below nominal coverage, never exceeding 0.257 at the 95% level.

The simultaneous occurrence of near-unit scale calibration and severe miscentring identifies the dominant finite-sample failure mode: smoothing bias is large relative to the stochastic standard error. It is therefore inappropriate to attribute the coverage failure to variance estimation. For |s|=1 and n≤2000, neither B1 nor the present B4 specification (δ=0.05) makes the target-centring bias negligible on the studentized scale. This does not contradict the asymptotic theorem: B4 is constructed to satisfy a stronger asymptotic exponent, but the corresponding regime is not yet numerically attained. Achieving useful target-centred coverage in this design would require more aggressive undersmoothing (equivalently, a larger additional bandwidth exponent δ), substantially larger *n*, or explicit bias correction. Studentized inference for the second regression derivative is not reported because the order-two point estimates are already strongly bias-dominated over the simulated range.

### 5.19. Practical Implementation and Interpretation

Four implementation points are especially important: First, regression derivatives are formed from the primitive kernel derivative estimators through the exact quotient identities of Section 4; they are not obtained by numerically differentiating a noisy fitted regression curve. Second, estimated propensities should be inspected for weak overlap, and any truncation or overlap restriction should be reported because it changes the finite-sample bias–variance trade-off and may alter the effective target. Third, a bandwidth that is attractive for estimation need not be valid for target-centred inference. Table 13 shows precisely this point: the estimated standard-error scale is close to the repeated-sampling scale, while coverage is poor because smoothing bias dominates the centred statistic. More aggressive undersmoothing, explicit bias correction, or larger samples may therefore be necessary. Fourth, higher derivative order, low local design density, and strong serial dependence reduce the effective information available in the local window, so asymptotic intervals should be interpreted conservatively in such regimes. These recommendations are diagnostics and implementation safeguards; they do not replace verification of the theorem assumptions for a particular stochastic model.

### 5.20. Empirical Convergence Rates

**Figure 14:**
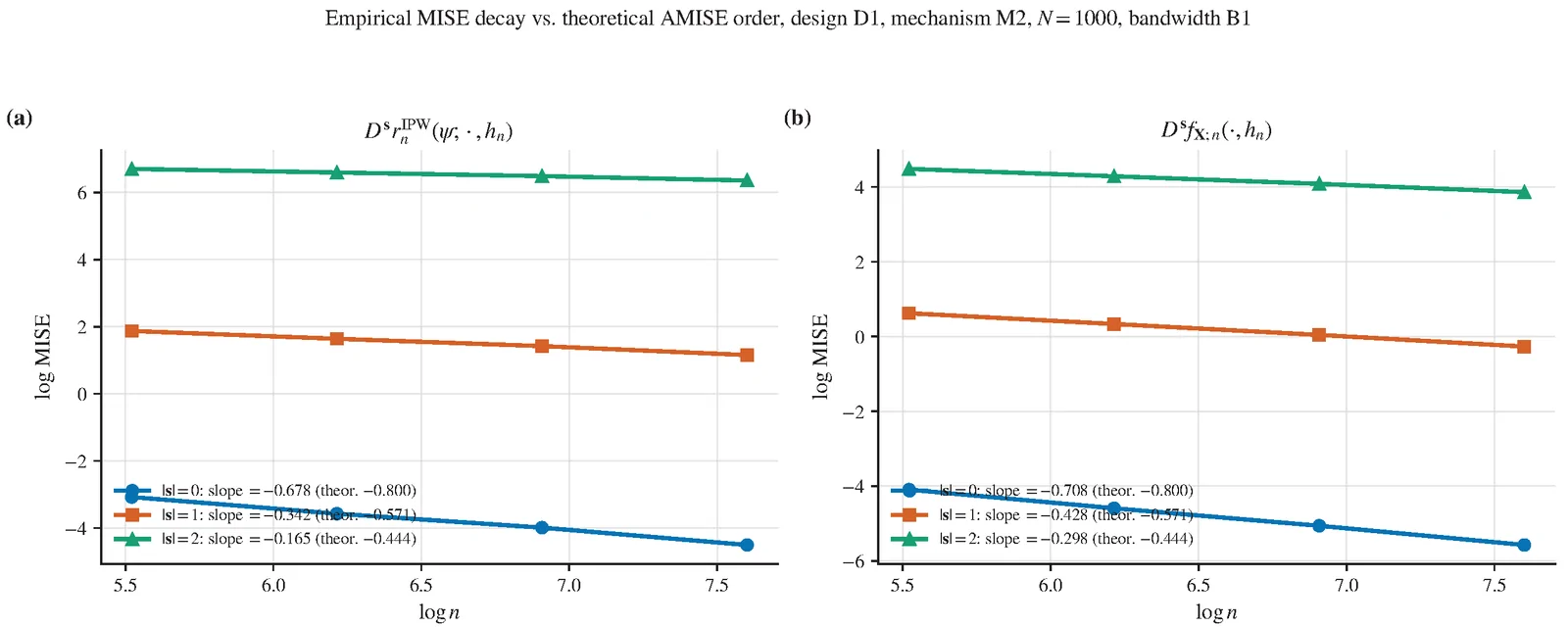
Empirical logMISE vs. logn, |s|=0,1,2, design D1, mechanism M2, N=1000, bandwidth B1.

All fitted log–log slopes are shallower than the corresponding AMISE exponent −2ℓ/(1+2|s|+2ℓ), with the discrepancy increasing with the derivative order. The very high $R^{2}$ values therefore quantify the linearity of the four-point log–log relation; they do not establish agreement with the asymptotic rate. Combined with the bandwidth comparison in Table 11 and the large order-two biases in Table 2, the slope pattern is consistent with a pre-asymptotic bias-dominated regime under the fixed constant c=0.35. With only four sample sizes between 250 and 2000, the experiment cannot separate convergence toward the eventual AMISE exponent from persistent finite-sample bias. No rate claim is therefore inferred from these regressions, see Table 14 and Figure 14.

### 5.21. Summary of Numerical Findings

The discussion is deliberately not framed only as confirmation of the asymptotic theory: it emphasizes the configurations in which bias remains substantial, coverage is poor, or the experiment lies outside the theorem hypotheses.

The simulation produces three qualitatively distinct messages: First, at the primitive order-zero level, inverse-probability correction behaves as expected under heterogeneous MAR: the oracle IPW estimator is far more accurate than the uncorrected primitive complete-case estimator, misspecification of the propensity model is costly, and the correctly specified feasible estimator is close to the oracle in MISE. Undersmoothing substantially improves the studentized order-zero approximation and attains nominal 95% coverage at the central evaluation point, although the improvement is not uniform over the domain.

Second, regression derivatives are materially more demanding. The first-order Fréchet remainder becomes small at the centre but remains non-negligible at low-density points; the full covariance structure is indispensable for variance propagation; and, most importantly, the studentized first regression derivative is dominated by smoothing bias for n≤2000. The near-unit SE ratios show that the principal coverage failure is miscentring rather than stochastic-scale estimation. The second derivative is even more strongly pre-asymptotic under the bandwidths considered.

Third, several experiments are intentionally theorem-external. The irrational-rotation design is a non-mixing ergodic stress test but does not satisfy the stated past-conditional density hypothesis, and the Gaussian kernel does not satisfy the compact-support clause of (K.1). Their numerical behaviour must therefore be read as robustness evidence, not as verification of the non-mixing theorem class. Similarly, the dependent Gaussian AR configurations are not used to infer projective or conditional-variance assumptions from finite-sample MISE values.

The numerical study does not include pilot-based bias correction, studentized second-derivative inference, a bias-separated component-wise verification of the highest-order stochastic reduction, or a theorem-level verification of the full dependence modules for D2. These omissions delimit the scope of the numerical claims. Within that scope, the experiments identify the practically relevant finite-sample mechanisms with unusual clarity: derivative order, local design density, propensity misspecification, smoothing bias, and covariance propagation all have effects that are quantitatively visible and must be distinguished from the asymptotic assumptions themselves.

## 6. Concluding Remarks and Perspectives

The principal scientific achievement of this paper is a probability-theoretic route from sequential-MAR identification and primitive IPW kernel derivative processes to asymptotic theory for nonlinear regression derivatives under stationary dependent sampling, with the dependence requirements stated through explicit quantitative conditions rather than a generic mixing coefficient. The construction exploits the asymmetric informational structure of the problem: response-dependent functionals are recovered through inverse probability weighting, whereas the marginal distribution of the covariates is estimated from the complete covariate trajectory. Uniform rates, pointwise Gaussian limits, AMISE, higher-order quotient identities, feasible propensity replacement, and the simulations are consequences or extensions of this core construction rather than separate co-equal claims of novelty.

The resulting theory applies first to derivatives of the density-weighted conditional functional $r_{\psi }$ and is subsequently transferred to derivatives of conditional regression functionals through the differential structure of the quotient map $m_{\psi }=r_{\psi }/f_{X}$. In particular, the framework simultaneously accommodates derivatives of the marginal covariate density, conditional means, conditional moments, and, whenever ψ is chosen as an indicator-type transformation and the required regularity conditions hold, covariate derivatives of conditional distribution functionals. The analysis thus provides a common probabilistic structure for a broad class of derivative-estimation problems that are often treated separately.

For the oracle IPW estimator, uniform almost-sure convergence is obtained by combining a precise decomposition of the centred estimator into martingale and predictable components with uniform control of the kernel-derivative class and an explicit deterministic bias expansion. The resulting rates display the expected interaction among effective dimension, derivative order, smoothing order, and bandwidth. In particular, differentiation amplifies stochastic fluctuations through the factor induced by kernel rescaling, while the approximation error remains governed by the order of the available smoothness and the moment cancellations of the kernel. This decomposition makes explicit the statistical price of estimating higher-order derivatives and clarifies the balance required between stochastic localization and deterministic smoothing.

At the pointwise level, central limit theorems are established under local conditional-variance stabilization together with appropriate conditional moment and Lindeberg-type requirements. The limiting variance explicitly exhibits the information loss induced by missing responses through the inverse propensity factor. Consequently, regions of weak observation probability produce intrinsically larger asymptotic uncertainty, even when the underlying regression functional is otherwise smooth. For regression derivatives, the asymptotic distribution is obtained by linearizing the relevant quotient functional with respect to the joint vector of density-weighted and marginal-density derivatives. The limiting covariance structure therefore necessarily contains the cross-covariances between numerator and denominator estimators; neglecting these terms generally yields an incorrect first-order variance approximation.

The integrated-risk analysis complements the pointwise theory. Under the corresponding strengthened assumptions, an asymptotic mean integrated squared error expansion is derived with explicit leading bias and variance contributions. The resulting bandwidth order simultaneously quantifies the effects of smoothness, derivative order, and dimension. In particular, the deterioration of the optimal convergence rate with increasing derivative order or covariate dimension is transparent from the expansion. Equally importantly, the analysis separates the bandwidth problem for estimation from that for inference. An AMISE-optimal bandwidth balances squared bias and variance and, therefore, does not generally render the bias negligible after central-limit normalization. Target-centred Gaussian inference consequently requires either additional undersmoothing or an explicit bias-removal procedure.

Stationarity and ergodicity provide the structural framework, but the quantitative rates and Gaussian limits rely on the theorem-specific projective/maximal, conditional-moment, conditional-density, and variance-stabilization conditions collected in Section 3.2 and Section 3.11. Likewise, feasible inference requires the propensity-estimation remainder to be negligible at the stochastic scale of the result being transferred; the direct plug-in conditions are summarized in Section 3.10. These two qualifications—quantitative dependence control and rate-compatible nuisance estimation—are the operative scope conditions for the asymptotic conclusions, and the detailed assumptions are not repeated here.

A natural continuation of the present work is consequently the construction of genuinely feasible inferential procedures whose first-order behaviour is less sensitive to nuisance estimation. Cross-fitting alone can remove empirical-process restrictions associated with using the same observations for nuisance estimation and target estimation, but it does not by itself eliminate first-order sensitivity to propensity error. A more complete theory should therefore combine sample splitting with Neyman-orthogonal or doubly robust representations specifically adapted to kernel-derivative functionals. Such a development would require deriving orthogonal scores at the derivative-functional level, identifying the admissible joint rates for propensity and outcome nuisance estimators, and establishing corresponding uniform and pointwise limit theory under dependence.

Several additional directions appear particularly consequential. First, the substantial finite-sample bias inherent in derivative estimation motivates a systematic theory of explicit bias correction and robust bias-corrected studentization, rather than reliance exclusively on undersmoothing. Second, local-polynomial constructions may reduce boundary effects and provide a natural mechanism for simultaneous estimation of several derivative orders. Third, anisotropic bandwidth matrices would permit coordinate-specific regularity and are particularly relevant when derivatives are taken only with respect to selected components of a multivariate covariate. Fourth, weak-overlap regimes require methods that remain stable when the propensity approaches zero; truncation, overlap weighting, or regularized inverse weighting would then have to be incorporated directly into both the bias and variance theory.

Further extensions concern the nature of the target and of the stochastic dependence. Conditional quantile and other implicitly defined regression derivatives require an additional functional inversion step and, therefore, a Bahadur-type linearization adapted to the MAR and dependent setting. Censoring or more general coarsening mechanisms would replace the simple IPW structure with more elaborate observation operators. Locally stationary processes would require the simultaneous control of time localization and covariate localization. Functional covariates raise a different set of difficulties, because Euclidean bandwidth powers must then be replaced by small-ball probabilities or other local concentration quantities, and the derivative concept itself must be formulated in an appropriate infinite-dimensional geometry. These extensions are structurally related to the present analysis but require genuinely new probabilistic arguments rather than formal substitutions.

Another important direction concerns inference beyond a fixed covariate point. The Gaussian results obtained here are pointwise. Simultaneous confidence bands, extrema of derivative processes, tests of monotonicity or curvature, and uniform inference for regression derivatives would require a functional Gaussian approximation for the appropriately normalized derivative process, together with anti-concentration and feasible covariance estimation. Under stationary dependent sampling and estimated propensities, establishing such results would considerably strengthen the inferential scope of the methodology.

The scope limitations are substantive. The theory does not cover arbitrary stationary ergodic sequences, because the theorem-specific quantitative conditions must still be verified. It is an MAR theory: MCAR is included, but MNAR generally requires additional identification structure, and zero propensity on the target region destroys identification. Direct multivariate smoothing also remains subject to the curse of dimensionality, while second and higher derivatives can require sample sizes much larger than those adequate for order-zero estimation. These limitations explain why the numerical section emphasizes dependence strength, weak overlap, derivative order, and smoothing bias rather than treating asymptotic validity as automatic.

The numerical investigation reinforces several of these theoretical distinctions. In the configurations considered, inverse-probability correction successfully addresses the target distortion induced by MAR sampling, whereas higher-order derivative estimation remains markedly bias-sensitive at moderate sample sizes. For the first regression derivative, undersmoothing reduces the centring error but does not uniformly deliver satisfactory target-centred coverage over the investigated sample-size range. The simulations therefore support the theoretical separation between variance calibration and bias control: an accurately estimated stochastic scale is insufficient for reliable inference when the smoothing bias remains non-negligible relative to that scale. They also illustrate that empirical performance under a stationary ergodic non-mixing design cannot substitute for analytical verification of the quantitative dependence hypotheses entering the theorems.

In summary, this paper provides a probability-theoretic foundation for kernel-derivative estimation under sequential MAR sampling and stationary dependent observations within a class characterized by explicit non-mixing quantitative conditions. The proposed framework preserves the complete-data target through inverse-probability correction of response-dependent quantities, exploits the fully observed covariate trajectory for marginal estimation, and propagates the resulting joint asymptotics through the nonlinear maps defining regression derivatives. Uniform almost-sure rates, pointwise Gaussian limits, integrated-risk expansions, optimal bandwidth orders, and oracle–feasible comparison bounds are obtained within a common analytical structure. Beyond the particular estimators considered here, the main contribution is therefore a general route from primitive kernel-derivative processes under incomplete dependent sampling to asymptotic theory for nonlinear conditional functionals. Extending that route to orthogonal feasible inference, explicit bias correction, simultaneous inference, weak overlap, and more general dependent or infinite-dimensional settings constitutes a natural programme for further investigation.

## 7. Mathematical Developments

This section provides the complete proofs of the results stated in Section 3 and Section 4, together with the auxiliary measure-theoretic, probabilistic, and deterministic results needed to make every reduction explicit. All arguments are formulated on the two-sided stochastic basis introduced in Section 2. The filtration is therefore indexed by Z, all sigma-fields are understood to be completed with respect to P, and conditional expectations and regular conditional distributions are fixed in jointly measurable versions compatible with the uniform-in-x statements used below. In particular, every assertion involving a conditional density or conditional moment field is understood relative to the version conventions specified in Section 7.1.

The proof strategy follows the logical structure of the main results. We first isolate the exact martingale-predictable decomposition of the localized IPW derivative process. We then establish the measure-theoretic identities, projective and maximal bounds, conditional convolution formulae, and bias expansions required for uniform consistency. Pointwise asymptotic normality is derived from a self-contained martingale triangular-array theorem, after which the primitive joint limits are propagated through the quotient map defining regression derivatives. The final part of this section treats nonlinear remainder control, local influence representations, feasible propensity replacement, studentization, complete-case alternatives, and integrated-risk expansions. No appeal to mixing is introduced at the proof stage; every use of temporal dependence is mediated by the quantitative modules stated explicitly in Section 3.

Throughout the section, write

$$b_{n}=h_{n}^{1/p},\;\kappa_{s}=D^{s}K,$$

and, for x∈I,

$$\Theta_{i,n}\left(x\right)=W_{i}\left(\psi \right)\kappa_{s}\left(\frac{x-X_{i}}{b_{n}}\right).$$

Set

$$A_{i,n}\left(x\right)=E\left\{\Theta_{i,n}\left(x\right)|G_{i-1}\right\},\;\Delta_{i,n}\left(x\right)=\Theta_{i,n}\left(x\right)-A_{i,n}\left(x\right).$$

Then

$$E\{\Delta_{i,n}\left(x\right)|G_{i-1}\}=0$$

and

$$D^{s}r_{n}^{\;\mathrm{IPW}}\left(\psi ;x,h_{n}\right)=\frac{1}{nh_{n}^{1+|s|/p}}\sum_{i=1}^{n}\Theta_{i,n}\left(x\right).$$

The centred estimator admits the exact decomposition

(164) $$\begin{array}{cc} \; & D^{s}r_{n}^{\;\mathrm{IPW}}\left(\psi ;x,h_{n}\right)-ED^{s}r_{n}^{\;\mathrm{IPW}}\left(\psi ;x,h_{n}\right)\\ \; & \;=\frac{1}{nh_{n}^{1+|s|/p}}\sum_{i=1}^{n}\Delta_{i,n}\left(x\right)\\ \; & \;+\frac{1}{nh_{n}^{1+|s|/p}}\sum_{i=1}^{n}\left[A_{i,n}\left(x\right)-EA_{i,n}\left(x\right)\right]\\ \; & \;=M_{n,s}\left(x\right)+P_{n,s}\left(x\right). \end{array}$$

The decomposition is exact. The term $M_{n,s}$ is the normalized sum of martingale differences relative to $\left(G_{i}\right)$, whereas $P_{n,s}$ is the centred predictable component generated by the conditional law given the past. This separation is the organizing device for the entire analysis: concentration and Gaussian fluctuation are derived from the martingale component, while the quantitative dependence assumptions are used to show that the predictable component is negligible at the relevant uniform or pointwise normalization.

### 7.1. Measure-Theoretic Foundations and Version Conventions

Several arguments below simultaneously involve regular conditional distributions, conditional densities viewed as random fields, Bochner-valued conditional expectations, differentiation under an integral sign, and weak integration by parts. Since later suprema are taken over uncountable covariate sets, pointwise almost-sure identities are not sufficient by themselves. We therefore fix the relevant versions and interchange conventions at the outset, so that all subsequent uniform statements are interpreted on a common probability-one set whenever the hypotheses permit such a selection.

Let

$$G_{-\infty }=\underset{j\in Z}{⋂}G_{j},\;G_{\infty }=\sigma \left(\underset{j\in Z}{⋃}G_{j}\right).$$

For 1≤r<∞ and a separable Banach space *B*, $L^{r}\left(\Omega ,A,P;B\right)$ denotes the Bochner space of strongly measurable *B*-valued random elements with finite *r*-th norm moment. Whenever *B* is a separable Hilbert space, conditional expectation is understood as the Bochner conditional expectation.

Since Euclidean state spaces are standard Borel, the required regular conditional distributions exist. We fix kernels

$$P_{i}^{X}\left(\omega ,du\right)=P\left(X_{i}\in du|G_{i-1}\right)\left(\omega \right)$$

and

$$P_{i}^{W,X}\left(\omega ,dw,du\right)=P\left(\left(W_{i}\left(\psi \right),X_{i}\right)\in dw\;du|G_{i-1}\right)\left(\omega \right).$$

Assumption (P.1) selects versions for which

$$P_{i}^{X}\left(\omega ,du\right)=f_{i}\left(\omega ,u\right)\;du$$

on J, with $\left(\omega ,u\right)\mapsto f_{i}\left(\omega ,u\right)$ measurable with respect to $G_{i-1}⊗B\left(J\right)$. Likewise,

$$g_{i,\psi }\left(\omega ,u\right)=\int_{R}w\;P_{i}^{W|X}\left(\omega ,u,dw\right)f_{i}\left(\omega ,u\right)$$

is selected jointly measurable. Whenever a subsequent assertion is uniform in the covariate argument, the versions are selected so that the relevant identities hold outside a single P-null set independent of u. This convention avoids reselecting versions after taking suprema and is used throughout without further comment.

We also fix the following measurability convention for suprema of random fields: Whenever a field is assumed to possess a continuous or Hölder representative on the compact set J, that representative is used; then, for any fixed countable dense subset $J_{0}\subset J$,

$$\underset{u\in J}{\sup}\left|Q\left(\omega ,u\right)\right|=\underset{u\in J_{0}}{\sup}\left|Q\left(\omega ,u\right)\right|.$$

The right-hand side is measurable. Likewise, when a field is controlled in $H^{m}\left(J\right)$ with m>p/2, we use its continuous Sobolev representative furnished by the embedding $H^{m}\left(J\right)↪C\left(J\right)$. Thus, every supremum appearing below is a genuine random variable rather than an outer-measurability shorthand.

**Lemma 4** 
(Conditional disintegration and conditional Fubini). *Let H⊂A be a sigma-field, let $X:\Omega \to R^{p}$ and V:Ω→R, and suppose that the conditional law of X given H has a jointly measurable density $f_{H}\left(\omega ,u\right)$. Assume that there is a jointly measurable function $m_{H}\left(\omega ,u\right)$ satisfying*

$$m_{H}\left(\omega ,u\right)=E\left(V|X=u,H\right)\left(\omega \right)$$

*in the regular-conditional sense; more precisely, the equality is understood for $P\left(d\omega \right)f_{H}\left(\omega ,u\right)\;du$-almost every (ω,u). If $\varphi :\Omega \times R^{p}\to R$ is $H⊗B(R^{p})$-measurable and*

$$E\int_{R^{p}}\left|\varphi \left(\omega ,u\right)|\right|m_{H}\left(\omega ,u\right)|f_{H}\left(\omega ,u\right)\;du<\infty ,$$

*then*

(165) $$E\left[V\varphi (\cdot ,X)|H\right]\left(\omega \right)=\int_{R^{p}}\varphi \left(\omega ,u\right)m_{H}\left(\omega ,u\right)f_{H}\left(\omega ,u\right)\;du$$

*for almost every ω.*

**Proof.**  Let

Q(ω,dv,du)=P((V,X)∈dvdu∣H)(ω)

be a regular conditional law. By the defining property of a regular conditional distribution, for every bounded H-measurable random variable *H*,

$$\begin{array}{cc} E\left[HV\varphi (\cdot ,X)\right] & =E\left[H\int_{R\times R^{p}}v\varphi \left(\cdot ,u\right)Q\left(\cdot ,dv,du\right)\right]. \end{array}$$

Disintegrate *Q* with respect to its *X*-marginal:

$$Q\left(\omega ,dv,du\right)=Q^{V|X}\left(\omega ,u,dv\right)f_{H}\left(\omega ,u\right)\;du.$$

The assumed absolute integrability permits Fubini–Tonelli, giving

$$\begin{array}{cc} & \int_{R\times R^{p}}v\varphi \left(\omega ,u\right)Q\left(\omega ,dv,du\right)\\ & =\int_{R^{p}}\varphi \left(\omega ,u\right)\left[\int_{R}vQ^{V|X}\left(\omega ,u,dv\right)\right]f_{H}\left(\omega ,u\right)\;du\\ & =\int_{R^{p}}\varphi \left(\omega ,u\right)m_{H}\left(\omega ,u\right)f_{H}\left(\omega ,u\right)\;du. \end{array}$$

The last random variable is H-measurable. Since its integral against every bounded H-measurable *H* equals E[HVφ(·,X)], it is a version of the conditional expectation in (165). □

**Lemma 5** 
(Conditional IPW moment identities). *Under* (S.2)*, for every a>0 for which the conditional moment exists,*

(166) $$E\left[|W_{i}{\left(\psi \right)|}^{a}|X_{i},G_{i-1}\right]=\pi {\left(X_{i}\right)}^{1-a}E\left[|\psi \left(Y_{i}\right){|}^{a}|X_{i},G_{i-1}\right]\;a.s.$$

*For a=1, the same identity holds with $|W_{i}\left(\psi \right)|$ and $\left|\psi (Y_{i})\right|$. Without absolute values,*

(167) $$E\left[W_{i}\left(\psi \right)|X_{i},G_{i-1}\right]=E\left[\psi \left(Y_{i}\right)|X_{i},G_{i-1}\right].$$

**Proof.**  Because $\xi_{i}\in \left\{0,1\right\}$,

$$|W_{i}{\left(\psi \right)|}^{a}=\frac{\xi_{i}{\left|\psi \left(Y_{i}\right)\right|}^{a}}{\pi {\left(X_{i}\right)}^{a}}.$$

Condition first on $\sigma \left(X_{i},Y_{i}\right)\vee G_{i-1}$. Sequential MAR gives

$$E\left[\xi_{i}|X_{i},Y_{i},G_{i-1}\right]=\pi \left(X_{i}\right).$$

Hence,

$$\begin{array}{cc} & E\left[|W_{i}{\left(\psi \right)|}^{a}|X_{i},Y_{i},G_{i-1}\right]\\ & =\frac{|\psi \left(Y_{i}\right){|}^{a}}{\pi {\left(X_{i}\right)}^{a}}\pi \left(X_{i}\right)=\pi {\left(X_{i}\right)}^{1-a}{\left|\psi \left(Y_{i}\right)\right|}^{a}. \end{array}$$

Apply the tower property with respect to $\sigma \left(X_{i}\right)\vee G_{i-1}$ to obtain (166). The signed first-moment identity is identical, with $\psi (Y_{i})$ in place of its absolute power. Positivity guarantees that every division is well defined. □

**Lemma 6** 
(Differentiation under the response integral). *Assume* (R.2)*. For every multi-index **β** with |β|≤|s|+ℓ, the map*

$$x⟼r\left(\psi ;x\right)=\int_{R^{q}}\psi \left(y\right)f_{X,Y}\left(x,y\right)\;dy$$

*is classically differentiable on the interior of J, and*

(168) $$D^{\beta }r\left(\psi ;x\right)=\int_{R^{q}}\psi \left(y\right)D_{x}^{\beta }f_{X,Y}\left(x,y\right)\;dy.$$

*The derivative is continuous and bounded on J.*

**Proof.**  It suffices to establish the assertion for a single coordinate derivative and then iterate the argument over the multi-index. Let $e_{j}$ denote the *j*-th coordinate vector, and take x in the interior of J. For sufficiently small t≠0,

$$\begin{array}{cc} & \frac{r\left(\psi ;x+te_{j}\right)-r\left(\psi ;x\right)}{t}\\ & =\int_{R^{q}}\psi \left(y\right)\frac{f_{X,Y}\left(x+te_{j},y\right)-f_{X,Y}\left(x,y\right)}{t}\;dy. \end{array}$$

For almost every y, the mean-value theorem gives a θ=θ(t,x,y)∈(0,1) such that the difference quotient equals

$$\partial_{x_{j}}f_{X,Y}\left(x+\theta te_{j},y\right).$$

Assumption (R.2) gives the integrable domination

$$\left|\psi \left(y\right)\right|\left|\partial_{x_{j}}f_{X,Y}\left(x+\theta te_{j},y\right)\right|\leq H_{e_{j},\psi }\left(y\right).$$

Dominated convergence therefore yields

$$\partial_{x_{j}}r\left(\psi ;x\right)=\int \psi \left(y\right)\partial_{x_{j}}f_{X,Y}\left(x,y\right)\;dy.$$

Apply this argument inductively to every derivative of order less than |s|+ℓ. Continuity follows by dominated convergence applied to the derivatives, and boundedness follows from

$$\begin{array}{cc} \underset{x\in J}{\sup}\left|D^{\beta }r\left(\psi ;x\right)\right| & \leq \int_{R^{q}}H_{\beta ,\psi }\left(y\right)\;dy<\infty . \end{array}$$

□

**Lemma 7** 
(Expectation and weak differentiation of conditional fields). *Let $O\subset R^{p}$ be open, and let G:Ω×O→R be jointly measurable. Suppose that, for some integer k≥0, $G\left(\omega ,\cdot \right)\in W_{\mathrm{loc}}^{k,1}\left(O\right)$ for almost every ω, and that for every compact C⋐O,*

$${E∥G∥}_{W^{k,1}\left(C\right)}<\infty .$$

*If EG(u)=g(u) for Lebesgue-almost every u∈O, then $g\in W_{\mathrm{loc}}^{k,1}\left(O\right)$ and, for every multi-index **β** with |β|≤k,*

(169) $$ED^{\beta }G\left(u\right)=D^{\beta }g\left(u\right)$$

*for Lebesgue-almost every u. If both sides admit continuous versions on a compact set, the equality holds pointwise there after choosing those versions.*

**Proof.**  Fix $\varphi \in C_{c}^{\infty }\left(O\right)$ and a multi-index β with |β|≤k. The local $W^{k,1}$-integrability permits Fubini’s theorem, and the definition of weak derivative gives

$$\begin{array}{cc} \int_{O}E\left[D^{\beta }G\left(u\right)\right]\varphi \left(u\right)\;du & =E\int_{O}D^{\beta }G\left(u\right)\varphi \left(u\right)\;du\\ & ={\left(-1\right)}^{\left|\beta \right|}E\int_{O}G\left(u\right)D^{\beta }\varphi \left(u\right)\;du\\ & ={\left(-1\right)}^{\left|\beta \right|}\int_{O}g\left(u\right)D^{\beta }\varphi \left(u\right)\;du. \end{array}$$

Thus, $E[D^{\beta }G]$ is the weak derivative $D^{\beta }g$, proving (169). If continuous representatives exist, equality almost everywhere implies equality everywhere on the compact set. □

Applied to the conditional moment-density field $G=g_{i,\psi }$, the tower property gives $Eg_{i,\psi }=r\left(\psi ;\cdot \right)$; hence,

$$ED^{\beta }g_{i,\psi }=D^{\beta }r\left(\psi ;\cdot \right)$$

whenever the local Sobolev integrability in (P.1) applies. The same argument with $G=f_{i}$ yields

$$ED^{\beta }f_{i}=D^{\beta }f_{X}.$$

These identities justify the centred derivative fields used in the projective and predictable components below.

**Lemma 8** 
(Weak integration by parts for a scaled derivative kernel). *Let $K\in W^{|s|,1}\left(R^{p}\right)\cap W^{|s|,\infty }\left(R^{p}\right)$ have compact support, and let $a\in W_{\mathrm{loc}}^{|s|,1}\left(R^{p}\right)$. Suppose that, for the fixed x and b>0, the function u↦a(x−bu) and all weak derivatives that occur below are integrable on a compact neighbourhood of supp(K). Then,*

(170) $$\int_{R^{p}}D^{s}K\left(u\right)a\left(x-bu\right)\;du=b^{\left|s\right|}\int_{R^{p}}K\left(u\right)D^{s}a\left(x-bu\right)\;du.$$

**Proof.**  We first treat the smooth case. For a derivative with respect to the *j*-th coordinate, classical integration by parts yields

$$\begin{array}{cc} \int \partial_{u_{j}}K\left(u\right)a\left(x-bu\right)\;du & =-\int K\left(u\right)\partial_{u_{j}}a\left(x-bu\right)\;du\\ & =b\int K\left(u\right)\partial_{x_{j}}a\left(x-bu\right)\;du. \end{array}$$

The boundary term vanishes because *K* has compact support. Iterating over the coordinates according to the multi-index s yields (170).

For weakly differentiable functions, use the Sobolev integration-by-parts identity directly. If $u\in W_{0}^{1,\infty }\left(C\right)$ and $v\in W^{1,1}\left(C\right)$ on a bounded Lipschitz set *C*, then

$$\int_{C}\partial_{j}u\;v=-\int_{C}u\;\partial_{j}v.$$

Because *K* is compactly supported, we may choose *C* so that every derivative of *K* occurring in the iteration has zero trace on ∂C. The Sobolev chain rule for the affine map u↦x−bu gives

$$\partial_{u_{j}}a\left(x-bu\right)=-b\;\partial_{x_{j}}a\left(x-bu\right)\;a.e.$$

Applying the $W^{1,\infty }$–$W^{1,1}$ integration-by-parts identity successively according to the multi-index s transfers all weak derivatives from *K* to a(x−b·). Each transfer contributes one factor *b* and one minus sign from the chain rule, which cancels the minus sign from integration by parts. After |s| iterations, one obtains exactly (170). Under (K.1), the required $W^{|s|,\infty }$-regularity of the kernel derivatives is part of the standing kernel regularity. □

**Lemma 9** 
(Bilateral martingale projection decomposition). *Let H be a separable Hilbert space, let $U\in L^{2}\left(\Omega ;H\right)$, and put*

$$P_{k}U=E\left(U|G_{k}\right)-E\left(U|G_{k-1}\right).$$

*Assume*

$$E\left(U|G_{-\infty }\right)=0\;\mathrm{and}\;E\left(U|G_{\infty }\right)=U\;\mathrm{in}\;L^{2}\left(\Omega ;H\right).$$

*Then,*

(171) $$U=\sum_{k\in Z}P_{k}U\;\mathrm{in}\;L^{2}\left(\Omega ;H\right),$$

*the summands are pairwise orthogonal in $L^{2}\left(\Omega ;H\right)$, and*

(172) $${∥U∥}_{L^{2}\left(\Omega ;H\right)}^{2}=\sum_{k\in Z}{∥P_{k}U∥}_{L^{2}\left(\Omega ;H\right)}^{2}.$$

**Proof.**  For integers m≤n, telescoping gives

$$\sum_{k=m}^{n}P_{k}U=E\left(U|G_{n}\right)-E\left(U|G_{m-1}\right).$$

The Hilbert-valued upward martingale convergence theorem gives

$$E\left(U|G_{n}\right)⟶E\left(U|G_{\infty }\right)=U$$

in $L^{2}\left(\Omega ;H\right)$ as n→∞. The reverse martingale convergence theorem gives

$$E\left(U|G_{m-1}\right)⟶E\left(U|G_{-\infty }\right)=0$$

in $L^{2}\left(\Omega ;H\right)$ as m→−∞. Hence, the rectangular partial sums converge to *U* in $L^{2}\left(\Omega ;H\right)$, which establishes (171).

If k<ℓ, then $P_{k}U$ is $G_{k}$-measurable and

$$E(P_{\ell }U|G_{\ell -1})=0.$$

Since $G_{k}\subset G_{\ell -1}$,

$$\begin{array}{cc} E{\langle P_{k}U,P_{\ell }U\rangle }_{H} & =E{\langle P_{k}U,E\left(P_{\ell }U|G_{\ell -1}\right)\rangle }_{H}=0. \end{array}$$

Applying Pythagoras’ identity to finite partial sums and then passing to the $L^{2}$-limit yields (172). □

**Lemma 10** 
(Stationarity of conditional fields). *Suppose the process is realized by a measure-preserving shift T:Ω→Ω such that $Z_{i}=Z_{0}\circ T^{i}$ and $G_{i}=T^{-i}G_{0}$. Conditional densities and conditional moment densities may be selected so that*

$$f_{i}\left(\omega ,u\right)=f_{0}\left(T^{i}\omega ,u\right),\;g_{i,\psi }\left(\omega ,u\right)=g_{0,\psi }\left(T^{i}\omega ,u\right)$$

*for Lebesgue-almost every u, outside one invariant null set. Consequently, whenever the displayed random fields are integrable,*

$$\frac{1}{n}\sum_{i=1}^{n}\underset{u\in J}{\sup}|D^{s}g_{i,\psi }\left(u\right)|⟶E\underset{u\in J}{\sup}\left|D^{s}g_{0,\psi }\left(u\right)\right|$$

*almost surely by the ergodic theorem.*

**Proof.**  Work on the two-sided shift representation, for which *T* may be taken invertible. Let *E* denote the state space of $Z_{0}$, and let

$$P_{0}\left(\omega ,B\right)=P\left(Z_{0}\in B|G_{-1}\right)\left(\omega \right),\;B\in B\left(E\right),$$

be a regular conditional law. Define

$$P_{i}\left(\omega ,B\right)=P_{0}\left(T^{i}\omega ,B\right).$$

Because

$$Z_{i}=Z_{0}\circ T^{i},\;G_{i-1}=T^{-i}G_{-1},$$

the kernel $P_{i}$ is a version of the conditional law of $Z_{i}$ given $G_{i-1}$. Indeed, if *H* is bounded and $G_{i-1}$-measurable, then $H=H_{0}\circ T^{i}$ for a bounded $G_{-1}$-measurable $H_{0}$, and invariance of P under *T* yields

$$\begin{array}{cc} E\left[H1_{\left\{Z_{i}\in B\right\}}\right] & =E\left[H_{0}1_{\left\{Z_{0}\in B\right\}}\right]\\ & =E\left[H_{0}P_{0}\left(\cdot ,B\right)\right]\\ & =E\left[HP_{0}\left(T^{i}\cdot ,B\right)\right]. \end{array}$$

Hence, the regular conditional kernels may be selected to shift covariantly.

Apply this construction to the *X*-marginal and to the joint $\left(W_{i}\left(\psi \right),X_{i}\right)$-law. Jointly measurable Radon–Nikodym derivatives can then be selected so that, outside one invariant null set,

$$f_{i}\left(\omega ,u\right)=f_{0}\left(T^{i}\omega ,u\right),\;g_{i,\psi }\left(\omega ,u\right)=g_{0,\psi }\left(T^{i}\omega ,u\right)$$

for Lebesgue-almost every u. The final assertion is Birkhoff’s ergodic theorem applied to

$$\omega ⟼\underset{u\in J}{\sup}\left|D^{s}g_{0,\psi }\left(\omega ,u\right)\right|,$$

which is integrable by hypothesis. □

### 7.2. Auxiliary Probability Inequalities and Projective Bounds

We begin with the conditional Bernstein inequality in precisely the normalization required for the localized martingale increments.

**Lemma 11** 
(Conditional Bernstein inequality). *Let ${\left(Z_{i},F_{i}\right)}_{1\leq i\leq n}$ be real-valued martingale differences. Suppose that there exist non-negative $F_{i-1}$-measurable random variables $v_{i}^{2}$ and a deterministic constant c>0, such that, for every integer ν≥2,*

(173) $$E\left(|Z_{i}{|}^{\nu }|F_{i-1}\right)\leq \frac{\nu !}{2}c^{\nu -2}v_{i}^{2}\;a.s.$$

*Assume that*

$$\sum_{i=1}^{n}v_{i}^{2}\leq V_{n}\;a.s.$$

*for a deterministic $V_{n}<\infty$. Then, for every t>0,*

(174) $$P\left(\left|\sum_{i=1}^{n}Z_{i}\right|\geq t\right)\leq 2\exp\left\{-\frac{t^{2}}{2(V_{n}+ct)}\right\}.$$

**Proof.**  Fix $0<\lambda <c^{-1}$. Since $E(Z_{i}|F_{i-1})=0$, expansion of the conditional moment-generating function gives

$$\begin{array}{cc} E\left(e^{\lambda Z_{i}}|F_{i-1}\right) & =1+\sum_{\nu =2}^{\infty }\frac{\lambda^{\nu }}{\nu !}E\left(Z_{i}^{\nu }|F_{i-1}\right)\\ & \leq 1+\sum_{\nu =2}^{\infty }\frac{\lambda^{\nu }}{\nu !}E\left(|Z_{i}{|}^{\nu }|F_{i-1}\right)\\ & \leq 1+\frac{\lambda^{2}v_{i}^{2}}{2}\sum_{\nu =2}^{\infty }{\left(\lambda c\right)}^{\nu -2}\\ & =1+\frac{\lambda^{2}v_{i}^{2}}{2(1-\lambda c)}\\ & \leq \exp\left\{\frac{\lambda^{2}v_{i}^{2}}{2(1-\lambda c)}\right\}. \end{array}$$

Iterating conditional expectations therefore yields

$$E\exp\left\{\lambda \sum_{i=1}^{n}Z_{i}\right\}\leq \exp\left\{\frac{\lambda^{2}V_{n}}{2(1-\lambda c)}\right\}.$$

By Chernoff’s inequality,

$$\begin{array}{cc} P\left(\sum_{i=1}^{n}Z_{i}\geq t\right) & \leq \exp\left\{-\lambda t+\frac{\lambda^{2}V_{n}}{2(1-\lambda c)}\right\}. \end{array}$$

Choose

$$\lambda =\frac{t}{V_{n}+ct}.$$

Then, $0<\lambda <c^{-1}$, and direct substitution gives

$$-\lambda t+\frac{\lambda^{2}V_{n}}{2(1-\lambda c)}=-\frac{t^{2}}{2(V_{n}+ct)}.$$

Applying the same argument to $-Z_{i}$ and adding the two one-sided probabilities proves (174). This completes the exponential-moment argument and, hence, the conditional Bernstein inequality; compare also [80]. □

The following projective estimates identify a verifiable route from the absolute projection summability condition of Section 3 to the linear $L^{2}$ growth required for predictable partial sums. The Hilbert-valued formulation is deliberate: once the random field is controlled in a Sobolev space $H^{m}\left(J\right)$, m>p/2, the corresponding uniform bound follows directly from the continuous Sobolev embedding.

**Lemma 12** 
(Projective partial-sum bound for the density component). *Let H be a separable Hilbert space, and let ${\left(U_{i}^{f}\right)}_{i\in Z}$ be a strictly stationary, centred, H-valued process adapted to $\left(G_{i}\right)$. Assume regularity of the filtration in the sense that*

$$U_{i}^{f}=\sum_{k\in Z}P_{k}U_{i}^{f}\;\mathrm{in}\;L^{2}\left(\Omega ;H\right),$$

*and assume that*

(175) $$\sum_{j\in Z}{∥P_{0}U_{j}^{f}∥}_{L^{2}\left(\Omega ;H\right)}<\infty .$$

*Then,*

(176) $$E{∥\sum_{i=1}^{n}U_{i}^{f}∥}_{H}^{2}\leq n{\left(\sum_{j\in Z}{∥P_{0}U_{j}^{f}∥}_{L^{2}\left(\Omega ;H\right)}\right)}^{2}.$$

*In particular, the left-hand side is O(n).*

**Proof.**  By the assumed $L^{2}\left(\Omega ;H\right)$ decomposition,

$$\sum_{i=1}^{n}U_{i}^{f}=\sum_{k\in Z}\sum_{i=1}^{n}P_{k}U_{i}^{f}.$$

For distinct *k*, the Hilbert-valued random variables $\sum_{i}P_{k}U_{i}^{f}$ are orthogonal in $L^{2}\left(\Omega ;H\right)$. Indeed, if k<ℓ, then $\sum_{i}P_{k}U_{i}^{f}$ is $G_{k}$-measurable, whereas

$$E\left(\sum_{i}P_{\ell }U_{i}^{f}|G_{\ell -1}\right)=0,$$

and $G_{k}\subset G_{\ell -1}$. Hence,

$$\begin{array}{cc} E{∥\sum_{i=1}^{n}U_{i}^{f}∥}_{H}^{2} & =\sum_{k\in Z}E{∥\sum_{i=1}^{n}P_{k}U_{i}^{f}∥}_{H}^{2}\\ & \leq \sum_{k\in Z}{\left(\sum_{i=1}^{n}{∥P_{k}U_{i}^{f}∥}_{L^{2}\left(\Omega ;H\right)}\right)}^{2}. \end{array}$$

By stationarity,

$${∥P_{k}U_{i}^{f}∥}_{L^{2}\left(\Omega ;H\right)}={∥P_{0}U_{i-k}^{f}∥}_{L^{2}\left(\Omega ;H\right)}.$$

Put

$$a_{j}={∥P_{0}U_{j}^{f}∥}_{L^{2}\left(\Omega ;H\right)},\;e_{i}=1_{\left\{1,\ldots ,n\right\}}\left(i\right).$$

The preceding bound equals

$$\sum_{k\in Z}{\left\{\sum_{i\in Z}e_{i}a_{i-k}\right\}}^{2}={∥e*a∥}_{\ell^{2}\left(Z\right)}^{2}.$$

Young’s convolution inequality gives

$${∥e*a∥}_{\ell^{2}}\leq {∥e∥}_{\ell^{2}}{∥a∥}_{\ell^{1}}=n^{1/2}\sum_{j\in Z}a_{j}.$$

Squaring proves (176). □

**Lemma 13** 
(Projective partial-sum bound for the IPW component). *Under the assumptions of Lemma 12, with $U_{i}^{f}$ replaced by the centred conditional derivative-density process*

$$U_{i}^{r}=D^{s}g_{i,\psi }-D^{s}r\left(\psi ;\cdot \right),$$

*one has*

(177) $$E{∥\sum_{i=1}^{n}U_{i}^{r}∥}_{H}^{2}=O\left(n\right).$$

*If $H=H^{m}\left(J\right)$ with m>p/2, then Sobolev embedding implies that*

$$E\underset{u\in J}{\sup}{\left|\sum_{i=1}^{n}U_{i}^{r}\left(u\right)\right|}^{2}=O\left(n\right).$$

**Proof.**  The Hilbert-space estimate follows by the same orthogonal-projection and Young-convolution argument as in Lemma 12. If m>p/2, the continuous embedding $H^{m}\left(J\right)↪C\left(J\right)$ gives a finite constant $C_{\mathrm{emb}}$ such that

$$\underset{u\in J}{\sup}\left|v\left(u\right)\right|\leq C_{\mathrm{emb}}{∥v∥}_{H^{m}\left(J\right)}.$$

Apply this inequality to $v=\sum_{i=1}^{n}U_{i}^{r}$, and then use (177). □

### 7.3. Verifiable Sufficient Conditions for the Abstract Dependence Modules

The dependence hypotheses in Section 3 are formulated modularly so that the main theorems are not tied to a single dependence coefficient. The results in this subsection supply concrete sufficient criteria for several of those modules. They are verification devices only: none is added to the standing assumptions unless explicitly invoked in an application.

**Lemma 14** 
(Entropy of a Lipschitz translation class). *Let $\kappa :R^{p}\to R$ be bounded and Lipschitz with constant $L_{\kappa }$, and let $I\subset R^{p}$ be compact. For b>0, define*

$$K_{b}=\left\{u\mapsto \kappa \left(\frac{x-u}{b}\right):x\in I\right\}.$$

*Then, for 0<ε≤1,*

$$N\left({\varepsilon ∥\kappa ∥}_{\infty },K_{b},{∥\cdot ∥}_{\infty }\right)\leq C_{I,p}{\left[1+\frac{L_{\kappa }\mathrm{diam}\left(I\right)}{{\varepsilon b∥\kappa ∥}_{\infty }}\right]}^{p}.$$

*Thus,* (U.3) *holds for $\kappa =D^{s}K$.*

**Proof.**  For x,y∈I,

$$\begin{array}{cc} & \underset{u\in R^{p}}{\sup}\left|\kappa \left(\frac{x-u}{b}\right)-\kappa \left(\frac{y-u}{b}\right)\right|\\ & \leq \frac{L_{\kappa }}{b}∥x-y∥. \end{array}$$

Hence, a Euclidean

$$\rho =\frac{{\varepsilon b∥\kappa ∥}_{\infty }}{L_{\kappa }}$$

net of I induces an ${\varepsilon ∥\kappa ∥}_{\infty }$-net of $K_{b}$ in sup-norm. A bounded subset of $R^{p}$ can be covered by at most

$$C_{I,p}{\left(1+\frac{\mathrm{diam}(I)}{\rho }\right)}^{p}$$

Euclidean balls of radius ρ. Substitute the value of ρ. □

**Lemma 15** 
(Uniform ergodic theorem for Hölder random fields). *Let T be an ergodic measure-preserving transformation, and let*

$$Q_{i}\left(\omega ,u\right)=Q_{0}\left(T^{i}\omega ,u\right)$$

*be a stationary random field on a compact set J. Suppose that there exist α∈(0,1] and integrable random variables $M_{0},L_{0}$ such that*

$$\underset{u\in J}{\sup}\left|Q_{0}\left(u\right)\right|\leq M_{0}$$

*and*

$$|Q_{0}\left(u\right)-Q_{0}\left(v\right)|\leq L_{0}{∥u-v∥}^{\alpha }\;\left(u,v\in J\right).$$

*Then,*

(178) $$\underset{u\in J}{\sup}\left|\frac{1}{n}\sum_{i=1}^{n}Q_{i}\left(u\right)-EQ_{0}\left(u\right)\right|⟶0\;a.s.$$

**Proof.**  Fix m≥1 and choose a finite $m^{-1}$-net $\left\{u_{m,1},\ldots ,u_{m,N_{m}}\right\}$ of J. For every net point, Birkhoff’s theorem gives

$$\frac{1}{n}\sum_{i=1}^{n}Q_{i}\left(u_{m,k}\right)⟶EQ_{0}\left(u_{m,k}\right)\;a.s.$$

Because the union over m,k is countable, one null set can be chosen so that all of these convergences hold simultaneously.

For arbitrary u∈J, choose a nearest net point $u_{m,k(u)}$. Then,

$$\begin{array}{cc} & \left|\frac{1}{n}\sum_{i=1}^{n}Q_{i}\left(u\right)-EQ_{0}\left(u\right)\right|\\ & \leq \left|\frac{1}{n}\sum_{i=1}^{n}Q_{i}\left(u_{m,k(u)}\right)-EQ_{0}\left(u_{m,k(u)}\right)\right|\\ & \;+\frac{1}{n}\sum_{i=1}^{n}L_{0}\circ T^{i}{∥u-u_{m,k(u)}∥}^{\alpha }\\ & \;+EL_{0}{∥u-u_{m,k(u)}∥}^{\alpha }. \end{array}$$

Taking the supremum over u gives

$$\begin{array}{cc} & \underset{u\in J}{\sup}\left|\frac{1}{n}\sum_{i=1}^{n}Q_{i}\left(u\right)-EQ_{0}\left(u\right)\right|\\ & \leq \max_{1\leq k\leq N_{m}}\left|\frac{1}{n}\sum_{i=1}^{n}Q_{i}\left(u_{m,k}\right)-EQ_{0}\left(u_{m,k}\right)\right|\\ & \;+m^{-\alpha }\left[\frac{1}{n}\sum_{i=1}^{n}L_{0}\circ T^{i}+EL_{0}\right]. \end{array}$$

For fixed *m*, let n→∞. The first term tends to zero, and the ergodic theorem sends the bracket to $2EL_{0}$. Therefore,

$$\underset{n\to \infty }{lim sup}\underset{u\in J}{\sup}\left|\frac{1}{n}\sum_{i=1}^{n}Q_{i}\left(u\right)-EQ_{0}\left(u\right)\right|\leq 2m^{-\alpha }EL_{0}$$

almost surely. Let m→∞. □

**Corollary 5** (A sufficient condition for (C.1)). *Suppose*

$$q_{i,\psi }\left(\omega ,u\right)=q_{0,\psi }\left(T^{i}\omega ,u\right)$$

*and the hypotheses of Lemma 15 hold on $N_{x}$. Then,*

$$\underset{u\in N_{x}}{\sup}\left|\frac{1}{n}\sum_{i=1}^{n}q_{i,\psi }\left(u\right)-q_{\psi }\left(u\right)\right|⟶0\;a.s.,$$

*where*

$$q_{\psi }\left(u\right)=Eq_{0,\psi }\left(u\right).$$

*Thus,* (C.1) *holds, in fact with almost-sure convergence.*

**Lemma 16**  (A martingale–coboundary sufficient condition for (P.3)). *Let $H=H^{m}\left(J\right)$ with m>p/2. Suppose that*

$$G_{i}=D_{i}+H_{i}-H_{i+1},$$

*where $\left(D_{i},G_{i}\right)$ is a stationary H-valued martingale difference sequence and $\left(H_{i}\right)$ is stationary. Assume that, for some δ>0,*

$$E∥D_{0}{∥}_{H}^{2+\delta }<\infty ,\;E{∥H_{0}∥}_{H}^{2+\delta }<\infty .$$

*Then,*

$${∥\sum_{i=1}^{n}G_{i}∥}_{H}=O_{a.s.}\left\{{\left(n\log n\right)}^{1/2}\right\}.$$

*By Sobolev embedding,*

$$\underset{u\in J}{\sup}\left|\sum_{i=1}^{n}G_{i}\left(u\right)\right|=O_{a.s.}\left\{{\left(n\log n\right)}^{1/2}\right\},$$

*so* (P.3) *follows.*

**Proof.**  Let

$$M_{n}=\sum_{i=1}^{n}D_{i}.$$

The Hilbert-space Burkholder inequality gives a constant $C_{\delta }<\infty$ such that

$$E\max_{1\leq k\leq N}{∥M_{k}∥}_{H}^{2+\delta }\leq C_{\delta }E{\left(\sum_{i=1}^{N}{∥D_{i}∥}_{H}^{2}\right)}^{1+\delta /2}.$$

By convexity,

$${\left(\sum_{i=1}^{N}{∥D_{i}∥}_{H}^{2}\right)}^{1+\delta /2}\leq N^{\delta /2}\sum_{i=1}^{N}{∥D_{i}∥}_{H}^{2+\delta }.$$

Stationarity therefore yields

$$E\max_{1\leq k\leq N}∥M_{k}{∥}_{H}^{2+\delta }\leq C_{\delta }N^{1+\delta /2}E{∥D_{0}∥}_{H}^{2+\delta }.$$

Take $N=2^{r}$ and threshold

$$\lambda_{r}=A{\left(2^{r}r\right)}^{1/2}.$$

Markov’s inequality gives

$$\begin{array}{cc} P\left(\max_{1\leq k\leq 2^{r}}{∥M_{k}∥}_{H}>\lambda_{r}\right) & \leq \frac{C_{\delta }2^{r(1+\delta /2)}}{A^{2+\delta }2^{r(1+\delta /2)}r^{1+\delta /2}}\\ & =\frac{C_{\delta }}{A^{2+\delta }r^{1+\delta /2}}. \end{array}$$

The series over *r* converges. Borel–Cantelli gives

$$\max_{1\leq k\leq 2^{r}}{∥M_{k}∥}_{H}=O_{a.s.}\left\{{\left(2^{r}r\right)}^{1/2}\right\}.$$

For $2^{r-1}<n\leq 2^{r}$, this implies that

$$∥M_{n}{∥}_{H}=O_{a.s.}\left\{{\left(n\log n\right)}^{1/2}\right\}.$$

The coboundary sum telescopes:

$$\sum_{i=1}^{n}\left(H_{i}-H_{i+1}\right)=H_{1}-H_{n+1}.$$

For every ε>0,

$$\begin{array}{cc} \sum_{n=2}^{\infty }P\left(∥H_{n+1}{∥}_{H}>\varepsilon {\left(n\log n\right)}^{1/2}\right) & \leq \frac{E∥H_{0}{∥}_{H}^{2+\delta }}{\varepsilon^{2+\delta }}\sum_{n=2}^{\infty }\frac{1}{{\left(n\log n\right)}^{1+\delta /2}}<\infty . \end{array}$$

Borel–Cantelli gives

$$∥H_{n+1}{∥}_{H}=o_{a.s.}\left\{{\left(n\log n\right)}^{1/2}\right\}.$$

Combining the martingale and coboundary bounds proves the Hilbert-space assertion. The continuous embedding $H^{m}\left(J\right)↪C\left(J\right)$ proves the sup-norm bound. □

### 7.4. Conditional Convolution Identities

The following convolution identity is the basic bridge between the predictable conditional field and the deterministic smoothing operator; it is used repeatedly in both the uniform and pointwise arguments.

**Lemma 17** 
(Predictable convolution representation). *Assume* (K.1) *and* (P.1)*. Then, for every x∈I and all sufficiently large n,*

(179) $$\frac{A_{i,n}\left(x\right)}{h_{n}^{1+|s|/p}}=\int_{R^{p}}K\left(v\right)D^{s}g_{i,\psi }\left(x-b_{n}v\right)\;dv.$$

*Moreover,*

(180) $$P_{n,s}\left(x\right)=\frac{1}{n}\int_{R^{p}}K\left(v\right)\sum_{i=1}^{n}G_{i,\psi }^{\left(s\right)}\left(x-b_{n}v\right)\;dv.$$

**Proof.**  Conditional disintegration gives

$$\begin{array}{cc} A_{i,n}\left(x\right) & =\int_{R^{p}}\kappa_{s}\left(\frac{x-u}{b_{n}}\right)g_{i,\psi }\left(u\right)\;du. \end{array}$$

With

$$u=x-b_{n}v,\;du=b_{n}^{p}\;dv=h_{n}\;dv,$$

this becomes

$$A_{i,n}\left(x\right)=h_{n}\int_{R^{p}}D^{s}K\left(v\right)g_{i,\psi }\left(x-b_{n}v\right)\;dv.$$

Repeated integration by parts, with the boundary terms equal to zero by (K.1), gives

$$\int D^{s}K\left(v\right)g_{i,\psi }\left(x-b_{n}v\right)\;dv=b_{n}^{\left|s\right|}\int K\left(v\right)D^{s}g_{i,\psi }\left(x-b_{n}v\right)\;dv.$$

Since $h_{n}b_{n}^{\left|s\right|}=h_{n}^{1+|s|/p}$, (179) follows.

Taking the expectations in (179) and applying Lemma 7 to the conditional moment-density field gives

$$ED^{s}g_{i,\psi }\left(u\right)=D^{s}r\left(\psi ;u\right)$$

for the selected versions. Hence,

$$\frac{EA_{i,n}\left(x\right)}{h_{n}^{1+|s|/p}}=\int K\left(v\right)D^{s}r\left(\psi ;x-b_{n}v\right)\;dv.$$

Subtracting, summing over *i*, and dividing by *n* proves (180). □

**Lemma 18** 
(Conditional moments of localized martingale increments). *Assume* (K.1), (U.1)*, and* (U.2)*. There are finite constants $\bar{v}>0$ and $\bar{c}>0$, independent of i,n,x, such that, for every integer ν≥2,*

(181) $$E\left[|\Delta_{i,n}{\left(x\right)|}^{\nu }|G_{i-1}\right]\leq \frac{\nu !}{2}{\bar{c}}^{\nu -2}{\bar{v}}^{2}h_{n}\;a.s.$$

**Proof.**  Conditioning first on $\sigma \left(X_{i}\right)\vee G_{i-1}$, then using (U.2), yields

$$\begin{array}{cc} & E\left[|\Theta_{i,n}{\left(x\right)|}^{\nu }|G_{i-1}\right]\\ & =\int_{J}E\left[|W_{i}{\left(\psi \right)|}^{\nu }|X_{i}=u,G_{i-1}\right]{\left|\kappa_{s}\left(\frac{x-u}{b_{n}}\right)\right|}^{\nu }f_{i}\left(u\right)\;du\\ & \leq \frac{\nu !}{2}v_{\psi }^{2}c_{\psi }^{\nu -2}C_{f}\int {\left|\kappa_{s}\left(\frac{x-u}{b_{n}}\right)\right|}^{\nu }\;du. \end{array}$$

Because

$$|\kappa_{s}{\left(z\right)|}^{\nu }\leq ∥\kappa_{s}{∥}_{\infty }^{\nu -2}{\left|\kappa_{s}\left(z\right)\right|}^{2}$$

and $du=h_{n}\;dz$,

$$\begin{array}{cc} \int {\left|\kappa_{s}\left(\frac{x-u}{b_{n}}\right)\right|}^{\nu }\;du & \leq h_{n}{∥\kappa_{s}∥}_{\infty }^{\nu -2}R_{s}\left(K\right). \end{array}$$

Hence,

$$E\left[|\Theta_{i,n}{\left(x\right)|}^{\nu }|G_{i-1}\right]\leq \frac{\nu !}{2}{\tilde{c}}^{\;\nu -2}{\tilde{v}}^{\;2}h_{n}$$

for suitable deterministic constants.

For every integrable random variable *U* and sigma-field H,

$${\left|U-E\left(U|H\right)\right|}^{\nu }\leq 2^{\nu -1}\left[{\left|U\right|}^{\nu }+{\left|E\left(U|H\right)\right|}^{\nu }\right].$$

Conditional Jensen gives

$${\left|E\left(U|H\right)\right|}^{\nu }\leq {E(|U|}^{\nu }|H).$$

After conditioning on H, the preceding two inequalities imply

$$E\left[{\left|U-E\left(U|H\right)\right|}^{\nu }|H\right]\leq 2^{\nu }E\left({\left|U\right|}^{\nu }|H\right).$$

Apply this with $U=\Theta_{i,n}\left(x\right)$ and $H=G_{i-1}$, and absorb the factor $2^{\nu }$ into new constants $\bar{c},\bar{v}$. This proves (181). □

### 7.5. Bias Calculations

We now identify the deterministic smoothing bias. The argument first derives an exact expectation-convolution identity and then performs a uniform multivariate Taylor expansion to isolate the leading kernel-moment term.

**Lemma 19** 
(Exact expectation and uniform Taylor expansion). *Assume* (S.2)–(S.3)*,* (K.1)–(K.2)*, and* (R.1)–(R.2)*. Then,*

(182) $$ED^{s}r_{n}^{\;\mathrm{IPW}}\left(\psi ;x,h_{n}\right)=\int_{R^{p}}K\left(v\right)D^{s}r\left(\psi ;x-b_{n}v\right)\;dv$$

*for every x∈I and all sufficiently large n. Furthermore,*

(183) $$ED^{s}r_{n}^{\;\mathrm{IPW}}\left(\psi ;x,h_{n}\right)-D^{s}r\left(\psi ;x\right)=h_{n}^{\ell /p}B_{s,\ell }\left(x\right)+\rho_{n}\left(x\right),$$

*where*

(184) $$\underset{x\in I}{\sup}\left|\rho_{n}\left(x\right)\right|=o\left(h_{n}^{\ell /p}\right).$$

**Proof.**  By stationarity,

$$\begin{array}{cc} ED^{s}r_{n}^{\;\mathrm{IPW}}\left(\psi ;x,h_{n}\right) & =\frac{1}{h_{n}^{1+|s|/p}}E\left[W_{0}\left(\psi \right)\kappa_{s}\left(\frac{x-X_{0}}{b_{n}}\right)\right]. \end{array}$$

Sequential MAR gives

$$E\left[W_{0}\left(\psi \right)|X_{0},Y_{0},G_{-1}\right]=\psi \left(Y_{0}\right)\;a.s.$$

Therefore, by the tower property,

$$\begin{array}{cc} & E\left[W_{0}\left(\psi \right)\kappa_{s}\left(\frac{x-X_{0}}{b_{n}}\right)\right]\\ & =E\left[\psi \left(Y_{0}\right)\kappa_{s}\left(\frac{x-X_{0}}{b_{n}}\right)\right]\\ & =\int_{R^{p}}\kappa_{s}\left(\frac{x-u}{b_{n}}\right)r\left(\psi ;u\right)\;du. \end{array}$$

The integrability required for Fubini follows from $E|\psi (Y_{0})|<\infty$, compact support of the kernel, and the local boundedness assumptions.

Set

$$u=x-b_{n}v,\;du=h_{n}\;dv.$$

Then,

$$\begin{array}{cc} ED^{s}r_{n}^{\;\mathrm{IPW}}\left(\psi ;x,h_{n}\right) & =b_{n}^{-|s|}\int D^{s}K\left(v\right)r\left(\psi ;x-b_{n}v\right)\;dv. \end{array}$$

Repeated integration by parts gives

$$b_{n}^{-|s|}\int D^{s}K\left(v\right)r\left(\psi ;x-b_{n}v\right)\;dv=\int K\left(v\right)D^{s}r\left(\psi ;x-b_{n}v\right)\;dv.$$

There is no residual sign because differentiation of $r(x-b_{n}v)$ with respect to v contributes a factor $-b_{n}$, which cancels the sign introduced by integration by parts. This proves (182).

Let

$$g\left(x\right)=D^{s}r\left(\psi ;x\right).$$

For v in the compact support of *K*, the multivariate Taylor formula with integral remainder yields

$$\begin{array}{cc} g(x-b_{n}v) & =g\left(x\right)+\sum_{1\leq |\alpha |\leq \ell -1}\frac{{\left(-b_{n}\right)}^{\left|\alpha \right|}}{\alpha !}v^{\alpha }D^{\alpha }g\left(x\right)\\ & \;+{\left(-b_{n}\right)}^{\ell }\sum_{|\alpha |=\ell }\frac{v^{\alpha }}{\alpha !}\ell \int_{0}^{1}{\left(1-t\right)}^{\ell -1}D^{\alpha }g\left(x-tb_{n}v\right)\;dt. \end{array}$$

After multiplication by K(v) and integration, all terms of total order 1,…,ℓ−1 vanish by (K.2). Add and subtract $D^{\alpha }g\left(x\right)$ in the remainder. Since

$$\ell \int_{0}^{1}{\left(1-t\right)}^{\ell -1}\;dt=1,$$

the non-remainder part equals

$${\left(-1\right)}^{\ell }b_{n}^{\ell }\sum_{|\alpha |=\ell }\frac{\mu_{\alpha }\left(K\right)}{\alpha !}D^{\alpha }g\left(x\right)=h_{n}^{\ell /p}B_{s,\ell }\left(x\right).$$

The residual term is bounded by

$$\begin{array}{cc} |\rho_{n}\left(x\right)| & \leq b_{n}^{\ell }\sum_{|\alpha |=\ell }\frac{1}{\alpha !}\int \left|v^{\alpha }K\left(v\right)\right|\\ & \;\times \ell \int_{0}^{1}{\left(1-t\right)}^{\ell -1}\left|D^{\alpha }g\left(x-tb_{n}v\right)-D^{\alpha }g\left(x\right)\right|dt\;dv. \end{array}$$

The derivatives of total order |s|+ℓ are uniformly continuous on the compact neighbourhood J. Hence, the inner difference tends to zero uniformly in x∈I, t∈[0,1], and v in the support of *K*. Dominated convergence applies because

$$\int ∥v{∥}^{\ell }\left|K\left(v\right)\right|\;dv<\infty .$$

Therefore,

$$\underset{x\in I}{\sup}\left|\rho_{n}\left(x\right)\right|=o\left(b_{n}^{\ell }\right)=o\left(h_{n}^{\ell /p}\right),$$

which proves the lemma. □

As an immediate coarser consequence of the preceding expansion,

(185) $$\underset{x\in I}{\sup}\left|ED^{s}r_{n}^{\;\mathrm{IPW}}\left(\psi ;x,h_{n}\right)-D^{s}r\left(\psi ;x\right)\right|=O\left(h_{n}^{\ell /p}\right).$$

**Proof of Proposition 2.**  The asserted expansion and uniform remainder are precisely (183) and (184). The $O(h_{n}^{\ell /p})$ conclusion follows because $B_{s,\ell }$ is continuous, and hence bounded, on the compact set I. □

### 7.6. Uniform Almost-Sure Stochastic Bounds

We next establish the uniform almost-sure stochastic rate for the centred oracle estimator. The proof combines a Bernstein bound on an increasingly fine deterministic net with a Lipschitz oscillation argument between net points.

**Proposition 9.** 
*Under the assumptions of Theorem 1,*

$$\underset{x\in I}{\sup}\left|M_{n,s}\left(x\right)\right|=O_{a.s.}\left[{\left\{\frac{\log n}{nh_{n}^{1+2|s|/p}}\right\}}^{1/2}\right].$$

**Proof.**  Define

$$\varepsilon_{n}={\left\{\frac{\log n}{nh_{n}^{1+2|s|/p}}\right\}}^{1/2}.$$

We first control the martingale sum on a deterministic finite net whose mesh is chosen small enough to make the subsequent oscillation term negligible.

Let

$$\delta_{n}=b_{n}n^{-2}.$$

Because I is compact, it admits a Euclidean $\delta_{n}$-net $\left\{x_{n,1},\ldots ,x_{n,N_{n}}\right\}$ satisfying

$$N_{n}\leq C_{I}\delta_{n}^{-p}=C_{I}n^{2p}h_{n}^{-1}.$$

Assumption (H.2) implies

$$\log\left(h_{n}^{-1}\right)=O\left(\log n\right).$$

Indeed, for all sufficiently large *n*,

$$h_{n}^{1+2|s|/p}\geq \frac{\log n}{n}a_{n}$$

for some deterministic $a_{n}\to \infty$; hence,

$$\log\left(h_{n}^{-1}\right)\leq \frac{1}{1+2|s|/p}\log\left(\frac{n}{\log n}\right)+o\left(\log n\right).$$

Consequently,

$$\log N_{n}=O\left(\log n\right).$$

Fix a net point $x_{n,k}$. By Lemma 18, the martingale differences $\Delta_{i,n}\left(x_{n,k}\right)$ satisfy the Bernstein condition with

$$V_{n}=n{\bar{v}}^{2}h_{n}.$$

Let

$$t_{n}=A\sqrt{nh_{n}\log n},$$

where A>0. Since (H.2) implies that

$$\frac{nh_{n}}{\log n}⟶\infty ,$$

we have

$$\frac{t_{n}}{nh_{n}}=A{\left(\frac{\log n}{nh_{n}}\right)}^{1/2}⟶0.$$

Lemma 11 therefore gives, for all sufficiently large *n*,

$$\begin{array}{cc} P\left(\left|\sum_{i=1}^{n}\Delta_{i,n}\left(x_{n,k}\right)\right|\geq t_{n}\right) & \leq 2\exp\left\{-\frac{A^{2}nh_{n}\log n}{2(n{\bar{v}}^{2}h_{n}+\bar{c}A\sqrt{nh_{n}\log n})}\right\}\\ & \leq 2n^{-c_{0}A^{2}} \end{array}$$

for a constant $c_{0}>0$.

The union bound gives

$$\begin{array}{cc} & P\left(\max_{1\leq k\leq N_{n}}\left|\sum_{i=1}^{n}\Delta_{i,n}\left(x_{n,k}\right)\right|\geq t_{n}\right)\\ & \;\leq 2N_{n}n^{-c_{0}A^{2}}. \end{array}$$

Since $N_{n}$ is polynomial in *n*, *A* may be chosen so large that

$$\sum_{n=1}^{\infty }N_{n}n^{-c_{0}A^{2}}<\infty .$$

The Borel–Cantelli lemma yields

(186) $$\max_{1\leq k\leq N_{n}}\left|\sum_{i=1}^{n}\Delta_{i,n}\left(x_{n,k}\right)\right|=O_{a.s.}\left(\sqrt{nh_{n}\log n}\right).$$

We next pass from the net to the full compact set by controlling the oscillation between an arbitrary point and its nearest net point. Let x∈I, and choose k=k(x) such that $∥x-x_{n,k}∥\leq \delta_{n}$. The Lipschitz property of $\kappa_{s}$ gives

$$\begin{array}{cc} & \left|\Theta_{i,n}\left(x\right)-\Theta_{i,n}\left(x_{n,k}\right)\right|\\ & \;\leq L_{s}|W_{i}\left(\psi \right)|\frac{∥x-x_{n,k}∥}{b_{n}}\leq L_{s}n^{-2}\left|W_{i}\left(\psi \right)\right|. \end{array}$$

Conditional Jensen gives the corresponding bound

$$\begin{array}{cc} & \left|A_{i,n}\left(x\right)-A_{i,n}\left(x_{n,k}\right)\right|\\ & \;\leq L_{s}n^{-2}E\left[|W_{i}\left(\psi \right)||G_{i-1}\right]. \end{array}$$

Therefore,

$$\begin{array}{cc} & \underset{x\in I}{\sup}\left|M_{n,s}\left(x\right)-M_{n,s}\left(x_{n,k(x)}\right)\right|\\ & \leq \frac{L_{s}n^{-2}}{nh_{n}^{1+|s|/p}}\sum_{i=1}^{n}\left[|W_{i}\left(\psi \right)|+E\left(|W_{i}\left(\psi \right)||G_{i-1}\right)\right]. \end{array}$$

By stationarity, ergodicity, and integrability, both Cesàro averages in the last display are $O_{a.s.}\left(1\right)$. Hence, the oscillation is

$$O_{a.s.}\left(n^{-2}h_{n}^{-1-|s|/p}\right).$$

Relative to $\varepsilon_{n}$,

$$\begin{array}{cc} & \frac{n^{-2}h_{n}^{-1-|s|/p}}{\varepsilon_{n}}\\ & =\frac{n^{-2}h_{n}^{-1-|s|/p}\sqrt{nh_{n}^{1+2|s|/p}}}{\sqrt{\log n}}\\ & =\frac{1}{n^{3/2}h_{n}^{1/2}\sqrt{\log n}}⟶0, \end{array}$$

because $nh_{n}\to \infty$.

Dividing (186) by the estimator normalization $nh_{n}^{1+|s|/p}$,

$$\begin{array}{cc} \frac{\sqrt{nh_{n}\log n}}{nh_{n}^{1+|s|/p}} & ={\left\{\frac{\log n}{nh_{n}^{1+2|s|/p}}\right\}}^{1/2}. \end{array}$$

Combining the net and oscillation bounds proves the proposition. □

**Proof of Theorem 1.**  By Proposition 9,

$$\underset{x\in I}{\sup}\left|M_{n,s}\left(x\right)\right|=O_{a.s.}\left(\varepsilon_{n}\right).$$

By Lemma 17,

$$\begin{array}{cc} \underset{x\in I}{\sup}\left|P_{n,s}\left(x\right)\right| & \leq \frac{{∥K∥}_{1}}{n}\underset{u\in J}{\sup}\left|\sum_{i=1}^{n}G_{i,\psi }^{\left(s\right)}\left(u\right)\right|. \end{array}$$

Assumption (P.3) gives

$$\underset{x\in I}{\sup}\left|P_{n,s}\left(x\right)\right|=O_{a.s.}\left\{{\left(\frac{\log n}{n}\right)}^{1/2}\right\}.$$

Since $h_{n}\to 0$,

$${\left(\frac{\log n}{n}\right)}^{1/2}=h_{n}^{1/2+|s|/p}\varepsilon_{n}=o\left(\varepsilon_{n}\right).$$

The decomposition (164) completes the proof. □

**Proof of Theorem 2.**  For every x∈I,

$$\begin{array}{cc} & D^{s}r_{n}^{\;\mathrm{IPW}}\left(\psi ;x,h_{n}\right)-D^{s}r\left(\psi ;x\right)\\ & =\left[D^{s}r_{n}^{\;\mathrm{IPW}}-ED^{s}r_{n}^{\;\mathrm{IPW}}\right]\left(x\right)\\ & \;+\left[ED^{s}r_{n}^{\;\mathrm{IPW}}-D^{s}r\right]\left(x\right). \end{array}$$

Taking suprema and applying Theorem 1 and Proposition 2 yields

$$\begin{array}{cc} & \underset{x\in I}{\sup}\left|D^{s}r_{n}^{\;\mathrm{IPW}}\left(\psi ;x,h_{n}\right)-D^{s}r\left(\psi ;x\right)\right|\\ & =O_{a.s.}\left[{\left\{\frac{\log n}{nh_{n}^{1+2|s|/p}}\right\}}^{1/2}\right]+O\left(h_{n}^{\ell /p}\right). \end{array}$$

Both terms converge to zero under (H.2) and $h_{n}\to 0$. □

**Proof of Corollary 1.**  Put $V_{i}\equiv 1$. Then, the response-dependent density $g_{i,\psi }$ is replaced by the conditional covariate density $f_{i}$, and the population target r(1;·) equals $f_{X}$. With this specialization, $\Theta_{i,n}$ becomes the ordinary localized kernel term, $A_{i,n}$ is its conditional-density convolution, and the predictable field $G_{i,\psi }^{\left(s\right)}$ is replaced by the centred derivative field $D^{s}f_{i}-D^{s}f_{X}$. The net Bernstein bound, the predictable-component estimate, and the deterministic bias expansion therefore yield the same uniform rate and consistency conclusion, now without any inverse-probability factor. □

### 7.7. A Self-Contained Martingale Triangular-Array Theorem

The pointwise limit theory will be reduced to the following martingale triangular-array central limit theorem. We include a complete proof in the normalization needed later. The argument uses successive predictable and realized-quadratic-variation localizations together with the McLeish product method, making the truncation and uniform-integrability steps explicit.

**Lemma 20** 
(Martingale triangular-array central limit theorem). *For every n, let*

$$F_{n,0}\subset F_{n,1}\subset \cdots \subset F_{n,k_{n}}$$

*be a filtration, and let ${\left(X_{n,j}\right)}_{1\leq j\leq k_{n}}$ be square-integrable martingale differences:*

$$X_{n,j}\in L^{2}\left(F_{n,j}\right),\;E\left(X_{n,j}|F_{n,j-1}\right)=0.$$

*Put*

$$S_{n}=\sum_{j=1}^{k_{n}}X_{n,j},$$

$$V_{n}=\sum_{j=1}^{k_{n}}E\left(X_{n,j}^{2}|F_{n,j-1}\right),\;Q_{n}=\sum_{j=1}^{k_{n}}X_{n,j}^{2}.$$

*Assume that, for some deterministic $\sigma^{2}\in \left[0,\infty \right)$,*

(187) $$V_{n}\overset{P}{\to }\sigma^{2},$$

*and that, for every ε>0,*

(188) $$L_{n}\left(\varepsilon \right):=\sum_{j=1}^{k_{n}}E\left[X_{n,j}^{2}1_{\left\{|X_{n,j}|>\varepsilon \right\}}|F_{n,j-1}\right]\overset{P}{\to }0.$$

*Then,*

$$S_{n}\overset{D}{\to }N\left(0,\sigma^{2}\right).$$

**Proof.**  Fix $C>\sigma^{2}+1$. Define the predictable-variation stopping time

$$\tau_{n}^{C}=\inf\left\{j\geq 1:\sum_{\ell =1}^{j}E\left(X_{n,\ell }^{2}|F_{n,\ell -1}\right)>C\right\}\wedge k_{n}$$

and the stopped increments

$$X_{n,j}^{C}=X_{n,j}1_{\left\{j\leq \tau_{n}^{C}\right\}}.$$

Because $\left\{j\leq \tau_{n}^{C}\right\}\in F_{n,j-1}$, the stopped variables remain martingale differences. Moreover,

$$P\left(\tau_{n}^{C}<k_{n}\right)\leq P\left(V_{n}>C\right)⟶0.$$

Since the stopping event has asymptotically vanishing probability, it suffices to prove the theorem for the stopped array. To simplify notation, we suppress the superscript *C*. The stopped predictable variation satisfies

$$V_{n}\leq C+\max_{j}E\left(X_{n,j}^{2}|F_{n,j-1}\right).$$

The conditional Lindeberg condition implies that

$$\begin{array}{cc} \max_{j}E\left(X_{n,j}^{2}|F_{n,j-1}\right) & \leq \varepsilon^{2}+L_{n}\left(\varepsilon \right) \end{array}$$

for every ε>0. Hence, the maximal conditional variance tends to zero in probability. Define the predictable stopping index

$$\kappa_{n}=\inf\left\{j:E(X_{n,j}^{2}|F_{n,j-1})>1\right\},$$

with $\inf⌀=k_{n}+1$, and replace $X_{n,j}$ with $X_{n,j}1_{\left\{j<\kappa_{n}\right\}}$. The indicator is $F_{n,j-1}$-measurable, so the martingale-difference property is preserved, and

$$P(\kappa_{n}\leq k_{n})⟶0.$$

For the twice-stopped array,

$$V_{n}\leq C+1\;a.s.$$

Under this localization,

$$0\leq L_{n}\left(\varepsilon \right)\leq V_{n}\leq C+1.$$

Convergence in probability in (188) and boundedness now imply

(189) $$EL_{n}\left(\varepsilon \right)⟶0\;\left(\varepsilon >0\right).$$

We first establish the infinitesimality condition

(190) $$\max_{1\leq j\leq k_{n}}\left|X_{n,j}\right|\overset{P}{\to }0.$$

By the union bound and conditional Markov inequality,

$$\begin{array}{cc} P\left(\max_{j}\left|X_{n,j}\right|>\varepsilon \right) & \leq \sum_{j}P(|X_{n,j}\left|>\varepsilon \right)\\ & =\sum_{j}E\left[P(|X_{n,j}|>\varepsilon |F_{n,j-1})\right]\\ & \leq \varepsilon^{-2}EL_{n}\left(\varepsilon \right)⟶0. \end{array}$$

We next identify realized and predictable quadratic variation asymptotically:

(191) $$Q_{n}-V_{n}\overset{P}{\to }0.$$

Fix ε>0 and decompose

$$Q_{n}-V_{n}=A_{n,\varepsilon }+B_{n,\varepsilon },$$

where

$$\begin{array}{cc} A_{n,\varepsilon } & =\sum_{j}\left[X_{n,j}^{2}1_{\left\{|X_{n,j}|\leq \varepsilon \right\}}-E\left(X_{n,j}^{2}1_{\left\{|X_{n,j}|\leq \varepsilon \right\}}|F_{n,j-1}\right)\right],\\ B_{n,\varepsilon } & =\sum_{j}\left[X_{n,j}^{2}1_{\left\{|X_{n,j}|>\varepsilon \right\}}-E\left(X_{n,j}^{2}1_{\left\{|X_{n,j}|>\varepsilon \right\}}|F_{n,j-1}\right)\right]. \end{array}$$

The summands in $A_{n,\varepsilon }$ form a martingale-difference array. Orthogonality gives

$$\begin{array}{cc} EA_{n,\varepsilon }^{2} & =\sum_{j}E\mathrm{Var}\left(X_{n,j}^{2}1_{\left\{|X_{n,j}|\leq \varepsilon \right\}}|F_{n,j-1}\right)\\ & \leq \sum_{j}E\left[X_{n,j}^{4}1_{\left\{|X_{n,j}|\leq \varepsilon \right\}}\right]\\ & \leq \varepsilon^{2}\sum_{j}EX_{n,j}^{2}\\ & =\varepsilon^{2}EV_{n}\leq \varepsilon^{2}\left(C+1\right). \end{array}$$

Thus, according to Chebyshev,

$$\underset{n\to \infty }{lim sup}P(|A_{n,\varepsilon }\left|>\delta \right)\leq \frac{\varepsilon^{2}\left(C+1\right)}{\delta^{2}}.$$

For the large-increment term,

$$\begin{array}{cc} E|B_{n,\varepsilon }| & \leq 2\sum_{j}E\left[X_{n,j}^{2}1_{\left\{|X_{n,j}|>\varepsilon \right\}}\right]\\ & =2EL_{n}\left(\varepsilon \right)⟶0. \end{array}$$

First, let n→∞, and then let ε↓0, to obtain (191). Combined with (187), this yields

(192) $$Q_{n}\overset{P}{\to }\sigma^{2}.$$

We finally establish convergence of the characteristic functions. Fix t∈R. By (190), the stopping index

$$\lambda_{n}=\inf\{j:|X_{n,j}\left|>1\right\},\;\inf⌀=k_{n}+1,$$

satisfies $P(\lambda_{n}\leq k_{n})\to 0$. Replace $X_{n,j}$ with $X_{n,j}1_{\left\{j<\lambda_{n}\right\}}$; this is again a predictable stopping and guarantees

$$\max_{j}\left|X_{n,j}\right|\leq 1\;a.s.$$

Choose $D>\sigma^{2}+1$, and stop the array once its realized quadratic variation exceeds *D*:

$$\rho_{n}^{D}=\inf\left\{j\geq 1:\sum_{\ell =1}^{j}X_{n,\ell }^{2}>D\right\}\wedge k_{n},$$

$${\bar{X}}_{n,j}=X_{n,j}1_{\left\{j\leq \rho_{n}^{D}\right\}}.$$

This is again a martingale-difference array because $\left\{j\leq \rho_{n}^{D}\right\}\in F_{n,j-1}$. By (192),

$$P(\rho_{n}^{D}<k_{n})⟶0.$$

We again suppress the bar. The stopped realized quadratic variation satisfies

$$Q_{n}\leq D+\max_{j}X_{n,j}^{2}\leq D+1\;a.s.$$

Define

$$P_{n}\left(t\right)=\prod_{j=1}^{k_{n}}\left(1+itX_{n,j}\right).$$

Since $X_{n,j}$ is a martingale difference,

$$\begin{array}{cc} E\left[P_{n}\left(t\right)\right] & =E\left[\prod_{j=1}^{k_{n}-1}\left(1+itX_{n,j}\right)E\left(1+itX_{n,k_{n}}|F_{n,k_{n}-1}\right)\right]\\ & =E\left[\prod_{j=1}^{k_{n}-1}\left(1+itX_{n,j}\right)\right]. \end{array}$$

Backward iteration gives

(193) $$EP_{n}\left(t\right)=1.$$

Integrability is guaranteed by

$$\begin{array}{cc} |P_{n}\left(t\right)| & =\prod_{j}{\left(1+t^{2}X_{n,j}^{2}\right)}^{1/2}\\ & \leq \exp\left(\frac{t^{2}}{2}\sum_{j}X_{n,j}^{2}\right)=\exp\left(t^{2}Q_{n}/2\right), \end{array}$$

which is bounded by $\exp\{t^{2}\left(D+1\right)/2\}$ after the stopping.

On the event

$$\left|t\right|\max_{j}\left|X_{n,j}\right|\leq \frac{1}{2},$$

use the principal logarithm. Taylor’s theorem for z↦log(1+z) on |z|≤1/2 gives

$$\log\left(1+itx\right)=itx+\frac{t^{2}x^{2}}{2}+r_{t}\left(x\right),\;|r_{t}\left(x\right)|\leq C_{t}{\left|x\right|}^{3}.$$

Consequently,

$$\log P_{n}\left(t\right)=itS_{n}+\frac{t^{2}}{2}Q_{n}+R_{n}\left(t\right),$$

where

$$\begin{array}{cc} |R_{n}\left(t\right)| & \leq C_{t}\sum_{j}{\left|X_{n,j}\right|}^{3}\\ & \leq C_{t}\left(\max_{j}\left|X_{n,j}\right|\right)Q_{n}\overset{P}{\to }0 \end{array}$$

by (190) and tightness of $Q_{n}$. Thus,

(194) $$P_{n}\left(t\right)-\exp\left(itS_{n}+\frac{t^{2}}{2}Q_{n}\right)\overset{P}{\to }0.$$

Both terms in (194) are uniformly bounded after stopping: the product is bounded by $\exp(t^{2}\left(D+1\right)/2)$, and the exponential on the right has the same modulus. Therefore, convergence in probability upgrades to convergence in $L^{1}$:

(195) $$E\left|P_{n}\left(t\right)-\exp\left(itS_{n}+\frac{t^{2}}{2}Q_{n}\right)\right|⟶0.$$

Multiplying by $\exp(-t^{2}Q_{n}/2)$, whose modulus is at most one, gives

$$E\left|e^{itS_{n}}-e^{-t^{2}Q_{n}/2}P_{n}\left(t\right)\right|⟶0.$$

Since $Q_{n}\to \sigma^{2}$ in probability and $Q_{n}$ is localized to a bounded set,

$$e^{-t^{2}Q_{n}/2}⟶e^{-t^{2}\sigma^{2}/2}$$

in $L^{1}$. The product $P_{n}\left(t\right)$ is uniformly bounded under the same localization, so

$$\begin{array}{cc} & E\left[e^{-t^{2}Q_{n}/2}P_{n}\left(t\right)\right]-e^{-t^{2}\sigma^{2}/2}EP_{n}\left(t\right)\\ & ⟶0. \end{array}$$

Using (193),

$$Ee^{itS_{n}}⟶e^{-t^{2}\sigma^{2}/2}.$$

Lévy’s continuity theorem yields

$$S_{n}\overset{D}{\to }N\left(0,\sigma^{2}\right)$$

for every fixed localization level C,D. The probability that either stopping operation changes the original sum tends to zero as first n→∞ and then C,D→∞. Slutsky’s theorem removes the localization and completes the proof. □

### 7.8. Pointwise Martingale Central Limit Calculations

The preceding abstract theorem is now applied to the localized kernel martingale array. The next lemma verifies the conditional variance and Lindeberg conditions at a fixed interior point.

**Lemma 21** 
(Martingale-array limit). *Assume* (S.1)–(S.3)*,* (K.1)*,* (C.1)–(C.3)*, and $nh_{n}\to \infty$. For fixed x∈I,*

(196) $$\sqrt{nh_{n}^{1+2|s|/p}}M_{n,s}\left(x\right)\overset{D}{\to }N\left(0,\sigma_{\psi ,s}^{2}\left(x\right)\right).$$

**Proof.**  Define the uncentred normalized increment

(197) $$\eta_{ni}\left(x,\psi \right)=\frac{1}{\sqrt{nh_{n}}}\Theta_{i,n}\left(x\right)={\left(\frac{1}{nh_{n}}\right)}^{1/2}W_{i}\left(\psi \right)D^{s}K\left(\frac{x-X_{i}}{b_{n}}\right)$$

and its martingale centring

(198) $$\zeta_{ni}\left(x,\psi \right)=\eta_{ni}\left(x,\psi \right)-E\left[\eta_{ni}\left(x,\psi \right)|G_{i-1}\right].$$

Equivalently,

$$\zeta_{ni}\left(x,\psi \right)=\frac{1}{\sqrt{nh_{n}}}\Delta_{i,n}\left(x\right).$$

For notational economy in this proof, put

$$d_{i,n}\left(x\right)=\zeta_{ni}\left(x,\psi \right).$$

Then,

$$\sum_{i=1}^{n}d_{i,n}\left(x\right)=\sqrt{nh_{n}^{1+2|s|/p}}M_{n,s}\left(x\right).$$

The array $\left(d_{i,n}\left(x\right),G_{i}\right)$ consists of martingale differences.

We first verify convergence of the predictable quadratic variation. Since

$$\Delta_{i,n}=\Theta_{i,n}-A_{i,n},$$

$$\begin{array}{cc} & \sum_{i=1}^{n}E\left[d_{i,n}^{2}\left(x\right)|G_{i-1}\right]\\ & =\frac{1}{nh_{n}}\sum_{i=1}^{n}E\left[\Theta_{i,n}^{2}\left(x\right)|G_{i-1}\right]-\frac{1}{nh_{n}}\sum_{i=1}^{n}A_{i,n}^{2}\left(x\right). \end{array}$$

By the definition of $q_{i,\psi }$,

$$\begin{array}{cc} & E\left[\Theta_{i,n}^{2}\left(x\right)|G_{i-1}\right]\\ & =\int \kappa_{s}^{2}\left(\frac{x-u}{b_{n}}\right)q_{i,\psi }\left(u\right)\;du. \end{array}$$

Changing variables gives

$$\begin{array}{cc} & \frac{1}{nh_{n}}\sum_{i=1}^{n}E\left[\Theta_{i,n}^{2}\left(x\right)|G_{i-1}\right]\\ & =\int_{R^{p}}\kappa_{s}^{2}\left(v\right)\left\{\frac{1}{n}\sum_{i=1}^{n}q_{i,\psi }\left(x-b_{n}v\right)\right\}dv. \end{array}$$

For all sufficiently large *n*, the points $x-b_{n}v$, with v in the support of *K*, belong to $N_{x}$. By (C.1),

$$\underset{u\in N_{x}}{\sup}\left|\frac{1}{n}\sum_{i=1}^{n}q_{i,\psi }\left(u\right)-q_{\psi }\left(u\right)\right|\overset{P}{\to }0.$$

Hence, the preceding integral differs by $o_{P}\left(1\right)$ from

$$\int \kappa_{s}^{2}\left(v\right)q_{\psi }\left(x-b_{n}v\right)\;dv.$$

Continuity of $q_{\psi }$ at x, dominated convergence, and $\kappa_{s}\in L^{2}$ imply that

$$\int \kappa_{s}^{2}\left(v\right)q_{\psi }\left(x-b_{n}v\right)\;dv⟶q_{\psi }\left(x\right)R_{s}\left(K\right).$$

It remains to verify that the contribution of the squared conditional means is negligible at the quadratic-variation scale. By Lemma 17,

$$A_{i,n}\left(x\right)=h_{n}^{1+|s|/p}\int K\left(v\right)D^{s}g_{i,\psi }\left(x-b_{n}v\right)\;dv.$$

The local $L^{2}$-boundedness in (P.1) gives

$$\begin{array}{cc} E\left[A_{i,n}^{2}\left(x\right)\right] & \leq h_{n}^{2+2|s|/p}{∥K∥}_{1}\int \left|K\left(v\right)\right|E\left[{\left|D^{s}g_{i,\psi }\left(x-b_{n}v\right)\right|}^{2}\right]dv\\ & \leq Ch_{n}^{2+2|s|/p}. \end{array}$$

Therefore,

$$\begin{array}{cc} E\left[\frac{1}{nh_{n}}\sum_{i=1}^{n}A_{i,n}^{2}\left(x\right)\right] & \leq Ch_{n}^{1+2|s|/p}⟶0. \end{array}$$

Markov’s inequality gives

$$\frac{1}{nh_{n}}\sum_{i=1}^{n}A_{i,n}^{2}\left(x\right)=o_{P}\left(1\right).$$

Thus,

$$\sum_{i=1}^{n}E\left[d_{i,n}^{2}\left(x\right)|G_{i-1}\right]\overset{P}{\to }\sigma_{\psi ,s}^{2}\left(x\right).$$

We finally verify the conditional Lindeberg condition. For every ε>0, conditional Markov’s inequality gives

$$\begin{array}{cc} & \sum_{i=1}^{n}E\left[d_{i,n}^{2}\left(x\right)1_{\left\{|d_{i,n}\left(x\right)|>\varepsilon \right\}}|G_{i-1}\right]\\ & \leq \varepsilon^{-\eta }\sum_{i=1}^{n}E\left[|d_{i,n}{\left(x\right)|}^{2+\eta }|G_{i-1}\right]. \end{array}$$

Conditional centring and Jensen’s inequality imply that

$$E\left[|\Delta_{i,n}{\left(x\right)|}^{2+\eta }|G_{i-1}\right]\leq 2^{2+\eta }E\left[|\Theta_{i,n}{\left(x\right)|}^{2+\eta }|G_{i-1}\right].$$

Using (C.2), the conditional density bound, and the same change of variables as above,

$$\begin{array}{cc} & E\left[|\Theta_{i,n}{\left(x\right)|}^{2+\eta }|G_{i-1}\right]\\ & \leq C_{f}C_{2+\eta }h_{n}\int {\left|\kappa_{s}\left(v\right)\right|}^{2+\eta }\;dv. \end{array}$$

Here, $C_{2+\eta }=O_{P}\left(1\right)$ is the random bound from (C.2). Since $\kappa_{s}$ is bounded and square-integrable, it belongs to $L^{2+\eta }$. Consequently,

$$\begin{array}{cc} \sum_{i=1}^{n}E\left[|d_{i,n}{\left(x\right)|}^{2+\eta }|G_{i-1}\right] & \leq C_{K}C_{2+\eta }\frac{nh_{n}}{{\left(nh_{n}\right)}^{1+\eta /2}}\\ & =C_{K}C_{2+\eta }{\left(nh_{n}\right)}^{-\eta /2}\overset{P}{\to }0. \end{array}$$

Indeed, for every δ>0, choose *M* such that $\sup_{n}P\left(C_{2+\eta }>M\right)<\delta$; on $\left\{C_{2+\eta }\leq M\right\}$, the right-hand side is bounded by the deterministic quantity $C_{K}M{\left(nh_{n}\right)}^{-\eta /2}\to 0$. Lemma 20, applied with $X_{n,i}=d_{i,n}\left(x\right)$, now proves (196). □

**Lemma 22** 
(Negligibility of the predictable component). *Under* (P.1)–(P.2)*,*

(199) $$\sqrt{nh_{n}^{1+2|s|/p}}P_{n,s}\left(x\right)=o_{P}\left(1\right)$$

*for every fixed x∈I.*

**Proof.**  By (180),

$$|P_{n,s}\left(x\right)|\leq \frac{{∥K∥}_{1}}{n}\underset{u\in J}{\sup}\left|\sum_{i=1}^{n}G_{i,\psi }^{\left(s\right)}\left(u\right)\right|.$$

Assumption (P.2) therefore gives

$$\begin{array}{cc} E\left[P_{n,s}^{2}\left(x\right)\right] & \leq \frac{{∥K∥}_{1}^{2}}{n^{2}}E\left[\underset{u\in J}{\sup}{\left|\sum_{i=1}^{n}G_{i,\psi }^{\left(s\right)}\left(u\right)\right|}^{2}\right]\\ & \leq \frac{C}{n}. \end{array}$$

Hence,

$$\begin{array}{cc} E\left[nh_{n}^{1+2|s|/p}P_{n,s}^{2}\left(x\right)\right] & \leq Ch_{n}^{1+2|s|/p}⟶0. \end{array}$$

Thus, the normalized predictable component converges to zero in $L^{2}$, and therefore in probability. □

**Lemma 23** 
(Normalized bias). *Under Proposition 2,*

(200) $$\begin{array}{cc} \; & \sqrt{nh_{n}^{1+2|s|/p}}\left[ED^{s}r_{n}^{\;\mathrm{IPW}}\left(\psi ;x,h_{n}\right)-D^{s}r\left(\psi ;x\right)\right]\\ \; & =\sqrt{nh_{n}^{1+2|s|/p}}h_{n}^{\ell /p}B_{s,\ell }\left(x\right)+o\left(\sqrt{nh_{n}^{1+2|s|/p}}h_{n}^{\ell /p}\right). \end{array}$$

*Consequently, under* (H.3)*,*

(201) $$\begin{array}{cc} \; & \sqrt{nh_{n}^{1+2|s|/p}}\left[ED^{s}r_{n}^{\;\mathrm{IPW}}\left(\psi ;x,h_{n}\right)-D^{s}r\left(\psi ;x\right)\right]⟶0. \end{array}$$

*Under* (H.4)*, the same left-hand side tends to $\lambda B_{s,\ell }\left(x\right)$.*

**Proof.**  Multiplying the bias expansion

$$ED^{s}r_{n}^{\;\mathrm{IPW}}-D^{s}r=h_{n}^{\ell /p}B_{s,\ell }+o\left(h_{n}^{\ell /p}\right)$$

by the central-limit normalization, the two asserted regimes follow immediately. □

**Proof of Theorem 3.**  By (164),

$$\begin{array}{cc} & \sqrt{nh_{n}^{1+2|s|/p}}\left[D^{s}r_{n}^{\;\mathrm{IPW}}-ED^{s}r_{n}^{\;\mathrm{IPW}}\right]\left(x\right)\\ & =\sqrt{nh_{n}^{1+2|s|/p}}M_{n,s}\left(x\right)+\sqrt{nh_{n}^{1+2|s|/p}}P_{n,s}\left(x\right). \end{array}$$

The martingale term converges in distribution to the stated Gaussian law by Lemma 21; the second converges to zero in probability by Lemma 22. Slutsky’s theorem therefore yields the asserted limit. □

**Proof of Theorem 4.**  Decompose

$$\begin{array}{cc} & \sqrt{nh_{n}^{1+2|s|/p}}\left[D^{s}r_{n}^{\;\mathrm{IPW}}-D^{s}r\right]\left(x\right)\\ & =\sqrt{nh_{n}^{1+2|s|/p}}\left[D^{s}r_{n}^{\;\mathrm{IPW}}-ED^{s}r_{n}^{\;\mathrm{IPW}}\right]\left(x\right)\\ & \;+\sqrt{nh_{n}^{1+2|s|/p}}\left[ED^{s}r_{n}^{\;\mathrm{IPW}}-D^{s}r\right]\left(x\right). \end{array}$$

The first term converges to $N(0,\sigma_{\psi ,s}^{2}\left(x\right))$ by Theorem 3. Lemma 23 shows that the second term tends either to zero under (H.3) or to $\lambda B_{s,\ell }\left(x\right)$ under (H.4). Slutsky’s theorem proves both assertions. □

**Proof of Corollary 2.**  Take $V_{i}\equiv 1$. The local second-moment density becomes

$$q_{1}\left(x\right)=f_{X}\left(x\right),$$

and therefore

$$q_{1}\left(x\right)R_{s}\left(K\right)=f_{X}\left(x\right)R_{s}\left(K\right).$$

All arguments in Lemmas 21–23 then apply with the IPW response component suppressed. □

### 7.9. Joint Primitive Limits

The regression-derivative theory requires joint, rather than merely marginal, limits for numerator and denominator derivatives. We therefore establish the finite-dimensional Gaussian limit for an arbitrary collection of primitive localized estimators, allowing derivative orders and response transforms to differ across coordinates. The proof proceeds by Cramér–Wold reduction to a scalar martingale array and retains the mixed kernel-derivative inner products that determine the off-diagonal covariance terms.

**Proof of Theorem 5.**  For each a∈{1,…,d}, define

$$\Theta_{i,n}^{\left(a\right)}=V_{i}^{\left(a\right)}D^{s_{a}}K\left(\frac{x-X_{i}}{b_{n}}\right)$$

and

$$d_{i,n}^{\left(a\right)}=\frac{1}{\sqrt{nh_{n}}}\left[\Theta_{i,n}^{\left(a\right)}-E\left(\Theta_{i,n}^{\left(a\right)}|G_{i-1}\right)\right].$$

Then,

$$\sum_{i=1}^{n}d_{i,n}^{\left(a\right)}=\sqrt{nh_{n}^{1+2|s_{a}|/p}}\left[T_{n,a}\left(x\right)-{\tilde{T}}_{n,a}\left(x\right)\right],$$

where ${\tilde{T}}_{n,a}$ denotes the predictable version.

Fix $t={\left(t_{1},\ldots ,t_{d}\right)}^{⊤}\in R^{d}$, and set

$$D_{i,n}\left(t\right)=\sum_{a=1}^{d}t_{a}d_{i,n}^{\left(a\right)}.$$

This is a scalar martingale-difference array. Its conditional variance is

$$\begin{array}{cc} & \sum_{i=1}^{n}E\left[D_{i,n}^{2}\left(t\right)|G_{i-1}\right]\\ & =\sum_{a=1}^{d}\sum_{b=1}^{d}t_{a}t_{b}\sum_{i=1}^{n}E\left[d_{i,n}^{\left(a\right)}d_{i,n}^{\left(b\right)}|G_{i-1}\right]. \end{array}$$

The uncentred cross-moment equals

$$\begin{array}{cc} & \frac{1}{nh_{n}}\sum_{i=1}^{n}E\left[\Theta_{i,n}^{\left(a\right)}\Theta_{i,n}^{\left(b\right)}|G_{i-1}\right]\\ & =\int D^{s_{a}}K\left(v\right)D^{s_{b}}K\left(v\right)\left\{\frac{1}{n}\sum_{i=1}^{n}q_{i,ab}\left(x-b_{n}v\right)\right\}dv, \end{array}$$

where $q_{i,ab}$ is the conditional cross-moment density. Joint stabilization gives convergence in probability to

$$q_{ab}\left(x\right)\int D^{s_{a}}K\left(v\right)D^{s_{b}}K\left(v\right)\;dv=\Sigma_{ab}\left(x\right).$$

It remains to remove the products of conditional means introduced by martingale centring. Write

$$A_{i,n}^{\left(a\right)}=E\left(\Theta_{i,n}^{\left(a\right)}|G_{i-1}\right).$$

The convolution representation associated with component *a* gives

$$A_{i,n}^{\left(a\right)}=h_{n}^{1+|s_{a}|/p}\int K\left(v\right)D^{s_{a}}g_{i}^{\left(a\right)}\left(x-b_{n}v\right)\;dv.$$

Under the local $L^{2}$-bounds imposed on the corresponding conditional moment-density fields, Cauchy–Schwarz yields, for every pair a,b,

$$\begin{array}{cc} E\left[\frac{1}{nh_{n}}\sum_{i=1}^{n}\left|A_{i,n}^{\left(a\right)}A_{i,n}^{\left(b\right)}\right|\right] & \leq Ch_{n}^{1+(|s_{a}|+|s_{b}|)/p}⟶0. \end{array}$$

Therefore,

$$\frac{1}{nh_{n}}\sum_{i=1}^{n}A_{i,n}^{\left(a\right)}A_{i,n}^{\left(b\right)}=o_{P}\left(1\right),$$

so martingale centring does not alter the limiting cross-covariance. Hence,

$$\sum_{i=1}^{n}E\left[D_{i,n}^{2}\left(t\right)|G_{i-1}\right]\overset{P}{\to }t^{⊤}\Sigma \left(x\right)t.$$

The joint conditional moment assumptions imply the Lindeberg condition for $D_{i,n}\left(t\right)$. Indeed, in finite dimension,

$${\left|\sum_{a=1}^{d}t_{a}d_{i,n}^{\left(a\right)}\right|}^{2+\eta }\leq C_{t,d}\sum_{a=1}^{d}{\left|d_{i,n}^{\left(a\right)}\right|}^{2+\eta },$$

and every sum on the right is $O_{P}\left({\left(nh_{n}\right)}^{-\eta /2}\right)$.

The scalar martingale CLT gives

$$\sum_{a=1}^{d}t_{a}\sqrt{nh_{n}^{1+2|s_{a}|/p}}\left[T_{n,a}\left(x\right)-{\tilde{T}}_{n,a}\left(x\right)\right]\overset{D}{\to }N\left(0,t^{⊤}\Sigma \left(x\right)t\right).$$

The predictable components are negligible component-wise by Lemma 22; therefore, they are negligible in every finite linear combination. The Cramér–Wold device proves the vector convergence.

If the normalized bias of every component tends to zero, replacing $ET_{n,a}$ with $\theta_{a}$ follows from Slutsky’s theorem. □

### 7.10. Variance and Integrated-Risk Expansions

We next pass from distributional convergence to second-order risk expansions. Because convergence in distribution alone does not identify variances or integrated risks, the variance expansion is derived directly from the exact martingale-predictable decomposition, and the AMISE calculation is based on separate integrated control of squared bias, martingale variance, predictable variance, and martingale-predictable covariance.

**Proof of Proposition 3.**  We derive the leading variance directly from the exact martingale-predictable decomposition rather than inferring it from the central limit theorem. Write

$${\hat{\theta }}_{n}\left(x\right)=D^{s}r_{n}^{\;\mathrm{IPW}}\left(\psi ;x,h_{n}\right)$$

and use the exact decomposition

$${\hat{\theta }}_{n}-E{\hat{\theta }}_{n}=M_{n,s}+P_{n,s}.$$

Thus,

(202) $$\begin{array}{cc} \mathrm{Var}\{{\hat{\theta }}_{n}\left(x\right)\}\; & =EM_{n,s}^{2}\left(x\right)+EP_{n,s}^{2}\left(x\right)\\ \; & \;+2E\left[M_{n,s}\left(x\right)P_{n,s}\left(x\right)\right]. \end{array}$$

The second and third terms are negligible at the stated scale by the predictable-variance and covariance assumptions. It therefore remains only to identify the leading martingale variance.

Since the increments $\Delta_{i,n}\left(x\right)$ are martingale differences, they are orthogonal in $L^{2}$: if i<j, then

$$\begin{array}{cc} E\left[\Delta_{i,n}\left(x\right)\Delta_{j,n}\left(x\right)\right] & =E\left[\Delta_{i,n}\left(x\right)E\left(\Delta_{j,n}\left(x\right)|G_{j-1}\right)\right]=0. \end{array}$$

Therefore,

(203) $$\begin{array}{cc} EM_{n,s}^{2}\left(x\right)\; & =\frac{1}{n^{2}h_{n}^{2+2|s|/p}}\sum_{i=1}^{n}E\Delta_{i,n}^{2}\left(x\right). \end{array}$$

Conditional variance decomposition gives

$$\begin{array}{cc} E\Delta_{i,n}^{2}\left(x\right) & =E\Theta_{i,n}^{2}\left(x\right)-EA_{i,n}^{2}\left(x\right). \end{array}$$

We first evaluate the uncentred localized second moment. By conditional disintegration,

$$\begin{array}{cc} E\Theta_{i,n}^{2}\left(x\right) & =E\int_{J}\kappa_{s}^{2}\left(\frac{x-u}{b_{n}}\right)q_{i,\psi }\left(u\right)\;du. \end{array}$$

Stationarity of the unconditional law implies that

$$Eq_{i,\psi }\left(u\right)=q_{\psi }\left(u\right)$$

for Lebesgue-almost every u. Hence,

$$\begin{array}{cc} E\Theta_{i,n}^{2}\left(x\right) & =\int_{J}\kappa_{s}^{2}\left(\frac{x-u}{b_{n}}\right)q_{\psi }\left(u\right)\;du\\ & =h_{n}\int_{R^{p}}\kappa_{s}^{2}\left(v\right)q_{\psi }\left(x-b_{n}v\right)\;dv. \end{array}$$

Continuity of $q_{\psi }$ at x and dominated convergence give

(204) $$E\Theta_{i,n}^{2}\left(x\right)=h_{n}q_{\psi }\left(x\right)R_{s}\left(K\right)+o\left(h_{n}\right).$$

The domination is

$$\kappa_{s}^{2}\left(v\right)\underset{u\in N_{x}}{\sup}q_{\psi }\left(u\right),$$

which is integrable because $\kappa_{s}\in L^{2}$.

For the squared conditional mean, Lemma 17 gives

$$A_{i,n}\left(x\right)=h_{n}^{1+|s|/p}\int K\left(v\right)D^{s}g_{i,\psi }\left(x-b_{n}v\right)\;dv.$$

By weighted Cauchy–Schwarz,

$$\begin{array}{cc} A_{i,n}^{2}\left(x\right) & \leq h_{n}^{2+2|s|/p}{∥K∥}_{1}\int \left|K\left(v\right)\right|{\left|D^{s}g_{i,\psi }\left(x-b_{n}v\right)\right|}^{2}\;dv. \end{array}$$

Taking expectations and using (P.1) yields

(205) $$EA_{i,n}^{2}\left(x\right)=O\left(h_{n}^{2+2|s|/p}\right)=o\left(h_{n}\right).$$

The last equality follows because 1+2|s|/p>0.

Substituting (204) and (205) into (203), and using stationarity, gives

$$\begin{array}{cc} EM_{n,s}^{2}\left(x\right) & =\frac{n[h_{n}q_{\psi }\left(x\right)R_{s}\left(K\right)+o\left(h_{n}\right)]}{n^{2}h_{n}^{2+2|s|/p}}\\ & =\frac{q_{\psi }\left(x\right)R_{s}\left(K\right)}{nh_{n}^{1+2|s|/p}}+o\left(\frac{1}{nh_{n}^{1+2|s|/p}}\right). \end{array}$$

By assumption,

$$EP_{n,s}^{2}\left(x\right)=o\left(\frac{1}{nh_{n}^{1+2|s|/p}}\right)$$

and

$$E\left[M_{n,s}\left(x\right)P_{n,s}\left(x\right)\right]=o\left(\frac{1}{nh_{n}^{1+2|s|/p}}\right).$$

Equation (202) therefore yields

$$\begin{array}{cc} \mathrm{Var}\{D^{s}r_{n}^{\;\mathrm{IPW}}\left(\psi ;x,h_{n}\right)\} & =\frac{\sigma_{\psi ,s}^{2}\left(x\right)}{nh_{n}^{1+2|s|/p}}\\ & \;+o\left(\frac{1}{nh_{n}^{1+2|s|/p}}\right), \end{array}$$

because

$$\sigma_{\psi ,s}^{2}\left(x\right)=q_{\psi }\left(x\right)R_{s}\left(K\right).$$

This calculation also isolates the precise role of the additional integrated dependence assumptions: martingale orthogonality removes cross-products among distinct martingale increments, but it does not eliminate either the predictable variance or the martingale-predictable covariance without further structure. □

**Proof of Theorem 6.**  Write

$${\hat{\theta }}_{n}\left(x\right)=D^{s}r_{n}^{\;\mathrm{IPW}}\left(\psi ;x,h_{n}\right),\;\theta \left(x\right)=D^{s}r\left(\psi ;x\right).$$

For every x,

$$\begin{array}{cc} E{\left[{\hat{\theta }}_{n}\left(x\right)-\theta \left(x\right)\right]}^{2} & =\mathrm{Var}\left\{{\hat{\theta }}_{n}\left(x\right)\right\}+{\left[E{\hat{\theta }}_{n}\left(x\right)-\theta \left(x\right)\right]}^{2}. \end{array}$$

Tonelli’s theorem applies because the integrand is non-negative, so

$${\mathrm{MISE}}_{n}\left(h_{n}\right)=\int_{I}\mathrm{Var}\left\{{\hat{\theta }}_{n}\left(x\right)\right\}\;dx+\int_{I}{\mathrm{Bias}}_{n}^{2}\left(x\right)\;dx.$$

By Proposition 2,

$${\mathrm{Bias}}_{n}\left(x\right)=h_{n}^{\ell /p}B_{s,\ell }\left(x\right)+\rho_{n}\left(x\right),$$

with

$$∥\rho_{n}{∥}_{L^{2}\left(I\right)}=o\left(h_{n}^{\ell /p}\right)$$

by (I.3). Therefore,

$$\begin{array}{cc} \int_{I}{\mathrm{Bias}}_{n}^{2} & =h_{n}^{2\ell /p}\int_{I}B_{s,\ell }^{2}+2h_{n}^{\ell /p}\int_{I}B_{s,\ell }\rho_{n}+\int_{I}\rho_{n}^{2}. \end{array}$$

Cauchy–Schwarz yields

$$\left|\int_{I}B_{s,\ell }\rho_{n}\right|\leq ∥B_{s,\ell }{∥}_{2}{∥\rho_{n}∥}_{2}=o\left(h_{n}^{\ell /p}\right),$$

and hence,

$$\int_{I}{\mathrm{Bias}}_{n}^{2}=h_{n}^{2\ell /p}B_{s,\ell }+o\left(h_{n}^{2\ell /p}\right).$$

For the variance, write

$${\hat{\theta }}_{n}-E{\hat{\theta }}_{n}=M_{n,s}+P_{n,s}.$$

Thus,

$$\begin{array}{cc} \mathrm{Var}({\hat{\theta }}_{n}) & =EM_{n,s}^{2}+EP_{n,s}^{2}+2E\left[M_{n,s}P_{n,s}\right]. \end{array}$$

The martingale variance calculation, together with the uniform-integrability requirement in (I.1), yields

$$nh_{n}^{1+2|s|/p}EM_{n,s}^{2}\left(x\right)⟶q_{\psi }\left(x\right)R_{s}\left(K\right)$$

for x∈I, with a family for which passage under the integral is justified. Therefore,

$$\begin{array}{cc} \int_{I}EM_{n,s}^{2}\left(x\right)\;dx & =\frac{R_{s}\left(K\right)\int_{I}q_{\psi }\left(x\right)\;dx}{nh_{n}^{1+2|s|/p}}\\ & \;+o\left(\frac{1}{nh_{n}^{1+2|s|/p}}\right). \end{array}$$

Assumption (I.2) makes the integrated predictable variance and the integrated covariance negligible at the same scale. Hence,

$$\int_{I}\mathrm{Var}\left\{{\hat{\theta }}_{n}\left(x\right)\right\}\;dx=\frac{V_{s,\psi }}{nh_{n}^{1+2|s|/p}}+o\left(\frac{1}{nh_{n}^{1+2|s|/p}}\right).$$

Adding the bias and variance expansions proves (64).

To determine the AMISE minimizer explicitly, consider the non-degenerate case $B_{s,\ell }>0$ and $V_{s,\psi }>0$, which is the regime in which the stated interior bandwidth formula is meaningful. Set

$$a=\frac{2\ell }{p},\;b=1+\frac{2|s|}{p},\;A=B_{s,\ell },\;V=V_{s,\psi }.$$

The leading risk is

$$R_{n}\left(h\right)=Ah^{a}+\frac{V}{nh^{b}}.$$

Its derivative is

$$R_{n}^{\prime }\left(h\right)=aAh^{a-1}-\frac{bV}{n}h^{-b-1}.$$

The unique critical point satisfies

$$aAh^{a+b}=\frac{bV}{n};$$

hence,

$$h={\left(\frac{bV}{aAn}\right)}^{1/(a+b)}={\left\{\frac{\left(p+2|s|\right)V_{s,\psi }}{2\ell B_{s,\ell }n}\right\}}^{p/(p+2|s|+2\ell )}.$$

At the critical point, the first-order condition implies that

$$\frac{V}{n}h^{-b-2}=\frac{a}{b}Ah^{a-2}.$$

Consequently,

$$\begin{array}{cc} R_{n}^{\prime \prime }\left(h\right) & =a\left(a-1\right)Ah^{a-2}+\frac{b(b+1)V}{n}h^{-b-2}\\ & =a\left(a+b\right)Ah^{a-2}>0. \end{array}$$

Thus, the critical point is the unique minimizer of the leading AMISE. If the leading integrated bias constant *A* vanishes, the displayed AMISE expansion remains valid but does not determine an interior bias–variance optimum at this order; the first non-vanishing higher-order bias term must then be used. The variance-degenerate case is excluded by the corresponding non-degeneracy assumption. □

### 7.11. Variance Estimation and Propensity-Score Replacement

This subsection addresses the two operations required to move from the oracle limit theory toward feasible inference: consistent estimation of the local asymptotic variance, and replacement of the true propensity function by an estimated one. The first argument is an order-zero kernel consistency reduction for the conditional second-moment density; the second is a deterministic perturbation bound that makes the bandwidth amplification of propensity-estimation error explicit.

**Proof of Proposition 4.**  Write

$${\hat{q}}_{\psi ,n}\left(x,c_{n}\right)=\frac{1}{nc_{n}}\sum_{i=1}^{n}W_{i}^{2}\left(\psi \right)L\left(\frac{x-X_{i}}{c_{n}^{1/p}}\right).$$

This is an order-zero kernel estimator whose conditional first-moment density is

$$q_{\psi }\left(u\right)=f_{X}\left(u\right)E\left[W_{0}^{2}\left(\psi \right)|X_{0}=u\right].$$

The assumed order-zero pointwise consistency theorem, applied to the transformed response $W_{i}^{2}\left(\psi \right)$, gives

$${\hat{q}}_{\psi ,n}\left(x,c_{n}\right)\overset{P}{\to }q_{\psi }\left(x\right).$$

The stronger moment condition on $W_{i}^{2}$ supplies the moment hypothesis required by that consistency theorem.

Since $R_{s}\left(K\right)$ is deterministic,

$${\hat{\sigma }}_{\psi ,s}^{2}\left(x\right)=R_{s}\left(K\right){\hat{q}}_{\psi ,n}\left(x,c_{n}\right)\overset{P}{\to }R_{s}\left(K\right)q_{\psi }\left(x\right)=\sigma_{\psi ,s}^{2}\left(x\right).$$

□

**Proof of Proposition 5.**  On the event $\left\{\delta_{n}\leq \pi_{★}/2\right\}$,

$${\hat{\pi }}_{n}^{†}\left(u\right)\geq \pi \left(u\right)-\delta_{n}\geq \frac{\pi_{★}}{2}$$

for every u∈J. Therefore,

$$\begin{array}{cc} \left|\frac{1}{{\hat{\pi }}_{n}^{†}\left(u\right)}-\frac{1}{\pi (u)}\right| & =\frac{|{\hat{\pi }}_{n}^{†}\left(u\right)-\pi \left(u\right)|}{{\hat{\pi }}_{n}^{†}\left(u\right)\pi \left(u\right)}\\ & \leq \frac{2\delta_{n}}{\pi_{★}^{2}}. \end{array}$$

Using the exact oracle–feasible decomposition,

$$\begin{array}{cc} & \left|D^{s}{\hat{r}}_{n}^{\;\mathrm{IPW}}\left(\psi ;x,h_{n}\right)-D^{s}r_{n}^{\;\mathrm{IPW}}\left(\psi ;x,h_{n}\right)\right|\\ & \leq \frac{2\delta_{n}}{\pi_{★}^{2}}\frac{1}{nh_{n}^{1+|s|/p}}\sum_{i=1}^{n}\xi_{i}\left|\psi \left(Y_{i}\right)\right|\left|D^{s}K\left(\frac{x-X_{i}}{b_{n}}\right)\right|\\ & =\frac{2\delta_{n}}{\pi_{★}^{2}}h_{n}^{-|s|/p}\left[\frac{1}{nh_{n}}\sum_{i=1}^{n}\xi_{i}\left|\psi \left(Y_{i}\right)\right|\left|D^{s}K\left(\frac{x-X_{i}}{b_{n}}\right)\right|\right]. \end{array}$$

Taking the supremum over x∈I proves (74). If $A_{n,\psi ,s}=O_{P}\left(1\right)$, multiplication by $\delta_{n}h_{n}^{-|s|/p}$ proves (75). □

### 7.12. Deterministic Quotient Calculus and Exact Differential Remainders

Before propagating the primitive stochastic limits through the regression functional, we establish the deterministic differential calculus of the quotient map. This isolates the purely analytic identities from the probabilistic approximation and makes the nonlinear remainder structure explicit.

**Lemma 24** 
(Multi-index Leibniz formula). *Let $a,b\in C^{\left|s\right|}\left(U\right)$ on an open set $U\subset R^{p}$. Then,*

(206) Ds(ab)=∑j≤ssjDjaDs−jb.

**Proof.**  We proceed by induction on |s|. The case s=0 is immediate. Suppose that it holds for s, and let $e_{k}$ be a coordinate multi-index. Differentiate (206) with respect to $x_{k}$:

Ds+ek(ab)=∑j≤ssjDj+ekaDs−jb+∑j≤ssjDjaDs−j+ekb.

In the first sum, set $l=j+e_{k}$; in the second, set l=j. For every admissible l, the combined coefficient is

sl−ek+sl=s+ekl,

where the identity reduces coordinate-wise to Pascal’s formula. This proves the result for $s+e_{k}$, completing the induction. □

**Lemma 25** 
(Reciprocal derivative recursion). *Let $f\in C^{\left|\alpha \right|}\left(U\right)$ satisfy $\inf_{U}\left|f\right|>0$. Put*

$$Q_{0}=f^{-1}.$$

*For every nonzero multi-index **α**,*

(207) Qα=−1f∑0<β≤ααβDβfQα−β.

*Then,*

$$Q_{\alpha }=D^{\alpha }\left(f^{-1}\right).$$

**Proof.**  The identity

$$ff^{-1}=1$$

implies, for every nonzero α,

$$0=D^{\alpha }\left(ff^{-1}\right).$$

By Lemma 24,

0=∑β≤ααβDβfDα−β(f−1)=fDα(f−1)+∑0<β≤ααβDβfDα−β(f−1).

Division by *f* gives the recursion. Conversely, induction on |α| shows that the recursively defined quantity equals the derivative, because every term on the right involves a reciprocal derivative of strictly smaller total order. □

**Verification of** (81)–(83). For p=1,

$$m_{\psi }=r_{\psi }f^{-1}.$$

The product rule gives

$$m_{\psi }^{\prime }=r_{\psi }^{\prime }f^{-1}+r_{\psi }{\left(f^{-1}\right)}^{\prime }.$$

Since

$${\left(f^{-1}\right)}^{\prime }=-f^{-2}f^{\prime },$$

$$m_{\psi }^{\prime }=\frac{r_{\psi }^{\prime }}{f}-\frac{r_{\psi }f^{\prime }}{f^{2}},$$

which is (81).

Differentiate once more:

$$\begin{array}{cc} m_{\psi }^{\prime \prime } & =\frac{r_{\psi }^{\prime \prime }}{f}-\frac{r_{\psi }^{\prime }f^{\prime }}{f^{2}}-\left[\frac{r_{\psi }^{\prime }f^{\prime }}{f^{2}}+\frac{r_{\psi }f^{\prime \prime }}{f^{2}}-\frac{2r_{\psi }{\left(f^{\prime }\right)}^{2}}{f^{3}}\right]\\ & =\frac{r_{\psi }^{\prime \prime }}{f}-\frac{2r_{\psi }^{\prime }f^{\prime }}{f^{2}}+\frac{r_{\psi }\left\{2{\left(f^{\prime }\right)}^{2}-ff^{\prime \prime }\right\}}{f^{3}}, \end{array}$$

which is (82).

For general *k*, the ordinary Leibniz formula gives

(rψf−1)(k)=∑j=0kkjrψ(j)(f−1)(k−j),

which is (83). The univariate reciprocal recursion is the one-dimensional instance of Lemma 25.

The Bell-polynomial expression (86) follows from the one-dimensional Faà di Bruno formula applied to

$$g\left(u\right)=u^{-1}.$$

Since

$$g^{\left(\nu \right)}\left(u\right)={\left(-1\right)}^{\nu }\nu !u^{-\nu -1},$$

Faà di Bruno gives

$$\begin{array}{cc} \frac{d^{a}}{dx^{a}}g\left(f\left(x\right)\right) & =\sum_{\nu =1}^{a}g^{\left(\nu \right)}\left(f\left(x\right)\right)B_{a,\nu }\left(f^{\prime }\left(x\right),\ldots ,f^{\left(a-\nu +1\right)}\left(x\right)\right)\\ & =\sum_{\nu =1}^{a}{\left(-1\right)}^{\nu }\nu !f{\left(x\right)}^{-\nu -1}B_{a,\nu }\left(f^{\prime }\left(x\right),\ldots ,f^{\left(a-\nu +1\right)}\left(x\right)\right). \end{array}$$

□

**Verification of** (87) **and** (89). Apply Lemma 24 to

$$m\left(\psi ;x\right)=r\left(\psi ;x\right)f_{X}^{-1}\left(x\right).$$

This gives

Dsm=∑j≤ssjDjrDs−j(fX−1),

which is (87). Lemma 25, with $f=f_{X}$, gives (89). □

**Lemma 26** 
(Rational structure of the quotient map). *For every fixed multi-index s, there are finitely many polynomials $P_{s,\nu }$ in the primitive derivative coordinates such that*

(208) $$T_{s}\left(a,b\right)=\sum_{\nu =1}^{|s|+1}\frac{P_{s,\nu }\left({\left(a_{\alpha }\right)}_{\alpha \leq s},{\left(b_{\alpha }\right)}_{0<\alpha \leq s}\right)}{b_{0}^{\;\nu }}.$$

*Consequently, $T_{s}$ is real analytic on the open set $\left\{b_{0}>0\right\}$. On every compact set on which $b_{0}\geq c>0$, all derivatives of every finite order are bounded.*

**Proof.**  The proof is by induction on |α| in the reciprocal recursion. For α=0, $Q_{0}=b_{0}^{-1}$. Suppose that every $Q_{\gamma }$, with |γ|<|α|, is a finite sum of polynomials divided by powers of $b_{0}$ no larger than |γ|+1. The recursion

Qα=−b0−1∑0<β≤ααβbβQα−β

adds one power of $b_{0}^{-1}$, so the maximal denominator power is at most |α|+1. Substitution into the finite Leibniz sum for $T_{s}$ proves (208). Analyticity and boundedness of derivatives on compact denominator-separated sets follow by ordinary finite-dimensional calculus. □

**Lemma 27** 
(Exact first- and second-derivative Taylor remainders). *Let*

$$z_{1}=\left(a_{0},a_{1},b_{0},b_{1}\right),\;\Phi_{1}\left(z_{1}\right)=a_{1}b_{0}^{-1}-a_{0}b_{1}b_{0}^{-2},$$

*and*

$$z_{2}=\left(a_{0},a_{1},a_{2},b_{0},b_{1},b_{2}\right),$$

$$\Phi_{2}\left(z_{2}\right)=a_{2}b_{0}^{-1}-2a_{1}b_{1}b_{0}^{-2}+a_{0}\left(2b_{1}^{2}-b_{0}b_{2}\right)b_{0}^{-3}.$$

*Let K be a convex compact subset of the domain on which $b_{0}\geq c>0$. Then, for every z,z+e∈K,*

$$\begin{array}{cc} \Phi_{j}\left(z+e\right)-\Phi_{j}\left(z\right) & =\nabla \Phi_{j}{\left(z\right)}^{⊤}e+R_{j}\left(z,e\right), \end{array}$$

*where*

(209) $$|R_{j}\left(z,e\right)|\leq \frac{1}{2}\underset{u\in K}{\sup}∥\nabla^{2}\Phi_{j}{\left(u\right)∥}_{\mathrm{op}}{∥e∥}_{2}^{2}.$$

*The gradients are exactly those displayed in* (115) *and* (117).

**Proof.**  The multivariate Taylor formula along the segment t↦z+te gives

$$\begin{array}{cc} \Phi_{j}\left(z+e\right)-\Phi_{j}\left(z\right) & =\int_{0}^{1}\nabla \Phi_{j}{\left(z+te\right)}^{⊤}e\;dt\\ & =\nabla \Phi_{j}{\left(z\right)}^{⊤}e\\ & \;+\int_{0}^{1}\left(1-t\right)e^{⊤}\nabla^{2}\Phi_{j}\left(z+te\right)e\;dt. \end{array}$$

Taking absolute values proves (209).

For $\Phi_{1}$, direct differentiation gives

$$\frac{\partial \Phi_{1}}{\partial a_{0}}=-\frac{b_{1}}{b_{0}^{2}},\;\frac{\partial \Phi_{1}}{\partial a_{1}}=\frac{1}{b_{0}},$$

$$\frac{\partial \Phi_{1}}{\partial b_{0}}=-\frac{a_{1}}{b_{0}^{2}}+\frac{2a_{0}b_{1}}{b_{0}^{3}},\;\frac{\partial \Phi_{1}}{\partial b_{1}}=-\frac{a_{0}}{b_{0}^{2}}.$$

Substitution of

$$\left(a_{0},a_{1},b_{0},b_{1}\right)=\left(r_{\psi },r_{\psi }^{\prime },f,f^{\prime }\right)$$

gives every coefficient in (115).

For $\Phi_{2}$,

$$\frac{\partial \Phi_{2}}{\partial a_{0}}=\frac{2b_{1}^{2}-b_{0}b_{2}}{b_{0}^{3}},\;\frac{\partial \Phi_{2}}{\partial a_{1}}=-\frac{2b_{1}}{b_{0}^{2}},\;\frac{\partial \Phi_{2}}{\partial a_{2}}=\frac{1}{b_{0}}.$$

For the denominator derivatives, first rewrite

$$\Phi_{2}=a_{2}b_{0}^{-1}-2a_{1}b_{1}b_{0}^{-2}+2a_{0}b_{1}^{2}b_{0}^{-3}-a_{0}b_{2}b_{0}^{-2}.$$

Termwise differentiation gives

$$\begin{array}{cc} \frac{\partial \Phi_{2}}{\partial b_{0}} & =-a_{2}b_{0}^{-2}+4a_{1}b_{1}b_{0}^{-3}-6a_{0}b_{1}^{2}b_{0}^{-4}+2a_{0}b_{2}b_{0}^{-3},\\ \frac{\partial \Phi_{2}}{\partial b_{1}} & =-2a_{1}b_{0}^{-2}+4a_{0}b_{1}b_{0}^{-3},\\ \frac{\partial \Phi_{2}}{\partial b_{2}} & =-a_{0}b_{0}^{-2}. \end{array}$$

Substitution of the population coordinates proves (117). Boundedness of the Hessians on K follows from Lemma 26. □

**Proof of** (115)–(120).  Apply Lemma 27 pointwise with the error vector formed from the primitive numerator and density estimators. On the denominator-stable event, all line segments between population and estimated primitive vectors remain in a fixed compact set with the denominator bounded below. The Hessian bound therefore holds uniformly over the evaluation region. Since all Euclidean norms are equivalent in finite dimension,

$${∥e\left(x\right)∥}_{2}\leq C\max_{j}\left\{|e_{r,j}\left(x\right)|+|e_{f,j}\left(x\right)|\right\},$$

which proves (116) and (119).

For general s, apply the same segment Taylor formula to the finite-dimensional analytic map $T_{s}$. Lemma 26 provides a uniform bound for its Hessian on compact denominator-stable sets, yielding

$$|R_{s,n}\left(x\right)|\leq C_{s}{∥{\hat{z}}_{n}\left(x\right)-z\left(x\right)∥}_{2}^{2}.$$

This is (120) with the asserted quadratic remainder. □

### 7.13. Quotient Calculus and Uniform Regression-Derivative Consistency

The deterministic quotient identities are now combined with the primitive uniform stochastic bounds. Since $f_{X}$ is bounded away from zero on the interior set under consideration, the quotient map is locally smooth on a deterministic neighbourhood of the true primitive derivative vector. This permits uniform consistency of regression derivatives to be obtained by a finite-dimensional local-Lipschitz argument rather than by differentiating a random ratio directly.

**Proof of Lemma 1.**  Let

$$I_{s}=\left\{\alpha :\alpha \leq s\right\}.$$

At every x, collect the primitive derivatives into finite vectors

$$z\left(x\right)=\left\{r\left(x\right),f\left(x\right)\right\},\;{\hat{z}}_{n}\left(x\right)=\left\{{\hat{r}}_{n}\left(x\right),{\hat{f}}_{n}\left(x\right)\right\}.$$

By denominator separation,

$$f_{X}\left(x\right)\geq f_{★}>0\;\left(x\in I\right).$$

Choose $\varepsilon_{s}\leq f_{★}/2$. On the event in the lemma,

$${\hat{f}}_{n}^{\left(0\right)}\left(x\right)\geq f_{★}-\varepsilon_{s}\geq f_{★}/2.$$

All primitive coordinates of z(x) are bounded on I, and the estimated coordinates lie in a fixed bounded enlargement. The finite-dimensional map $T_{s}$ is continuously differentiable on this compact set. Hence,

$$C_{s}=\underset{z\in K_{s}}{\sup}{∥DT_{s}\left(z\right)∥}_{\mathrm{op}}<\infty .$$

The multivariate mean-value formula gives

$$\begin{array}{cc} & T_{s}\left({\hat{z}}_{n}\left(x\right)\right)-T_{s}\left(z\left(x\right)\right)\\ & =\int_{0}^{1}DT_{s}\left(z\left(x\right)+t[{\hat{z}}_{n}\left(x\right)-z\left(x\right)]\right)\left[{\hat{z}}_{n}\left(x\right)-z\left(x\right)\right]\;dt. \end{array}$$

Therefore,

$$\begin{array}{cc} & \left|D^{s}{\hat{m}}_{\psi ,n}^{\;\mathrm{IPW}}\left(x\right)-D^{s}m\left(\psi ;x\right)\right|\\ & \leq C_{s}\sum_{\alpha \leq s}\left[\left|{\hat{r}}_{\psi ,n}^{(\alpha ),\mathrm{IPW}}\left(x\right)-D^{\alpha }r\left(\psi ;x\right)\right|+\left|{\hat{f}}_{n}^{\left(\alpha \right)}\left(x\right)-D^{\alpha }f_{X}\left(x\right)\right|\right]. \end{array}$$

Taking the supremum proves (110). □

**Proof of Corollary 3.**  Apply Lemma 1 with s=1 and s=2. Uniform consistency of the density estimator of order zero implies that

$$P(E_{n})⟶1$$

or, under almost-sure primitive convergence, $1_{E_{n}}\to 1$ almost surely. On $E_{n}$, trimming is inactive and the local Lipschitz bound applies. Substitution of the primitive error bounds gives (111) and (112). □

**Proof of Corollary 4.**  The index set $I_{s}$ is finite. Every primitive error in (110) converges uniformly to zero in the stated mode. Their finite sum therefore converges to zero in the same mode. □

### 7.14. Quantitative Control of Nonlinear Remainders

The regression-derivative limit theory requires the nonlinear remainder of the quotient map to be negligible relative to the target stochastic normalization. The following bounds make this requirement quantitative and reduce it to explicit rates for the primitive derivative vector:

**Lemma 28** 
(Primitive maximal error and quadratic quotient remainder). *Let*

$$a_{n,s}=\max_{\alpha \leq s}\left[{\left(nh_{n}^{1+2|\alpha |/p}\right)}^{-1/2}+h_{n}^{\ell /p}\right].$$

*Assume denominator separation and suppose that, for every α≤s,*

$${\hat{r}}_{\psi ,n}^{(\alpha ),\mathrm{IPW}}\left(x\right)-D^{\alpha }r\left(\psi ;x\right)=O_{P}\left[{\left(nh_{n}^{1+2|\alpha |/p}\right)}^{-1/2}+h_{n}^{\ell /p}\right],$$

*and for every α≤s,*

$${\hat{f}}_{n}^{\left(\alpha \right)}\left(x\right)-D^{\alpha }f_{X}\left(x\right)=O_{P}\left[{\left(nh_{n}^{1+2|\alpha |/p}\right)}^{-1/2}+h_{n}^{\ell /p}\right].$$

*Then,*

$$R_{s,n}\left(x\right)=O_{P}\left(a_{n,s}^{2}\right).$$

*Since the highest derivative has the slowest stochastic rate,*

$$a_{n,s}=O\left[{\left(nh_{n}^{1+2|s|/p}\right)}^{-1/2}+h_{n}^{\ell /p}\right].$$

*Consequently,*

(210) $$\begin{array}{cc} \; & \sqrt{nh_{n}^{1+2|s|/p}}R_{s,n}\left(x\right)\\ \; & =O_{P}\left[\frac{1}{\sqrt{nh_{n}^{1+2|s|/p}}}+h_{n}^{\ell /p}+\sqrt{nh_{n}^{1+2|s|/p}}h_{n}^{2\ell /p}\right]. \end{array}$$

*In particular, the normalized remainder is $o_{P}\left(1\right)$ if*

$$nh_{n}^{1+2|s|/p}\to \infty ,\;h_{n}^{\ell /p}\to 0,\;\sqrt{nh_{n}^{1+2|s|/p}}h_{n}^{2\ell /p}\to 0.$$

*Under undersmoothing* (H.3)*, the last condition holds automatically because*

$$\sqrt{nh_{n}^{1+2|s|/p}}h_{n}^{2\ell /p}=h_{n}^{\ell /p}\left[\sqrt{nh_{n}^{1+2|s|/p}}h_{n}^{\ell /p}\right]⟶0.$$

**Proof.**  By Lemma 26, the Hessian of $T_{s}$ is bounded on the compact denominator-stable set containing the population and estimated primitive vectors with probability tending to one. The second-order Taylor remainder therefore satisfies

$$|R_{s,n}\left(x\right)|\leq C{∥{\hat{z}}_{n}\left(x\right)-z\left(x\right)∥}_{2}^{2}.$$

The primitive vector has finitely many coordinates, so its Euclidean norm is bounded by a fixed multiple of the largest coordinate error. This gives $R_{s,n}=O_{P}\left(a_{n,s}^{2}\right)$.

Let

$$N_{n,s}=nh_{n}^{1+2|s|/p}.$$

Then,

$$a_{n,s}^{2}=O\left(N_{n,s}^{-1}+h_{n}^{2\ell /p}+N_{n,s}^{-1/2}h_{n}^{\ell /p}\right).$$

Multiplication by $N_{n,s}^{1/2}$ yields (210). □

**Lemma 29** 
(Expectation remainder bound). *Under the hypotheses of Lemma 28, suppose additionally that the primitive squared errors satisfy the corresponding $L^{1}$ bounds uniformly on I. Then,*

$$\underset{x\in I}{\sup}E\left|R_{s,n}\left(x\right)\right|\leq C\left[\frac{1}{nh_{n}^{1+2|s|/p}}+h_{n}^{2\ell /p}\right].$$

*Therefore, the assumption*

$$\underset{x\in I}{\sup}E\left|R_{s,n}\left(x\right)\right|=o\left(h_{n}^{\ell /p}\right)$$

*used in Proposition 7 follows from*

$$nh_{n}^{1+2|s|/p+\ell /p}⟶\infty .$$

**Proof.**  The deterministic Hessian inequality gives

$$E|R_{s,n}\left(x\right)|\leq CE{∥{\hat{z}}_{n}\left(x\right)-z\left(x\right)∥}_{2}^{2}.$$

Sum the finitely many primitive mean-square bounds. The largest variance term is the one corresponding to total derivative order |s|, and all bias squares are $O(h_{n}^{2\ell /p})$. This proves the first assertion. Now,

$$\frac{{\left(nh_{n}^{1+2|s|/p}\right)}^{-1}}{h_{n}^{\ell /p}}=\frac{1}{nh_{n}^{1+2|s|/p+\ell /p}}⟶0,$$

while

$$\frac{h_{n}^{2\ell /p}}{h_{n}^{\ell /p}}=h_{n}^{\ell /p}⟶0.$$

□

### 7.15. Regression-Derivative Bias Propagation

We now propagate the primitive kernel bias through the nonlinear quotient map. The relevant object is the Fréchet derivative of the finite-dimensional map that sends the collection of numerator and density derivatives to $D^{s}m$. The leading regression-derivative bias is therefore a deterministic linear image of the primitive bias vector; all genuinely nonlinear contributions are isolated in a second-order remainder controlled in Section 7.14.

**Proof of Proposition 7.**  Let

$$z\left(x\right)=\left\{r\left(x\right),f\left(x\right)\right\},\;{\hat{z}}_{n}\left(x\right)=\left\{{\hat{r}}_{n}\left(x\right),{\hat{f}}_{n}\left(x\right)\right\}.$$

On denominator-stable events, the second-order Taylor formula gives

$$\begin{array}{cc} & T_{s}\left({\hat{z}}_{n}\right)-T_{s}\left(z\right)\\ & =DT_{s}\left(z\right)\left[{\hat{z}}_{n}-z\right]+R_{s,n}, \end{array}$$

with

$$|R_{s,n}\left(x\right)|\leq C∥{\hat{z}}_{n}{\left(x\right)-z\left(x\right)∥}^{2}.$$

Taking expectations,

$$\begin{array}{cc} & ED^{s}{\hat{m}}_{\psi ,n}^{\;\mathrm{IPW}}\left(x\right)-D^{s}m\left(\psi ;x\right)\\ & =DT_{s}\left(z\left(x\right)\right)\left[E{\hat{z}}_{n}\left(x\right)-z\left(x\right)\right]+ER_{s,n}\left(x\right). \end{array}$$

The primitive bias expansions give

$$\begin{array}{cc} E{\hat{r}}_{n}-r & =h_{n}^{\ell /p}{\left(D^{\alpha }\beta_{r,\ell }\right)}_{\alpha \leq s}+o\left(h_{n}^{\ell /p}\right),\\ E{\hat{f}}_{n}-f & =h_{n}^{\ell /p}{\left(D^{\alpha }\beta_{f,\ell }\right)}_{\alpha \leq s}+o\left(h_{n}^{\ell /p}\right), \end{array}$$

uniformly on I. To identify the linear image explicitly, introduce the deterministic path

$$r_{t}=r+t\beta_{r,\ell },\;f_{t}=f_{X}+t\beta_{f,\ell }.$$

For |t| sufficiently small, denominator separation implies $\inf_{I}f_{t}>0$. By the deterministic quotient calculus already established,

$$T_{s}\left({\left(D^{\alpha }r_{t}\right)}_{\alpha \leq s},{\left(D^{\alpha }f_{t}\right)}_{\alpha \leq s}\right)=D^{s}\left(\frac{r_{t}}{f_{t}}\right).$$

Differentiating this identity with respect to *t* at t=0 is legitimate because the map is analytic on a denominator-separated neighbourhood and the path is finite-dimensional in the primitive coordinates. Therefore,

$$\begin{array}{cc} & DT_{s}\left(z\right)\left[{\left(D^{\alpha }\beta_{r,\ell }\right)}_{\alpha \leq s},{\left(D^{\alpha }\beta_{f,\ell }\right)}_{\alpha \leq s}\right]\\ & =D^{s}\left[{\left.\frac{d}{dt}\frac{r+t\beta_{r,\ell }}{f_{X}+t\beta_{f,\ell }}\right|}_{t=0}\right]\\ & =D^{s}\left[\frac{\beta_{r,\ell }}{f_{X}}-\frac{r\left(\psi ;\cdot \right)\beta_{f,\ell }}{f_{X}^{2}}\right]=B_{m,s,\ell }. \end{array}$$

By the additional assumption,

$$\underset{x\in I}{\sup}\left|ER_{s,n}\left(x\right)\right|=o\left(h_{n}^{\ell /p}\right).$$

Combining these displays proves (129). □

### 7.16. Highest-Order Reduction and Regression Gaussian Limits

At the central-limit scale associated with the highest derivative order |s|, primitive errors of strictly lower derivative order are asymptotically negligible after common normalization. This scale separation reduces the full Fréchet linearization to two highest-order coordinates: the IPW numerator derivative and the marginal-density derivative. The resulting two-dimensional primitive CLT then yields the Gaussian limit for the regression derivative, with its covariance term retained explicitly.

**Proof of Lemma 3.**  Apply the Fréchet expansion (120). For a primitive derivative indexed by α, its stochastic scale is

$${\left(nh_{n}^{1+2|\alpha |/p}\right)}^{-1/2}.$$

After multiplication by the highest-order normalization,

$$\begin{array}{cc} & \sqrt{nh_{n}^{1+2|s|/p}}{\left(nh_{n}^{1+2|\alpha |/p}\right)}^{-1/2}\\ & =h_{n}^{(|s|-|\alpha |)/p}. \end{array}$$

If |α|<|s|, this factor tends to zero. Since the derivative of $T_{s}$ is bounded on denominator-stable sets, every lower-order centred primitive fluctuation is $o_{P}\left(1\right)$ at the highest-order normalization.

It remains to identify the coefficients of the order-s coordinates. In

Ds(rfX−1)=∑j≤ssjDjrDs−j(fX−1),

the coordinate $D^{s}r$ occurs only in the term j=s, with coefficient $f_{X}^{-1}$. The coordinate $D^{s}f_{X}$ occurs only in $D^{s}\left(f_{X}^{-1}\right)$. Differentiating $f_{X}f_{X}^{-1}=1$ shows that the coefficient of $D^{s}f_{X}$ in this reciprocal derivative is $-f_{X}^{-2}$. Multiplication by the order-zero numerator r(ψ;x) gives the coefficient $-r\left(\psi ;x\right)f_{X}^{-2}\left(x\right)$.

Subtracting the propagated first-order bias from Proposition 7 and invoking the assumed negligibility of the quadratic remainder leaves exactly the two highest-order centred primitive terms displayed in (130). □

**Proof of Theorem 7.**  By Lemma 3, the bias-corrected normalized regression derivative equals

$$a{\left(x\right)}^{⊤}Z_{n}\left(x\right)+o_{P}\left(1\right),$$

where

$$a\left(x\right)=\left(\begin{array}{c} f_{X}^{-1}\left(x\right)\\ -r\left(\psi ;x\right)f_{X}^{-2}\left(x\right) \end{array}\right)$$

and $Z_{n}$ is the normalized, bias-corrected pair consisting of the order-s IPW numerator derivative and the order-s density derivative.

Theorem 5 gives

$$Z_{n}\left(x\right)\overset{D}{\to }N_{2}\left(0,R_{s}\left(K\right)\left(\begin{array}{cc} f_{X}\left(x\right)E\left\{W_{0}^{2}\left(\psi \right)|X_{0}=x\right\} & r(\psi ;x)\\ r(\psi ;x) & f_{X}\left(x\right) \end{array}\right)\right).$$

The off-diagonal entry follows from

$$\begin{array}{cc} f_{X}\left(x\right)E\left[W_{0}\left(\psi \right)\cdot 1|X_{0}=x\right] & =f_{X}\left(x\right)m\left(\psi ;x\right)\\ & =r(\psi ;x). \end{array}$$

The variance of the limiting linear combination is

$$\begin{array}{cc} & R_{s}\left(K\right)a^{⊤}\left(\begin{array}{cc} f_{X}E\left(W_{0}^{2}|X_{0}=x\right) & r\\ r & f_{X} \end{array}\right)a\\ & =R_{s}\left(K\right)\left[\frac{E(W_{0}^{2}|X_{0}=x)}{f_{X}}+\frac{r^{2}}{f_{X}^{3}}-\frac{2r^{2}}{f_{X}^{3}}\right]\\ & =\frac{R_{s}\left(K\right)}{f_{X}\left(x\right)}\left[E\left\{W_{0}^{2}\left(\psi \right)|X_{0}=x\right\}-m^{2}\left(\psi ;x\right)\right]\\ & =\tau_{\psi ,s}^{2}\left(x\right). \end{array}$$

Under sequential MAR,

$$E\left\{W_{0}^{2}\left(\psi \right)|X_{0}=x\right\}=\frac{E\{\psi^{2}\left(Y_{0}\right)|X_{0}=x\}}{\pi (x)}.$$

Thus,

$$\begin{array}{cc} & \sqrt{nh_{n}^{1+2|s|/p}}\left[D^{s}{\hat{m}}_{\psi ,n}^{\;\mathrm{IPW}}\left(x\right)-D^{s}m\left(\psi ;x\right)-h_{n}^{\ell /p}B_{m,s,\ell }\left(x\right)\right]\\ & \overset{D}{\to }N\left(0,\tau_{\psi ,s}^{2}\left(x\right)\right). \end{array}$$

Adding back the deterministic normalized bias gives mean $\lambda B_{m,s,\ell }\left(x\right)$ under (136). Under (138), this mean is zero. □

### 7.17. Direct Local Influence Representation

The preceding two-dimensional reduction can be rewritten as a single localized influence representation. This formulation makes transparent the residual $W_{i}\left(\psi \right)-m\left(\psi ;x\right)$, the role of the fully observed covariate density in the denominator, and the conditional variance that generates the limiting regression-derivative variance. It is also the natural starting point for feasible studentization and orthogonal extensions.

**Proof of** (141). At the highest derivative order, Lemma 3 gives the leading centred primitive combination

$$\begin{array}{cc} L_{n}\left(x\right) & =\frac{1}{f_{X}\left(x\right)}\frac{1}{\sqrt{nh_{n}}}\sum_{i=1}^{n}\left[W_{i}\left(\psi \right)\kappa_{s}\left(\frac{x-X_{i}}{b_{n}}\right)-A_{i,n}^{W}\left(x\right)\right]\\ & \;-\frac{m(\psi ;x)}{f_{X}\left(x\right)}\frac{1}{\sqrt{nh_{n}}}\sum_{i=1}^{n}\left[\kappa_{s}\left(\frac{x-X_{i}}{b_{n}}\right)-A_{i,n}^{1}\left(x\right)\right], \end{array}$$

where

$$A_{i,n}^{W}\left(x\right)=E\left[W_{i}\left(\psi \right)\kappa_{s}\left(\frac{x-X_{i}}{b_{n}}\right)|G_{i-1}\right]$$

and

$$A_{i,n}^{1}\left(x\right)=E\left[\kappa_{s}\left(\frac{x-X_{i}}{b_{n}}\right)|G_{i-1}\right].$$

Collect the observed terms:

$$\begin{array}{cc} & \frac{1}{f_{X}\left(x\right)\sqrt{nh_{n}}}\sum_{i=1}^{n}\left[W_{i}\left(\psi \right)-m\left(\psi ;x\right)\right]\kappa_{s}\left(\frac{x-X_{i}}{b_{n}}\right). \end{array}$$

The remaining predictable centring is

$$\begin{array}{cc} C_{n}\left(x\right) & =\frac{1}{f_{X}\left(x\right)\sqrt{nh_{n}}}\sum_{i=1}^{n}\left[A_{i,n}^{W}\left(x\right)-m\left(\psi ;x\right)A_{i,n}^{1}\left(x\right)\right]. \end{array}$$

Consequently,

$$\begin{array}{cc} L_{n}\left(x\right) & =\frac{1}{f_{X}\left(x\right)\sqrt{nh_{n}}}\sum_{i=1}^{n}\left[W_{i}\left(\psi \right)-m\left(\psi ;x\right)\right]\kappa_{s}\left(\frac{x-X_{i}}{b_{n}}\right)-C_{n}\left(x\right). \end{array}$$

This establishes the exact algebraic representation, including the sign of the predictable centring term.

We next identify the conditional quadratic variation of the leading martingale influence term. Define

$$U_{i}\left(x\right)=W_{i}\left(\psi \right)-m\left(\psi ;x\right).$$

The martingale increment before normalization is

$$\begin{array}{cc} \Gamma_{i,n}\left(x\right) & =\frac{1}{f_{X}\left(x\right)}\left[U_{i}\left(x\right)\kappa_{s}\left(\frac{x-X_{i}}{b_{n}}\right)-E\left\{U_{i}\left(x\right)\kappa_{s}\left(\frac{x-X_{i}}{b_{n}}\right)|G_{i-1}\right\}\right]. \end{array}$$

Thus, the predictable quadratic variation is

$$\frac{1}{nh_{n}}\sum_{i=1}^{n}E\left[\Gamma_{i,n}^{2}\left(x\right)|G_{i-1}\right].$$

As in Lemma 21, the squared conditional-mean term is $o_{P}\left(1\right)$. The leading uncentred conditional second moment is

$$\begin{array}{cc} & \frac{1}{f_{X}^{2}\left(x\right)nh_{n}}\sum_{i=1}^{n}E\left[U_{i}^{2}\left(x\right)\kappa_{s}^{2}\left(\frac{x-X_{i}}{b_{n}}\right)|G_{i-1}\right]\\ & =\frac{1}{f_{X}^{2}\left(x\right)}\int \kappa_{s}^{2}\left(v\right)\left[\frac{1}{n}\sum_{i=1}^{n}q_{i,U}\left(x-b_{n}v\right)\right]\;dv, \end{array}$$

where

$$q_{i,U}\left(u\right)=E\left[{\left\{W_{i}\left(\psi \right)-m\left(\psi ;x\right)\right\}}^{2}|X_{i}=u,G_{i-1}\right]f_{i}\left(u\right).$$

Local stabilization and continuity imply convergence in probability to

$$\begin{array}{cc} & \frac{R_{s}\left(K\right)}{f_{X}^{2}\left(x\right)}f_{X}\left(x\right)E\left[{\left\{W_{0}\left(\psi \right)-m\left(\psi ;x\right)\right\}}^{2}|X_{0}=x\right]\\ & =\frac{R_{s}\left(K\right)}{f_{X}\left(x\right)}\mathrm{Var}\left[W_{0}\left(\psi \right)|X_{0}=x\right]\\ & =\tau_{\psi ,s}^{2}\left(x\right). \end{array}$$

This establishes the variance assertion accompanying (141). □

### 7.18. Consequences for Feasible Propensity Weighting and Studentization

We now translate the oracle–feasible perturbation inequality into explicit asymptotic regimes and combine the regression Gaussian limit with a consistent variance estimator. The key point is that differentiation amplifies propensity error by $h_{n}^{-|s|/p}$, whereas after central-limit normalization this amplification simplifies to the condition $\sqrt{nh_{n}}\;\delta_{n}\to 0$. The resulting statements distinguish consistency transfer, uniform-rate transfer, pointwise oracle equivalence, and studentized inference.

**Lemma 30** 
(Boundedness of the localized absolute kernel average). *Assume the order-zero uniform consistency conditions for the transformed response $\xi_{i}\left|\psi \left(Y_{i}\right)\right|$ and the non-negative kernel $|D^{s}K|$. Then,*

$$A_{n,\psi ,s}=\underset{x\in I}{\sup}\frac{1}{nh_{n}}\sum_{i=1}^{n}\xi_{i}\left|\psi \left(Y_{i}\right)\right|\left|D^{s}K\left(\frac{x-X_{i}}{b_{n}}\right)\right|=O_{P}\left(1\right).$$

*Under the corresponding strong uniform assumptions, the bound holds almost surely.*

**Proof.**  The displayed quantity is an order-zero kernel estimator with response $\xi_{i}\left|\psi \left(Y_{i}\right)\right|$ and kernel $|D^{s}K|$. Its deterministic limit is

$$\begin{array}{cc} a_{\psi ,s}\left(x\right) & =\int_{R^{q}}|\psi \left(y\right)|\pi \left(x\right)f_{X,Y}\left(x,y\right)\;dy\int_{R^{p}}\left|D^{s}K\left(v\right)\right|\;dv. \end{array}$$

This limit is bounded on I by positivity, continuity, the moment assumptions, and compactness. Uniform consistency therefore implies

$$A_{n,\psi ,s}\leq \underset{x\in I}{\sup}\left|a_{\psi ,s}\left(x\right)\right|+o_{P}\left(1\right),$$

which is $O_{P}\left(1\right)$. Under the strong uniform assumptions, the same argument uses the almost-sure uniform approximation in place of the $o_{P}\left(1\right)$ term and yields $A_{n,\psi ,s}=O_{a.s.}\left(1\right)$. □

**Corollary 6** 
(Oracle-equivalence regimes). *Under Proposition 5 and Lemma 30, the following hold:*

1. *If*
  $$\delta_{n}h_{n}^{-|s|/p}\overset{P}{\to }0,$$
  *then the feasible and oracle derivative estimators are asymptotically equivalent for consistency.*
2. *If*
  $$\delta_{n}h_{n}^{-|s|/p}=o_{P}\left[{\left\{\frac{\log n}{nh_{n}^{1+2|s|/p}}\right\}}^{1/2}\right],$$
  *then the feasible estimator has the same uniform stochastic rate as the oracle estimator.*
3. *If*
  $$\sqrt{nh_{n}}\;\delta_{n}\overset{P}{\to }0,$$
  *then*
  $$\begin{array}{cc} & \sqrt{nh_{n}^{1+2|s|/p}}\left[D^{s}{\hat{r}}_{n}^{\;\mathrm{IPW}}-D^{s}r_{n}^{\;\mathrm{IPW}}\right]\\ & =o_{P}\left(1\right), \end{array}$$
  *so the feasible and oracle pointwise central limit theorems coincide.*

**Proof.**  The deterministic comparison gives

$$\begin{array}{cc} & \underset{x\in I}{\sup}\left|D^{s}{\hat{r}}_{n}^{\;\mathrm{IPW}}-D^{s}r_{n}^{\;\mathrm{IPW}}\right|\\ & =O_{P}\left(\delta_{n}h_{n}^{-|s|/p}\right). \end{array}$$

The first two assertions follow directly from the displayed uniform bound. For the pointwise central-limit scale,

$$\begin{array}{cc} & \sqrt{nh_{n}^{1+2|s|/p}}\delta_{n}h_{n}^{-|s|/p}\\ & =\sqrt{nh_{n}}\;\delta_{n}. \end{array}$$

□

**Proof of the studentized limit** (147). Under undersmoothing, Theorem 7 gives

$$Z_{n}\left(x\right):=\frac{\sqrt{nh_{n}^{1+2|s|/p}}\left[D^{s}{\hat{m}}_{\psi ,n}^{\;\mathrm{IPW}}\left(x\right)-D^{s}m\left(\psi ;x\right)\right]}{\tau_{\psi ,s}\left(x\right)}\overset{D}{\to }N\left(0,1\right).$$

Proposition 8 and positivity of $\tau_{\psi ,s}^{2}\left(x\right)$ imply that

$$\frac{{\hat{\tau }}_{\psi ,s}\left(x\right)}{\tau_{\psi ,s}\left(x\right)}\overset{P}{\to }1.$$

Therefore,

$$\begin{array}{cc} & \frac{\sqrt{nh_{n}^{1+2|s|/p}}\left[D^{s}{\hat{m}}_{\psi ,n}^{\;\mathrm{IPW}}\left(x\right)-D^{s}m\left(\psi ;x\right)\right]}{{\hat{\tau }}_{\psi ,s}\left(x\right)}\\ & =Z_{n}\left(x\right)\frac{\tau_{\psi ,s}\left(x\right)}{{\hat{\tau }}_{\psi ,s}\left(x\right)}\overset{D}{\to }N\left(0,1\right) \end{array}$$

by Slutsky’s theorem.

Let $z_{1-\alpha /2}$ denote the corresponding standard-normal quantile. The coverage event for (148) is

$$\begin{array}{cc} & \left\{D^{s}m\left(\psi ;x\right)\in D^{s}{\hat{m}}_{\psi ,n}^{\;\mathrm{IPW}}\left(x\right)\pm z_{1-\alpha /2}\frac{{\hat{\tau }}_{\psi ,s}\left(x\right)}{\sqrt{nh_{n}^{1+2|s|/p}}}\right\}\\ & =\left\{\left|\frac{\sqrt{nh_{n}^{1+2|s|/p}}\left[D^{s}{\hat{m}}_{\psi ,n}^{\;\mathrm{IPW}}\left(x\right)-D^{s}m\left(\psi ;x\right)\right]}{{\hat{\tau }}_{\psi ,s}\left(x\right)}\right|\leq z_{1-\alpha /2}\right\}. \end{array}$$

Since the limiting standard normal distribution is continuous at the two endpoints,

P(coverageevent)⟶1−α.

□

**Bias-corrected studentization under** (149). Write

$$\begin{array}{cc} & \sqrt{nh_{n}^{1+2|s|/p}}\left[D^{s}{\hat{m}}_{\psi ,n}^{\;\mathrm{BC}}\left(x\right)-D^{s}m\left(\psi ;x\right)\right]\\ & =\sqrt{nh_{n}^{1+2|s|/p}}\left[D^{s}{\hat{m}}_{\psi ,n}^{\;\mathrm{IPW}}\left(x\right)-D^{s}m\left(\psi ;x\right)-h_{n}^{\ell /p}B_{m,s,\ell }\left(x\right)\right]\\ & \;-\sqrt{nh_{n}^{1+2|s|/p}}h_{n}^{\ell /p}\left[{\hat{B}}_{m,s,\ell }\left(x\right)-B_{m,s,\ell }\left(x\right)\right]. \end{array}$$

The first term converges to the centred Gaussian law by Theorem 7, whereas the second is $o_{P}\left(1\right)$ by (149). Hence, the bias-corrected numerator has the same centred Gaussian limit. Proposition 8 still gives

$${\hat{\tau }}_{\psi ,s}\left(x\right)/\tau_{\psi ,s}\left(x\right)\overset{P}{\to }1,$$

and a final application of Slutsky’s theorem yields the corresponding bias-corrected studentized N(0,1) limit. □

### 7.19. Complete-Case and Weighted-Denominator Calculations

We finally isolate the population identities underlying the complete-case and alternative-denominator constructions discussed in Section 4. These calculations distinguish equality of population ratio targets from equality of primitive bias and covariance structures.

**Lemma 31** 
(Population targets of complete-case local moments). *Under sequential MAR,*

$$E\left(\xi_{0}|X_{0}=x\right)=\pi \left(x\right)$$

*and*

$$E\left[\xi_{0}\psi \left(Y_{0}\right)|X_{0}=x\right]=\pi \left(x\right)m\left(\psi ;x\right).$$

*Consequently, the unweighted complete-case numerator and denominator have density-weighted targets*

$$r_{\mathrm{cc}}\left(\psi ;x\right)=\pi \left(x\right)r\left(\psi ;x\right)$$

*and*

$$f_{\mathrm{cc}}\left(x\right)=\pi \left(x\right)f_{X}\left(x\right),$$

*respectively. Their order-zero ratio equals m(ψ;x) wherever $\pi \left(x\right)f_{X}\left(x\right)>0$.*

**Proof.**  The first identity is the definition of the propensity score after applying the tower property to the sequential MAR relation. For the second,

$$\begin{array}{cc} & E\left[\xi_{0}\psi \left(Y_{0}\right)|X_{0}\right]\\ & =E\left[E\left(\xi_{0}\psi \left(Y_{0}\right)|X_{0},Y_{0},G_{-1}\right)|X_{0}\right]\\ & =E\left[\psi \left(Y_{0}\right)\pi \left(X_{0}\right)|X_{0}\right]\\ & =\pi \left(X_{0}\right)E\left[\psi \left(Y_{0}\right)|X_{0}\right]. \end{array}$$

Multiplication by the marginal density gives the two density-weighted targets. Their ratio is

$$\frac{\pi (x)r(\psi ;x)}{\pi \left(x\right)f_{X}\left(x\right)}=\frac{r(\psi ;x)}{f_{X}\left(x\right)}=m\left(\psi ;x\right).$$

□

**Lemma 32** 
(Derivative structure of a complete-case ratio). *Let*

$$\tilde{r}=\pi r,\;\tilde{f}=\pi f_{X}.$$

*Although $\tilde{r}/\tilde{f}=r/f_{X}$ exactly at the population level, a primitive derivative construction based on $\tilde{r}$ and $\tilde{f}$ contains derivatives of the propensity. For every multi-index **α**,*

Dαr˜=∑β≤ααβDβπDα−βr

*and*

Dαf˜=∑β≤ααβDβπDα−βfX.

*Thus, the stochastic primitive vector, its bias constants, and its covariance matrix differ from those of the IPW numerator combined with the fully observed density denominator.*

**Proof.**  Both derivative identities follow directly from the multi-index Leibniz formula. At the exact population level, all propensity terms cancel after the complete quotient is simplified. At the estimator level, however, numerator and denominator derivatives are estimated separately, and their smoothing errors and covariance structure depend on the local design density $\pi f_{X}$. Therefore, one cannot obtain the complete-case derivative theory by inserting complete-case primitive estimators into the IPW variance formula. □

**Lemma 33** 
(Population target of an IPW-weighted denominator). *The IPW-weighted local denominator based on $\xi_{i}/\pi \left(X_{i}\right)$ has the same population target $f_{X}$ as the ordinary full-covariate denominator:*

$$E\left[\frac{\xi_{0}}{\pi (X_{0})}|X_{0}\right]=1.$$

*Its local conditional second moment is*

$$E\left[\left.{\left\{\frac{\xi_{0}}{\pi (X_{0})}\right\}}^{2}\right|X_{0}=x\right]=\frac{1}{\pi (x)}.$$

*Hence, its primitive marginal variance is inflated relative to the unweighted denominator. No universal ordering of the final ratio variance follows, because numerator–denominator covariance also changes.*

**Proof.**  Sequential MAR gives

$$\begin{array}{cc} E\left[\frac{\xi_{0}}{\pi (X_{0})}|X_{0}\right] & =\frac{1}{\pi (X_{0})}E\left(\xi_{0}|X_{0}\right)=1. \end{array}$$

Since $\xi_{0}^{2}=\xi_{0}$,

$$\begin{array}{cc} E\left[\left.{\left\{\frac{\xi_{0}}{\pi (X_{0})}\right\}}^{2}\right|X_{0}\right] & =\frac{1}{\pi {\left(X_{0}\right)}^{2}}E\left(\xi_{0}|X_{0}\right)\\ & =\frac{1}{\pi (X_{0})}. \end{array}$$

The absence of a universal ordering follows from the variance identity for a linearized ratio:

$$\mathrm{Var}\left(aU+bV\right)=a^{2}\mathrm{Var}\left(U\right)+b^{2}\mathrm{Var}\left(V\right)+2ab\mathrm{Cov}\left(U,V\right).$$

Increasing the marginal variance of *V* does not determine the total when the covariance changes simultaneously. □

### 7.20. Regression Variance Estimation and AMISE

The section concludes by completing the feasible variance and integrated-risk analysis for regression derivatives. A pilot order-zero estimator consistently recovers the conditional variance of the localized regression influence variable, and the highest-order linear representation transfers the primitive variance expansion to the quotient functional. Combining this variance term with the propagated leading bias yields the regression-derivative AMISE and its optimal bandwidth order.

**Proof of Proposition 8.**  Define the pilot local moments

$${\hat{a}}_{j,n}\left(x\right)=\frac{1}{nc_{n}}\sum_{i=1}^{n}W_{i}^{j}\left(\psi \right)L\left(\frac{x-X_{i}}{c_{n}^{1/p}}\right),\;j=0,1,2,$$

with $W_{i}^{0}=1$. The assumed order-zero consistency gives

$$\begin{array}{cc} {\hat{a}}_{0,n}\left(x\right) & \overset{P}{\to }f_{X}\left(x\right),\\ {\hat{a}}_{1,n}\left(x\right) & \overset{P}{\to }f_{X}\left(x\right)m\left(\psi ;x\right),\\ {\hat{a}}_{2,n}\left(x\right) & \overset{P}{\to }f_{X}\left(x\right)E\left\{W_{0}^{2}\left(\psi \right)|X_{0}=x\right\}. \end{array}$$

Therefore,

$${\hat{m}}_{\psi ,n,L}^{\;\mathrm{IPW}}\left(x;c_{n}\right)=\frac{{\hat{a}}_{1,n}\left(x\right)}{{\hat{a}}_{0,n}\left(x\right)}\overset{P}{\to }m\left(\psi ;x\right).$$

The residual variance estimator satisfies the exact algebraic identity

$$\begin{array}{cc} {\hat{v}}_{\psi ,n}\left(x;c_{n}\right) & =\frac{{\hat{a}}_{2,n}}{{\hat{a}}_{0,n}}-2{\hat{m}}_{\psi ,n,L}^{\;\mathrm{IPW}}\frac{{\hat{a}}_{1,n}}{{\hat{a}}_{0,n}}+{\left({\hat{m}}_{\psi ,n,L}^{\;\mathrm{IPW}}\right)}^{2}\\ & =\frac{{\hat{a}}_{2,n}}{{\hat{a}}_{0,n}}-{\left({\hat{m}}_{\psi ,n,L}^{\;\mathrm{IPW}}\right)}^{2}. \end{array}$$

Slutsky’s theorem gives

$${\hat{v}}_{\psi ,n}\left(x;c_{n}\right)\overset{P}{\to }E\left\{W_{0}^{2}\left(\psi \right)|X_{0}=x\right\}-m^{2}\left(\psi ;x\right)=v_{\psi }\left(x\right).$$

Since the limiting density and variance are strictly positive, trimming and the positive-part operation are asymptotically inactive. Hence,

$$\begin{array}{cc} {\hat{\tau }}_{\psi ,s}^{2}\left(x\right) & =\frac{R_{s}\left(K\right)}{{\hat{f}}_{n,L}\left(x;c_{n}\right)\vee \tau_{n}}\left\{{\hat{v}}_{\psi ,n}\left(x;c_{n}\right)\vee 0\right\}\\ & \overset{P}{\to }\frac{R_{s}\left(K\right)}{f_{X}\left(x\right)}v_{\psi }\left(x\right)=\tau_{\psi ,s}^{2}\left(x\right). \end{array}$$

□

**Proof of Theorem 8.**  Let

$${\hat{\vartheta }}_{n}\left(x\right)=D^{s}{\hat{m}}_{\psi ,n}^{\;\mathrm{IPW}}\left(x\right),\;\vartheta \left(x\right)=D^{s}m\left(\psi ;x\right).$$

Tonelli’s theorem and the bias–variance identity give

$$\begin{array}{cc} {\mathrm{MISE}}_{m,s}\left(h_{n}\right) & =\int_{I}\mathrm{Var}\left\{{\hat{\vartheta }}_{n}\left(x\right)\right\}\;dx\\ & \;+\int_{I}{\left[E{\hat{\vartheta }}_{n}\left(x\right)-\vartheta \left(x\right)\right]}^{2}\;dx. \end{array}$$

By Proposition 7,

$$E{\hat{\vartheta }}_{n}\left(x\right)-\vartheta \left(x\right)=h_{n}^{\ell /p}B_{m,s,\ell }\left(x\right)+\rho_{m,n}\left(x\right),$$

where the integrated remainder assumptions imply

$$∥\rho_{m,n}{∥}_{L^{2}\left(I\right)}=o\left(h_{n}^{\ell /p}\right).$$

The same Cauchy–Schwarz calculation used in the proof of Theorem 6 yields

$$\begin{array}{cc} \int_{I}{\left[E{\hat{\vartheta }}_{n}-\vartheta \right]}^{2} & =h_{n}^{2\ell /p}B_{m,s,\ell }+o\left(h_{n}^{2\ell /p}\right). \end{array}$$

By the highest-order linear representation,

$$\begin{array}{cc} {\hat{\vartheta }}_{n}-E{\hat{\vartheta }}_{n} & =\frac{1}{f_{X}}\left[{\hat{r}}_{\psi ,n}^{(s),\mathrm{IPW}}-E{\hat{r}}_{\psi ,n}^{(s),\mathrm{IPW}}\right]\\ & \;-\frac{r}{f_{X}^{2}}\left[{\hat{f}}_{n}^{\left(s\right)}-E{\hat{f}}_{n}^{\left(s\right)}\right]+R_{n}, \end{array}$$

where the integrated contribution of $R_{n}$ is negligible by assumption. The joint primitive variance expansion therefore gives

$$\begin{array}{cc} & nh_{n}^{1+2|s|/p}\mathrm{Var}\left\{{\hat{\vartheta }}_{n}\left(x\right)\right\}\\ & ⟶\frac{R_{s}\left(K\right)}{f_{X}\left(x\right)}v_{\psi }\left(x\right)=\tau_{\psi ,s}^{2}\left(x\right). \end{array}$$

Uniform integrability permits integration of this convergence:

$$\begin{array}{cc} \int_{I}\mathrm{Var}\left\{{\hat{\vartheta }}_{n}\left(x\right)\right\}\;dx & =\frac{1}{nh_{n}^{1+2|s|/p}}\int_{I}\tau_{\psi ,s}^{2}\left(x\right)\;dx\\ & \;+o\left(\frac{1}{nh_{n}^{1+2|s|/p}}\right)\\ & =\frac{V_{m,\psi ,s}}{nh_{n}^{1+2|s|/p}}+o\left(\frac{1}{nh_{n}^{1+2|s|/p}}\right). \end{array}$$

Adding integrated squared bias and variance proves (154).

For the bandwidth optimization, assume the non-degenerate case

$$B_{m,s,\ell }>0,\;V_{m,\psi ,s}>0.$$

Put

$$a=\frac{2\ell }{p},\;b=1+\frac{2|s|}{p},\;A_{m}=B_{m,s,\ell },\;V_{m}=V_{m,\psi ,s}.$$

The leading regression risk is

$$R_{m,n}\left(h\right)=A_{m}h^{a}+\frac{V_{m}}{nh^{b}}.$$

Differentiation gives

$$R_{m,n}^{\prime }\left(h\right)=aA_{m}h^{a-1}-\frac{bV_{m}}{n}h^{-b-1}.$$

The unique positive critical point therefore satisfies

$$aA_{m}h^{a+b}=\frac{bV_{m}}{n},$$

and hence,

$$h_{m,\mathrm{AMISE}}={\left\{\frac{\left(p+2|s|\right)V_{m,\psi ,s}}{2\ell B_{m,s,\ell }n}\right\}}^{p/(p+2|s|+2\ell )}.$$

At this point,

$$R_{m,n}^{\prime \prime }\left(h\right)=a\left(a-1\right)A_{m}h^{a-2}+\frac{b\left(b+1\right)V_{m}}{n}h^{-b-2}=a\left(a+b\right)A_{m}h^{a-2}>0,$$

where the first-order condition is used in the final equality. Thus, the critical point is the unique minimizer of the leading AMISE. If $B_{m,s,\ell }=0$, the displayed expansion still holds, but the leading-order optimization is degenerate and must be based on the first non-vanishing higher-order bias term. □

## Figures and Tables

**Table 1:** Propensity mechanisms: local range, theoretical mean under $f_{X}$, and empirical response rate (n=1000, N=500).

Mechanism	$\min_{I}\pi$	$\max_{I}\pi$	$E_{f_{X}}\left[\pi \left(X\right)\right]$	Empirical Response Rate
M0 (complete)	1.000	1.000	1.000	1.000
M1a (MCAR, $\pi_{0}=0.8$)	0.800	0.800	0.800	0.800
M1b (MCAR, $\pi_{0}=0.6$)	0.600	0.600	0.600	0.601
M2 (moderate MAR)	0.443	0.751	0.585	0.582
M3 (severe MAR)	0.202	0.803	0.437	0.434
M4 (nonmonotone MAR)	0.300	0.850	0.699	0.702

**Table 2:** Finite-sample accuracy of $D^{s}r_{n}^{\mathrm{IPW}}\left(\psi ;\cdot ,h_{n}\right)$ over *I*, design D1 (i.i.d.), mechanism M2, ψ(y)=y, N=1000, bandwidth B1.

*n*	|s|	Mean |Bias|	Mean sd	RMSE	MISE	Sup-Norm Err.
250	0	0.164	0.123	0.240	0.046	0.439
500	0	0.130	0.093	0.188	0.028	0.345
1000	0	0.106	0.073	0.152	0.019	0.284
2000	0	0.083	0.055	0.118	0.011	0.223
250	1	2.384	0.475	2.851	6.501	4.839
500	1	2.126	0.394	2.537	5.148	4.329
1000	1	1.912	0.338	2.275	4.140	3.897
2000	1	1.678	0.276	1.992	3.173	3.430
250	2	27.47	2.42	31.84	811.1	54.68
500	2	26.13	2.09	30.17	728.2	51.42
1000	2	24.85	1.87	28.61	654.7	48.45
2000	2	23.34	1.58	26.78	573.9	45.14

**Table 3:** Finite-sample accuracy of the plug-in estimators ${\hat{m}}_{\psi ,n}^{\prime ,\mathrm{IPW}}$ and ${\hat{m}}_{\psi ,n}^{\prime \prime ,\mathrm{IPW}}$, design D1, mechanism M2, N=1000.

Target	*n*	Mean |Bias|	RMSE	MISE
${\hat{m}}^{\prime }$	250	1.613	1.921	2.953
${\hat{m}}^{\prime }$	500	1.503	1.796	2.581
${\hat{m}}^{\prime }$	1000	1.398	1.680	2.258
${\hat{m}}^{\prime }$	2000	1.286	1.556	1.938
${\hat{m}}^{\prime \prime }$	250	14.64	15.77	198.9
${\hat{m}}^{\prime \prime }$	500	14.06	15.15	183.5
${\hat{m}}^{\prime \prime }$	1000	13.47	14.52	168.6
${\hat{m}}^{\prime \prime }$	2000	12.84	13.84	153.3

**Table 4:** Fréchet linearization remainder for ${\hat{m}}_{\psi ,n}^{\prime ,\mathrm{IPW}}$, design D1, mechanism M2, N=1000. “Ratio” is mean$|R_{1,n}|/$mean|full error|.

Rule	*n*	Low π (0.15)	Center (0.50)	High π (0.85)
B1	250	0.447	0.174	0.208
B1	500	0.411	0.147	0.194
B1	1000	0.377	0.122	0.201
B1	2000	0.345	0.102	0.203
B4	250	0.412	0.112	0.255
B4	500	0.331	0.086	0.214
B4	1000	0.267	0.068	0.182
B4	2000	0.211	0.051	0.150

**Table 5:** Numerator–denominator covariance decomposition for ${\hat{m}}_{\psi ,n}^{\prime ,\mathrm{IPW}}$, design D1, mechanism M2, N=500. $\mathrm{Cov}(r^{\left(0\right)},f^{\left(0\right)})$ is the numerator–denominator covariance entering the ratio at |s|=0.

*n*	Point	$\mathrm{Var}(r^{\left(0\right)})$	$\mathrm{Var}(f^{\left(0\right)})$	$\mathrm{Cov}(r^{\left(0\right)},f^{\left(0\right)})$	$\mathrm{Var}({\hat{m}}^{\prime })$ Delta	$\mathrm{Var}({\hat{m}}^{\prime })$ Direct MC
1000	low π	0.00450	0.00088	0.00117	0.818	0.175
1000	center	0.00447	0.00116	0.00075	0.042	0.051
1000	high π	0.00053	0.00073	0.00020	0.135	0.034

**Table 6:** Denominator stability, design D1, mechanism M2, bandwidth B1 (|s|=0), N=500, trim threshold $10^{-6}$.

*n*	$h_{n}$	Min. $\hat{f}$ over All Replications	Frac. Replications with a Trim Event	Mean Grid Points Trimmed
250	0.116	0.2558	0	0
500	0.101	0.2441	0	0
1000	0.088	0.2626	0	0
2000	0.077	0.2485	0	0

**Table 7:** Effect of dependence on $D^{0}r_{n}^{\mathrm{IPW}}$, mechanism M2, N=500.

Design	*n*	Mean sd	RMSE	MISE
D1 (i.i.d.)	1000	0.0717	0.1505	0.0181
D2, ρ=0.3	1000	0.0748	0.1510	0.0182
D2, ρ=0.7	1000	0.0859	0.1618	0.0209
D2, ρ=0.9 (strong)	1000	0.1265	0.1930	0.0298
D4 (rotation, non-mixing)	1000	0.0626	0.1455	0.0169

**Table 8:** Variance under six missingness mechanisms, $D^{0}r_{n}^{\mathrm{IPW}}$, design D1, n=1000, N=500.

Mechanism	Mean π	Response Rate	Mean sd	MISE
M0 (complete)	1.000	1.000	0.0487	0.0161
M1a (MCAR, $\pi_{0}=0.8$)	0.800	0.800	0.0576	0.0165
M1b (MCAR, $\pi_{0}=0.6$)	0.600	0.601	0.0657	0.0172
M2 (moderate MAR)	0.582	0.582	0.0723	0.0179
M3 (severe MAR)	0.434	0.434	0.0944	0.0216
M4 (non-monotone MAR)	0.702	0.702	0.0629	0.0164

**Table 9:** Oracle, feasible, and misspecified propensity estimation, target $r_{\psi }\left(x\right)$ (|s|=0) unless noted, design D1, mechanism M2, n=1000, N=500.

Method	Estimand	Bias	sd	MISE
E5 full-response (infeasible)	$r_{\psi }$	0.104	0.049	0.0150
E2 feasible, correctly specified ${\hat{\pi }}_{n}$	$r_{\psi }$	0.109	0.063	0.0179
E1 oracle IPW	$r_{\psi }$	0.105	0.072	0.0181
E3 feasible, misspecified ${\hat{\pi }}_{n}$	$r_{\psi }$	0.167	0.064	0.0389
E4 naive complete-case	$r_{\psi }$	0.436	0.040	0.2707

**Table 10:** Oracle→feasible propensity transfer, correctly-specified logistic ${\hat{\pi }}_{n}$, design D1, mechanism M2, N=500.

*n*	Mean $\delta_{n}$ (sd)	Mean $\delta_{n}h_{n}^{-0}$	Mean $\sqrt{{\mathrm{nh}}_{n}}\;\delta_{n}$ (sd)
250	0.0818 (0.0414)	0.0818	0.440 (0.223)
500	0.0641 (0.0298)	0.0641	0.456 (0.212)
1000	0.0486 (0.0216)	0.0486	0.456 (0.203)
2000	0.0410 (0.0161)	0.0410	0.507 (0.200)

**Table 11:** Comparison of bandwidth rules for $D^{0}r_{n}^{\mathrm{IPW}}$, design D1, mechanism M2.

*n*	h(B1)	$\bar{h}\;$(B2, Oracle)	MISE (B1)	MISE (B2)	$\bar{h}\;$(B3, CV)
250	0.116	0.074	0.0471	0.0290	0.186
500	0.101	0.063	0.0281	0.0161	0.213
1000	0.088	0.054	0.0183	0.0101	0.223
2000	0.077	0.047	0.0114	0.0060	—

**Table 12:** Studentized-statistic centring and coverage for $D^{0}r_{n}^{\mathrm{IPW}}$: AMISE-type bandwidth (B1) vs. undersmoothed bandwidth (B4, δ=0.05), design D1, mechanism M2, n=1000, N=1000.

Rule	Point	$h_{n}$	Mean (Stat)	Cov. 90%	Cov. 95%
B1	low π	0.0879	1.353	0.619	0.765
B1	center	0.0879	−1.043	0.748	0.852
B1	high π	0.0879	0.001	0.917	0.957
B4	low π	0.0622	0.568	0.888	0.944
B4	center	0.0622	−0.448	0.898	0.950
B4	high π	0.0622	−0.229	0.874	0.930

**Table 13:** Studentized inference for ${\hat{m}}_{\psi ,n}^{\prime ,\mathrm{IPW}}$, design D1, mechanism M2, N=1000. “SE ratio” is mean estimated SE divided by empirical sd of ${\hat{m}}^{\prime }$ across replications.

Rule	*n*	Point	Mean (Stat)	SE Ratio	Cov. 90%	Cov. 95%
B1	1000	low π	−7.44	0.990	0.000	0.000
B1	1000	center	7.08	0.984	0.000	0.000
B1	1000	high π	−11.98	0.950	0.000	0.000
B1	2000	low π	−8.14	0.963	0.000	0.000
B1	2000	center	7.65	1.009	0.000	0.000
B1	2000	high π	−13.37	0.998	0.000	0.000
B4	1000	low π	−3.04	0.997	0.064	0.118
B4	1000	center	2.73	1.012	0.144	0.223
B4	1000	high π	−5.14	0.956	0.003	0.006
B4	2000	low π	−2.96	1.030	0.074	0.143
B4	2000	center	2.63	0.996	0.174	0.257
B4	2000	high π	−5.01	0.993	0.001	0.004

**Table 14:** Empirical log–log MISE-vs-*n* slopes, n∈{250,500,1000,2000}, design D1, mechanism M2, bandwidth B1.

Target	|s|	Empirical Slope (SE)	Theoretical Slope	$R^{2}$
$D^{s}r_{n}^{\mathrm{IPW}}$	0	−0.678(0.019)	−0.800	0.998
$D^{s}r_{n}^{\mathrm{IPW}}$	1	−0.342(0.010)	−0.571	0.998
$D^{s}r_{n}^{\mathrm{IPW}}$	2	−0.165(0.006)	−0.444	0.997
$D^{s}f_{X;n}$	0	−0.708(0.008)	−0.800	0.9997
$D^{s}f_{X;n}$	1	−0.428(0.005)	−0.571	0.9997
$D^{s}f_{X;n}$	2	−0.298(0.006)	−0.444	0.9993

## Data Availability

The original contributions presented in this study are included in the article. Further inquiries can be directed to the corresponding authors.
